# National, regional, and worldwide estimates of low birthweight in 2015, with trends from 2000: a systematic analysis

**DOI:** 10.1016/S2214-109X(18)30565-5

**Published:** 2019-05-15

**Authors:** Hannah Blencowe, Julia Krasevec, Mercedes de Onis, Robert E Black, Xiaoyi An, Gretchen A Stevens, Elaine Borghi, Chika Hayashi, Diana Estevez, Luca Cegolon, Suhail Shiekh, Victoria Ponce Hardy, Joy E Lawn, Simon Cousens

**Affiliations:** a Maternal Adolescent Reproductive & Child Health (MARCH) Centre, London School of Hygiene & Tropical Medicine, London, UK; b Institute for Maternal and Child Health, IRCCS “Burlo Garofolo”, Trieste, Italy; c Local Health Unit N2, Public Health Department Treviso, Italy; d Department of Nutrition for Health and Development, World Health Organization, Geneva, Switzerland; e Department of Information Evidence and Research, World Health Organization, Geneva, Switzerland; f Data and Analytics, Division of Data, Research and Policy, UNICEF, NY, USA; g Institute for International Programs, Johns Hopkins Bloomberg School of Public Health, Johns Hopkins University, Baltimore, MD, USA

## Abstract

**Background:**

Low birthweight (LBW) of less than 2500 g is an important marker of maternal and fetal health, predicting mortality, stunting, and adult-onset chronic conditions. Global nutrition targets set at the World Health Assembly in 2012 include an ambitious 30% reduction in LBW prevalence between 2012 and 2025. Estimates to track progress towards this target are lacking; with this analysis, we aim to assist in setting a baseline against which to assess progress towards the achievement of the World Health Assembly targets.

**Methods:**

We sought to identify all available LBW input data for livebirths for the years 2000–16. We considered population-based national or nationally representative datasets for inclusion if they contained information on birthweight or LBW prevalence for livebirths. A new method for survey adjustment was developed and used. For 57 countries with higher quality time-series data, we smoothed country-reported trends in birthweight data by use of B-spline regression. For all other countries, we estimated LBW prevalence and trends by use of a restricted maximum likelihood approach with country-level random effects. Uncertainty ranges were obtained through bootstrapping. Results were summed at the regional and worldwide level.

**Findings:**

We collated 1447 country-years of birthweight data (281 million births) for 148 countries of 195 UN member states (47 countries had no data meeting inclusion criteria). The estimated worldwide LBW prevalence in 2015 was 14·6% (uncertainty range [UR] 12·4–17·1) compared with 17·5% (14·1–21·3) in 2000 (average annual reduction rate [AARR] 1·23%). In 2015, an estimated 20·5 million (UR 17·4–24·0 million) livebirths were LBW, 91% from low-and-middle income countries, mainly southern Asia (48%) and sub-Saharan Africa (24%).

**Interpretation:**

Although these estimates suggest some progress in reducing LBW between 2000 and 2015, achieving the 2·74% AARR required between 2012 and 2025 to meet the global nutrition target will require more than doubling progress, involving both improved measurement and programme investments to address the causes of LBW throughout the lifecycle.

**Funding:**

Bill & Melinda Gates Foundation, The Children's Investment Fund Foundation, United Nations Children's Fund (UNICEF), and WHO.

## Introduction

Low birthweight (LBW) is defined as a birthweight below 2500 g regardless of gestational age^1^ and is usually applied to livebirths only. LBW includes both appropriately grown preterm neonates (<37 completed weeks of gestation) and term and preterm growth-restricted neonates (<10th centile of weight for gestational age and sex) but remains an important public health indicator, especially in settings where accurate gestational age assessment is not possible.^2^ LBW is a substantial public health problem in every country, associated with a range of both short-term and long-term consequences affecting human capital.^3^ More than 80% of neonatal deaths are in LBW newborns, of which two thirds are preterm and one third are term small-for-gestational-age.^3^, ^4^, ^5^, ^6^ LBW newborns also have a higher risk of morbidity, stunting in childhood, and long-term developmental and physical ill health including adult-onset chronic conditions such as cardiovascular disease.^7^, ^8^, ^9^, ^10^ Factors influencing LBW include extremes of maternal age (especially younger than 16 years of age or older than 40 years), multiple pregnancy, obstetric complications, chronic maternal conditions (eg, hypertensive disorders of pregnancy), infections (eg, malaria), and nutritional status.^11^, ^12^, ^13^, ^14^ Other contributors include exposure to environmental factors, such as indoor air pollution, and tobacco and drug use.^15^

In 2012, the World Health Assembly (WHA) endorsed a Comprehensive Implementation Plan on Maternal, Infant and Young Child Nutrition, which specified six global nutrition targets, including a 30% reduction in the number of LBW livebirths between 2012 and 2025.^16^ LBW is thus a key indicator of progress towards the achievement of the global nutrition targets and monitoring LBW trends is an essential component of the Global Nutrition Monitoring Framework approved by member states at the WHA in May, 2015.^17^ These targets are reiterated in the Sustainable Development Goals (SDGs).

**Research in context**

**Evidence before this study**

Low birthweight (LBW; <2500 g), a composite measure of fetal growth and gestational length, is an important indicator of maternal and perinatal health and a predictor of adverse short-term and long-term health outcomes. LBW is a key outcome in global nutrition targets. However, LBW data from administrative data sources have not been systematically collated, existing methods for adjusting survey LBW data are recognised to have several limitations, and no standardised, systematic estimates for LBW prevalence have been produced.

**Added value of this study**

Through systematic searches (eg, of national statistical offices, ministry of health websites, and websites of the major household survey programmes of Multiple Indicator Cluster Surveys and Demographic and Health surveys), we compiled a global LBW dataset (1447 datapoints from 148 countries). New methods to adjust survey data were developed with UNICEF. We estimate that 20·5 million (uncertainty range 17·4–24·0) livebirths had a birthweight of less than 2500 g in 2015. Most (91%) were in low-income and middle-income countries, with nearly three-quarters in sub-Saharan Africa and southern Asia. Reported data from 57 mostly high-income countries with relatively low baseline suggests almost no change in LBW prevalence. For the remaining countries, we estimate a 17% reduction in LBW prevalence over the years 2000–15, most notably in the countries with the highest LBW prevalence in 2000. Globally, the annual rate of reduction in LBW from 2000 to 2015 was 1·23%.

**Implications of all the available evidence**

Data meeting inclusion criteria were available for three quarters of all UN member states, with survey data remaining the primary source in low-income and middle-income countries and administrative data the major source in high-income countries. Data adhering to the inclusion criteria were not available for 47 countries. Closing this data gap is an important priority. Data quality remains a concern, with evidence of missing birthweights and heaping. Our methods attempt to correct for heaping in survey data, but correction was not possible for administrative data. To increase data quality and availability, every newborn, whether live or stillborn, must be weighed, and data systems improved to capture the birthweight of every newborn, including those at home or in private facilities. Rates of LBW reduction worldwide will need to more than double to reach the annual rate of reduction of 2·74% required to meet the ambitious global nutrition target of 30% reduction of LBW by 2025. Action is required both to tackle the underlying causes of LBW and to improve the data.

Previously, it was estimated that there were 20·6 million LBW livebirths in the year 2000;^18^ however, there are no contemporary standardised worldwide, regional, and national estimates or systematic trend estimates for LBW, which are essential for tracking progress towards the global nutrition target. The LBW prevalence and trends presented here aim to fill this gap and assist in setting the baseline against which to assess progress towards the achievement of the WHA targets.

## Methods

### Overview

Our study was a systematic analysis of livebirth LBW input data from national administrative sources and nationally representative surveys. We sought to identify all available LBW input data for livebirths. We accessed data that met preset inclusion criteria, and implemented data preprocessing steps, including adjustments to raw data where applicable, to calculate an LBW prevalence from each datapoint—ie, the number of livebirths (regardless of the gestational age) with a birthweight of less than 2500 g divided by the total number of liveborn babies who are weighed or for whom a birthweight could be imputed. Finally, we estimated the LBW prevalence for 195 countries for the years 2000–15 and summed the results to obtain regional and global estimates. We report national-level estimates for 148 countries with data meeting our inclusion criteria. We present our methods in a manner that follows the Guidelines for Accurate and Transparent Health Estimates Reporting (GATHER) checklist, which promotes transparency, including the sharing of input data and modelling code (appendix).^16^

### Input data

**Figure 1:**
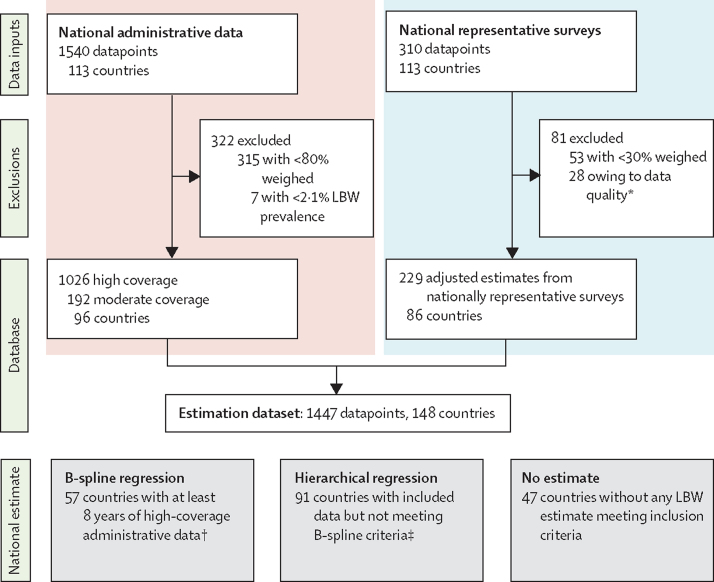
Administrative and survey data inputs and estimation methods LBW=low birthweight. *28 survey datasets were excluded on quality criteria: seven datasets were excluded because of extreme heaping around three values, nine because more than 10% of births weighed at least 4500 g, one because of excessive heaping on the tail end of the birthweight distribution, seven because of an inability to obtain results from adjustment procedures, and four because very low numbers of livebirths were weighed. †8 years of data between 2000 and 2015, with at least one datapoint before 2005 and one after 2010. ‡The estimate for India was based on partial data for the most recent survey; therefore, modelled estimates are not shown for individual country.

Figure 1 summarises the administrative and survey data inputs and estimation methods. We considered population-based national or nationally representative datasets for inclusion if they contained information on birthweight or LBW prevalence for livebirths (appendix). Nationally representative estimates of LBW prevalence can be derived from a range of sources, broadly defined as administrative data or representative household surveys. National administrative data are defined as data from national systems including Civil Registration and Vital Statistics (CRVS) systems, national Health Management Information Systems (HMISs), and birth registries. Nationally representative household surveys include Demographic and Health Surveys (DHSs), Multiple Indicator Cluster Surveys (MICSs), and other national surveys.

The optimal data source is a CRVS system that records details on all births, including their birthweight, on a continuous basis.^19^ Where all newborns are weighed accurately at birth, birthweight is recorded, registration is complete, and the system functions efficiently, the resulting LBW prevalence will be accurate and timely. However, existing administrative data systems might not cover all births, or might not collect birthweight data at all. In these settings, household surveys, such as the UNICEF-supported MICS and the USAID-supported DHS are important data sources for estimates of child health, including LBW, but are recognised to have biases. These data systems rely on accurate birthweight measurement, but despite increasing prevalence of facility births, many newborns are not weighed, and when weighed, so-called heaping at specific birthweights (eg, multiples of 100 g or 500 g) is common. We excluded subnational or other non-population-based data such as those from demographic surveillance sites and individual hospital data from the LBW data searches as they are rarely nationally representative.

To identify national administrative data, we searched the websites of the national statistical offices (NSOs) and ministries of health of all countries. Data from years 2000–16 were included. For countries with more than one source of national administrative data available for a given year, we gave preference to NSO website data where available. Where NSO data were unavailable, we used data obtained from the Ministry of Health website. We used WHO regional databases and a UNICEF database (TRANSOMNEE)^20^ to identify countries with national administrative data not retrieved through initial searches. These data were only included if they contained a reference to their source or could be verified as national administrative data from the NSO or Ministry of Health. Where necessary we contacted WHO and UNICEF regional and country offices to request further details of data sources.

We obtained datasets for all DHSs and MICSs with a midpoint of data collection of 1998 or later, and for which raw datasets were publicly available and contained birthweight data.^21^, ^22^, ^23^ A national team from the China Health Information and Statistics Center of the National Health Commission reanalysed data from the Chinese National Health Services Surveys. If data were available from both national administrative or nationally representative surveys for a given country, all data meeting the inclusion criteria were included in the database and subsequent modelling process.

Where no national administrative or nationally representative survey data were readily available through web-based searches, we contacted UNICEF and WHO regional and country offices in September–December, 2014, and again in autumn 2015 and invited them to provide details of any available national LBW data.

From October, 2017, to January, 2018, we did a joint WHO–UNICEF country consultation process to enable each country to provide feedback on the LBW input data used, modelling methods, and preliminary estimates for their country. We received further data from 55 countries through the consultation process, resulting in 341 new or updated country-year observations.

### Exclusions based on population representativeness at a national level

We excluded national administrative data covering less than 80% of the population, or from countries with less than 80% facility births in the data source year or less than 80% of the UN estimated livebirths in a given year. We also excluded survey data that were not nationally representative, as well as those with less than 30% weighed at birth. We applied a lower threshold for coverage of livebirths weighed to surveys (≥30%) compared with administrative data sources (≥80%) because raw data are available for surveys, allowing multiple imputation of missing birthweights by use of other covariates from the survey. This was not possible for data from administrative sources.

### Data quality assessment

We identified several potential sources of bias in LBW data sources (table 1). These include errors in birthweight measurement and recording (including heaping of recorded birthweights on 2500 g), misclassification between livebirths and stillbirths, missing birthweight data, and, for administrative data, non-representativeness at national level of births captured in the data system. Overall, these biases are likely to result in an underestimate of LBW prevalence. We took a two-step approach to seek to adjust for possible biases. First, we did a quality assessment of all the available data. Second, where possible, we adjusted included data.

**Table 1:** Potential sources of bias in low birthweight data

	Likely effect on LBW prevalence estimates*
**Coverage of weighing: bias in newborns weighed at birth**	
Many newborns in LMICs are not weighed at birth, especially if born at home. These are more likely to be socioeconomically disadvantaged and at higher risk of LBW.	↓
Extremely preterm or sick babies, those stillborn or dying soon after birth and those born around threshold of viability are the most likely to not be weighed. These babies are at high risk of being LBW.	↓
**Coverage of data system: bias in newborns included in data source**	
Low coverage of administrative data systems in many LMICs (eg, lower coverage of birth registration for those who die shortly after birth, missing home births, and births in private facilities even if weighed). Births in private facilities are more likely to be socioeconomically advantaged and at lower biological risk of LBW; however, high prevalence of medical interventions (eg, caesarean sections both indicated and elective before 37 weeks, may increase risk of LBW).	↓ or ↑
**Loss of birthweight data: biases in missing birthweight data**†	
In surveys, biases in card retention (eg, birthweight not available for babies who died who are more likely to have been LBW).	↓
Missing administrative birthweight data on sickest babies (frequently LBW) who are transferred immediately to (and weighed in) a newborn ward.	↓
**Measurement errors: individual measurement or recording error**	
Heaping of recording of birthweight on 2500 g. As definition excludes babies with birthweight exactly 2500 g, those LBW newborns with birthweight near the threshold frequently heaped at 2500 g.	↓
Errors in birthweight measurement (eg, poorly calibrated scales, inappropriate devices), suboptimal weighing practices (eg, clothed or delayed weighing until days after birth).	↓ or ↑
Extremely preterm or sick babies and those born around threshold of viability who die soon after birth are more likely to be misclassified as stillbirth. These babies are at high risk of being LBW.	↓
**Measurement units error**	
Confusion in surveys collecting data in both lbs and grams (eg, LBW baby weighing 4·0 lb recorded as 4·0 kg).	↓
**Denominator calculation errors in LBW prevalence calculation**	
LBW prevalence calculated as: number with birthweight <2500 per all livebirths (whether weighed or not).	↓

LBW=low birthweight.

* ↓=the potential bias is likely to lead to a decreased LBW prevalence. ↑=the potential bias is likely to lead to an increased LBW prevalence.

† For newborns who are both included in the data source and weighed at birth.

Raw individual-level data were available from household surveys as the datasets are in the public domain, allowing analysis of data quality and recording errors. We excluded surveys with inadequate data quality in three areas as follows. First, implausible birthweight distribution defined as extreme heaping whereby more than 55% of all birthweights in the dataset fall on only three values (eg, >55% of birthweights in the dataset were 2500 g, 3000 g, or 3500 g); more than 10% of births weighed at least 4500 g; or excessive heaping on the tail end of the birthweight distribution with more than 5% of birthweights at 250–500 g and 5500 g. Second, inability to obtain from adjustment procedures of multiple imputation or fitting of a mixture of two normal curves, or both. Third, data from surveys with very low numbers of livebirths weighed (<200) and hence high stochastic variation.

We made no further categorisation of data quality among included surveys. We made adjustments to the data from nationally representative household surveys by use of a revised methodology to seek to overcome the limitations of the previously used approach to address missing birthweights and heaping. We implemented a modelling approach that comprised multiple imputation with individually linked variables for all surveys (appendix). We replicated multiple imputations five times per survey and used several variables related to birthweight available in the survey datasets, including maternal factors (height, body-mass index [BMI], and parity), and newborn factors (sex, singleton–multiple status, and perceived size at birth).

To address heaping, we fitted a mixture of two normal distributions to each survey dataset. Whereas previous studies have found that, under ideal conditions such as low-risk full-term singleton livebirths included in the WHO child growth standards, birthweight is approximately normally distributed,^24^ this assumption might not apply to all national populations. We tested this assumption in an analysis of high-quality administrative data from the USA.^25^ Fitting a single normal distribution to this data from which the proportion of LBW could be estimated resulted in an overestimate of the proportion of livebirths with LBW compared with the raw data. This might indicate that the population of all births comprises two or more subpopulations with different distributions. Fitting a mixture of two normal distributions resulted in an estimated proportion of LBW very close to that seen in the raw data. We also investigated fitting a mixture of three normal distributions. However, this did not substantially improve the estimate of the proportion of LBW.

In summary, we estimated the proportion of LBW livebirths from each survey by the use of five steps. First, we developed five datasets that had a birthweight for each livebirth (reported where available or imputed). Second, we fitted two normal distributions to the datasets. Third, we calculated the LBW *Z* score for each of the two normal distributions:

$$Z_{2500}=\frac{2500 g-\text{mean birthweight}}{{\text{SD}}_{\text{birthweight}}}$$

Fourth, we estimated the percentage of LBW (LBW[%]) for each of the two distribution curves:

$$\text{LBW}(\%)=\text{P}(x<Z_{2500})$$

(ie, the percentage of the area under the curve <*Z*_2500_). Finally, we estimated the overall LBW prevalence by calculating the LBW(%) of the full dataset, which was a weighted average of the LBW(%) from both curves. The weights used were based on the proportion of the population estimated to belong to each subpopulation.

Since data from administrative data sources in the public domain usually only provide an aggregate number of LBW livebirths—ie, total livebirths or the reported LBW prevalence without individual-level data, or both—it was not possible to adjust LBW estimates to account for missing data and heaping in these data. To our knowledge, there are no previously used markers of data quality specifically for reported aggregated LBW prevalence. To assess and categorise the quality of available national level routine data, we reviewed previously used data quality criteria from other related maternal and perinatal global estimation exercises.^6^, ^26^, ^27^ Of these, only population representativeness, assessed by completeness of birthweight data, was feasible to apply (appendix). Datapoints from countries with less than 80% facility births or reporting a birthweight for less than 80% of the UN estimated livebirths in a given year were excluded. We further categorised included data into higher quality administrative data (data from countries with a facility birth prevalence ≥90% and with the data source covering ≥90% of UN estimated livebirths in the given year) and moderate quality administrative data (data from countries with a facility birth prevalence of at least 80% and with the data source covering at least 80% of UN estimated livebirths in the given year, not fulfilling higher quality criteria).

### Exclusions based on implausibility

We used conservative cutoffs to identify implausible LBW data. We excluded datapoints with an LBW prevalence of less than 2·1%, on the basis of the lowest population-based LBW prevalence in any country from the INTERGROWTH study.^28^ Since the INTERGROWTH study only included healthy women at low risk of pregnancy complications, including preterm birth and fetal growth restriction, the national LBW prevalence for all countries would be expected to be substantially higher than this cutoff. For example, the lowest national LBW prevalences from countries with strong national reporting systems are around 4%. The highest population-based LBW prevalence from any data source was 37%.^29^ We therefore decided to exclude datapoints with LBW prevalence greater than 40%; however, no datapoints were excluded on the basis of LBW prevalence of more than 40% (figure 1).

### Estimation of LBW prevalence by year and country

We defined higher quality time series administrative data for LBW prevalence as data from countries with the earliest year of data available before 2005, the latest year after 2010 with data available for at least half of all years, and no evidence of large year-on-year variability in LBW prevalence (coefficient of variation <15%). We estimated LBW prevalence for all other countries by means of a regression model. We modelled the logarithm of LBW prevalence as the outcome variable by use of restricted maximum likelihood estimation and including a country-level random effect.

We investigated multiple predictor variables associated with LBW, including distal determinants such as geographical and socioeconomic factors, more proximal demographic and biomedical factors, and markers of perinatal outcome (appendix). We included dummy variables in the model to account for systematic bias in different data types (higher quality national administrative data, moderate quality national administrative data, and nationally representative survey). We included all potential predictors with time series data or estimates available by country for 2000–15 in the model selection process (appendix).

We assessed correlations between predictors by use of the variance inflation factor. We dropped predictors with a variance inflation factor of greater than 10 as this is likely to indicate high correlation with other predictors. We retained predictors when the direction of the coefficient was biologically plausible. We sought to maximise the predictive power of the model, while avoiding overfitting. We removed one predictor at a time from the model, commencing with the predictor with the largest value of the Bayesian information criterion (BIC) on univariate analysis, and refitted the model. If removing this predictor improved the model (lower BIC compared with the model containing the predictor), we dropped the predictor from the model. If the BIC was higher, we retained the predictor. We cycled through all the predictors once.

The final model included the logarithm of neonatal mortality rate, the proportion of children underweight (below −2SDs from median weight for age of reference population), data type (higher quality administrative data, lower quality administrative data, household survey), UN region (southern Asia, sub-Saharan Africa or other region), and a country-specific random effect (table 2). We assessed model performance by use of diagnostic plots. The model seemed to fit the data reasonably well overall (*R*^2^ = 0·48), and both the estimates of the country-specific random effects (SD 0·31) and the residuals for the individual datapoints included (SD 0·11) appeared to be approximately normally distributed (appendix).

**Table 2:** Model coefficients for included predictor variables of low birthweight prevalence

		**Coefficient (95% CI)**
Neonatal mortality prevalence		0·009 (0·005 to 0·012)
Child underweight		0·615 (−0·031 to 1·260)
Region		
	Other regions	..
	Sub-Saharan Africa	0·300 (0·169 to 0·4)
	Southern Asia	0·6 (0·355 to 0·915)
Data type		
	High-quality administration data	..
	Moderate-quality administration data	−0·008 (−0·0 to 0·002)
	Nationally representative survey	0·165 (0·132 to 0·198)

..=baseline category.

For the 91 countries with data in the input dataset, we included the best linear unbiased prediction of the country-specific effect in the LBW prediction. For countries with no data, contributing only to the regional and global levels, we assumed the country random effect to be zero. We used high-quality national administrative data as the reference standard for prediction purposes for all countries in the higher-income regions (North America, Europe, and Australia and New Zealand). We used nationally representative household surveys as the reference for prediction purposes for countries from all other regions. We generated uncertainty ranges (URs) for modelled estimates by use of a bootstrap approach. When presenting by region we used an aggregate grouping of the United Nations regional subgroups (appendix). To obtain worldwide and regional estimates of uncertainty we summed the country LBW estimate at worldwide or regional level for each of the 1000 samples obtained from the bootstrap or B-spline approach and used the 2·5th and 97·5th centiles of the resulting distributions (appendix). Analyses were done with Stata 14.

**Figure 2:**
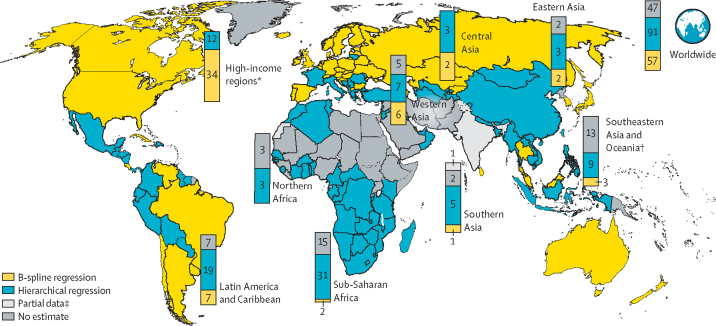
Low birthweight estimate methodology, by country (map) and region (bars), 2000–15 B-spline regression countries met criteria for minimum number of years of highly representative administrative estimates, hierarchical regression countries did not meet B-spline criteria but had at least one estimate meeting inclusion criteria; no estimate countries did not have any LBW estimate which met the inclusion criteria. See appendix for details. *High-income regions include North America, Europe, and Australia and New Zealand. †Southeast Asia and Oceania excluding Australia and New Zealand. ‡Estimate based on partial data for most recent survey; therefore, modelled estimates are not shown for the individual country.

We used livebirth estimates from the World Population Prospects: the 2017 revision^30^ to estimate the absolute number of LBW livebirths (livebirths × low birthweight rate) in a given year. LBW estimates generated from all 195 countries contributed to the regional and global estimates. National-level estimates are presented for the 57 countries with higher quality time series data and 91 other countries with at least one LBW prevalence datapoint since 2000 meeting the inclusion criteria (total 148 countries; figure 2; appendix). The modelled national-level estimate generated is not shown for 47 countries without any input data.

### Role of the funding source

The funders of the study had no role in the study design, data collection, data analysis, data interpretation, or writing of the report. HB had full access to all the data in the study and all authors had final responsibility for the decision to submit for publication.

## Results

Our final dataset was 1447 country-years of birthweight data (281 million births), comprised of 1026 high-coverage and 192 moderate-coverage datapoints from administrative data sources and data from 229 surveys (figure 1; table 3; appendix). Although data were available for 148 countries, most datapoints were categorised as national administrative data, predominantly from high (65%) or upper middle-income (28%) settings. The majority (54%) of LBW datapoints meeting inclusion criteria from low-income and lower middle-income settings were from household surveys. Countries from high-income regions had an average of 13 datapoints included compared with eight for upper-middle-income, four for lower-middle-income, and two for low-income regions (appendix). For 47 countries, no data fulfilling the inclusion criteria were located.

**Table 3:** Low birthweight prevalence input data by type

	**Number of data inputs**	**Number of livebirths included**	**Low birthweight prevalence**		
			Mean (SD)	Minimum	Maximum
Overall	1447	281 418 400	8·1% (3·9)	2·2%	32·9%
High-quality administrative data	1026	235 500 000	7·1% (2·5)	2·2%	17·6%
Moderate-quality administrative data	192	44 631 000	7·9% (3·1)	2·4%	15·7%
Nationally representative surveys	229	1 287 000	12·9% (5·6)	3·1%	32·9%

**Figure 3:**
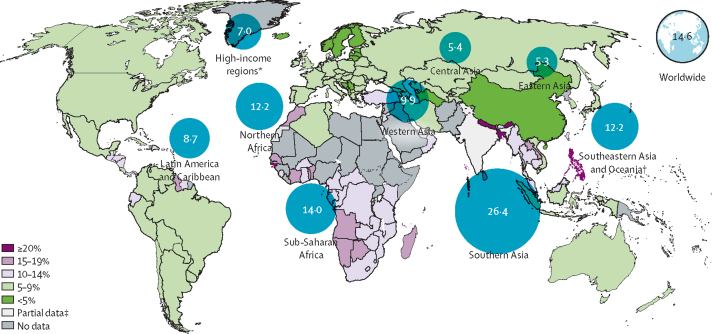
National and regional low birthweight prevalence, 2015 *High-income regions include North America, Europe and Australia and New Zealand. †Southeastern Asia and Oceania does not include Australia or New Zealand. ‡Estimate based on partial data for most recent survey; therefore, modelled estimates are not shown for the individual country.

**Figure 4:**
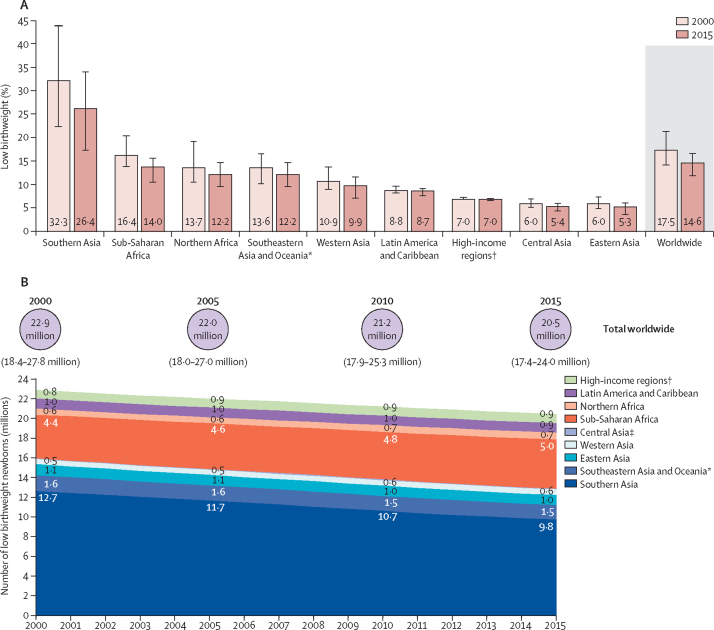
Regional and worldwide change in low birthweight between 2000 and 2015 (A) Changes in low birthweight rates. (B) Changes in absolute numbers of low birthweight newborns. *Southeastern Asia and Oceania does not include Australia or New Zealand. †High-income regions include North America, Europe, and Australia and New Zealand. ‡Central Asia labels not on graph due to space limitations, the number LBW is 0·1 million in all years.

We estimate that the global LBW prevalence in 2015 was 14·6% (UR 12·4–17·1), compared with 17·5% (14·1–21·3) in 2000 (table 4). This represents an estimated 16·6% decline in the LBW prevalence over this period (average annual rate of reduction [AARR] 1·23%). Although the uncertainty around these estimates is sizeable, they suggest some reduction in LBW prevalence over this time period. The highest burden of LBW is in the southern Asian, southeastern Asian, and sub-Saharan African regions (table 4; figure 3). The estimated rate of reduction in LBW prevalence is fastest in the regions with the highest baseline LBW prevalence and slowest in high-income regions and Latin America and the Caribbean (table 4; figure 4). In 2015, 85 of the 148 countries with data had an estimated LBW prevalence of less than 10%, whereas six countries were estimated to have LBW prevalence of at least 20% (appendix).

**Table 4:** Estimated low birthweight prevalence and number of low birthweight babies for 2000 and 2015, by region

	**2000**		**2015**		**Annual rate of reduction in low birthweight prevalence 2000–15**
	Low birthweight prevalence per 100 livebirths	Number of low birthweight newborns (UR)	Low birthweight prevalence per 100 livebirths	Number of low birthweight newborns (UR)	
North America, Europe, Australia, and New Zealand	7·0 (6·8–7·2)	832 900 (813 800–856 600)	7·0 (6·8–7·1)	884 400 (866 900–905 600)	0·01%
Northern Africa	13·7 (10·4–19·3)	602 400 (458 800–846 700)	12·2 (9·4–17·9)	712 600 (546 300–1 043 500)	0·77%
Sub-Saharan Africa	16·4 (13·8–20·4)	4 436 000 (3 729 700–5 499 000)	14·0 (12·2–17·2)	5 000 100 (4 349 600–6 146 300)	1·09%
Central Asia	6·0 (5·1–6·9)	71 700 (62 000–83 500)	5·4 (4.8-6.1)	85 500 (76 200–96 700)	0·71%
Southern Asia	32·3 (22·4–44·0)	12 694 600 (8 800 300–17 292 700)	26·4 (18·6–35·2)	9 807 400 (6 913 700–13 104 600)	1·37%
Eastern Asia	6·0 (4·9–7·4)	1 111 000 (900 100–1 364 100)	5·3 (4·3–6·6)	1 010 600 (822 600–1 264 800)	0·83%
Western Asia	10·9 (9·0–13·7)	532 300 (437 400–667 200)	9·9 (8·1–12·5)	560 200 (456 400–703 000)	0·63%
Southeast Asia and Oceania*	13·6 (10·1–16·6)	1 598 600 (1 190 300–1 947 200)	12·2 (9·5–14.6)	1 471 000 (1 151 700–1 763 800)	0·75%
Latin America and Caribbean	8·8 (8·1–9·6)	1 023 300 (945 800–1 113 500)	8·7 (8·1–9·6)	938 300 (871 500–1 032 100)	0·07%
Global	17·5 (14·1–21·3)	22 902 400 (18 405 800–27 798 400)	14·6 (12·4–17·1)	20 469 700 (17 375 000–24 017 900)	1·23%

* Excluding Australia and New Zealand.

The absolute number of livebirths with LBW globally is estimated at 20·5 million (UR 17·4–24·0) in 2015 compared with 22·9 million (18·4–27·8) in 2000 (figure 4). This represents a 10·6% decline in the point estimate against a 7·7% increase in the number of livebirths overall during this period. However, in some regions, despite reducing LBW prevalence, the overall estimated number of LBW livebirths has increased owing to demographic trends. In sub-Saharan Africa, the number of LBW livebirths is estimated to have increased from 4·4 million in 2000 to 5·0 million in 2015 (table 4). Southern Asia remains the region with the largest burden in terms of numbers, despite progress in reducing LBW prevalence (AARR 1·37%). An estimated 9·8 million LBW livebirths were born in this region in 2015—nearly half (48%) of the worldwide total.

## Discussion

We present global, regional, and national estimates for LBW with trend estimates, which are essential for tracking progress towards the Global Nutrition World Health Assembly target regarding LBW. Our estimates suggest that 20·5 million (UR 17·4–24·0) livebirths had a birthweight of less than 2500 g in 2015. Estimated progress in reducing LBW prevalence is slower than that required to meet the global nutrition target^16^—with an AARR of 1·23% between 2000 and 2015 compared with the required 2·74% between 2012 and 2025 to reach the target of a 30% reduction.

A strength of this work is that this LBW dataset is the largest compilation to date, including data from 148 countries and a more than 281 million births. In addition to the increased data quantity, we have applied new methods to adjust estimates on the basis of survey data that are more able to account for both data heaping and missing data. However, an important challenge is that almost half (48%) of all datapoints are from the high-income regions of North America, Europe, and Australia and New Zealand, which account for 4% of the world's LBW livebirths. By contrast, only 13% of data are from sub-Saharan Africa and southern Asia, the regions with the highest LBW prevalence, accounting for nearly three quarters of all LBW livebirths in 2015. 47 countries—the majority (87%) low-income or middle-income—that account for 23% of all births worldwide had no data meeting inclusion criteria. This is a classic example of the inverse data law—the least data for the highest burden settings.^31^ In addition, when available, these data tend to be lower quality with more heaping and other challenges, which probably lead to underestimates of LBW (table 1).

Regarding trends, no high-quality LBW trend data were available for 138 countries (91 with some LBW data meeting inclusion criteria and 47 without such data), and we therefore predicted LBW prevalence by use of a statistical model. The regions with the highest estimated change in LBW prevalence (and numbers) are sub-Saharan Africa and southern Asia, where the data are most uncertain and the estimated trends are driven by changes in predictors, which might not accurately reflect true changes in LBW prevalence over the same time period. Hence, it is plausible that the true change in prevalence for LBW worldwide is lower than our estimation of 1·23%, and the gap to reach the target is even greater.

The LBW data available from the highest burden settings are predominantly from household surveys and are susceptible to bias owing to missing birthweights and heaping. From 2004 to 2017, UNICEF used a simple cross tabulation to adjust for missing birthweight by use of data from a single variable (perceived size at birth), and a crude standard adjustment for heaping that assumed that 25% of birthweights reported as 2500 g were actually below 2500 g in every survey.^18^, ^32^ This previously used method had a number of important limitations.^33^ Hence, we used multiple imputation to impute missing birthweights. We used several variables including perceived size. We sought to address heaping throughout the birthweight distribution by fitting a mixture of two normal distributions to the observed data to obtain an estimate of the proportion of livebirths with a birthweight of less than 2500 g. Although we believe our approach represents an advance on the previous method, it does require assumptions—namely, that missing birthweights are missing at random and that the true distribution of birthweights in a population can be well approximated by a mixture of two normal distributions.

Although we were able to adjust for heaping in the survey data for which we had individual birthweight data, we were unable to do so for national administrative data sources for which such data were unavailable. This might lead to an underestimate of the LBW prevalence from these sources when LBW livebirths with birthweights of less than 2500 g are recorded as (heaped on) 2500 g and categorised as normal birthweight.

Global estimates have well recognised limitations,^34^ and investments in data systems are needed to improve multicountry tracking of progress towards global targets. Large countries, such as India, are taking steps to improve the data. However, ongoing efforts are required to support countries in strengthening their routine reporting systems to decrease missing and erroneous birthweight measurements as part of their commitment to report on the Global Nutrition Monitoring Framework and SDGs.^17^ Improving measurement of birthweight must occur alongside improvements in recording and reporting of all birth outcomes for mothers and their newborns, whether live or stillborn.^35^, ^36^ Challenges arising from the low quality of some data are compounded by absence of clear, internationally harmonised guidelines on how to assess LBW data quality.

More than 80% of all births worldwide are now in health facilities, yet despite this, most of the included datapoints from the highest burden regions are from household surveys, often with relatively low proportions having a reported birthweight. Improving the coverage and quality of birthweight data is crucial to drive actions to reduce LBW and will require action at many levels of the health system (table 5). Closing the gap between facility births and accurate birthweight recording should be feasible and would transform data availability. At the individual clinical level, appropriate equipment and trained staff are needed in both the public and private sectors. Weighing devices have been available since antiquity and routine birthweight measurement has been standard practice in Europe since the late 19th century; however, accurate information on birthweight is absent for most births worldwide. For example, heaping has been shown to be worse when analogue scales are used rather than digital ones and where scales with low precision are used.^37^, ^38^ There is a pressing need to develop affordable, robust, portable, and accurate weighing devices for use in both facility and community settings.

**Table 5:** Recommendations for improving birthweight data

	**Potential approaches**
**Ensure accurate birthweight for all births**	
Equipment	Improve availability and maintenance of suitable devices for birthweight measurement in all locations where births occur (facility or community). Establish minimum standards for equipment, including precision and scale type.
Training–human resources	Develop and disseminate protocols and guidelines. Preservice and in-service birthweight measurement training. Promote culture of weighing all babies (including the smallest and sickest). Identify and address barriers to weighing (eg, layout, staffing, etc). Improve awareness of clinical and public health importance of birthweight (eg, local data use in birthweight specific mortality).
**Ensure all birthweights captured in data systems**	
Data management	Standardise and streamline recording process for clinical staff, reduce repetitive recording.
Data coverage	Improve coverage of routine data systems in all facilities (including private) and timeliness of reporting. In settings with high rates of home birth, strengthen weighing in the community (eg, by CHW or TBA and link to health data system). Improve coverage of birth certificates and health cards including birthweight and motivate for birthweight to be included on all birth certificates.
**Maximise data quality**	
Data quality	Ensure minimum data collated (including number LBW, number weighed, number missing birthweight). Data quality checks and feedback as required. Correct data for heaping where required. Promote data literacy so that poor data are recognised and improved.
**Use data to inform policy**	
Data use	Improve timely data availability and use at local, district, and national level for policy, programming, and practice.

CHW=community health worker. TBA=traditional birth attendant.

Recording of birthweight data on health cards, which can be used as a data source at the time of the survey, could substantially improve the quality of survey birthweight data and reduce the need for adjustments (table 5).

The sickest and smallest newborns are often missing from the data systems, including those who die soon after birth, or are admitted to another ward. Data system improvements and linkages are required to capture information on these most vulnerable newborns.

Misclassification of early neonatal deaths as stillbirths remains an issue. Since these babies are more likely to be LBW, this can lead to an underestimate of LBW prevalence if stillbirths are excluded.^39^ Therefore, it is important that every newborn, whether live or stillborn, is weighed at birth and that core information including birthweight and gestational age is captured within the data system.

Societal and family demand for birthweight data is an understudied issue. Little is known about family and community perceptions and demand for birthweight measurement, including cultural barriers to birthweight measurement, especially in some community settings, and for stillbirths. Innovations that increase the value parents attach to birthweight data might help recall, and lead to improved recording on handheld health cards and birth certificates.

Birthweight reflects both intrauterine fetal growth and length of gestation. Assessing measures of weight for gestational age, for example small-for-gestational age, enables these two components to be distinguished. However, challenges in assessing gestational age accurately in many low-income and middle-income countries limit its use as a routine public health measure.^40^, ^41^ Debate has focused on the appropriateness of a single birthweight-for-gestational age cutoff for defining fetal growth restriction, with ethnic-specific standards associated with more accurate prediction of neonatal mortality and morbidity.^42^, ^43^, ^44^ Clear guidance on appropriate standards will be required as more data on gestational age become available at a national level worldwide, enabling tracking of fetal growth.

Reducing LBW requires a multifaceted approach.^46^ Even in the absence of accurate gestational age data at a national level, an understanding of the underlying pathways to LBW in a given setting is required to reduce the burden. For example, in southern Asia around half of LBW newborns are phenotypically term but small-for-gestational age, which is driven by underlying maternal undernutrition including maternal stunting.^4^ Conversely preterm birth is the major contributor to LBW in settings with many adolescent pregnancies or with high prevalence of infection (eg, in east and southern Africa) or where pregnancy is highly medicalised with high levels of fertility treatment and intensive obstetric management including high prevalence of caesarean sections (eg, the USA and Brazil).^47^ Improved birthweight data, coupled with high-quality data on gestational age, will be needed to target interventions appropriately and to track progress. Ongoing initiatives to improve CRVS and HMISs should be designed to ensure that this information is captured for all births.

We estimate that there were 20·5 million LBW livebirths in 2015 worldwide, nearly three quarters of them in southern Asia and sub-Saharan Africa. Progress in reducing LBW prevalence (AARR 1·23%) is insufficient to reach the global nutrition targets, which will require an AARR of 2·74%. Accurate birthweight data are needed for all births to improve both individual clinical care and public health action. There are large data gaps for the countries with the highest burden. In addition to better birthweight data, better gestational age assessment would help to identify the most appropriate interventions in a given setting. Targeted action to address the underlying causes of LBW (preterm birth and fetal growth restriction) and improved care for those born with LBW is needed to ensure that all realise their full potential to survive and thrive. In the SDG era, these most vulnerable babies must not be left behind.

## References

[1] WHO. International Classification of Diseases 10th revision (ICD-10). 2010. http://wwwwhoint/classifications/icd/ICD10Volume2_en_2010pdf?ua=1 (accessed Feb 2, 2018).

[2] Hughes MM, Black RE, Katz J. 2500-g low birth weight cutoff: history and implications for future research and policy. Matern Child Health J 2017; 21: 283–89. 10.1007/s10995-016-2131-9 PMC5290050 27449779

[3] Katz J, Lee AC, Kozuki N, et al. Mortality risk in preterm and small-for-gestational-age infants in low-income and middle-income countries: a pooled country analysis. Lancet 2013; 382: 417–25. 10.1016/S0140-6736(13)60993-9 PMC3796350 23746775

[4] Lee AC, Katz J, Blencowe H, et al. National and regional estimates of term and preterm babies born small for gestational age in 138 low-income and middle-income countries in 2010. Lancet Glob Health 2013; 1: e26–36. 10.1016/S2214-109X(13)70006-8 PMC4221634 25103583

[5] Lawn JE, Blencowe H, Oza S, et al. Every Newborn: progress, priorities, and potential beyond survival. Lancet 2014; 384: 189–205. 10.1016/S0140-6736(14)60496-7 24853593

[6] Blencowe H, Cousens S, Oestergaard MZ, et al. National, regional, and worldwide estimates of preterm birth rates in the year 2010 with time trends since 1990 for selected countries: a systematic analysis and implications. Lancet 2012; 379: 2162–72. 10.1016/S0140-6736(12)60820-4 22682464

[7] Blencowe H, Lee AC, Cousens S, et al. Preterm birth-associated neurodevelopmental impairment estimates at regional and global levels for 2010. Pediatr Res 2013; 74 (suppl 1): 17–34. 10.1038/pr.2013.204 PMC3873710 24366461

[8] Fall CH. Fetal malnutrition and long-term outcomes. Nestlé Nutr Inst Workshop Ser 2013; 74: 11–25. 10.1159/000348384 PMC5081104 23887100

[9] Christian P, Lee SE, Donahue Angel M, et al. Risk of childhood undernutrition related to small-for-gestational age and preterm birth in low- and middle-income countries. Int J Epidemiol 2013; 42: 1340–55. 10.1093/ije/dyt109 PMC3816349 23920141

[10] Gluckman PD, Hanson MA, Beedle AS. Early life events and their consequences for later disease: a life history and evolutionary perspective. Am J Human Biol 2007; 19: 1–19. 10.1002/ajhb.20590 17160980

[11] Pereira PP, Da Mata FA, Figueiredo AC, de Andrade KR, Pereira MG. Maternal active smoking during pregnancy and low birth weight in the Americas: a systematic review and meta-analysis. Nicotine Tob Res 2017; 19: 497–505. 10.1093/ntr/ntw228 28403455

[12] Accrombessi M, Zeitlin J, Massougbodji A, Cot M, Briand V. What do we know about risk factors for fetal growth restriction in Africa at the time of sustainable development goals? A scoping review. Paediatr Perinat Epidemiol 2018; 32: 184–96. 10.1111/ppe.12433 29253317

[13] Lean SC, Derricott H, Jones RL, Heazell AEP. Advanced maternal age and adverse pregnancy outcomes: a systematic review and meta-analysis. PLoS One 2017; 12: e0186287. 10.1371/journal.pone.0186287 PMC5645107 29040334

[14] Althabe F, Moore JL, Gibbons L, et al. Adverse maternal and perinatal outcomes in adolescent pregnancies: The Global Network's Maternal Newborn Health Registry study. Reprod Health 2015; 12 (suppl 2): S8. 10.1186/1742-4755-12-S2-S8 PMC4464033 26063350

[15] Amegah AK, Quansah R, Jaakkola JJ. Household air pollution from solid fuel use and risk of adverse pregnancy outcomes: a systematic review and meta-analysis of the empirical evidence. PLoS One 2014; 9: e113920. 10.1371/journal.pone.0113920 PMC4252082 25463771

[16] WHO. Comprehensive implementation plan on maternal, infant and young child nutrition. 2014. http://www.who.int/nutrition/publications/CIP_document/en/ (accessed Feb 2, 2018). 10.3945/an.114.007781 PMC4288273 25593153

[17] WHO. Global nutrition monitoring framework: operational guidance for tracking progress in meeting targets for 2025. 2017 http://wwwwhoint/nutrition/publications/operational-guidance-GNMF-indicators/en/ (accessed Feb 2, 2018).

[18] UNICEF, WHO. Low birthweight: country, regional and global estimates. 2004. https://wwwuniceforg/publications/index_24840html (accessed January, 2016).

[19] UN. Handbook on civil registration and vital statistics systems, management, operation and maintenance; studies in methods, Series F, No. 72 (Paragraph 22). United Nations: New York, 1998.

[20] UNICEF Regional Office for Eastern Europe and Central Asia. TransMonEE database on live births by weight. http://transmoneeorg/database/ (accessed June 3, 2017).

[21] DHS Program. Demographic and health surveys. http://www.dhsprogram.com/ (accessed Feb 27, 2018).

[22] Centers for Disease Control and Prevention. Reproductive Health Surveys. http://www.cdc.gov/reproductivehealth/global/tools/surveys.htm (accessed Feb 27, 2018).

[23] UNICEF. Multiple Indicator Surveys. http://mics.unicef.org/ (accessed Feb 27, 2018).

[24] WHO Multicentre Growth Reference Study Group; de Onis M, Garza C, Onyango A, Martorell R, eds. WHO child growth standards. Acta Paediatr 2006; 95 (suppl 450): 5–101.

[25] CDC National Center for Health Statistics. Birth data files. 2015. https://wwwcdcgov/nchs/data_access/Vitalstatsonlinehtm (accessed Dec 13, 2016).

[26] Blencowe H, Cousens S, Jassir FB, et al. National, regional, and worldwide estimates of stillbirth rates in 2015, with trends from 2000: a systematic analysis. Lancet Glob Health 2016; 4: e98–108. 10.1016/S2214-109X(15)00275-2 26795602

[27] WHO, UNICEF, UNFPA, The World Bank, and the United Nations Population Division. Trends in maternal mortality: 1990 to 2013. 2014. http://www.who.int/reproductivehealth/publications/monitoring/maternal-mortality-2013/en/ (accessed Feb 12, 2018).

[28] Villar J, Cheikh Ismail L, Victora CG, et al. International standards for newborn weight, length, and head circumference by gestational age and sex: the Newborn Cross-Sectional Study of the INTERGROWTH-21st Project. Lancet 2014; 384: 857–68. 10.1016/S0140-6736(14)60932-6 25209487

[29] UNICEF, Bangladesh Bureau of Statistics. National low birth weight survey of Bangladesh, 2003–04. 2005. Dhaka: Planning Division, Ministry of Planning, Government of Bangladesh.

[30] UN Population Division. World population prospects: the 2015 revision. 2017. https://population.un.org/wpp/ (accessed Dec 16, 2017).

[31] Lawn JE, Cousens S, Zupan J. 4 million neonatal deaths: when? Where? Why? Lancet 2005; 365: 891–900. 10.1016/S0140-6736(05)71048-5 15752534

[32] Blanc AK, Wardlaw T. Monitoring low birth weight: an evaluation of international estimates and an updated estimation procedure. Bull World Health Organ 2005; 83: 178–85. PMC2624216 15798841

[33] Channon AA, Padmadas SS, McDonald JW. Measuring birth weight in developing countries: does the method of reporting in retrospective surveys matter? Matern Child Health J 2011; 15: 12–18. 10.1007/s10995-009-0553-3 20063179

[34] Byass P. The imperfect world of global health estimates. PLoS Med 2010; 7: e1001006. 10.1371/journal.pmed.1001006 PMC2994666 21152416

[35] Moxon SG, Ruysen H, Kerber KJ, et al. Count every newborn; a measurement improvement roadmap for coverage data. BMC Pregnancy Childbirth 2015; 15 (suppl 2): S8. 10.1186/1471-2393-15-S2-S8 PMC4577758 26391444

[36] WHO. WHO technical consultation on newborn health indicators: Every Newborn Action Plan metrics. Geneva: World Health Organization, 2015.

[37] Mullany LC, Darmstadt GL, Katz J, Khatry SK, Tielsch JM. Effect of instrument precision on estimation of low birth weight prevalence. J Perinatol 2005; 25: 11–13. 10.1038/sj.jp.7211209 PMC1317297 15496868

[38] Sone T, Matsuda S, Doi T, Kahyo H. Digit preference in birth weight data of obstetric facilities. Nihon Eiseigaku Zasshi 1993; 47: 1050–57 (in Japanese). 10.1265/jjh.47.1050 8492482

[39] Liu L, Kalter HD, Chu Y, et al. Understanding misclassification between neonatal deaths and stillbirths: empirical evidence from Malawi. PloS One 2016; 11: e0168743. 10.1371/journal.pone.0168743 PMC5193424 28030594

[40] Chang KT, Mullany LC, Khatry SK, LeClerq SC, Munos MK, Katz J. Validation of maternal reports for low birthweight and preterm birth indicators in rural Nepal. J Glob Health 2018; 8: 010604. 10.7189/jogh.08.010604 PMC5997365 29899981

[41] Lee AC, Panchal P, Folger L, et al. Diagnostic accuracy of neonatal assessment for gestational age determination: a systematic review. Pediatrics 2017; 140. 10.1542/peds.2017-1423 29150458

[42] Anderson NH, Sadler LC, McKinlay CJD, McCowan LME. INTERGROWTH-21st vs customized birthweight standards for identification of perinatal mortality and morbidity. Am J Obstet Gynecol 2016; 214: 509.e1–7. 10.1016/j.ajog.2015.10.931 26546850

[43] Buck Louis GM, Grewal J, Albert PS, et al. Racial/ethnic standards for fetal growth: the NICHD Fetal Growth Studies. Am J Obstet Gynecol 2015; 213: 449.e1–41. 10.1016/j.ajog.2015.08.032 PMC4584427 26410205

[44] Hanley GE, Janssen PA. Ethnicity-specific birthweight distributions improve identification of term newborns at risk for short-term morbidity. Am J Obstet Gynecol 2013; 209: 428.e1–6. 10.1016/j.ajog.2013.06.042 23816839

[46] WHO. Global nutrition targets 2025: low birth weight policy brief. 2015. http://www.who.int/nutrition/publications/globaltargets2025_policybrief_lbw/en/ (accessed Oct 11, 2018).

[47] Lima MC, de Oliveira GS, Lyra Cde O, Roncalli AG, Ferreira MA. The spatial inequality of low birth weight in Brazil. Cien Saude Colet 2013; 18: 2443–52 (in Portuguese). 10.1590/s1413-81232013000800029 23896927

