# Supplementary material for: National, regional, and worldwide estimates of low birthweight in 2015, with trends from 2000: a systematic analysis
Source: Lancet Glob Health. 2019 May 15;7(7):e849–60. doi: 10.1016/S2214-109X(18)30565-5 (PMC6560046; doi:10.1016/S2214-109X(18)30565-5)
Supplement: Supplementary appendix [file mmc1.pdf]

THE LANCET
Global Health

Supplementary appendix

This appendix formed part of the original submission and has been peer reviewed. We post it as supplied by the authors.

Supplement to: Blencowe H, Krasevec J, de Onis M, et al. National, regional, and worldwide estimates of low birthweight in 2015, with trends from 2000: a systematic analysis. *Lancet Glob Health* 2019; published online May 15. http://dx.doi.org/10.1016/S2214-109X(18)30565-5.

National, regional, and worldwide estimates of low birthweight in 2015, with trends from 2000: a systematic analysis

Authors
Hannah Blencowe, Julia Krasevec, Mercedes de Onis, Robert E. Black, Xiaoyi An, Gretchen A. Stevens, Elaine Borghi, Chika Hayashi, Diana Estevez, Luca Cegolon, Suhail Shiekh, Victoria Ponce Hardy, Joy E Lawn*, Simon Cousens*

*joint senior authors

Contents

1. Guidelines for Accurate and Transparent Health Estimates Reporting 3
    Table 1.1: GATHER Checklist of information that should be included in reports of global health estimates. 3
2. Definitions and country groupings 5
    Table 2.1: Countries by United Nations sub-regions 5
  Source: United Nations 5
    Table 2.2: Aggregated UN sub-region groupings used in reporting these estimates 6
3. Data – inclusion criteria and adjustments 7
  Inclusion criteria 7
  Exclusion criteria 7
  Survey low birthweight estimate adjustments 7
4. Data - final inputs 8
    Table 4.1: Nationally-representative survey data meeting inclusion criteria (N=229) 8
    Table 4.2: Administrative data meeting inclusion criteria (N=1218) 13
    Figure 4.1: Summary of regional low birthweight rate data inputs meeting inclusion criteria 45
    Table 4.3: Summary of datapoints included by World Bank income region: 46
  Quality categorisation of administrative data 47
    Table 4.3: Summary of previously used and proposed quality criteria for the assessment of National Routine Data in global estimation exercises. 47
    Table 4.4: Countries with higher quality national routine data (N=57) 49
    Table 4.5: Summary of input data by data type 49
5. Predictor variables 50
  Identification of predictor variables for model input 50
    Table 5.1: Source of potential predictor variables tested in models 50
6. Model selection and estimation 51
    Table 6.1: Summary of model fitting process 51
  Final model equation 52

Figure 6.1: Diagnostic plots for the low birthweight prediction regression model 52
Table 6.2: Model coefficients 54
Calculation of low birthweight numbers 55
Uncertainty estimation 55
Annual Average Rate of Reduction (AARR) 55
7. Results 56
Table 7.1: Estimated low birthweight rate by country for 2000 and 2015 for 148 countries with data 56
Table 7.2: Estimated low birthweight number and rate globally and by region for low birthweight regions (2000-2015) 60
Figure 7.3: Graphs by country of data inputs and low birthweight rate estimates with associated uncertainty 64

1. Guidelines for Accurate and Transparent Health Estimates Reporting

Table 1.1: GATHER Checklist of information that should be included in reports of global health estimates.

Item #	Checklist item	Place Reported
**Objectives and funding**		
1	Define the indicator(s), populations (including age, sex, and geographic entities), and time period(s) for which estimates were made.	Paper page 3<br>Web appendix page 55
2	List the funding sources for the work.	Paper pages 2, 10
**Data Inputs**		
For all data inputs from multiple sources that are synthesized as part of the study:		
3	Describe how the data were identified and how the data were accessed.	Paper pages 4-5
4	Specify the inclusion and exclusion criteria. Identify all ad-hoc exclusions.	Paper pages 4-10<br>Web appendix pages 7
5	Provide information on all included data sources and their main characteristics. For each data source used, report reference information or contact name/institution, population represented, data collection method, year(s) of data collection, sex and age range, diagnostic criteria or measurement method, and sample size, as relevant.	Web appendix pages 8-50
6	Identify and describe any categories of input data that have potentially important biases (e.g., based on characteristics listed in item 5).	Table 1
For data inputs that contribute to the analysis but were not synthesized as part of the study:		
7	Describe and give sources for any other data inputs.	Web appendix page 50
For all data inputs:		
8	Provide all data inputs in a file format from which data can be efficiently extracted (e.g., a spreadsheet rather than a PDF), including all relevant meta-data listed in item 5. For any data inputs that cannot be shared because of ethical or legal reasons, such as third-party ownership, provide a contact name or the name of the institution that retains the right to the data.	Available on LSHTM data compass
**Data analysis**		
9	Provide a conceptual overview of the data analysis method. A diagram may be helpful.	Papers page 3
10	Provide a detailed description of all steps of the analysis, including mathematical formulae. This description should cover, as relevant, data cleaning, data pre-processing, data adjustments and weighting of data sources, and mathematical or statistical model(s).	Paper pages 4-10
11	Describe how candidate models were evaluated and how the final model(s) were selected.	Web appendix page 51-55
12	Provide the results of an evaluation of model performance, if done, as well as the results of any relevant sensitivity analysis.	Paper pages 8-9
13	Describe methods for calculating uncertainty of the estimates. State which sources of uncertainty were, and were not, accounted for in the uncertainty analysis.	Web appendix page 55
14	State how analytic or statistical source code used to generate estimates can be accessed.	Available on LSHTM data compass
**Results and Discussion**		
15	Provide published estimates in a file format from which data can be efficiently extracted.	Available on LSHTM data compass
16	Report a quantitative measure of the uncertainty of the estimates (e.g. uncertainty intervals).	Paper pages 10-11

**17**	Interpret results in light of existing evidence. If updating a previous set of estimates, describe the reasons for changes in estimates.	Paper pages 11-13
**18**	Discuss limitations of the estimates. Include a discussion of any modelling assumptions or data limitations that affect interpretation of the estimates.	Paper pages 11-12

Data source: Stevens et al[1]

2. Definitions and country groupings

Table 2.1: Countries by United Nations sub-regions

Source: United Nations

Africa

Western Africa	Eastern Africa	Middle Africa	Northern Africa	Southern Africa
Benin<br>Burkina Faso<br>Cabo Verde<br>Côte d'Ivoire<br>Gambia<br>Ghana<br>Guinea<br>Guinea-Bissau<br>Liberia<br>Mali<br>Mauritania<br>Niger<br>Nigeria<br>Senegal<br>Sierra Leone<br>Togo	Burundi<br>Comoros<br>Djibouti<br>Eritrea<br>Ethiopia<br>Kenya<br>Madagascar<br>Malawi<br>Mauritius<br>Mozambique<br>Rwanda<br>Seychelles<br>Somalia<br>South Sudan<br>Uganda<br>United Republic of Tanzania<br>Zambia<br>Zimbabwe	Angola<br>Cameroon<br>Central African Republic<br>Chad<br>Congo<br>Democratic Republic of the Congo<br>Equatorial Guinea<br>Gabon<br>Sao Tome and Principe	Algeria<br>Egypt<br>Libya<br>Morocco<br>Sudan<br>Tunisia	Botswana<br>Lesotho<br>Namibia<br>South Africa<br>Swaziland

Europe

Western Europe	Southern Europe	Northern Europe	Eastern Europe
Austria<br>Belgium<br>France<br>Germany<br>Liechtenstein<br>Luxembourg<br>Monaco<br>Netherlands<br>Switzerland	Albania<br>Andorra<br>Bosnia and Herzegovina<br>Croatia<br>Greece<br>Holy See<br>Italy<br>Malta<br>Montenegro<br>Portugal<br>San Marino<br>Serbia<br>Slovenia<br>Spain<br>The former Yugoslav Republic of Macedonia	Denmark<br>Estonia<br>Finland<br>Iceland<br>Ireland<br>Latvia<br>Lithuania<br>Norway<br>Sweden<br>United Kingdom	Belarus<br>Bulgaria<br>Czechia<br>Hungary<br>Poland<br>Republic of Moldova<br>Romania<br>Russian Federation<br>Slovakia<br>Ukraine

Latin America and the Caribbean

South America	Caribbean	Central America
Guyana<br>Suriname<br>Argentina<br>Bolivia (Plurinational State of)<br>Brazil<br>Chile<br>Colombia<br>Ecuador<br>Paraguay<br>Peru<br>Uruguay<br>Venezuela (Bolivarian Republic of)	Haiti<br>Antigua and Barbuda<br>Bahamas<br>Barbados<br>Cuba<br>Dominica<br>Dominican Republic<br>Grenada<br>Jamaica<br>Saint Kitts and Nevis<br>Saint Lucia<br>Saint Vincent and the Grenadines<br>Trinidad and Tobago	Belize<br>Costa Rica<br>El Salvador<br>Guatemala<br>Honduras<br>Mexico<br>Nicaragua<br>Panama

Asia

Western Asia	Southern Asia	South-Eastern Asia	Eastern Asia	Central Asia
Turkey<br>Yemen<br>Bahrain<br>Iraq<br>Jordan<br>Kuwait<br>Lebanon<br>Oman<br>Qatar<br>Saudi Arabia<br>State of Palestine<br>Syrian Arab Republic<br>United Arab Emirates<br>Cyprus<br>Israel<br>Azerbaijan<br>Armenia<br>Georgia	Afghanistan<br>Bangladesh<br>Maldives<br>Pakistan<br>Iran (Islamic Republic of)<br>Bhutan<br>Nepal<br>Sri Lanka<br>India	Brunei Darussalam<br>Indonesia<br>Malaysia<br>Cambodia<br>Myanmar<br>Lao People's Democratic Republic<br>Timor-Leste<br>Philippines<br>Singapore<br>Thailand<br>Viet Nam	Democratic People's Republic of Korea<br>Mongolia<br>Republic of Korea<br>China<br>Japan	Kazakhstan<br>Kyrgyzstan<br>Tajikistan<br>Turkmenistan<br>Uzbekistan

Oceania

Polynesia	Micronesia	Melanesia	Australia/New Zealand
Tuvalu<br>Cook Islands<br>Niue<br>Samoa<br>Tonga	Kiribati<br>Marshall Islands<br>Micronesia<br>Nauru<br>Palau	Solomon Islands<br>Vanuatu<br>Fiji<br>Papua New Guinea	Australia<br>New Zealand

Northern America

Northern America
United States of America<br>Canada

For the purposes of these estimates the UN sub-regions listed above were aggregated into 9 regional groups as shown in Table 2.2 below.

Table 2.2: Aggregated UN sub-region groupings used in reporting these estimates

**i)**	**ii)**	**iii)**	**iv)**	**v)**	**vi)**	**vii)**	**viii)**	**ix)**
United States of America<br>Canada<br>Australia<br>New Zealand<br>Albania<br>Andorra<br>Bosnia and Herzegovina<br>Croatia<br>Greece<br>Italy<br>Malta<br>Montenegro<br>Portugal<br>San Marino<br>Serbia<br>Slovenia<br>Spain<br>The former Yugoslav Republic of Macedonia<br>Holy See<br>Austria<br>Belgium<br>France<br>Germany<br>Liechtenstein<br>Luxembourg<br>Monaco<br>Netherlands<br>Switzerland<br>Denmark<br>Estonia<br>Finland<br>Iceland<br>Ireland<br>Latvia<br>Lithuania<br>Norway<br>Sweden<br>United Kingdom<br>Belarus<br>Bulgaria<br>Czechia<br>Hungary<br>Poland<br>Republic of Moldova<br>Romania<br>Russian Federation<br>Slovakia<br>Ukraine	Algeria<br>Egypt<br>Libya<br>Morocco<br>Sudan<br>Tunisia	**West**<br>Benin<br>Burkina Faso<br>Cabo Verde<br>Côte d'Ivoire<br>Gambia<br>Ghana<br>Guinea<br>Guinea-Bissau<br>Liberia<br>Mali<br>Mauritania<br>Niger<br>Nigeria<br>Senegal<br>Sierra Leone<br>Togo<br><br>**Middle**<br>Angola<br>Cameroon<br>Central African Republic<br>Chad<br>Congo<br>Democratic Republic of the Congo<br>Equatorial Guinea<br>Gabon<br>Sao Tome and Principe<br><br>**East**<br>Burundi<br>Comoros<br>Djibouti<br>Eritrea<br>Ethiopia<br>Kenya<br>Madagascar<br>Malawi<br>Mauritius<br>Mozambique<br>Rwanda<br>Seychelles<br>Somalia<br>South Sudan<br>Uganda<br>United Republic of Tanzania<br>Zambia<br>Zimbabwe<br><br>**South**<br>Botswana<br>Lesotho<br>Namibia<br>South Africa<br>Swaziland	Kazakhstan<br>Kyrgyzstan<br>Tajikistan<br>Turkmenistan<br>Uzbekistan	Afghanistan<br>Bangladesh<br>Maldives<br>Pakistan<br>Iran (Islamic Republic of)<br>Bhutan<br>Nepal<br>Sri Lanka<br>India	Democratic People's Republic of Korea<br>Mongolia<br>Republic of Korea<br>China<br>Japan	Turkey<br>Yemen<br>Bahrain<br>Iraq<br>Jordan<br>Kuwait<br>Lebanon<br>Oman<br>Qatar<br>Saudi Arabia<br>State of Palestine<br>Syrian Arab Republic<br>United Arab Emirates<br>Cyprus<br>Israel<br>Azerbaijan<br>Armenia<br>Georgia	Brunei Darussalam<br>Indonesia<br>Malaysia<br>Cambodia<br>Lao People's Democratic Republic<br>Myanmar<br>Timor-Leste<br>Philippines<br>Singapore<br>Thailand<br>Viet Nam<br>Tuvalu<br>Cook Islands<br>Niue<br>Samoa<br>Tonga<br>Kiribati<br>Marshall Islands<br>Micronesia (Federated States of)<br>Nauru<br>Palau<br>Solomon Islands<br>Vanuatu<br>Fiji Papua New Guinea	Belize<br>Costa Rica<br>El Salvador<br>Guatemala<br>Honduras<br>Mexico<br>Nicaragua<br>Panama<br>Haiti<br>Antigua and Barbuda<br>Bahamas<br>Barbados<br>Cuba<br>Dominica<br>Dominican Republic<br>Grenada<br>Jamaica<br>Saint Kitts and Nevis<br>Saint Lucia<br>Saint Vincent and the Grenadines<br>Trinidad and Tobago<br>Guyana<br>Suriname<br>Argentina<br>Bolivia (Plurinational State of)<br>Brazil<br>Chile<br>Colombia<br>Ecuador<br>Paraguay<br>Peru<br>Uruguay<br>Venezuela (Bolivarian Republic of)

i) North America, Europe, Australia and New Zealand
ii) Northern Africa
iii) Sub-saharan Africa (Southern/East/Middle/West Africa)
iv) Central Asia
v) Southern Asia
vi) Eastern Asia
vii) Western Asia
viii) South Eastern Asia and Oceania (without Australia and New Zealand)
ix) Latin America and Caribbean

3. Data – inclusion criteria and adjustments

Inclusion criteria

Population based national or nationally representative datasets containing information on birthweight or LBW rates without exclusion criteria and:

- For national routine data: a median year of birth from 2000 onwards.
- For household survey data a midpoint of data collection of 1998 or later, and for which raw datasets were publically available.

Exclusion criteria

- Sub-national or other non-population-based data such as those from demographic surveillance sites and individual hospital data.
- National administrative data covering less than 80% of the population, or from countries with less than 80% facility births in the data source year, or reporting a birthweight for less than 80% of the UN estimated livebirths in a given year.
- Survey data that were not nationally representative, or with less than 30% weighed at birth.
- Surveys assessed as inadequate data quality (see main paper for details).
- Data assessed to be implausible (see main paper for details).

Survey low birthweight estimate adjustments

Preparation of datasets: Birthweights reported to be <250g or >5500g were considered to be implausible and such births were treated as missing birthweights in the datasets for all analyses. Missing birthweights were imputed using the Multiple Imputation (MI) command in Stata. The imputation model included the following variables: (i) mother's perception of size at birth, and (ii) maternal parity for MICS and these same variables plus (iii) sex of child, (iv) multiple/singleton status, (v) maternal height and (vi) maternal body mass index (BMI) for DHS. Five imputations were performed for each survey.

Two normal distributions were fitted to each dataset, and the mean birthweight and standard deviation were calculated for each of these distributions. These were then used to calculation the LBW Z-score. The %LBW for each of the distributions was calculated as the % area under the curve < $Z_{2,500.}$ The overall %LBW for the dataset was calculated as the weighted average of %LBW from each distribution, based on the proportion of the population estimated to belong to each sub-population.

4. Data - final inputs

Table 4.1: Nationally-representative survey data meeting inclusion criteria (N=229[1])

Country	Data source	Year Published[1]	Number of births captured in the survey	Proportion of births weighed	LBW estimate from the survey
ALB	MICS	2000	254	0.87	4.0
ALB	MICS	2005	395	0.97	7.0
ALB	DHS	2008	1616	0.98	4.6
DZA	MICS	2012	5978	0.89	6.8
AGO	MICS	2001	1308	0.45	17.9
ARM	DHS	2000	1726	0.96	8.6
ARM	DHS	2005	1430	0.98	9.2
ARM	DHS	2010	1473	0.98	7.4
ARM	DHS	2015	1706	1	7.1
AZE	DHS	2006	2297	0.73	11.3
BGD	MICS	2013	7866	0.32	30.2
BLR	MICS	2005	1179	0.99	4.0
BLR	MICS	2012	1324	1	4.4
BLZ	MICS	2006	312	0.95	8.9
BEN	DHS	2001	5321	0.57	19.7
BEN	DHS	2006	16075	0.59	17.8
BEN	DHS	2011	13407	0.61	19.5
BEN	MICS	2014	5052	0.73	15.0
BTN	MICS	2010	2465	0.72	11.6
BOL	DHS	1998	7275	0.59	10.8
BOL	MICS	2000	876	0.72	5.4
BOL	DHS	2003	10417	0.6	9.3
BOL	DHS	2008	8605	0.71	7.4
BIH	MICS	2006	1174	0.99	4.9
BIH	MICS	2011	718	0.98	3.1
BFA	MICS	2006	2384	0.39	20.0
BFA	DHS	2010	15044	0.64	19.5
BDI	MICS	2000	882	0.39	18.0
BDI	DHS	2010	7742	0.57	15.5
KHM	DHS	2005	8290	0.39	16.3
KHM	DHS	2010	8232	0.72	12.8
KHM	DHS	2014	7153	0.91	11.7
CMR	DHS	1998	2297	0.51	11.3
CMR	MICS	2000	904	0.5	13.8
CMR	DHS	2004	8090	0.56	15.0
CMR	MICS	2006	2878	0.59	14.0
CMR	MICS	2014	2899	0.6	10.7
CAF	MICS	2000	3595	0.48	15.5
CAF	MICS	2006	4129	0.56	15.3
CAF	MICS	2010	4545	0.61	14.8
CHN	Survey	2008	7646	0.99	4.7
CHN	Survey	2013	11904	1	5.2
COL	DHS	2000	4659	0.73	9.1

[1] The 229 surveys include those from one country (India) for which only partial data were available for the most recent survey; and the individual adjusted survey estimates for this country are not shown in the table above

Country	Data source	Year Published[1]	Number of births captured in the survey	Proportion of births weighed	LBW estimate from the survey
COL	DHS	2005	14621	0.74	10.2
COL	DHS	2010	17756	0.76	11.6
COL	DHS	2015	11647	0.82	11.4
COM	MICS	2000	1231	0.44	30.2
COM	DHS	2012	3149	0.67	22.6
COG	DHS	2005	4835	0.85	13.9
COG	DHS	2011	9329	0.91	11.5
CRI	MICS	2011	854	0.98	7.4
CIV	DHS	1998	1991	0.54	20.5
CIV	MICS	2000	2139	0.71	19.8
CIV	MICS	2006	3711	0.59	19.2
CIV	DHS	2011	7776	0.6	19.2
CUB	MICS	2014	2193	0.95	6.2
COD	MICS	2001	2614	0.5	13.6
COD	DHS	2007	8992	0.68	12.0
COD	MICS	2010	4809	0.7	11.5
COD	DHS	2013	18640	0.76	9.6
DOM	DHS	1999	595	0.97	8.6
DOM	MICS	2000	435	0.95	15.3
DOM	DHS	2002	11201	0.98	9.9
DOM	DHS	2007	11149	0.96	9.6
DOM	DHS	2013	3708	0.98	15.9
DOM	MICS	2014	7566	0.96	12.4
SLV	MICS	2014	2832	0.93	9.8
GAB	DHS	2000	4405	0.88	15.1
GAB	DHS	2012	6067	0.91	15.1
GMB	DHS	2013	8088	0.59	17.7
GEO	MICS	2005	760	0.96	5.0
GHA	MICS	2006	1459	0.36	15.1
GHA	DHS	2008	2992	0.43	16.8
GHA	MICS	2011	2873	0.54	14.1
GHA	DHS	2014	5881	0.6	14.1
GTM	DHS	1999	4923	0.78	12.2
GTM	DHS	2015	12435	0.94	13.7
GNB	MICS	2006	2455	0.41	25.5
GNB	MICS	2014	3196	0.44	21.1
GUY	MICS	2000	497	0.79	15.1
GUY	MICS	2006	917	0.78	20.5
GUY	DHS	2009	2178	0.84	15.2
GUY	MICS	2014	1258	0.89	14.8
HND	DHS	2005	10800	0.69	11.3
HND	DHS	2011	10888	0.83	12.0
IDN	DHS	1997	17443	0.62	11.8
IDN	DHS	2002	16206	0.78	10.9
IDN	DHS	2007	18645	0.82	10.7
IDN	DHS	2012	17874	0.9	10.7
JAM	MICS	2005	538	0.97	16.2
JAM	MICS	2011	630	0.96	16.2
JOR	DHS	1997	6490	0.95	13.7
JOR	DHS	2002	6073	0.98	14.0
JOR	DHS	2007	10426	0.99	13.9

Country	Data source	Year Published[1]	Number of births captured in the survey	Proportion of births weighed	LBW estimate from the survey
JOR	DHS	2009	9650	0.99	14.7
JOR	DHS	2012	10360	0.99	16.9
KAZ	DHS	1999	1345	0.97	8.8
KAZ	MICS	2006	1784	0.99	5.8
KAZ	MICS	2010	2027	0.97	4.7
KAZ	MICS	2015	2106	0.99	4.6
KEN	DHS	1998	3511	0.45	13.6
KEN	DHS	2003	5949	0.44	12.2
KEN	DHS	2014	10048	0.66	10.5
KGZ	DHS	1997	1127	0.97	8.0
KGZ	MICS	2005	1152	0.97	6.3
KGZ	DHS	2012	4339	0.99	6.9
KGZ	MICS	2014	1766	0.98	5.5
LAO	MICS	2011	4444	0.42	19.1
LSO	MICS	2000	851	0.63	16.6
LSO	DHS	2004	3658	0.63	14.9
LSO	DHS	2009	3995	0.74	15.2
LSO	DHS	2014	3136	0.83	13.9
MDG	DHS	1997	3651	0.34	22.1
MDG	MICS	2000	1231	0.34	17.5
MDG	DHS	2003	5266	0.37	21.3
MDG	DHS	2008	12448	0.4	18.7
MWI	DHS	2000	11880	0.45	17.7
MWI	DHS	2004	10914	0.49	16.5
MWI	MICS	2006	10374	0.48	15.6
MWI	DHS	2010	19967	0.66	15.3
MWI	MICS	2013	7576	0.83	14.8
MWI	DHS	2016	17148	0.84	15.3
MDV	DHS	2009	3817	0.98	13.6
MEX	MICS	2015	3032	0.98	10.3
MNG	MICS	2000	1515	0.95	6.5
MNG	MICS	2005	1460	0.98	6.0
MNG	MICS	2010	1690	0.98	5.3
MNG	MICS	2014	2375	0.99	5.3
MNE	MICS	2005	351	0.96	4.0
MNE	MICS	2013	494	0.99	3.7
MAR	DHS	2003	6156	0.49	19.5
MOZ	DHS	1997	3999	0.4	20.7
MOZ	DHS	2003	10275	0.46	18.0
MOZ	DHS	2011	11102	0.51	20.6
MMR	DHS	2015	4813	0.45	12.7
NAM	DHS	2000	3911	0.68	15.8
NAM	DHS	2006	5064	0.77	16.9
NAM	DHS	2013	5013	0.86	16.3
NPL	DHS	2011	5306	0.36	19.9
NPL	MICS	2014	2086	0.59	28.7
NPL	DHS	2016	4994	0.61	20.4
NIC	DHS	1998	8273	0.75	12.0
NIC	DHS	2001	6918	0.71	11.9
PAN	MICS	2013	2278	0.91	8.9
PRY	MICS	2016	1745	0.97	10.0

Country	Data source	Year Published[1]	Number of births captured in the survey	Proportion of births weighed	LBW estimate from the survey
PER	DHS	2000	13658	0.7	11.5
PER	DHS	2004	17189	0.84	10.0
PER	DHS	2008	17189	0.84	10.0
PER	DHS	2009	10289	0.9	9.4
PER	DHS	2010	9281	0.9	10.1
PER	DHS	2011	9146	0.91	8.8
PER	DHS	2012	9620	0.93	9.3
PHL	DHS	1998	8083	0.59	19.0
PHL	DHS	2003	7095	0.68	21.2
PHL	DHS	2008	6572	0.73	21.9
PHL	DHS	2013	7216	0.81	25.5
MDA	DHS	2005	1552	0.99	6.5
MDA	MICS	2012	723	0.99	5.8
RWA	DHS	2005	8649	0.3	8.2
RWA	DHS	2010	9002	0.68	8.7
RWA	DHS	2014	7848	0.92	8.9
STP	DHS	2008	1931	0.82	8.8
STP	MICS	2014	758	0.93	8.6
SEN	DHS	1997	7341	0.35	19.7
SEN	DHS	2005	10887	0.47	22.9
SEN	DHS	2010	12326	0.61	22.0
SEN	DHS	2012	6862	0.54	20.6
SEN	DHS	2014	6842	0.52	19.4
SEN	DHS	2015	6935	0.55	18.6
SEN	DHS	2016	6725	0.53	16.7
SRB	MICS	2005	1445	0.98	4.9
SRB	MICS	2010	1187	1	5.9
SRB	MICS	2014	959	0.98	5.2
SLE	DHS	2008	5631	0.33	19.6
SLE	MICS	2010	3415	0.4	14.3
SLE	DHS	2013	11938	0.48	13.5
ZAF	DHS	1998	4942	0.7	14.7
SUR	MICS	2000	408	0.78	14.5
SUR	MICS	2006	798	0.74	11.9
SUR	MICS	2010	1265	0.81	15.6
SWZ	MICS	2000	807	0.72	13.8
SWZ	DHS	2006	2812	0.84	10.2
SWZ	MICS	2010	1018	0.91	10.5
SWZ	MICS	2014	987	0.91	8.8
TJK	MICS	2000	827	0.47	16.2
TJK	MICS	2005	1622	0.66	11.0
TJK	DHS	2012	4963	0.84	8.8
THA	MICS	2005	3365	0.99	10.2
THA	MICS	2012	2762	0.99	9.2
MKD	MICS	2005	1436	0.93	6.8
MKD	MICS	2011	503	0.96	6.8
TGO	MICS	2000	757	0.35	24.2
TGO	MICS	2006	1751	0.44	14.9
TGO	DHS	2013	6947	0.6	14.3
TTO	MICS	2006	415	0.9	16.6
TUN	MICS	2011	1135	0.97	7.2

Country	Data source	Year Published[1]	Number of births captured in the survey	Proportion of births weighed	LBW estimate from the survey
TUR	DHS	1998	3543	0.65	17.4
TUR	DHS	2003	4492	0.71	17.0
TKM	MICS	2015	1467	0.99	3.6
UKR	MICS	2005	1128	1	4.2
UKR	DHS	2007	1221	0.99	4.4
UKR	MICS	2012	1564	0.97	3.5
TZA	DHS	1999	3206	0.44	15.4
TZA	DHS	2004	8564	0.5	10.9
TZA	DHS	2010	8023	0.53	10.1
TZA	DHS	2015	10143	0.64	10.6
URY	MICS	2012	433	0.94	15.1
UZB	MICS	2000	686	0.94	6.7
UZB	MICS	2006	2095	0.99	5.4
VUT	MICS	2007	680	0.79	11.4
VNM	DHS	1997	1775	0.58	11.9
VNM	MICS	2000	550	0.7	11.6
VNM	DHS	2002	1316	0.8	10.2
VNM	MICS	2006	1023	0.87	7.5
VNM	MICS	2010	1363	0.93	7.0
VNM	MICS	2013	1484	0.94	7.1
PSE	MICS	2014	2891	1	8.9
ZMB	MICS	1999	1361	0.55	13.1
ZMB	DHS	2001	6857	0.42	14.5
ZMB	DHS	2007	6401	0.48	12.2
ZMB	DHS	2013	13399	0.66	11.7
ZWE	DHS	1999	3643	0.76	13.2
ZWE	DHS	2005	5246	0.72	12.7
ZWE	MICS	2006	2850	0.66	12.5
ZWE	DHS	2010	5563	0.69	13.3
ZWE	MICS	2014	3913	0.83	11.4
ZWE	DHS	2015	6075	0.82	12.8

[1] Year of publication is provided here. However for the purposes of estimation the median data year of the recall period is used e.g. for surveys with 3-year recall period the year of data collection, the data year used= (year of publication-1), for surveys with a 5-year recall the data year = (year of publication-2).

Table 4.2: Administrative data meeting inclusion criteria (N=1218)

ISO3 code	Data source	Year of data collection	Reported low birthweight rate	Number of weighed livebirths	National representativeness
ALB	National Statistical Office	2000	5.0	51250	Higher coverage
ALB	National Statistical Office	2001	4.6	53210	Higher coverage
ALB	National Statistical Office	2002	4.3	42530	Higher coverage
ALB	National Statistical Office	2003	4.8	45320	Higher coverage
ALB	National Statistical Office	2004	4.8	40990	Higher coverage
ALB	National Statistical Office	2005	3.7	38900	Higher coverage
ALB	National Statistical Office	2006	4.1	35900	Higher coverage
ALB	National Statistical Office	2007	4.3	34450	Higher coverage
ALB	National Statistical Office	2008	5.4	33450	Higher coverage
ALB	National Statistical Office	2009	4.0	34120	Higher coverage
ALB	National Statistical Office	2010	5.5	34070	Higher coverage
ALB	General Directory of Civil Status	2012	6.3	35480	Higher coverage
ALB	General Directory of Civil Status	2013	6.2	35750	Higher coverage
ALB	General Directory of Civil Status	2014	4.7	35760	Higher coverage
ALB	General Directory of Civil Status	2015	5.2	32720	Higher coverage
AND	National Statistical Office	2000	7.6	760	Higher coverage
AND	National Statistical Office	2001	7.7	760	Higher coverage
AND	National Statistical Office	2002	6.7	780	Higher coverage
AND	National Statistical Office	2003	8.5	750	Higher coverage
AND	National Statistical Office	2004	7.5	820	Higher coverage
AND	National Statistical Office	2005	6.6	820	Higher coverage
AND	National Statistical Office	2006	5.9	840	Higher coverage
AND	National Statistical Office	2007	6.3	820	Higher coverage
AND	National Statistical Office	2008	6.8	870	Higher coverage
AND	National Statistical Office	2009	9.9	840	Higher coverage
AND	National Statistical Office	2010	7.9	830	Higher coverage
AND	National Statistical Office	2011	9.0	800	Higher coverage
AND	National Statistical Office	2012	7.8	740	Higher coverage
ARE	Ministry of Health & Prevention	2015	11.5	89110	Higher coverage
ARG	National Statistical Office	2000	7.2	701880	Higher coverage
ARG	National Statistical Office	2001	7.4	683500	Higher coverage
ARG	National Statistical Office	2002	7.8	694690	Higher coverage
ARG	National Statistical Office	2003	8.0	697960	Higher coverage
ARG	National Statistical Office	2004	7.6	736270	Higher coverage
ARG	National Statistical Office	2005	7.3	712220	Higher coverage
ARG	National Statistical Office	2006	7.2	696460	Higher coverage
ARG	National Statistical Office	2007	7.2	700800	Higher coverage
ARG	National Statistical Office	2008	7.3	746460	Higher coverage
ARG	National Statistical Office	2009	7.1	745340	Higher coverage
ARG	National Statistical Office	2010	7.2	756180	Higher coverage

ISO3 code	Data source	Year of data collection	Reported low birthweight rate	Number of weighed livebirths	National representativeness
ARG	National Statistical Office	2011	7.2	758050	Higher coverage
ARG	National Statistical Office	2012	7.2	738320	Higher coverage
ARG	National Statistical Office	2013	7.4	754610	Higher coverage
ARG	National Statistical Office	2014	7.2	777020	Higher coverage
ARG	National Statistical Office	2015	7.2	770040	Higher coverage
ARM	Ministry of Health	2000	8.5	34280	Moderate coverage
ARM	Ministry of Health	2001	7.3	32070	Moderate coverage
ARM	Ministry of Health	2002	8.0	32230	Moderate coverage
ARM	Ministry of Health	2003	8.2	35800	Moderate coverage
ARM	Ministry of Health	2004	7.5	37520	Higher coverage
ARM	Ministry of Health	2005	7.3	37500	Moderate coverage
ARM	Ministry of Health	2006	7.7	37640	Moderate coverage
ARM	Ministry of Health	2007	7.3	40110	Higher coverage
ARM	Ministry of Health	2008	7.4	41190	Higher coverage
ARM	Ministry of Health	2009	7.5	44420	Higher coverage
ARM	Ministry of Health	2010	7.5	44830	Higher coverage
ARM	Ministry of Health	2011	7.7	43340	Higher coverage
ARM	Ministry of Health	2012	7.9	42480	Higher coverage
ARM	Ministry of Health	2013	8.1	41790	Higher coverage
ARM	Ministry of Health	2014	9.2	43070	Higher coverage
ARM	Ministry of Health	2015	8.6	41850	Higher coverage
ATG	Ministry of Health	2000	8.6	1520	Moderate coverage
ATG	Ministry of Health	2001	6.5	1480	Moderate coverage
ATG	Ministry of Health	2008	8.0	1440	Moderate coverage
ATG	Ministry of Health	2009	8.6	1420	Moderate coverage
AUS	Australia's mothers and babies	2000	6.3	255440	Higher coverage
AUS	Australia's mothers and babies	2001	6.2	252580	Higher coverage
AUS	Australia's mothers and babies	2002	6.4	253390	Higher coverage
AUS	Australia's mothers and babies	2003	6.3	255100	Higher coverage
AUS	Australia's mothers and babies	2004	6.4	255290	Higher coverage
AUS	Australia's mothers and babies	2005	6.4	270440	Higher coverage
AUS	Australia's mothers and babies	2006	6.4	280080	Higher coverage
AUS	Australia's mothers and babies	2007	6.2	292030	Higher coverage
AUS	Australia's mothers and babies	2008	6.1	294740	Higher coverage
AUS	Australia's mothers and babies	2009	6.2	296800	Higher coverage
AUS	Australia's mothers and babies	2010	6.2	297360	Higher coverage
AUS	Australia's mothers and babies	2011	6.3	299590	Higher coverage
AUS	Australia's mothers and babies	2012	6.2	309870	Higher coverage
AUS	Australia's mothers and babies	2013	6.4	307300	Higher coverage
AUS	Australia's mothers and babies	2014	6.4	310350	Higher coverage
AUS	Australia's mothers and babies	2015	6.5	306730	Higher coverage

ISO3 code	Data source	Year of data collection	Reported low birthweight rate	Number of weighed livebirths	National representativeness
AUT	National Statistical Office	2000	6.3	78270	Higher coverage
AUT	National Statistical Office	2001	6.7	75460	Higher coverage
AUT	National Statistical Office	2002	6.6	78400	Higher coverage
AUT	National Statistical Office	2003	7.1	76950	Higher coverage
AUT	National Statistical Office	2004	6.8	78970	Higher coverage
AUT	National Statistical Office	2005	6.8	78190	Higher coverage
AUT	National Statistical Office	2006	7.1	77920	Higher coverage
AUT	National Statistical Office	2007	7.2	76250	Higher coverage
AUT	National Statistical Office	2008	7.1	77760	Higher coverage
AUT	National Statistical Office	2009	7.1	76350	Higher coverage
AUT	National Statistical Office	2010	7.0	78750	Higher coverage
AUT	National Statistical Office	2011	6.9	78110	Higher coverage
AUT	National Statistical Office	2012	6.8	78960	Higher coverage
AUT	National Statistical Office	2013	6.8	79330	Higher coverage
AUT	National Statistical Office	2014	6.6	81730	Higher coverage
AUT	National Statistical Office	2015	6.5	84390	Higher coverage
AZE	National Statistical Office	2000	6.6	117000	Moderate coverage
AZE	National Statistical Office	2001	6.7	110360	Moderate coverage
AZE	National Statistical Office	2002	7.2	110720	Moderate coverage
AZE	National Statistical Office	2003	7.0	113470	Moderate coverage
AZE	National Statistical Office	2004	7.8	131610	Higher coverage
AZE	National Statistical Office	2005	7.7	141910	Higher coverage
AZE	National Statistical Office	2006	7.3	148950	Higher coverage
AZE	National Statistical Office	2007	7.2	151970	Higher coverage
AZE	National Statistical Office	2008	6.9	152090	Higher coverage
AZE	National Statistical Office	2009	7.1	152140	Higher coverage
AZE	National Statistical Office	2010	7.4	165650	Higher coverage
AZE	National Statistical Office	2011	7.3	176080	Higher coverage
AZE	National Statistical Office	2012	6.9	174470	Higher coverage
AZE	National Statistical Office	2013	7.0	172680	Higher coverage
BEL	National Statistical Office	2000	7.0	116400	Higher coverage
BEL	National Statistical Office	2001	7.3	115380	Higher coverage
BEL	National Statistical Office	2002	7.4	113100	Higher coverage
BEL	National Statistical Office	2003	7.4	114010	Higher coverage
BEL	National Statistical Office	2004	6.9	117300	Higher coverage
BEL	National Statistical Office	2005	7.0	119630	Higher coverage
BEL	National Statistical Office	2006	7.1	122530	Higher coverage
BEL	National Statistical Office	2007	6.8	124100	Higher coverage
BEL	National Statistical Office	2008	6.9	127210	Higher coverage
BEL	National Statistical Office	2009	7.0	127200	Higher coverage
BEL	National Statistical Office	2010	7.0	129180	Higher coverage

ISO3 code	Data source	Year of data collection	Reported low birthweight rate	Number of weighed livebirths	National representativeness
BEL	National Statistical Office	2011	6.8	127660	Higher coverage
BEL	National Statistical Office	2012	6.9	127000	Higher coverage
BEL	National Statistical Office	2013	7.1	124870	Higher coverage
BEL	National Statistical Office	2014	7.0	124420	Higher coverage
BFA	National Statistical Office	2011	9.7	561950	Moderate coverage
BFA	National Statistical Office	2012	9.9	610710	Moderate coverage
BFA	National Statistical Office	2013	10.3	593040	Moderate coverage
BFA	National Statistical Office	2014	9.9	662340	Higher coverage
BFA	National Statistical Office	2015	9.9	675070	Higher coverage
BGR	National Statistical Office	2000	8.5	73680	Higher coverage
BGR	National Statistical Office	2001	8.6	68180	Higher coverage
BGR	National Statistical Office	2002	8.7	66500	Higher coverage
BGR	National Statistical Office	2003	8.9	67360	Higher coverage
BGR	National Statistical Office	2004	8.6	69890	Higher coverage
BGR	National Statistical Office	2005	8.8	71080	Higher coverage
BGR	National Statistical Office	2006	9.3	73980	Higher coverage
BGR	National Statistical Office	2007	8.8	75350	Higher coverage
BGR	National Statistical Office	2008	8.5	77720	Higher coverage
BGR	National Statistical Office	2009	9.0	80960	Higher coverage
BGR	National Statistical Office	2010	9.5	75520	Higher coverage
BGR	National Statistical Office	2011	9.7	70850	Higher coverage
BGR	National Statistical Office	2012	9.4	69130	Higher coverage
BGR	National Statistical Office	2013	9.4	66580	Higher coverage
BGR	National Statistical Office	2014	9.8	67590	Higher coverage
BGR	National Statistical Office	2015	9.4	65950	Higher coverage
BHR	Ministry of Health	2000	8.7	14970	Higher coverage
BHR	Ministry of Health	2001	8.3	14970	Higher coverage
BHR	Ministry of Health	2002	8.1	14970	Higher coverage
BHR	Ministry of Health	2003	10.0	14970	Higher coverage
BHR	Ministry of Health	2004	8.4	14970	Higher coverage
BHR	Ministry of Health	2005	7.9	15200	Moderate coverage
BHR	Ministry of Health	2006	8.0	15060	Moderate coverage
BHR	Ministry of Health	2007	8.7	16070	Moderate coverage
BHR	Ministry of Health	2008	8.6	17030	Higher coverage
BHR	Ministry of Health	2009	9.8	17850	Higher coverage
BHR	Ministry of Health	2010	9.7	18020	Higher coverage
BHR	Ministry of Health	2011	10.0	17450	Moderate coverage
BHR	Ministry of Health	2012	9.8	19000	Higher coverage
BHR	Ministry of Health	2013	10.7	19880	Higher coverage
BHR	Ministry of Health	2014	11.3	20760	Higher coverage

ISO3 code	Data source	Year of data collection	Reported low birthweight rate	Number of weighed livebirths	National representativeness
BHS	Health Information and Research Unit consultation	2006	9.9	5300	Higher coverage
BHS	Health Information and Research Unit consultation	2007	10.8	5860	Higher coverage
BHS	Health Information and Research Unit consultation	2008	10.9	5480	Higher coverage
BHS	Health Information and Research Unit consultation	2009	12.3	5350	Higher coverage
BHS	Health Information and Research Unit consultation	2010	11.6	5050	Higher coverage
BHS	Health Information and Research Unit consultation	2011	11.5	5000	Higher coverage
BHS	Health Information and Research Unit consultation	2012	12.7	4870	Moderate coverage
BLR	National Statistical Office	2000	5.0	93700	Higher coverage
BLR	National Statistical Office	2001	5.2	91720	Higher coverage
BLR	National Statistical Office	2002	5.2	88750	Higher coverage
BLR	National Statistical Office	2003	5.4	88520	Higher coverage
BLR	National Statistical Office	2004	5.5	88950	Higher coverage
BLR	National Statistical Office	2005	5.4	90510	Higher coverage
BLR	National Statistical Office	2006	5.2	96730	Higher coverage
BLR	National Statistical Office	2007	5.1	103630	Higher coverage
BLR	National Statistical Office	2008	4.9	107880	Higher coverage
BLR	National Statistical Office	2009	5.0	109270	Higher coverage
BLR	National Statistical Office	2010	5.0	108050	Higher coverage
BLR	National Statistical Office	2011	5.1	109150	Higher coverage
BLR	National Statistical Office	2012	5.1	115900	Higher coverage
BLR	National Statistical Office	2013	5.0	118000	Higher coverage
BLR	National Statistical Office	2014	4.8	118540	Higher coverage
BRA	Ministry of Health	2000	7.7	3206770	Higher coverage
BRA	Ministry of Health	2001	8.0	3115480	Moderate coverage
BRA	Ministry of Health	2002	8.1	3059410	Moderate coverage
BRA	Ministry of Health	2003	8.3	3038260	Higher coverage
BRA	Ministry of Health	2004	8.2	3026550	Higher coverage
BRA	Ministry of Health	2005	8.1	3035100	Higher coverage
BRA	Ministry of Health	2006	8.2	2944930	Higher coverage
BRA	Ministry of Health	2007	8.2	2891330	Higher coverage
BRA	Ministry of Health	2008	8.3	2934830	Higher coverage
BRA	Ministry of Health	2009	8.4	2881590	Higher coverage
BRA	Ministry of Health	2010	8.4	2861870	Higher coverage
BRA	Ministry of Health	2011	8.5	2913160	Higher coverage
BRA	Ministry of Health	2012	8.5	2905790	Higher coverage
BRA	Ministry of Health	2013	8.5	2904030	Higher coverage

ISO3 code	Data source	Year of data collection	Reported low birthweight rate	Number of weighed livebirths	National representativeness
BRA	Ministry of Health	2014	8.4	2979260	Higher coverage
BRA	Sistema de Informações sobre Nascidos Vivos - SINASC	2015	8.3	3017670	Higher coverage
BRN	Health Information Booklet 2004	2000	10.0	6420	Moderate coverage
BRN	Health Information Booklet 2004	2001	10.4	6420	Higher coverage
BRN	Health Information Booklet 2004	2002	9.2	6420	Higher coverage
BRN	Health Information Booklet 2004	2003	7.0	6420	Higher coverage
BRN	Health Information Booklet 2008	2005	9.4	6420	Higher coverage
BRN	Health Information Booklet 2008	2006	11.0	6420	Higher coverage
BRN	Health Information Booklet 2008	2007	11.5	6420	Higher coverage
BRN	Health Information Booklet 2008	2008	11.1	6420	Higher coverage
BRN	Health Information Booklet 2009	2009	11.2	6420	Higher coverage
BRN	Health Information Booklet 2014	2010	10.8	6420	Higher coverage
BRN	Health Information Booklet 2014	2011	11.9	6730	Higher coverage
BRN	Health Information Booklet 2014	2012	12.1	6910	Higher coverage
BRN	Health Information Booklet 2014	2013	11.9	6680	Higher coverage
BRN	Health Information Booklet 2014	2014	11.9	6700	Higher coverage
BWA	National Statistical Office	2000	15.0	40640	Moderate coverage
BWA	National Statistical Office	2001	15.2	41080	Moderate coverage
BWA	National Statistical Office	2002	15.4	41860	Moderate coverage
BWA	National Statistical Office	2003	15.7	41210	Moderate coverage
BWA	National Statistical Office	2004	10.0	42180	Moderate coverage
BWA	National Statistical Office	2005	14.4	46080	Higher coverage
BWA	National Statistical Office	2006	12.4	44330	Higher coverage
BWA	National Statistical Office	2007	13.3	41140	Moderate coverage
BWA	National Statistical Office	2008	13.8	40880	Moderate coverage
BWA	National Statistical Office	2009	14.3	48710	Higher coverage
BWA	National Statistical Office	2010	12.5	48540	Higher coverage
CAN	National Statistical Office	2000	5.6	327890	Higher coverage
CAN	National Statistical Office	2001	5.5	333750	Higher coverage
CAN	National Statistical Office	2002	5.7	328810	Higher coverage

ISO3 code	Data source	Year of data collection	Reported low birthweight rate	Number of weighed livebirths	National representativeness
CAN	National Statistical Office	2003	5.9	335210	Higher coverage
CAN	National Statistical Office	2004	5.9	337080	Higher coverage
CAN	National Statistical Office	2005	6.0	342180	Higher coverage
CAN	National Statistical Office	2006	6.1	354620	Higher coverage
CAN	National Statistical Office	2007	6.0	367870	Higher coverage
CAN	National Statistical Office	2008	6.0	377890	Higher coverage
CAN	National Statistical Office	2009	6.1	380870	Higher coverage
CAN	National Statistical Office	2010	6.2	377220	Higher coverage
CAN	National Statistical Office	2011	6.1	377640	Higher coverage
CAN	National Statistical Office	2012	6.1	381870	Higher coverage
CAN	National Statistical Office	2013	6.3	380330	Higher coverage
CHE	National Statistical Office	2000	5.9	78460	Higher coverage
CHE	National Statistical Office	2001	6.2	72300	Higher coverage
CHE	National Statistical Office	2002	6.2	72380	Higher coverage
CHE	National Statistical Office	2003	6.2	71850	Higher coverage
CHE	National Statistical Office	2004	6.3	73090	Higher coverage
CHE	National Statistical Office	2005	6.2	72910	Higher coverage
CHE	National Statistical Office	2006	6.4	73380	Higher coverage
CHE	National Statistical Office	2007	6.4	74500	Higher coverage
CHE	National Statistical Office	2008	6.4	76700	Higher coverage
CHE	National Statistical Office	2009	6.6	78290	Higher coverage
CHE	National Statistical Office	2010	6.6	80290	Higher coverage
CHE	National Statistical Office	2011	6.4	80810	Higher coverage
CHE	National Statistical Office	2012	6.7	82170	Higher coverage
CHE	National Statistical Office	2013	6.6	82740	Higher coverage
CHE	National Statistical Office	2014	6.4	85290	Higher coverage
CHE	National Statistical Office	2015	6.5	86560	Higher coverage
CHL	National Statistical Office	2000	5.3	248900	Higher coverage
CHL	National Statistical Office	2001	5.3	246120	Higher coverage
CHL	National Statistical Office	2002	5.4	238990	Higher coverage
CHL	National Statistical Office	2003	5.6	234490	Higher coverage
CHL	Consultation	2004	5.6	230360	Higher coverage
CHL	National Statistical Office	2005	5.5	230840	Higher coverage
CHL	National Statistical Office	2006	5.8	231390	Higher coverage
CHL	National Statistical Office	2007	5.8	240570	Higher coverage
CHL	National Statistical Office	2008	5.9	246590	Higher coverage
CHL	National Statistical Office	2009	5.9	252240	Higher coverage
CHL	National Statistical Office	2010	5.9	250650	Higher coverage
CHL	National Statistical Office	2011	6.0	247360	Higher coverage
CHL	National Statistical Office	2012	6.0	243640	Higher coverage
CHL	National Statistical Office	2013	6.2	242010	Higher coverage

ISO3 code	Data source	Year of data collection	Reported low birthweight rate	Number of weighed livebirths	National representativeness
CHL	National Statistical Office	2014	6.2	251000	Higher coverage
COK	Consultation	2003	2.4	300	Moderate coverage
COK	Consultation	2004	3.2	320	Moderate coverage
COK	Consultation	2005	2.4	300	Moderate coverage
COK	Consultation	2007	4.4	320	Moderate coverage
COL	National Statistical Office	2000	7.7	752840	Moderate coverage
COL	National Statistical Office	2001	7.8	724320	Moderate coverage
COL	National Statistical Office	2002	7.9	700460	Moderate coverage
COL	National Statistical Office	2003	8.0	710710	Moderate coverage
COL	National Statistical Office	2004	8.1	723100	Moderate coverage
COL	National Statistical Office	2005	8.3	719970	Moderate coverage
COL	National Statistical Office	2006	8.6	714450	Moderate coverage
COL	National Statistical Office	2007	8.7	709260	Moderate coverage
COL	National Statistical Office	2008	8.9	715460	Moderate coverage
COL	National Statistical Office	2009	9.1	699780	Moderate coverage
COL	National Statistical Office	2010	9.1	654630	Moderate coverage
COL	National Statistical Office	2011	9.1	665500	Moderate coverage
COL	National Statistical Office	2012	9.0	676840	Moderate coverage
COL	National Statistical Office	2013	9.0	658840	Moderate coverage
COL	National Statistical Office	2014	9.0	669140	Moderate coverage
COL	National Statistical Office	2015	8.8	659260	Moderate coverage
CRI	National Statistical Office	2001	6.9	71150	Higher coverage
CRI	National Statistical Office	2003	6.9	72940	Higher coverage
CRI	National Statistical Office	2004	6.7	72250	Higher coverage
CRI	National Statistical Office	2005	6.8	71550	Higher coverage
CRI	National Statistical Office	2006	7.0	71300	Higher coverage
CRI	National Statistical Office	2007	7.0	73150	Higher coverage
CRI	National Statistical Office	2008	7.0	75190	Higher coverage
CRI	National Statistical Office	2009	6.9	75000	Higher coverage
CRI	National Statistical Office	2010	7.1	70930	Higher coverage
CRI	National Statistical Office	2011	7.2	73460	Higher coverage
CRI	National Statistical Office	2012	7.3	73330	Higher coverage
CRI	National Statistical Office	2013	7.4	70550	Higher coverage
CRI	National Statistical Office	2014	7.4	71800	Higher coverage
CRI	National Statistical Office	2015	7.5	71820	Higher coverage
CUB	National Statistical Office	2000	6.1	143530	Higher coverage
CUB	National Statistical Office	2001	5.9	138720	Higher coverage
CUB	National Statistical Office	2002	5.9	141280	Higher coverage
CUB	National Statistical Office	2003	5.5	136800	Higher coverage
CUB	National Statistical Office	2004	5.5	127200	Higher coverage
CUB	National Statistical Office	2005	5.4	120720	Higher coverage

ISO3 code	Data source	Year of data collection	Reported low birthweight rate	Number of weighed livebirths	National representativeness
CUB	National Statistical Office	2006	5.4	111330	Moderate coverage
CUB	National Statistical Office	2007	5.2	112480	Moderate coverage
CUB	National Statistical Office	2008	5.1	122570	Higher coverage
CUB	National Statistical Office	2009	5.1	130040	Higher coverage
CUB	National Statistical Office	2010	5.4	127750	Higher coverage
CUB	National Statistical Office	2011	5.3	133070	Higher coverage
CUB	National Statistical Office	2012	5.2	125680	Higher coverage
CUB	National Statistical Office	2013	5.1	125880	Higher coverage
CUB	National Statistical Office	2014	5.3	122650	Higher coverage
CUB	National Statistical Office	2015	5.3	125070	Higher coverage
CZE	National Statistical Office	2000	5.8	90910	Higher coverage
CZE	National Statistical Office	2001	6.0	90720	Higher coverage
CZE	National Statistical Office	2002	6.2	92790	Higher coverage
CZE	National Statistical Office	2003	6.6	93690	Higher coverage
CZE	National Statistical Office	2004	6.8	97670	Higher coverage
CZE	National Statistical Office	2005	6.7	102220	Higher coverage
CZE	National Statistical Office	2006	7.1	105840	Higher coverage
CZE	National Statistical Office	2007	7.4	114640	Higher coverage
CZE	National Statistical Office	2008	7.2	119570	Higher coverage
CZE	National Statistical Office	2009	7.6	118350	Higher coverage
CZE	National Statistical Office	2010	7.7	117160	Higher coverage
CZE	National Statistical Office	2011	7.6	108680	Higher coverage
CZE	National Statistical Office	2012	8.0	108580	Higher coverage
CZE	National Statistical Office	2013	8.1	106760	Higher coverage
CZE	National Statistical Office	2014	7.8	109860	Higher coverage
CZE	National Statistical Office	2015	7.8	110770	Higher coverage
DEU	National Statistical Office	2000	6.4	767000	Higher coverage
DEU	National Statistical Office	2001	6.6	734480	Higher coverage
DEU	National Statistical Office	2002	6.7	719250	Higher coverage
DEU	National Statistical Office	2003	6.8	706730	Higher coverage
DEU	National Statistical Office	2004	7.0	705630	Higher coverage
DEU	National Statistical Office	2005	6.8	685800	Higher coverage
DEU	National Statistical Office	2006	6.8	672730	Higher coverage
DEU	National Statistical Office	2007	6.9	684870	Higher coverage
DEU	National Statistical Office	2008	6.8	682520	Higher coverage
DEU	National Statistical Office	2009	6.9	665130	Higher coverage
DEU	National Statistical Office	2010	6.9	677950	Higher coverage
DEU	National Statistical Office	2011	6.9	662690	Higher coverage
DEU	National Statistical Office	2012	6.9	673550	Higher coverage
DEU	National Statistical Office	2013	6.6	682070	Higher coverage
DNK	National Statistical Office	2000	5.0	67060	Higher coverage

ISO3 code	Data source	Year of data collection	Reported low birthweight rate	Number of weighed livebirths	National representativeness
DNK	National Statistical Office	2001	5.4	65310	Higher coverage
DNK	National Statistical Office	2002	5.4	64480	Higher coverage
DNK	National Statistical Office	2003	5.4	64550	Higher coverage
DNK	National Statistical Office	2004	5.3	64590	Higher coverage
DNK	National Statistical Office	2005	5.2	64250	Higher coverage
DNK	National Statistical Office	2006	5.1	64940	Higher coverage
DNK	National Statistical Office	2007	5.3	64090	Higher coverage
DNK	National Statistical Office	2008	5.4	65040	Higher coverage
DNK	National Statistical Office	2009	5.3	62820	Higher coverage
DNK	National Statistical Office	2010	5.2	63410	Higher coverage
DNK	National Statistical Office	2011	5.5	59000	Higher coverage
DNK	National Statistical Office	2012	5.3	57920	Higher coverage
DNK	National Statistical Office	2013	5.2	55880	Higher coverage
DNK	National Statistical Office	2014	5.3	56870	Higher coverage
ECU	National Statistical Office	2009	9.6	332600	Higher coverage
ECU	National Statistical Office	2010	9.5	320630	Higher coverage
ECU	National Statistical Office	2011	9.5	328220	Higher coverage
ECU	National Statistical Office	2012	9.6	317350	Higher coverage
ECU	National Statistical Office	2013	9.7	292020	Moderate coverage
ECU	National Statistical Office	2014	9.5	285960	Moderate coverage
ECU	National Statistical Office	2015	9.8	283320	Moderate coverage
ESP	National Statistical Office	2000	6.9	397640	Higher coverage
ESP	National Statistical Office	2001	7.2	406380	Higher coverage
ESP	National Statistical Office	2002	7.5	418850	Higher coverage
ESP	National Statistical Office	2003	7.5	441890	Higher coverage
ESP	National Statistical Office	2004	7.4	454600	Higher coverage
ESP	National Statistical Office	2005	7.6	466380	Higher coverage
ESP	National Statistical Office	2006	7.5	482960	Higher coverage
ESP	National Statistical Office	2007	8.0	492530	Higher coverage
ESP	National Statistical Office	2008	8.0	519780	Higher coverage
ESP	National Statistical Office	2009	8.2	495000	Higher coverage
ESP	National Statistical Office	2010	8.1	486580	Higher coverage
ESP	National Statistical Office	2011	8.1	472000	Higher coverage
ESP	National Statistical Office	2012	8.2	454650	Higher coverage
ESP	National Statistical Office	2013	8.1	425720	Higher coverage
ESP	National Statistical Office	2014	8.2	427600	Higher coverage
ESP	National Statistical Office	2015	8.3	420290	Higher coverage
EST	National Statistical Office	2000	4.3	13060	Higher coverage
EST	National Statistical Office	2001	4.3	12630	Higher coverage
EST	National Statistical Office	2002	4.6	12990	Higher coverage
EST	National Statistical Office	2003	4.4	13020	Higher coverage

ISO3 code	Data source	Year of data collection	Reported low birthweight rate	Number of weighed livebirths	National representativeness
EST	National Statistical Office	2004	4.3	13980	Higher coverage
EST	National Statistical Office	2005	4.6	14340	Higher coverage
EST	National Statistical Office	2006	4.4	14870	Higher coverage
EST	National Statistical Office	2007	4.5	15800	Higher coverage
EST	National Statistical Office	2008	4.6	16050	Higher coverage
EST	National Statistical Office	2009	4.5	15800	Higher coverage
EST	National Statistical Office	2010	4.0	15930	Higher coverage
EST	National Statistical Office	2011	4.4	14890	Higher coverage
EST	National Statistical Office	2012	4.6	14370	Higher coverage
EST	National Statistical Office	2013	4.3	13940	Higher coverage
EST	National Statistical Office	2014	4.6	13790	Higher coverage
EST	National Statistical Office	2015	4.2	14060	Higher coverage
FIN	National Statistical Office	2000	4.3	56540	Higher coverage
FIN	National Statistical Office	2001	4.3	55790	Higher coverage
FIN	National Statistical Office	2002	4.3	55340	Higher coverage
FIN	National Statistical Office	2003	4.1	56450	Higher coverage
FIN	National Statistical Office	2004	4.2	57570	Higher coverage
FIN	National Statistical Office	2005	4.1	57640	Higher coverage
FIN	National Statistical Office	2006	4.3	58860	Higher coverage
FIN	National Statistical Office	2007	4.3	58730	Higher coverage
FIN	National Statistical Office	2008	4.1	59610	Higher coverage
FIN	National Statistical Office	2009	4.3	60590	Higher coverage
FIN	National Statistical Office	2010	4.3	61200	Higher coverage
FIN	National Statistical Office	2011	4.1	60100	Higher coverage
FIN	National Statistical Office	2012	4.1	59700	Higher coverage
FIN	National Statistical Office	2013	4.1	58380	Higher coverage
FIN	National Statistical Office	2014	4.2	57640	Higher coverage
FIN	National Statistical Office	2015	4.2	55590	Higher coverage
FRA	PMSI	2012	7.4	776070	Higher coverage
FRA	PMSI	2013	7.4	766050	Higher coverage
FRA	PMSI	2014	7.5	769120	Higher coverage
FRA	PMSI	2015	7.6	772520	Higher coverage
GBR	National Statistical Office	2000	7.3	NA	Higher coverage
GBR	National Statistical Office	2001	7.3	NA	Higher coverage
GBR	National Statistical Office	2002	7.4	NA	Higher coverage
GBR	National Statistical Office	2003	7.4	NA	Higher coverage
GBR	National Statistical Office	2004	7.3	NA	Higher coverage
GBR	National Statistical Office	2005	7.4	NA	Higher coverage
GBR	National Statistical Office	2006	7.5	NA	Higher coverage
GBR	National Statistical Office	2007	7.1	NA	Higher coverage
GBR	National Statistical Office	2008	7.1	NA	Higher coverage

ISO3 code	Data source	Year of data collection	Reported low birthweight rate	Number of weighed livebirths	National representativeness
GBR	National Statistical Office	2009	7.1	NA	Higher coverage
GBR	National Statistical Office	2010	7.0	NA	Higher coverage
GBR	National Statistical Office	2011	7.0	NA	Higher coverage
GBR	National Statistical Office	2012	7.0	NA	Higher coverage
GBR	National Statistical Office	2013	7.0	NA	Higher coverage
GBR	National Statistical Office	2014	6.9	NA	Higher coverage
GBR	National Statistical Office	2015	6.9	NA	Higher coverage
GEO	National Statistical Office	2000	5.9	48800	Moderate coverage
GEO	National Statistical Office	2001	6.4	47590	Moderate coverage
GEO	National Statistical Office	2002	6.3	46610	Moderate coverage
GEO	National Statistical Office	2003	6.8	46200	Moderate coverage
GEO	National Statistical Office	2004	5.5	49580	Moderate coverage
GEO	National Statistical Office	2005	6.0	46520	Moderate coverage
GEO	National Statistical Office	2006	5.3	47800	Moderate coverage
GEO	National Statistical Office	2007	5.3	49290	Moderate coverage
GEO	National Statistical Office	2008	4.7	56570	Higher coverage
GEO	National Statistical Office	2009	5.2	63380	Higher coverage
GEO	National Statistical Office	2010	4.7	62590	Higher coverage
GEO	National Statistical Office	2011	4.0	58020	Higher coverage
GEO	National Statistical Office	2012	5.4	57040	Higher coverage
GEO	National Statistical Office	2013	5.4	57880	Higher coverage
GEO	National Statistical Office	2014	5.6	60640	Higher coverage
GEO	National Statistical Office	2015	5.8	59250	Higher coverage
GRC	National Statistical Office	2000	8.1	103280	Higher coverage
GRC	National Statistical Office	2001	8.4	102290	Higher coverage
GRC	National Statistical Office	2002	8.3	103570	Higher coverage
GRC	National Statistical Office	2003	7.8	104420	Higher coverage
GRC	National Statistical Office	2004	8.6	105660	Higher coverage
GRC	National Statistical Office	2005	8.0	107550	Higher coverage
GRC	National Statistical Office	2006	8.3	112050	Higher coverage
GRC	National Statistical Office	2007	8.8	111930	Higher coverage
GRC	National Statistical Office	2008	8.4	118310	Higher coverage
GRC	National Statistical Office	2009	9.6	117940	Higher coverage
GRC	National Statistical Office	2010	10.0	114770	Higher coverage
GRC	National Statistical Office	2011	9.8	106430	Higher coverage
GRC	National Statistical Office	2012	9.8	100380	Higher coverage
GRC	National Statistical Office	2013	9.4	94140	Higher coverage
GRC	National Statistical Office	2014	9.4	92150	Higher coverage
GRC	National Statistical Office	2015	9.3	91850	Higher coverage
GTM	National Statistical Office	2009	8.4	351630	Moderate coverage
GTM	National Statistical Office	2010	8.2	361910	Moderate coverage

ISO3 code	Data source	Year of data collection	Reported low birthweight rate	Number of weighed livebirths	National representativeness
GTM	National Statistical Office	2011	8.9	373700	Higher coverage
GTM	National Statistical Office	2012	9.3	388620	Higher coverage
GTM	National Statistical Office	2013	9.5	387350	Higher coverage
GTM	National Statistical Office	2014	10.1	386200	Higher coverage
GTM	National Statistical Office	2015	10.4	391430	Higher coverage
HRV	Croatian Institute of Public Health	2000	5.1	44190	Higher coverage
HRV	Croatian Institute of Public Health	2001	5.6	41260	Higher coverage
HRV	Croatian Institute of Public Health	2002	5.7	40280	Higher coverage
HRV	Croatian Institute of Public Health	2003	5.4	39790	Higher coverage
HRV	Croatian Institute of Public Health	2004	5.0	40530	Higher coverage
HRV	Croatian Institute of Public Health	2005	4.8	42800	Higher coverage
HRV	Croatian Institute of Public Health	2006	4.8	41750	Higher coverage
HRV	Croatian Institute of Public Health	2007	5.0	42260	Higher coverage
HRV	Croatian Institute of Public Health	2008	4.6	44110	Higher coverage
HRV	Croatian Institute of Public Health	2009	5.3	44870	Higher coverage
HRV	Croatian Institute of Public Health	2010	4.8	43630	Higher coverage
HRV	Croatian Institute of Public Health	2011	5.1	41400	Higher coverage
HRV	Croatian Institute of Public Health	2012	4.9	41910	Higher coverage
HRV	Croatian Institute of Public Health	2013	4.7	40170	Higher coverage
HRV	Croatian Institute of Public Health	2014	5.0	39770	Higher coverage
HRV	Croatian Institute of Public Health	2015	5.1	37700	Higher coverage
HUN	National Statistical Office	2000	8.4	97600	Higher coverage
HUN	National Statistical Office	2001	8.5	97050	Higher coverage
HUN	National Statistical Office	2002	8.5	96810	Higher coverage
HUN	National Statistical Office	2003	8.7	94650	Higher coverage
HUN	National Statistical Office	2004	8.3	95140	Higher coverage
HUN	National Statistical Office	2005	8.2	97500	Higher coverage
HUN	National Statistical Office	2006	8.3	99880	Higher coverage
HUN	National Statistical Office	2007	8.2	97620	Higher coverage

ISO3 code	Data source	Year of data collection	Reported low birthweight rate	Number of weighed livebirths	National representativeness
HUN	National Statistical Office	2008	8.3	99150	Higher coverage
HUN	National Statistical Office	2009	8.4	96450	Higher coverage
HUN	National Statistical Office	2010	8.6	90340	Higher coverage
HUN	National Statistical Office	2011	8.5	88050	Higher coverage
HUN	National Statistical Office	2012	8.6	90270	Higher coverage
HUN	National Statistical Office	2013	8.8	88690	Higher coverage
HUN	National Statistical Office	2014	8.9	91510	Higher coverage
HUN	National Statistical Office	2015	8.5	91690	Higher coverage
IRL	National Statistical Office	2000	4.8	54790	Higher coverage
IRL	National Statistical Office	2001	5.1	57860	Higher coverage
IRL	National Statistical Office	2002	4.9	60510	Higher coverage
IRL	National Statistical Office	2003	5.0	61530	Higher coverage
IRL	National Statistical Office	2004	4.9	61980	Higher coverage
IRL	National Statistical Office	2005	4.9	61380	Higher coverage
IRL	National Statistical Office	2006	4.8	65430	Higher coverage
IRL	National Statistical Office	2007	5.1	71390	Higher coverage
IRL	National Statistical Office	2008	5.3	75180	Higher coverage
IRL	National Statistical Office	2009	4.9	75560	Higher coverage
IRL	National Statistical Office	2010	5.0	75180	Higher coverage
IRL	National Statistical Office	2011	5.2	74040	Higher coverage
IRL	National Statistical Office	2012	5.4	71680	Higher coverage
IRL	National Statistical Office	2013	5.6	68960	Higher coverage
IRL	National Statistical Office	2014	5.6	67300	Higher coverage
ISL	National Statistical Office	2000	3.7	4320	Higher coverage
ISL	National Statistical Office	2001	3.2	4100	Higher coverage
ISL	National Statistical Office	2002	3.9	4050	Higher coverage
ISL	National Statistical Office	2003	3.0	4150	Higher coverage
ISL	National Statistical Office	2004	3.5	4240	Higher coverage
ISL	National Statistical Office	2005	3.7	4280	Higher coverage
ISL	National Statistical Office	2006	3.9	4420	Higher coverage
ISL	National Statistical Office	2007	3.9	4560	Higher coverage
ISL	National Statistical Office	2008	3.8	4840	Higher coverage
ISL	National Statistical Office	2009	4.0	5030	Higher coverage
ISL	National Statistical Office	2010	3.6	4910	Higher coverage
ISL	National Statistical Office	2011	3.2	4500	Higher coverage
ISL	National Statistical Office	2012	4.2	4540	Higher coverage
ISL	National Statistical Office	2013	3.7	4330	Higher coverage
ISL	National Statistical Office	2014	4.0	4380	Higher coverage
ISL	National Statistical Office	2015	4.4	4130	Higher coverage
ISR	Public Health Service, Ministry of Health	2000	8.4	136490	Higher coverage

ISO3 code	Data source	Year of data collection	Reported low birthweight rate	Number of weighed livebirths	National representativeness
ISR	Public Health Service, Ministry of Health	2001	8.2	136710	Higher coverage
ISR	Public Health Service, Ministry of Health	2002	8.3	140090	Higher coverage
ISR	Public Health Service, Ministry of Health	2003	8.3	145400	Higher coverage
ISR	Public Health Service, Ministry of Health	2004	8.2	144400	Higher coverage
ISR	Public Health Service, Ministry of Health	2005	8.2	144010	Higher coverage
ISR	Public Health Service, Ministry of Health	2006	8.2	148250	Higher coverage
ISR	Public Health Service, Ministry of Health	2007	8.4	151640	Higher coverage
ISR	Public Health Service, Ministry of Health	2008	8.4	156870	Higher coverage
ISR	Public Health Service, Ministry of Health	2009	8.2	161010	Higher coverage
ISR	Public Health Service, Ministry of Health	2010	8.1	166300	Higher coverage
ISR	Public Health Service, Ministry of Health	2011	8.1	166540	Higher coverage
ISR	Public Health Service, Ministry of Health	2012	8.0	171060	Higher coverage
ISR	Public Health Service, Ministry of Health	2013	7.8	171430	Higher coverage
ISR	Public Health Service, Ministry of Health	2014	7.8	176420	Higher coverage
ISR	Public Health Service, Ministry of Health	2015	8.0	178750	Higher coverage
ITA	Ministry of Health	2003	6.7	457310	Moderate coverage
ITA	Ministry of Health	2004	6.7	479410	Moderate coverage
ITA	Ministry of Health	2005	6.8	509980	Higher coverage
ITA	Ministry of Health	2006	6.9	522830	Higher coverage
ITA	Ministry of Health	2007	6.7	520360	Higher coverage
ITA	Ministry of Health	2008	6.9	549150	Higher coverage
ITA	Ministry of Health	2009	7.0	553530	Higher coverage
ITA	Ministry of Health	2010	7.1	550980	Higher coverage
ITA	Ministry of Health	2011	7.1	538470	Higher coverage
ITA	Ministry of Health	2012	7.1	532480	Higher coverage
ITA	Ministry of Health	2013	7.2	509740	Higher coverage
ITA	Ministry of Health	2014	7.2	500280	Higher coverage
ITA	Ministry of Health	2015	7.4	484520	Higher coverage
JAM	Ministry of Health	2005	11.5	42290	Moderate coverage
JPN	National Statistical Office	2000	8.6	1190550	Higher coverage

ISO3 code	Data source	Year of data collection	Reported low birthweight rate	Number of weighed livebirths	National representativeness
JPN	National Statistical Office	2001	8.8	1170670	Higher coverage
JPN	National Statistical Office	2002	9.0	1153860	Higher coverage
JPN	National Statistical Office	2003	9.1	1123610	Higher coverage
JPN	National Statistical Office	2004	9.4	1110730	Higher coverage
JPN	National Statistical Office	2005	9.5	1062530	Higher coverage
JPN	National Statistical Office	2006	9.6	1092680	Higher coverage
JPN	National Statistical Office	2007	9.7	1089820	Higher coverage
JPN	National Statistical Office	2008	9.6	1091160	Higher coverage
JPN	National Statistical Office	2009	9.6	1070040	Higher coverage
JPN	National Statistical Office	2010	9.6	1071310	Higher coverage
JPN	National Statistical Office	2011	9.6	1050810	Higher coverage
JPN	National Statistical Office	2012	9.6	1037240	Higher coverage
JPN	National Statistical Office	2013	9.6	1029820	Higher coverage
JPN	National Statistical Office	2014	9.5	1003540	Higher coverage
JPN	National Statistical Office	2015	9.5	1005680	Higher coverage
KAZ	National Statistical Office	2000	5.9	222060	Higher coverage
KAZ	National Statistical Office	2001	5.3	221490	Higher coverage
KAZ	National Statistical Office	2002	6.1	227180	Higher coverage
KAZ	National Statistical Office	2003	5.5	247950	Higher coverage
KAZ	National Statistical Office	2004	5.2	273030	Higher coverage
KAZ	National Statistical Office	2005	4.8	278980	Higher coverage
KAZ	National Statistical Office	2006	5.0	301760	Higher coverage
KAZ	National Statistical Office	2007	5.2	321970	Higher coverage
KAZ	National Statistical Office	2008	6.1	357560	Higher coverage
KAZ	National Statistical Office	2009	6.1	356380	Higher coverage
KAZ	National Statistical Office	2010	6.1	367950	Higher coverage
KAZ	National Statistical Office	2011	6.1	372810	Higher coverage
KAZ	National Statistical Office	2012	6.1	381010	Higher coverage
KAZ	National Statistical Office	2013	5.9	387230	Higher coverage
KAZ	National Statistical Office	2014	5.8	399960	Higher coverage
KGZ	Ministry of Health	2004	6.2	103110	Higher coverage
KGZ	Ministry of Health	2005	6.2	109100	Higher coverage
KGZ	Ministry of Health	2006	6.3	111230	Higher coverage
KGZ	Ministry of Health	2007	6.0	122540	Higher coverage
KGZ	Ministry of Health	2008	5.9	130380	Higher coverage
KGZ	Ministry of Health	2009	5.8	137680	Higher coverage
KGZ	Ministry of Health	2010	5.7	141730	Higher coverage
KGZ	Ministry of Health	2011	5.6	142570	Higher coverage
KGZ	Ministry of Health	2012	5.7	147170	Higher coverage
KGZ	Ministry of Health	2013	5.7	151530	Higher coverage
KGZ	Ministry of Health	2014	5.7	158680	Higher coverage

ISO3 code	Data source	Year of data collection	Reported low birthweight rate	Number of weighed livebirths	National representativeness
KGZ	Ministry of Health	2015	5.4	160140	Higher coverage
KOR	National Statistical Office	2000	3.8	634510	Higher coverage
KOR	National Statistical Office	2001	4.0	554900	Higher coverage
KOR	National Statistical Office	2002	4.0	492120	Higher coverage
KOR	National Statistical Office	2003	4.1	490550	Higher coverage
KOR	National Statistical Office	2004	4.2	472770	Higher coverage
KOR	National Statistical Office	2005	4.3	435040	Higher coverage
KOR	National Statistical Office	2006	4.4	448160	Higher coverage
KOR	National Statistical Office	2007	4.7	493190	Higher coverage
KOR	National Statistical Office	2008	4.9	465900	Higher coverage
KOR	National Statistical Office	2009	5.0	444850	Higher coverage
KOR	National Statistical Office	2010	5.0	470180	Higher coverage
KOR	National Statistical Office	2011	5.2	471270	Higher coverage
KOR	National Statistical Office	2012	5.3	484550	Higher coverage
KOR	National Statistical Office	2013	5.5	436460	Higher coverage
KOR	National Statistical Office	2014	5.7	435440	Higher coverage
KOR	National Statistical Office	2015	5.7	438420	Higher coverage
KWT	National Statistical Office	2000	8.2	41850	Higher coverage
KWT	National Statistical Office	2001	8.3	41350	Higher coverage
KWT	National Statistical Office	2002	8.2	43490	Higher coverage
KWT	National Statistical Office	2003	8.2	43990	Higher coverage
KWT	National Statistical Office	2004	8.7	47280	Higher coverage
KWT	National Statistical Office	2005	8.5	50950	Higher coverage
KWT	National Statistical Office	2006	9.3	52760	Higher coverage
KWT	National Statistical Office	2007	9.7	53590	Higher coverage
LBN	National Statistical Office	2011	7.5	77240	Higher coverage
LBN	National Statistical Office	2012	7.5	79430	Higher coverage
LBN	National Statistical Office	2013	7.6	89380	Higher coverage
LBN	National Vital Data Observatory, MOPH	2014	8.1	105350	Higher coverage
LBN	National Vital Data Observatory, MOPH	2015	8.8	110420	Higher coverage
LKA	Routine hospital based information system	2000	16.7	339440	Higher coverage
LKA	Routine hospital based information system	2001	16.1	NA	Higher coverage
LKA	Routine hospital based information system	2002	17.1	NA	Higher coverage
LKA	Routine hospital based information system	2003	16.9	NA	Higher coverage
LKA	Routine hospital based information system	2005	17.6	339440	Higher coverage

ISO3 code	Data source	Year of data collection	Reported low birthweight rate	Number of weighed livebirths	National representativeness
LKA	Routine hospital based information system	2006	17.0	NA	Higher coverage
LKA	Routine hospital based information system	2007	17.3	NA	Higher coverage
LKA	Routine hospital based information system	2008	17.6	NA	Higher coverage
LKA	Routine hospital based information system	2009	17.2	339440	Higher coverage
LKA	Routine hospital based information system	2010	16.9	336770	Higher coverage
LKA	Routine hospital based information system	2011	16.6	340930	Higher coverage
LKA	Routine hospital based information system	2012	16.3	340800	Higher coverage
LKA	Routine hospital based information system	2013	16.7	347040	Higher coverage
LKA	Routine hospital based information system	2014	16.0	315230	Higher coverage
LKA	Routine hospital based information system	2015	16.0	330900	Higher coverage
LTU	Consultation	2000	4.6	33800	Higher coverage
LTU	Consultation	2001	4.5	31050	Higher coverage
LTU	Consultation	2002	4.9	29420	Higher coverage
LTU	Consultation	2003	4.7	29770	Higher coverage
LTU	Consultation	2004	4.5	29480	Higher coverage
LTU	Consultation	2005	4.7	29130	Higher coverage
LTU	Consultation	2006	4.6	29070	Higher coverage
LTU	Consultation	2007	4.5	29360	Higher coverage
LTU	Consultation	2008	4.6	31290	Higher coverage
LTU	Consultation	2009	4.5	32450	Higher coverage
LTU	Consultation	2010	4.7	30830	Higher coverage
LTU	Consultation	2011	4.7	28890	Higher coverage
LTU	Consultation	2012	4.8	28470	Higher coverage
LTU	Consultation	2013	4.6	27320	Moderate coverage
LTU	Consultation	2014	4.5	28070	Moderate coverage
LTU	Consultation	2015	4.5	28900	Higher coverage
LUX	SUSANA-database	2001	6.0	5500	Higher coverage
LUX	SUSANA-database	2002	6.1	5410	Higher coverage
LUX	SUSANA-database	2003	4.9	5330	Higher coverage
LUX	SUSANA-database	2004	6.3	5660	Higher coverage
LUX	SUSANA-database	2005	6.1	5630	Higher coverage
LUX	SUSANA-database	2006	5.7	5810	Higher coverage
LUX	SUSANA-database	2007	4.8	5740	Higher coverage
LUX	SUSANA-database	2008	6.5	6070	Higher coverage

ISO3 code	Data source	Year of data collection	Reported low birthweight rate	Number of weighed livebirths	National representativeness
LUX	SUSANA-database	2009	6.5	6170	Higher coverage
LUX	SUSANA-database	2010	6.7	6520	Higher coverage
LUX	SUSANA-database	2011	6.7	6320	Higher coverage
LUX	SUSANA-database	2012	6.5	6650	Higher coverage
LUX	SUSANA-database	2013	7.1	6790	Higher coverage
LUX	SUSANA-database	2014	6.6	6960	Higher coverage
LUX	SUSANA-database	2015	6.5	6840	Higher coverage
LVA	Centre for Disease Prevention and Control (CDPC) of Latvia, Medical Birth Register	2000	5.0	20220	Higher coverage
LVA	Centre for Disease Prevention and Control (CDPC) of Latvia, Medical Birth Register	2001	5.2	19610	Higher coverage
LVA	Centre for Disease Prevention and Control (CDPC) of Latvia, Medical Birth Register	2002	5.0	20000	Higher coverage
LVA	Centre for Disease Prevention and Control (CDPC) of Latvia, Medical Birth Register	2003	5.1	20990	Higher coverage
LVA	Centre for Disease Prevention and Control (CDPC) of Latvia, Medical Birth Register	2004	5.0	20360	Higher coverage
LVA	Centre for Disease Prevention and Control (CDPC) of Latvia, Medical Birth Register	2005	4.9	21540	Higher coverage
LVA	Centre for Disease Prevention and Control (CDPC) of Latvia, Medical Birth Register	2006	4.6	22300	Higher coverage
LVA	Centre for Disease Prevention and Control (CDPC) of Latvia, Medical Birth Register	2007	5.1	23280	Higher coverage
LVA	Centre for Disease Prevention and Control (CDPC) of Latvia, Medical Birth Register	2008	4.3	23970	Higher coverage
LVA	Centre for Disease Prevention and Control (CDPC) of Latvia, Medical Birth Register	2009	4.4	21680	Higher coverage
LVA	Centre for Disease Prevention and Control (CDPC) of Latvia, Medical Birth Register	2010	4.8	19140	Moderate coverage
LVA	Centre for Disease Prevention and Control (CDPC) of Latvia, Medical Birth Register	2011	4.7	18450	Moderate coverage
LVA	Centre for Disease Prevention and Control (CDPC) of Latvia, Medical Birth Register	2012	4.6	19550	Higher coverage

ISO3 code	Data source	Year of data collection	Reported low birthweight rate	Number of weighed livebirths	National representativeness
LVA	Centre for Disease Prevention and Control (CDPC) of Latvia, Medical Birth Register	2013	4.5	20260	Higher coverage
LVA	Centre for Disease Prevention and Control (CDPC) of Latvia, Medical Birth Register	2014	4.4	21450	Higher coverage
LVA	Centre for Disease Prevention and Control (CDPC) of Latvia, Medical Birth Register	2015	4.5	21720	Higher coverage
MCO	National Statistical Office	2003	6.0	830	Higher coverage
MCO	National Statistical Office	2004	4.7	820	Higher coverage
MCO	National Statistical Office	2005	5.7	890	Higher coverage
MCO	National Statistical Office	2006	5.1	880	Higher coverage
MCO	National Statistical Office	2007	5.0	930	Higher coverage
MCO	National Statistical Office	2008	4.0	970	Higher coverage
MCO	National Statistical Office	2009	5.5	1010	Higher coverage
MCO	National Statistical Office	2010	4.6	960	Higher coverage
MCO	National Statistical Office	2011	7.5	1040	Higher coverage
MCO	National Statistical Office	2012	6.3	980	Higher coverage
MCO	National Statistical Office	2013	6.0	1000	Higher coverage
MCO	National Statistical Office	2014	5.3	980	Higher coverage
MCO	National Statistical Office	2015	5.9	1070	Higher coverage
MDA	National Statistical Office	2002	5.1	35710	Moderate coverage
MDA	National Statistical Office	2003	5.3	36480	Moderate coverage
MDA	National Statistical Office	2004	4.8	38280	Moderate coverage
MDA	National Statistical Office	2005	4.6	37700	Moderate coverage
MDA	National Statistical Office	2006	4.9	37590	Moderate coverage
MDA	National Statistical Office	2007	5.0	37980	Moderate coverage
MDA	National Statistical Office	2008	5.2	39020	Moderate coverage
MDA	National Statistical Office	2009	5.2	40810	Higher coverage
MDA	National Statistical Office	2010	5.4	40480	Moderate coverage
MDA	National Statistical Office	2011	5.1	39190	Moderate coverage
MDA	National Statistical Office	2012	4.6	39440	Moderate coverage
MDA	National Statistical Office	2013	4.8	37880	Moderate coverage
MDA	National Statistical Office	2014	5.2	41210	Higher coverage
MDA	National Statistical Office	2015	5.4	41170	Higher coverage
MDV	Ministry of Health	2007	11.6	NA	Higher coverage
MDV	Ministry of Health	2008	9.9	6970	Higher coverage
MDV	Ministry of Health	2009	9.8	7430	Higher coverage
MDV	Ministry of Health	2010	11.2	7090	Higher coverage
MDV	Ministry of Health	2011	10.2	7190	Higher coverage
MDV	Ministry of Health	2012	9.8	7140	Higher coverage

ISO3 code	Data source	Year of data collection	Reported low birthweight rate	Number of weighed livebirths	National representativeness
MDV	istrative data	2014	9.5	7250	Higher coverage
MEX	National Statistical Office	2008	8.2	1978380	Moderate coverage
MEX	National Statistical Office	2009	8.5	2058710	Moderate coverage
MEX	National Statistical Office	2010	8.5	2073120	Moderate coverage
MEX	National Statistical Office	2011	5.7	2167060	Higher coverage
MEX	National Statistical Office	2012	5.8	2206700	Higher coverage
MEX	National Statistical Office	2013	5.9	2195080	Higher coverage
MEX	National Statistical Office	2014	6.1	2177320	Higher coverage
MEX	National Statistical Office	2015	6.1	2145200	Higher coverage
MKD	National Statistical Office	2000	9.4	29310	Higher coverage
MKD	National Statistical Office	2001	8.1	27010	Higher coverage
MKD	National Statistical Office	2002	8.8	27770	Higher coverage
MKD	National Statistical Office	2003	9.4	27020	Higher coverage
MKD	National Statistical Office	2004	8.1	23370	Higher coverage
MKD	National Statistical Office	2005	8.8	22490	Higher coverage
MKD	National Statistical Office	2006	9.4	22590	Higher coverage
MKD	National Statistical Office	2007	8.1	22690	Higher coverage
MKD	National Statistical Office	2008	8.8	22950	Higher coverage
MKD	National Statistical Office	2009	9.4	23690	Higher coverage
MKD	National Statistical Office	2010	8.1	24300	Higher coverage
MKD	National Statistical Office	2011	8.8	22770	Higher coverage
MKD	National Statistical Office	2012	9.4	23570	Higher coverage
MKD	National Statistical Office	2013	8.1	23140	Higher coverage
MKD	National Statistical Office	2014	8.8	23600	Higher coverage
MKD	National Statistical Office	2015	9.4	23080	Higher coverage
MLT	National obstetric info system	2000	5.9	4370	Higher coverage
MLT	National obstetric info system	2001	5.9	3940	Higher coverage
MLT	National obstetric info system	2002	5.9	3910	Higher coverage
MLT	National obstetric info system	2003	7.1	4040	Higher coverage
MLT	National obstetric info system	2004	7.7	3890	Higher coverage
MLT	National obstetric info system	2005	6.5	3860	Higher coverage
MLT	National obstetric info system	2006	6.4	3880	Higher coverage
MLT	National obstetric info system	2007	6.4	3890	Higher coverage
MLT	National obstetric info system	2008	6.8	4200	Higher coverage
MLT	National obstetric info system	2009	7.3	4160	Higher coverage
MLT	National obstetric info system	2010	7.2	4020	Higher coverage
MLT	National obstetric info system	2011	7.8	4290	Higher coverage
MLT	National obstetric info system	2012	7.0	4240	Higher coverage
MLT	National obstetric info system	2013	6.6	4130	Higher coverage
MLT	National obstetric info system	2014	6.5	4310	Higher coverage
MLT	National obstetric info system	2015	6.3	4440	Higher coverage

ISO3 code	Data source	Year of data collection	Reported low birthweight rate	Number of weighed livebirths	National representativeness
MNE	Institute of Public Health	2000	5.1	8840	Higher coverage
MNE	Institute of Public Health	2001	4.8	8500	Higher coverage
MNE	Institute of Public Health	2002	4.9	8350	Higher coverage
MNE	Institute of Public Health	2003	4.9	7850	Higher coverage
MNE	Institute of Public Health	2004	4.8	7360	Moderate coverage
MNE	Institute of Public Health	2005	4.9	7540	Higher coverage
MNE	Institute of Public Health	2006	4.2	7840	Higher coverage
MNE	Institute of Public Health	2007	3.9	8260	Higher coverage
MNE	Institute of Public Health	2008	4.4	8650	Higher coverage
MNE	Institute of Public Health	2009	5.1	7420	Higher coverage
MNE	Institute of Public Health	2010	5.2	7800	Higher coverage
MNE	Institute of Public Health	2011	5.6	7570	Higher coverage
MNE	Institute of Public Health	2012	5.1	7610	Higher coverage
MNE	Institute of Public Health	2013	5.0	6940	Higher coverage
MNE	Institute of Public Health	2014	5.5	7410	Higher coverage
MNE	Institute of Public Health	2015	5.4	7360	Higher coverage
MUS	National Statistical Office	2000	12.9	16780	Moderate coverage
MUS	National Statistical Office	2001	12.9	16780	Moderate coverage
MUS	National Statistical Office	2002	13.0	16780	Moderate coverage
MUS	National Statistical Office	2003	13.8	16780	Moderate coverage
MUS	National Statistical Office	2004	14.3	16780	Higher coverage
MUS	National Statistical Office	2005	15.8	16780	Higher coverage
MUS	National Statistical Office	2006	14.3	17610	Higher coverage
MUS	National Statistical Office	2007	15.7	17040	Higher coverage
MUS	National Statistical Office	2008	14.9	16380	Higher coverage
MUS	National Statistical Office	2009	16.4	15350	Higher coverage
MUS	National Statistical Office	2010	17.1	15010	Higher coverage
MUS	National Statistical Office	2011	17.0	14710	Higher coverage
MUS	National Statistical Office	2012	17.0	14500	Higher coverage
MUS	National Statistical Office	2013	16.7	13690	Higher coverage
MUS	National Statistical Office	2014	16.9	13420	Higher coverage
MUS	National Statistical Office	2015	17.3	12740	Higher coverage
MYS	Consultation	2001	9.9	515990	Higher coverage
MYS	Consultation	2002	9.9	484040	Higher coverage
MYS	Consultation	2003	9.9	480090	Higher coverage
MYS	Consultation	2004	10.1	477770	Higher coverage
MYS	Consultation	2005	10.1	469210	Higher coverage
MYS	Consultation	2006	10.4	465120	Higher coverage
MYS	Consultation	2007	10.7	472050	Higher coverage
MYS	Consultation	2008	10.7	487350	Higher coverage
MYS	Consultation	2009	10.9	496320	Higher coverage

ISO3 code	Data source	Year of data collection	Reported low birthweight rate	Number of weighed livebirths	National representativeness
MYS	Consultation	2010	11.3	491240	Higher coverage
MYS	Consultation	2011	11.2	511600	Higher coverage
MYS	Consultation	2012	11.3	526020	Higher coverage
MYS	Consultation	2013	11.2	503920	Higher coverage
MYS	Consultation	2015	11.4	521140	Higher coverage
NLD	Perined	2001	6.9	188630	Higher coverage
NLD	Perined	2002	6.6	188140	Higher coverage
NLD	Perined	2003	6.5	190060	Higher coverage
NLD	Perined	2004	6.4	181010	Higher coverage
NLD	Perined	2005	6.4	176330	Higher coverage
NLD	Perined	2006	6.3	175000	Higher coverage
NLD	Perined	2007	6.2	172290	Higher coverage
NLD	Perined	2008	6.2	176570	Higher coverage
NLD	Perined	2009	6.1	179530	Higher coverage
NLD	Perined	2010	6.2	178270	Higher coverage
NLD	Perined	2011	6.0	177620	Higher coverage
NLD	Perined	2012	6.1	175190	Higher coverage
NLD	Perined	2013	6.3	169000	Higher coverage
NLD	Perined	2014	6.3	174400	Higher coverage
NLD	Perined	2015	6.1	168460	Higher coverage
NOR	National Statistical Office	2000	4.9	59410	Higher coverage
NOR	National Statistical Office	2001	5.0	56940	Higher coverage
NOR	National Statistical Office	2002	5.1	56000	Higher coverage
NOR	National Statistical Office	2003	5.0	57150	Higher coverage
NOR	National Statistical Office	2004	4.9	57530	Higher coverage
NOR	National Statistical Office	2005	4.9	57250	Higher coverage
NOR	National Statistical Office	2006	4.9	59000	Higher coverage
NOR	National Statistical Office	2007	5.1	58960	Higher coverage
NOR	National Statistical Office	2008	5.0	61190	Higher coverage
NOR	National Statistical Office	2009	4.9	62760	Higher coverage
NOR	National Statistical Office	2010	4.9	62350	Higher coverage
NOR	National Statistical Office	2011	4.6	61100	Higher coverage
NOR	National Statistical Office	2012	4.7	61150	Higher coverage
NOR	National Statistical Office	2013	4.6	59760	Higher coverage
NOR	National Statistical Office	2014	4.6	59760	Higher coverage
NOR	National Statistical Office	2015	4.5	59710	Higher coverage
NZL	National Statistical Office	2000	6.3	57000	Higher coverage
NZL	National Statistical Office	2001	6.5	56230	Higher coverage
NZL	National Statistical Office	2002	6.5	54520	Higher coverage
NZL	National Statistical Office	2003	6.1	56580	Higher coverage
NZL	National Statistical Office	2004	6.1	58730	Higher coverage

ISO3 code	Data source	Year of data collection	Reported low birthweight rate	Number of weighed livebirths	National representativeness
NZL	National Statistical Office	2005	6.0	58730	Higher coverage
NZL	National Statistical Office	2006	5.8	60280	Higher coverage
NZL	National Statistical Office	2007	5.9	65130	Higher coverage
NZL	National Statistical Office	2008	5.9	65340	Higher coverage
NZL	National Statistical Office	2009	5.9	63290	Higher coverage
NZL	National Statistical Office	2010	5.8	64700	Higher coverage
NZL	National Statistical Office	2011	5.9	62180	Higher coverage
NZL	National Statistical Office	2012	6.1	62040	Higher coverage
NZL	National Statistical Office	2013	5.9	59710	Higher coverage
NZL	National Statistical Office	2014	5.6	59510	Higher coverage
NZL	National Statistical Office	2015	5.7	59310	Higher coverage
OMN	Ministry of Health	2000	8.1	58000	Higher coverage
OMN	Ministry of Health	2001	7.9	58000	Higher coverage
OMN	Ministry of Health	2002	7.9	58000	Higher coverage
OMN	Ministry of Health	2003	8.5	58000	Higher coverage
OMN	Ministry of Health	2004	8.2	58000	Higher coverage
OMN	Ministry of Health	2005	8.3	58000	Higher coverage
OMN	Ministry of Health	2006	8.9	58000	Higher coverage
OMN	Ministry of Health	2007	9.2	58000	Higher coverage
OMN	Ministry of Health	2008	9.2	58000	Higher coverage
OMN	National Statistical Office	2010	10.1	58000	Moderate coverage
OMN	National Statistical Office	2012	9.5	61730	Moderate coverage
OMN	National Statistical Office	2013	10.2	67240	Moderate coverage
OMN	National Statistical Office	2014	10.6	67240	Moderate coverage
OMN	National Statistical Office	2015	10.6	70620	Moderate coverage
PAN	National Statistical Office	2000	9.2	64840	Higher coverage
PAN	National Statistical Office	2001	9.3	63900	Moderate coverage
PAN	National Statistical Office	2002	9.6	61680	Moderate coverage
PAN	National Statistical Office	2003	10.3	61760	Moderate coverage
PAN	National Statistical Office	2004	9.8	62750	Moderate coverage
PAN	National Statistical Office	2006	9.3	59860	Moderate coverage
PAN	National Statistical Office	2007	8.5	61650	Moderate coverage
PAN	National Statistical Office	2008	9.1	63140	Moderate coverage
PAN	National Statistical Office	2009	9.1	68370	Moderate coverage
PAN	National Statistical Office	2010	8.8	63020	Moderate coverage
PAN	National Statistical Office	2011	8.3	68550	Moderate coverage
PAN	National Statistical Office	2012	8.0	71160	Higher coverage
PAN	National Statistical Office	2013	7.7	68510	Moderate coverage
PAN	National Statistical Office	2014	7.8	70580	Moderate coverage
PAN	National Statistical Office	2015	7.8	72100	Higher coverage
POL	National Statistical Office	2000	5.7	378350	Higher coverage

ISO3 code	Data source	Year of data collection	Reported low birthweight rate	Number of weighed livebirths	National representativeness
POL	National Statistical Office	2001	5.8	368210	Higher coverage
POL	National Statistical Office	2002	5.9	353770	Higher coverage
POL	National Statistical Office	2003	5.9	351080	Higher coverage
POL	National Statistical Office	2004	6.1	356140	Higher coverage
POL	National Statistical Office	2005	6.0	364390	Higher coverage
POL	National Statistical Office	2006	6.0	374250	Higher coverage
POL	National Statistical Office	2007	6.0	387880	Higher coverage
POL	National Statistical Office	2008	5.6	414500	Higher coverage
POL	National Statistical Office	2009	5.7	417590	Higher coverage
POL	National Statistical Office	2010	5.6	413300	Higher coverage
POL	National Statistical Office	2011	5.6	388420	Higher coverage
POL	Central Statistical Office	2012	5.7	386260	Higher coverage
POL	Central Statistical Office	2013	6.0	369580	Higher coverage
POL	Central Statistical Office	2014	5.9	375160	Higher coverage
POL	Central Statistical Office	2015	5.8	369310	Higher coverage
PRT	National Statistical Office	2000	7.1	120010	Higher coverage
PRT	National Statistical Office	2001	7.9	112780	Higher coverage
PRT	National Statistical Office	2002	7.4	114390	Higher coverage
PRT	National Statistical Office	2003	7.4	112520	Higher coverage
PRT	National Statistical Office	2004	7.6	109300	Higher coverage
PRT	National Statistical Office	2005	7.5	109400	Higher coverage
PRT	National Statistical Office	2006	7.6	105450	Higher coverage
PRT	National Statistical Office	2007	7.8	102500	Higher coverage
PRT	National Statistical Office	2008	7.7	104600	Higher coverage
PRT	National Statistical Office	2009	8.2	99500	Higher coverage
PRT	National Statistical Office	2010	8.3	101390	Higher coverage
PRT	National Statistical Office	2011	8.4	96860	Higher coverage
PRT	National Statistical Office	2012	8.5	89850	Higher coverage
PRT	National Statistical Office	2013	8.7	82790	Higher coverage
PRT	National Statistical Office	2014	8.7	82620	Higher coverage
PRT	National Statistical Office	2015	8.9	85780	Higher coverage
PRY	National Statistical Office	2014	6.2	112650	Moderate coverage
PRY	National Statistical Office	2015	6.0	116190	Moderate coverage
PSE	Palestinian Central Bureau of Statistics	2003	5.3	101650	Moderate coverage
PSE	Palestinian Central Bureau of Statistics	2009	8.4	115890	Moderate coverage
PSE	Palestinian Central Bureau of Statistics	2010	6.4	125590	Higher coverage
PSE	Palestinian Central Bureau of Statistics	2011	6.4	121500	Moderate coverage

ISO3 code	Data source	Year of data collection	Reported low birthweight rate	Number of weighed livebirths	National representativeness
PSE	Palestinian Central Bureau of Statistics	2012	7.6	118020	Moderate coverage
PSE	Palestinian Central Bureau of Statistics	2013	9.4	116210	Moderate coverage
PSE	National Statistical Office	2014	6.0	121330	Moderate coverage
PSE	National Statistical Office	2015	5.7	127270	Moderate coverage
QAT	National Statistical Office	2000	8.8	11250	Higher coverage
QAT	National Statistical Office	2001	9.5	12120	Higher coverage
QAT	National Statistical Office	2002	9.7	12200	Higher coverage
QAT	National Statistical Office	2003	7.1	12860	Higher coverage
QAT	National Statistical Office	2004	8.4	13190	Higher coverage
QAT	National Statistical Office	2005	8.5	13410	Higher coverage
QAT	National Statistical Office	2006	8.8	14120	Higher coverage
QAT	National Statistical Office	2007	8.3	15690	Higher coverage
QAT	National Statistical Office	2008	8.1	17210	Higher coverage
QAT	National Statistical Office	2009	7.2	18360	Higher coverage
QAT	National Statistical Office	2010	7.6	19510	Higher coverage
QAT	National Statistical Office	2011	7.9	20630	Higher coverage
ROU	National Statistical Office	2000	8.9	234530	Higher coverage
ROU	National Statistical Office	2001	8.9	220370	Higher coverage
ROU	National Statistical Office	2002	8.8	210530	Higher coverage
ROU	National Statistical Office	2003	9.0	212460	Higher coverage
ROU	National Statistical Office	2004	9.5	216270	Higher coverage
ROU	National Statistical Office	2005	9.5	221020	Higher coverage
ROU	National Statistical Office	2006	8.4	219490	Higher coverage
ROU	National Statistical Office	2007	8.1	214730	Higher coverage
ROU	National Statistical Office	2008	8.1	221900	Higher coverage
ROU	National Statistical Office	2009	7.9	222390	Higher coverage
ROU	National Statistical Office	2010	7.8	212200	Higher coverage
ROU	National Statistical Office	2011	8.0	196250	Higher coverage
ROU	National Statistical Office	2012	8.4	180720	Moderate coverage
ROU	National Statistical Office	2013	8.4	188600	Higher coverage
ROU	National Statistical Office	2014	8.3	198740	Higher coverage
ROU	National Statistical Office	2015	8.1	197500	Higher coverage
RUS	National Statistical Office	2000	7.7	1250860	Higher coverage
RUS	National Statistical Office	2001	6.4	1300550	Higher coverage
RUS	National Statistical Office	2002	6.2	1382980	Higher coverage
RUS	National Statistical Office	2003	6.1	1441920	Higher coverage
RUS	National Statistical Office	2004	6.1	1466440	Higher coverage
RUS	National Statistical Office	2005	6.0	1418970	Higher coverage
RUS	National Statistical Office	2006	5.9	1461100	Higher coverage
RUS	National Statistical Office	2007	6.0	1584730	Higher coverage

ISO3 code	Data source	Year of data collection	Reported low birthweight rate	Number of weighed livebirths	National representativeness
RUS	National Statistical Office	2008	5.8	1687400	Higher coverage
RUS	National Statistical Office	2009	5.7	1736890	Higher coverage
RUS	National Statistical Office	2010	5.8	1762790	Higher coverage
RUS	National Statistical Office	2011	5.9	1767610	Higher coverage
RUS	National Statistical Office	2012	6.1	1871240	Higher coverage
RUS	National Statistical Office	2013	6.2	1866450	Higher coverage
RUS	National Statistical Office	2014	5.9	1942690	Higher coverage
SGP	Registry of Births and Deaths	2000	8.1	44770	Higher coverage
SGP	Registry of Births and Deaths	2001	8.5	39290	Moderate coverage
SGP	Registry of Births and Deaths	2002	8.4	38560	Moderate coverage
SLV	National Statistical Office	2009	8.6	99570	Moderate coverage
SLV	National Statistical Office	2010	8.7	98360	Moderate coverage
SLV	National Statistical Office	2011	8.7	103840	Moderate coverage
SLV	National Statistical Office	2012	8.3	105520	Moderate coverage
SLV	National Statistical Office	2013	9.3	104630	Moderate coverage
SLV	National Statistical Office	2014	8.9	105770	Moderate coverage
SLV	National Statistical Office	2015	9.4	105240	Moderate coverage
SMR	National Statistical Office	2002	2.2	300	Higher coverage
SMR	National Statistical Office	2003	2.8	300	Higher coverage
SMR	National Statistical Office	2004	2.5	310	Higher coverage
SMR	National Statistical Office	2005	3.1	290	Moderate coverage
SMR	National Statistical Office	2006	3.6	310	Higher coverage
SMR	National Statistical Office	2008	3.8	350	Higher coverage
SMR	National Statistical Office	2009	3.5	310	Higher coverage
SMR	National Statistical Office	2010	4.0	340	Higher coverage
SMR	National Statistical Office	2011	3.3	330	Higher coverage
SMR	National Statistical Office	2012	2.6	300	Higher coverage
SMR	National Statistical Office	2013	3.1	320	Higher coverage
SMR	National Statistical Office	2014	4.5	300	Higher coverage
SMR	National Statistical Office	2015	3.8	270	Higher coverage
STP	National Statistical Office	2012	5.0	5430	Moderate coverage
STP	National Statistical Office	2013	5.3	5320	Moderate coverage
STP	National Statistical Office	2014	4.4	5600	Moderate coverage
STP	National Statistical Office	2015	4.6	5550	Moderate coverage
SUR	National Statistical Office	2007	13.5	8400	Moderate coverage
SUR	National Statistical Office	2008	12.4	8810	Moderate coverage
SUR	National Statistical Office	2011	13.1	8480	Moderate coverage
SUR	National Statistical Office	2012	14.6	8590	Moderate coverage
SUR	National Statistical Office	2013	13.9	8720	Moderate coverage
SUR	National Statistical Office	2014	13.3	9150	Moderate coverage
SVK	National Statistical Office	2000	6.7	55160	Higher coverage

ISO3 code	Data source	Year of data collection	Reported low birthweight rate	Number of weighed livebirths	National representativeness
SVK	National Statistical Office	2001	7.0	51140	Higher coverage
SVK	National Statistical Office	2002	6.9	50850	Higher coverage
SVK	National Statistical Office	2003	7.0	51720	Higher coverage
SVK	National Statistical Office	2004	7.2	53750	Higher coverage
SVK	National Statistical Office	2005	7.3	54430	Higher coverage
SVK	National Statistical Office	2006	7.3	53910	Higher coverage
SVK	National Statistical Office	2007	7.5	54430	Higher coverage
SVK	National Statistical Office	2008	7.3	57360	Higher coverage
SVK	National Statistical Office	2009	7.4	61220	Higher coverage
SVK	National Statistical Office	2010	9.0	60410	Higher coverage
SVK	National Statistical Office	2011	8.1	60820	Higher coverage
SVK	National Statistical Office	2012	7.9	55540	Higher coverage
SVK	National Statistical Office	2013	7.6	54830	Higher coverage
SVK	National Statistical Office	2014	7.8	55040	Higher coverage
SVK	National Statistical Office	2015	7.7	55610	Higher coverage
SVN	National Statistical Office	2000	5.6	18120	Higher coverage
SVN	National Statistical Office	2001	5.7	17420	Higher coverage
SVN	National Statistical Office	2002	6.0	17490	Higher coverage
SVN	National Statistical Office	2003	5.9	17070	Higher coverage
SVN	National Statistical Office	2004	5.8	17810	Higher coverage
SVN	National Statistical Office	2005	6.0	18090	Higher coverage
SVN	National Statistical Office	2006	5.8	18950	Higher coverage
SVN	National Statistical Office	2007	6.3	19820	Higher coverage
SVN	National Statistical Office	2008	6.3	21760	Higher coverage
SVN	National Statistical Office	2009	5.9	21650	Higher coverage
SVN	National Statistical Office	2010	6.2	22200	Higher coverage
SVN	National Statistical Office	2011	6.2	21740	Higher coverage
SVN	National Statistical Office	2012	6.0	21700	Higher coverage
SVN	National Statistical Office	2013	6.2	21120	Higher coverage
SWE	Medical Birth registry	2000	4.4	89380	Higher coverage
SWE	Medical Birth registry	2001	4.3	90190	Higher coverage
SWE	Medical Birth registry	2002	4.3	94750	Higher coverage
SWE	Medical Birth registry	2003	4.3	98140	Higher coverage
SWE	Medical Birth registry	2004	4.2	100670	Higher coverage
SWE	Medical Birth registry	2005	4.1	100460	Higher coverage
SWE	Medical Birth registry	2006	4.3	104300	Higher coverage
SWE	Medical Birth registry	2007	4.2	105650	Higher coverage
SWE	Medical Birth registry	2008	4.4	107960	Higher coverage
SWE	Medical Birth registry	2009	4.2	109330	Higher coverage
SWE	Medical Birth registry	2010	4.2	114730	Higher coverage
SWE	Medical Birth registry	2011	4.2	110900	Higher coverage

ISO3 code	Data source	Year of data collection	Reported low birthweight rate	Number of weighed livebirths	National representativeness
SWE	Medical Birth registry	2012	4.0	111850	Higher coverage
SWE	Medical Birth registry	2013	3.0	109360	Higher coverage
SWE	Medical Birth registry	2014	3.1	111780	Higher coverage
SYC	National Statistical Office	2000	8.7	1530	Higher coverage
SYC	National Statistical Office	2001	10.3	1450	Higher coverage
SYC	National Statistical Office	2002	9.2	1500	Higher coverage
SYC	National Statistical Office	2003	10.4	1500	Higher coverage
SYC	National Statistical Office	2004	8.9	1460	Moderate coverage
SYC	National Statistical Office	2005	10.1	1550	Higher coverage
SYC	National Statistical Office	2006	12.0	1480	Moderate coverage
SYC	National Statistical Office	2007	9.9	1530	Moderate coverage
SYC	National Statistical Office	2008	11.5	1550	Higher coverage
SYC	National Statistical Office	2009	10.9	1590	Higher coverage
SYC	National Statistical Office	2010	10.8	1500	Moderate coverage
SYC	National Statistical Office	2011	8.9	1660	Higher coverage
SYC	National Statistical Office	2012	12.4	1670	Higher coverage
SYC	National Statistical Office	2013	11.1	1680	Higher coverage
THA	National Statistical Office	2001	12.9	790430	Moderate coverage
THA	National Statistical Office	2002	12.5	782920	Moderate coverage
THA	National Statistical Office	2003	12.1	742190	Moderate coverage
THA	National Statistical Office	2004	11.3	813070	Higher coverage
THA	National Statistical Office	2005	11.1	809490	Higher coverage
THA	National Statistical Office	2006	11.4	793630	Higher coverage
THA	National Statistical Office	2007	11.0	797590	Higher coverage
THA	National Statistical Office	2008	10.8	784260	Higher coverage
THA	National Statistical Office	2009	11.4	765050	Higher coverage
THA	National Statistical Office	2010	11.3	761690	Higher coverage
THA	Ministry of Interior & MICS4	2011	10.4	NA	Higher coverage
THA	Health Information Unit, Strategy and Planning Division	2012	10.2	780980	Higher coverage
THA	Health Information Unit, Strategy and Planning Division	2013	10.9	748090	Higher coverage
THA	Health Information Unit, Strategy and Planning Division	2014	10.6	711810	Higher coverage
THA	Health Information Unit, Strategy and Planning Division	2015	10.6	679510	Higher coverage
TJK	National Statistical Office	2000	3.9	167250	Moderate coverage
TJK	National Statistical Office	2001	3.5	171630	Higher coverage
TJK	National Statistical Office	2002	3.6	175600	Higher coverage
TJK	National Statistical Office	2003	3.1	177940	Higher coverage
TJK	National Statistical Office	2004	3.3	179570	Higher coverage
TJK	National Statistical Office	2005	3.7	180790	Higher coverage

ISO3 code	Data source	Year of data collection	Reported low birthweight rate	Number of weighed livebirths	National representativeness
TJK	National Statistical Office	2006	3.6	186470	Higher coverage
TJK	National Statistical Office	2007	3.8	200010	Higher coverage
TJK	National Statistical Office	2008	4.3	203340	Higher coverage
TJK	National Statistical Office	2009	4.9	199830	Moderate coverage
TJK	National Statistical Office	2010	4.8	239810	Higher coverage
TJK	National Statistical Office	2011	5.1	224180	Higher coverage
TJK	National Statistical Office	2012	5.6	219290	Higher coverage
TJK	National Statistical Office	2013	5.9	209420	Moderate coverage
TJK	National Statistical Office	2014	7.2	229460	Higher coverage
TKM	National Statistical Office	2002	3.2	NA	Moderate coverage
TKM	National Statistical Office	2003	3.9	NA	Moderate coverage
TKM	National Statistical Office	2004	3.5	NA	Moderate coverage
TKM	National Statistical Office	2005	3.7	NA	Moderate coverage
TKM	National Statistical Office	2006	3.8	NA	Moderate coverage
TKM	National Statistical Office	2007	5.0	NA	Moderate coverage
TKM	National Statistical Office	2008	5.0	NA	Moderate coverage
TKM	National Statistical Office	2009	4.9	NA	Moderate coverage
TKM	National Statistical Office	2010	4.6	NA	Moderate coverage
TKM	Ministry of Health and Medical Industry of Turkmenistan	2011	4.6	NA	Moderate coverage
TKM	Ministry of Health and Medical Industry of Turkmenistan	2012	4.9	NA	Moderate coverage
TKM	Ministry of Health and Medical Industry of Turkmenistan	2013	4.9	NA	Moderate coverage
TKM	Ministry of Health and Medical Industry of Turkmenistan	2014	5.0	NA	Moderate coverage
TKM	Ministry of Health and Medical Industry of Turkmenistan	2015	4.8	NA	Moderate coverage
TTO	National Statistical Office	2006	8.6	16010	Moderate coverage
TTO	National Statistical Office	2008	10.2	16830	Moderate coverage
TTO	National Statistical Office	2009	10.3	16500	Moderate coverage
TTO	National Statistical Office	2013	11.6	15940	Moderate coverage
TUR	Ministry of Health	2012	8.8	1265550	Higher coverage
TUR	National Statistical Office	2013	8.4	1271250	Higher coverage
TUR	Ministry of Health	2014	8.6	1318690	Higher coverage
TUR	Ministry of Health	2015	8.6	1301540	Higher coverage
UKR	National Statistical Office	2000	5.4	NA	Higher coverage
UKR	National Statistical Office	2001	5.3	NA	Higher coverage
UKR	National Statistical Office	2002	5.2	NA	Higher coverage
UKR	National Statistical Office	2003	5.4	NA	Higher coverage
UKR	National Statistical Office	2004	5.1	NA	Higher coverage
UKR	National Statistical Office	2005	5.1	NA	Higher coverage

ISO3 code	Data source	Year of data collection	Reported low birthweight rate	Number of weighed livebirths	National representativeness
UKR	National Statistical Office	2006	5.1	NA	Higher coverage
UKR	National Statistical Office	2007	5.1	NA	Higher coverage
UKR	National Statistical Office	2008	5.1	NA	Higher coverage
UKR	National Statistical Office	2009	5.2	NA	Higher coverage
UKR	National Statistical Office	2010	5.3	NA	Higher coverage
UKR	National Statistical Office	2011	5.3	NA	Higher coverage
UKR	National Statistical Office	2012	5.3	NA	Higher coverage
UKR	National Statistical Office	2013	5.4	NA	Higher coverage
URY	National Statistical Office	2000	7.6	NA	Higher coverage
URY	National Statistical Office	2001	8.2	NA	Higher coverage
URY	National Statistical Office	2002	8.1	NA	Higher coverage
URY	National Statistical Office	2003	9.1	NA	Higher coverage
URY	National Statistical Office	2004	8.7	NA	Higher coverage
URY	National Statistical Office	2005	8.7	NA	Higher coverage
URY	National Statistical Office	2006	8.5	NA	Higher coverage
URY	National Statistical Office	2007	8.4	NA	Higher coverage
URY	National Statistical Office	2008	8.4	NA	Higher coverage
URY	National Statistical Office	2009	8.4	NA	Higher coverage
URY	National Statistical Office	2010	8.3	NA	Higher coverage
URY	National Statistical Office	2011	8.3	NA	Higher coverage
URY	National Statistical Office	2012	8.1	NA	Higher coverage
URY	National Statistical Office	2013	7.6	NA	Higher coverage
URY	National Statistical Office	2014	7.5	NA	Higher coverage
URY	National Statistical Office	2015	7.7	NA	Higher coverage
USA	National Statistical Office	2000	7.6	NA	Higher coverage
USA	National Statistical Office	2001	7.7	NA	Higher coverage
USA	National Statistical Office	2002	7.8	NA	Higher coverage
USA	National Statistical Office	2003	7.9	NA	Higher coverage
USA	National Statistical Office	2004	8.1	NA	Higher coverage
USA	National Statistical Office	2005	8.2	NA	Higher coverage
USA	National Statistical Office	2006	8.3	NA	Higher coverage
USA	National Statistical Office	2007	8.2	NA	Higher coverage
USA	National Statistical Office	2008	8.2	NA	Higher coverage
USA	National Statistical Office	2009	8.2	NA	Higher coverage
USA	National Statistical Office	2010	8.1	NA	Higher coverage
USA	National Statistical Office	2011	8.1	NA	Higher coverage
USA	National Statistical Office	2012	8.0	NA	Higher coverage
USA	National Statistical Office	2013	8.0	NA	Higher coverage
USA	National Statistical Office	2014	8.0	NA	Higher coverage
USA	National Statistical Office	2015	8.1	NA	Higher coverage
UZB	National Statistical Office	2000	4.7	NA	Moderate coverage

ISO3 code	Data source	Year of data collection	Reported low birthweight rate	Number of weighed livebirths	National representativeness
UZB	National Statistical Office	2001	4.6	NA	Moderate coverage
UZB	National Statistical Office	2002	4.6	NA	Moderate coverage
UZB	National Statistical Office	2003	4.8	NA	Moderate coverage
UZB	National Statistical Office	2004	4.8	NA	Moderate coverage
UZB	National Statistical Office	2005	4.5	NA	Moderate coverage
UZB	National Statistical Office	2006	4.3	NA	Moderate coverage
UZB	National Statistical Office	2007	4.7	NA	Moderate coverage
UZB	National Statistical Office	2008	4.4	NA	Moderate coverage
UZB	National Statistical Office	2009	4.7	NA	Moderate coverage
UZB	National Statistical Office	2010	4.9	NA	Moderate coverage
UZB	National Statistical Office	2011	5.1	NA	Moderate coverage
UZB	National Statistical Office	2012	4.6	NA	Moderate coverage
UZB	National Statistical Office	2013	4.3	NA	Moderate coverage
UZB	National Statistical Office	2014	3.9	NA	Moderate coverage
VEN	National Statistical Office	2000	8.8	NA	Higher coverage
VEN	National Statistical Office	2001	8.7	NA	Higher coverage
VEN	National Statistical Office	2002	8.5	NA	Higher coverage
VEN	National Statistical Office	2003	8.7	NA	Higher coverage
VEN	National Statistical Office	2004	8.8	NA	Higher coverage
VEN	National Statistical Office	2005	9.0	NA	Higher coverage
VEN	National Statistical Office	2006	9.0	NA	Higher coverage
VEN	National Statistical Office	2007	8.9	NA	Higher coverage
VEN	National Statistical Office	2008	8.2	NA	Higher coverage
VEN	National Statistical Office	2009	8.2	NA	Higher coverage
VEN	National Statistical Office	2010	8.4	NA	Higher coverage
VEN	National Statistical Office	2011	8.7	NA	Higher coverage
VEN	National Statistical Office	2012	8.5	NA	Higher coverage
VEN	National Statistical Office	2013	8.7	NA	Higher coverage
VEN	National Statistical Office	2014	9.0	NA	Higher coverage
VEN	National Statistical Office	2015	9.0	NA	Higher coverage
ZAF	HMIS	2005	10.5	940060	Moderate coverage
ZAF	HMIS	2006	11.8	940060	Moderate coverage
ZAF	HMIS	2007	11.7	940060	Moderate coverage
ZAF	HMIS	2008	11.8	940060	Moderate coverage
ZAF	HMIS	2009	12.3	940060	Moderate coverage
ZAF	HMIS	2010	12.7	940060	Moderate coverage
ZAF	HMIS	2011	13.2	940060	Moderate coverage
ZAF	HMIS	2012	13.5	940060	Moderate coverage
ZAF	HMIS	2014	12.6	958260	Moderate coverage

NA=Not available. For datapoints with missing data on number of babies weighed assumed: (1) For High Income Countries; assumed higher coverage (2) For countries with higher coverage time series with missing data for some years; assumed

higher coverage. (3) For countries with high facility birth from TRANSMONEE database (TKM, UZB); assumed moderate coverage

Figure 4.1: Summary of regional low birthweight rate data inputs meeting inclusion criteria

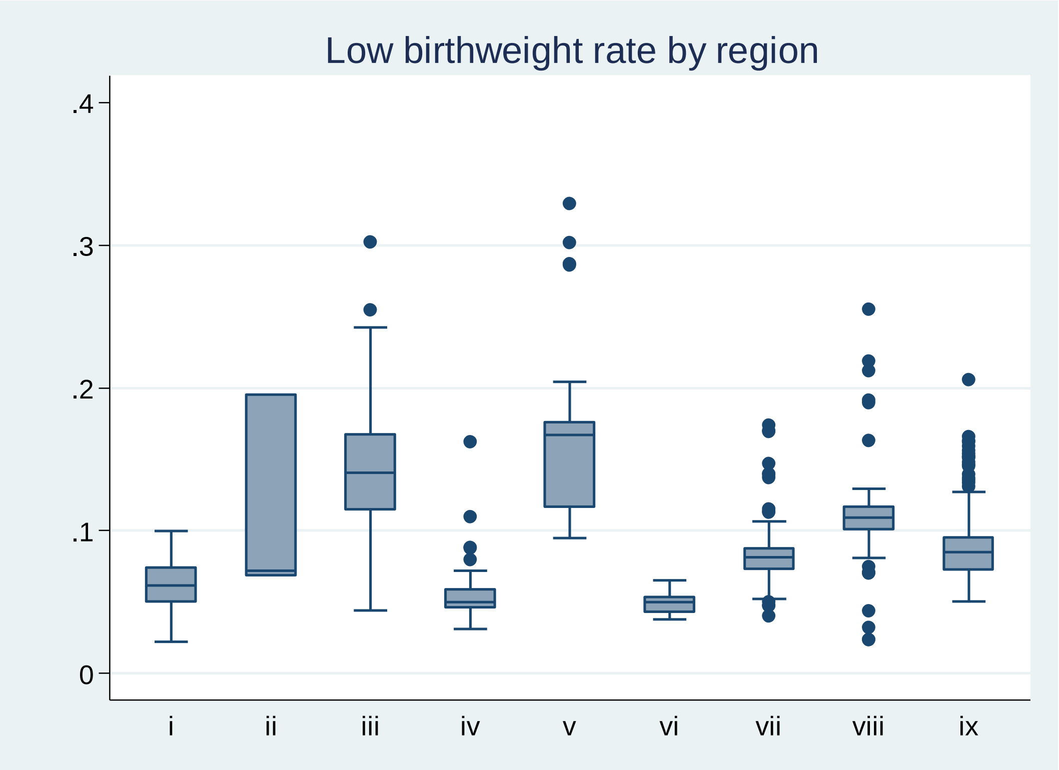

Region	Constituting regions	Number data points	% of input database
i	North America, Europe, Australia and New Zealand	696	48.1
ii	Northern Africa	3	0.2
iii	Southern/East/West Africa	158	10.9
iv	Central Asia	85	5.9
v	Southern Asia	30	2.1
vi	Eastern Asia	22	1.5
vii	Western Asia	143	9.9
viii	South Eastern Asia and Oceania[1]	72	5.0
ix	Latin America and Caribbean	238	16.5

[1]without Australia and New Zealand

Table 4.3: Summary of datapoints included by World Bank income region:

Income group	Number of countries	Number of countries with data	% of countries with no data	Mean number datapoints for countries with data	Mean number of datapoints per country overall	Number of livebirths in 2015	% of global livebirths in 2015
High income	56	51	8.9	14	13	12,665,700	9.0%
Upper middle income	55	43	21.8	11	8	37,067,400	26.4%
Lower middle income	51	35	31.4	6	4	67,634,500	48.1%
Low income	31	18	41.9	4	2	23,186,200	16.5%

* Cook Islands is not included in the above table as it is not classified in the World Bank income regions.

Quality categorisation of administrative data

To seek to categorise the quality of available national level routine data: first we reviewed previously used quality criteria from other maternal and perinatal global estimation exercises, and secondly further criteria suggested by the expert group that were plausibly associated with the strength of low birthweight reporting were added (see Table 4.3). All of the identified potential methods had limitations, either in the proximity of the indicator to birthweight reporting, or in the feasibility of collecting the data. Of the identified potential low birthweight administrative data quality criteria, only population representativeness was assessed to be currently feasible.

Table 4.3: Summary of previously used and proposed quality criteria for the assessment of National Routine Data in global estimation exercises.

Method	Components	Potential benefits	Potential limitations
Vital Statistics Performance Index (VSPI)[2]	VSPI uses mortality data as proxy for quality & utility of CRVS. Six components:<br>• Completeness of death reporting<br>• Quality of death reporting<br>• Level of cause-specific detail<br>• Internal consistency<br>• Quality of age & sex reporting<br>• Data availability or timeliness<br>High VSPI (70-100): 69 countries<br>Medium VSPI (50-69): 17 countries	Published and used classification, developed with robust methodology	Only relates to CRVS strength for death reporting, distal to birthweight on birth certificate<br><br>Could be used in conjunction with another method
Maternal death reporting quality criteria (2015 estimates)[3]	CRVS system operational for more than 20 years (earliest data from before 1996), and reporting data after 2007<br>Low rate of ill-defined causes of maternal mortality (<0.2)	Used in published literature for maternal death estimates<br>Outcome (maternal death) occurring around the time of birth, so may be more linked than all-age CRVS death reporting	Less robust methodology in its development<br>Death reporting, therefore distal to birthweight on birth certificate
Stillbirth estimates (2015)[4]	Composite measure of:<br>*CRVS strength:*<br>VR is considered 'good quality' for the purposes of maternal death reporting	Used in published literature for stillbirth estimates<br>Outcome (stillbirth) occurring around the time of birth,	Less robust methodology in its development<br>Death reporting, therefore distal to birthweight on birth certificate

	For given year female child mortality capture>85%<br>*CRVS capture of perinatal events:*<br>Stillbirth rate (adjusted to 28 week definition): national UN-IGME NMR ratio >0.5 for all years<br>*Completeness of data:*<br>Earliest year of data available is before 2005<br>Latest year of data available is after 2010.<br>Number of years of data >0.5 *(max year – min year + 1).	so may be more linked than all-age CRVS death reporting<br>Uses a composite measure that seeks to capture several dimensions of the quality	
Proposed potential quality criteria specific to low birthweight			
Availability of LBW sub-envelop data	Are data presented by LBW sub-group	Can be more critically reviewed, e.g. are they missing most of the extremely preterm babies (<1000g)	Data may be available for a country – but not included in the reporting statistics collected.
Coverage of birth certificate	Coverage of birth certificate	Proximal to birthweight collection (on certificate)<br>If no birth certificate, will not have been able to collect birthweight information from it	Data not available on birth certificate around time of birth. Many initiatives to increase birth certificate coverage use immunisations or catch up later in childhood – so high coverage, does not equate to good birthweight data
Population representativeness	Proportion of estimated total livebirths in the country in the given year covered by the reporting system	Proximal to outcome	Acceptable levels of population representativeness need defining
Facility birth rate	Proportion of all births in a country that are in facilities	Birthweight is less likely to be recorded for births outside a facility	Facility birth is not a guarantee of birthweight being measured or recorded, and the population representativeness above provides a closer measure of the outcome – however may be useful for

			excluding countries with low coverage of birthweight data
Completeness of birthweight reporting within the data system	% of all births captured in the system with a birthweight	Proximal to outcome	In general, very low levels of missing birthweight in data systems reporting this. % missing is absent from many data sources.

Table 4.4: Countries with higher quality national routine data (N=57)

Argentina	Cuba	Luxembourg	Slovenia
Armenia	Czechia	Malaysia	Spain
Australia	Denmark	Malta	Sri Lanka
Austria	Estonia	Mauritius	Sweden
Azerbaijan	Finland	Montenegro	Switzerland
Bahrain	Georgia	Netherlands	Thailand
Belarus	Germany	New Zealand	Macedonia
Belgium	Hungary	Norway	Ukraine
Brazil	Iceland	Poland	United Kingdom
Brunei Darussalam	Ireland	Portugal	USA
Bulgaria	Israel	Qatar	Uruguay
Canada	Japan	Republic of Korea	Venezuela
Chile	Kazakhstan	Russian Federation	
Costa Rica	Kyrgyzstan	Seychelles	
Croatia	Latvia	Slovakia	

Table 4.5: Summary of input data by data type

		Low birthweight rate			
**Data type**	Number of data inputs	Mean	Standard Deviation	Minimum	Maximum
**Overall**	1447	8.1	3.9	2.2	32.9
**High quality admin data**	1026	7.1	2.5	2.2	17.6
**Moderate quality admin data**	192	7.9	3.1	2.4	15.7
**Nationally representative survey**	229	12.9	5.6	3.1	32.9

5. Predictor variables

Identification of predictor variables for model input

A scoping literature review was conducted to identify covariates which were associated with LBW rates (not necessarily determinants of LBW) at a population level. These included distal determinants such as geographical and socio-economic factors, interacting and overlapping demographic and biomedical factors, associated perinatal outcome markers and access to health care. Out of these covariates the ones for which complete time-series data was available for the period we are predicting for (2000-2015) were included in the modelling process and are listed in Table 5.1 with the respective sources they were obtained from.

Table 5.1: Source of potential predictor variables tested in models

Predictor	Source
Gross National Income (GINI)	World Bank http://data.worldbank.org/indicator
GINI coefficient	World Bank http://data.worldbank.org/indicator
% of the population that are urban	UNPD World Urbanization Prospects 2014 https://esa.un.org/unpd/wup/publications/files/wup2014-highlights.pdf
Mean adult female education (years)	UNESCO Institute for Statistics and HDRO estimates based on data on education attainment from UNESCO Institute for Statistics (http://uis.unesco.org/) and on methodology from Barro-Lee[5]
Human Development Index (HDI)	UNDP Human Development Report 2014 http://hdr.undp.org/sites/default/files/hdr14-report-en-1.pdf
Stunting in children under 5 years	Prevalence HAZ <-2 from Stevens et al[6]
Underweight in children under 5 years	Prevalence WAZ <-2 from Stevens et al[6]
Neonatal Mortality Rate (NMR)	UN-IGME 2015 available from http://www.childmortality.org/
Adult Female Underweight (age-standardised)[1]	NCD Risk Factor Collaboration (NCD-RisC) http://ncdrisc.org/
Adult Female Overweight (age-standardised)[1]	NCD Risk Factor Collaboration (NCD-RisC) http://ncdrisc.org/
Adult Female Obesity (age-standardised)[1]	NCD Risk Factor Collaboration (NCD-RisC) http://ncdrisc.org/
General Fertility Rate (GFR)	UNPD
Plasmodium falciparum parasite rate	Malaria Atlas Project http://www.map.ox.ac.uk/ (Oxford Cube)
% pregnant women attending 4 antenatal care visits (ANC)	UNICEF database http://data.unicef.org/#
Adolescent fertility rate	World Bank http://data.worldbank.org/indicator
Modern contraceptive rate prevalence	United Nations Department of Economic and Social Affairs (UNDESA) https://www.un.org/development/desa/en/
Adult Female Smoking rate	Institute of Health Metrics and Evaluation (IHME) http://ghdx.healthdata.org/ihme_data
Geographical Region (based on UN region)	United Nations Statistics Division http://unstats.un.org/unsd/methods/m49/m49regin.htm

[1]Excluded as a covariate in the final model fitting process due to its non-linear association with low birthweight.

6. Model selection and estimation

All identified predictor variables with full time series were included in the initial model. For continuous variables, a univariate analysis was undertaken to identify whether the relationship between the log low birthweight (ln_lbw) and the predictor was best described using a ln transformation, or using the non-transformed predictor. For all variables the BIC was calculated. Predictors were retained when the direction of the coefficient was plausible. All covariates were retained at this stage as the direction of all the coefficients were plausible. Correlation between predictors was assessed using Variance Inflation Factor (VIF). Predictors with a VIF of >10 were dropped. Predictors were retained when the direction of the coefficient was biologically plausible.

We sought to maximise the predicting power of the model, whilst reducing the noise within the model from a large number of predictors. We removed one predictor at a time from the model, commencing with the predictor with the largest BIC on univariate analysis, and re-fitted the model. If the model was improved by removing this predictor (lower BIC compared to the model containing the predictor), the predictor was dropped from the model. If the BIC was higher, the predictor was retained. The p.falciparum parasite, female smoking, gini index, modern contraception, general fertility rate, GNI, % urban, Maternal education, antenatal care coverage, adolescent fertility rate and female underweight skilled birth attendant, were dropped as a result of this process. Once this had been completed the final step was to check if the direction of the model coefficient for all the remaining predictors was plausible. See table 5.1.

Table 6.1: Summary of model fitting process

	Effect of dropping predictor	Outcome	BIC
Full Model		-	-1491.9
Dropping malaria	Improved BIC	Dropped from model	-1498.5
Dropping smoking	Improved BIC	Dropped from model	-1500.1
Dropping gini	Improved BIC	Dropped from model	-1504.5
Dropping modern contraception	Improved BIC	Dropped from model	-1510.5
Dropping general fertility rate	Improved BIC	Dropped from model	-1515.7
Dropping gross national income	Improved BIC	Dropped from model	-1519.9
Dropping urban	Improved BIC	Dropped from model	-1525.9
Dropping maternal education	Improved BIC	Dropped from model	-1531.2
Dropping ANC	Improved BIC	Dropped from model	-1538.5
Dropping stunting	Worsened BIC	Retained in model	-1517.5
Dropping adolescent fertility rate	Improved BIC	Dropped from model	-1542.7
Dropping region	Worsened BIC	Retained in model	-1523.2
Dropping adult female underweight	Improved BIC	Dropped from model	-1547.8
Dropping underweight	Worsened BIC	Retained in model	-1529.9
Dropping NMR	Worsened BIC	Retained in model	-1519.5
Dropping context	Worsened BIC	Retained in model	-1448.4
**Checking direction of covariates**	Stunting non-plausible direction of coefficient	Stunting dropped from the model	
**Final model**			-1531.039

[1] *Context= data type variable – higher quality admin, lower quality admin, or nationally representative survey*

The final model included ln low birthweight,neonatal mortality rate, child underweight, region (sub-Saharan Africa, Southern Asia or other) and a dummy variable for data type

Final model equation

$Log(\text{Low birthweight rate}_{ij}) = a + b(NMR_{ij}) + c(\text{child underweight}_{ij}) + d(\text{region}_{ij}) + e(\text{datatype}_{ij}) + u_j + e_{ij}$

b() and c()represent functions each involving 2 parameters,

d() indicates a 3 parameter function associated with 3 dummy variables representing different aggregated regional grouping (south asia, sub-saharan Africa, other regions)

d() indicates a 3 parameter function associated with 3 dummy variables representing different data types (see paper for details)

u_j represent country-specific random effects, assumed to be independent normally distributed with constant variance

e_{ij} represent individual data point-level residuals, assumed to be independent normally distributed with constant variance

Figure 6.1: Diagnostic plots for the low birthweight prediction regression model

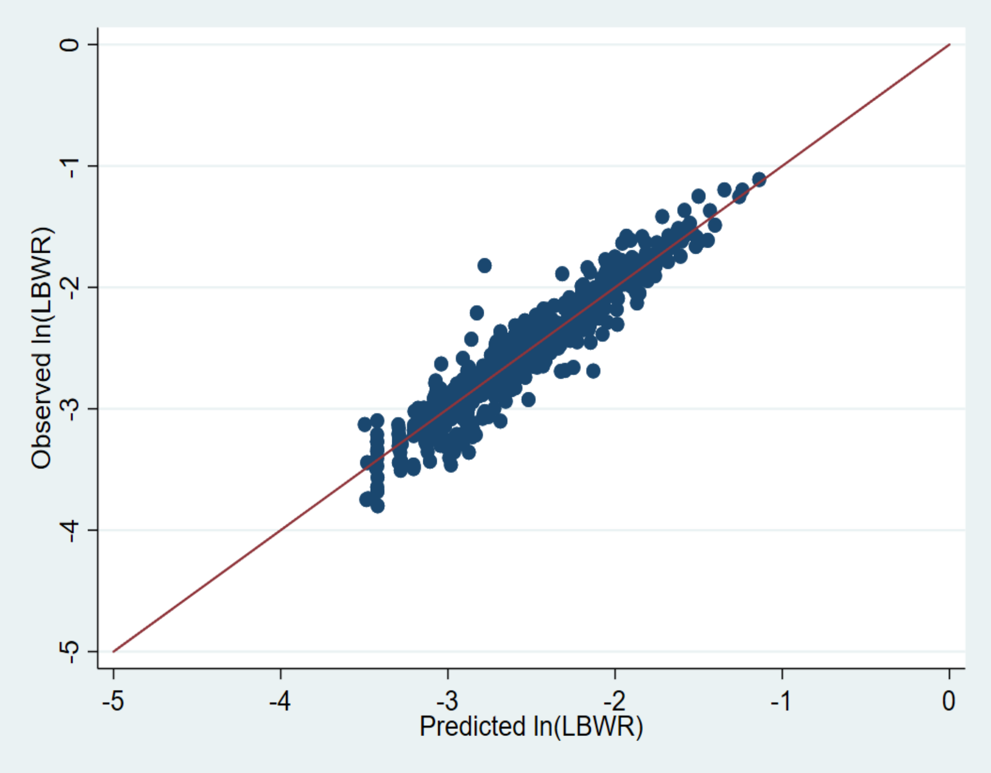

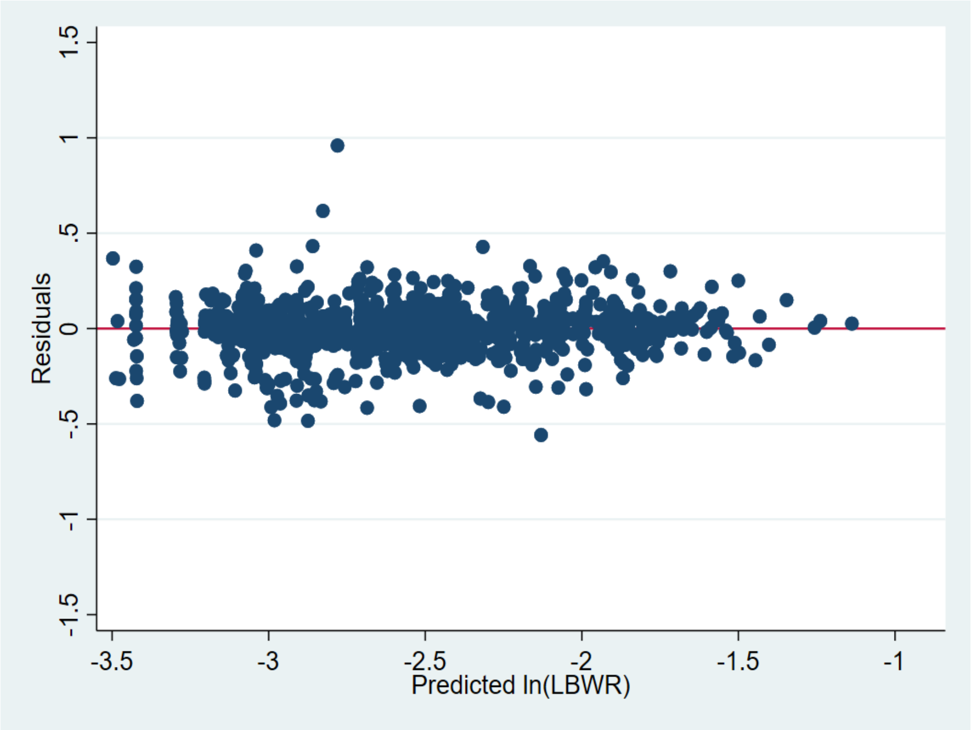

Residuals
Predicted ln(LBWR)

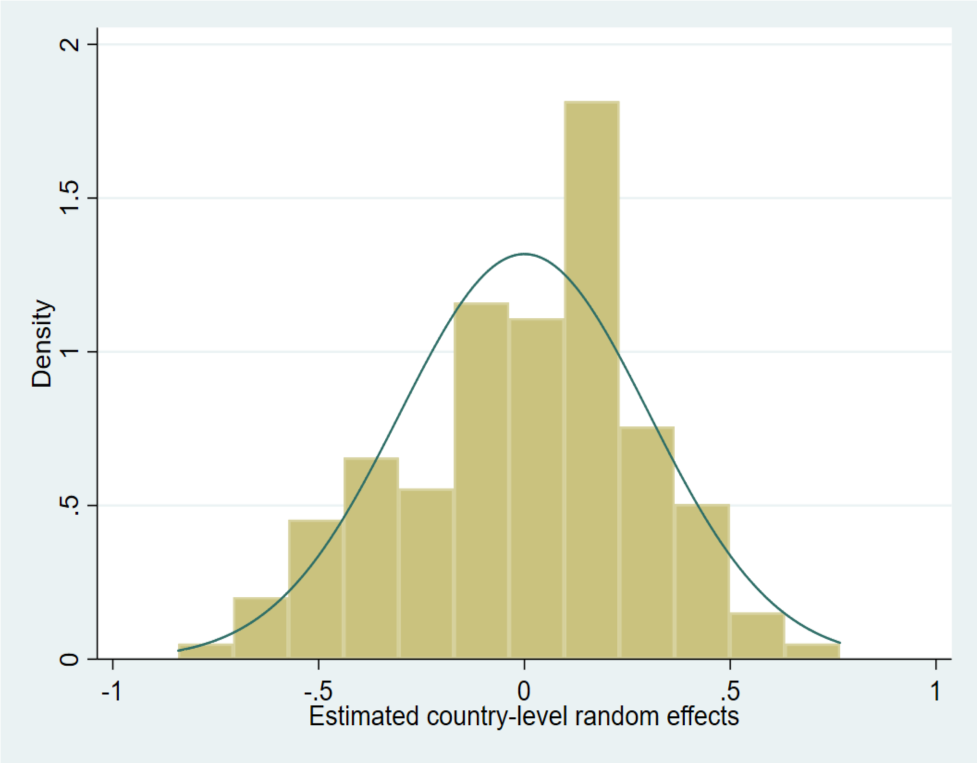

Density
Estimated country-level random effects

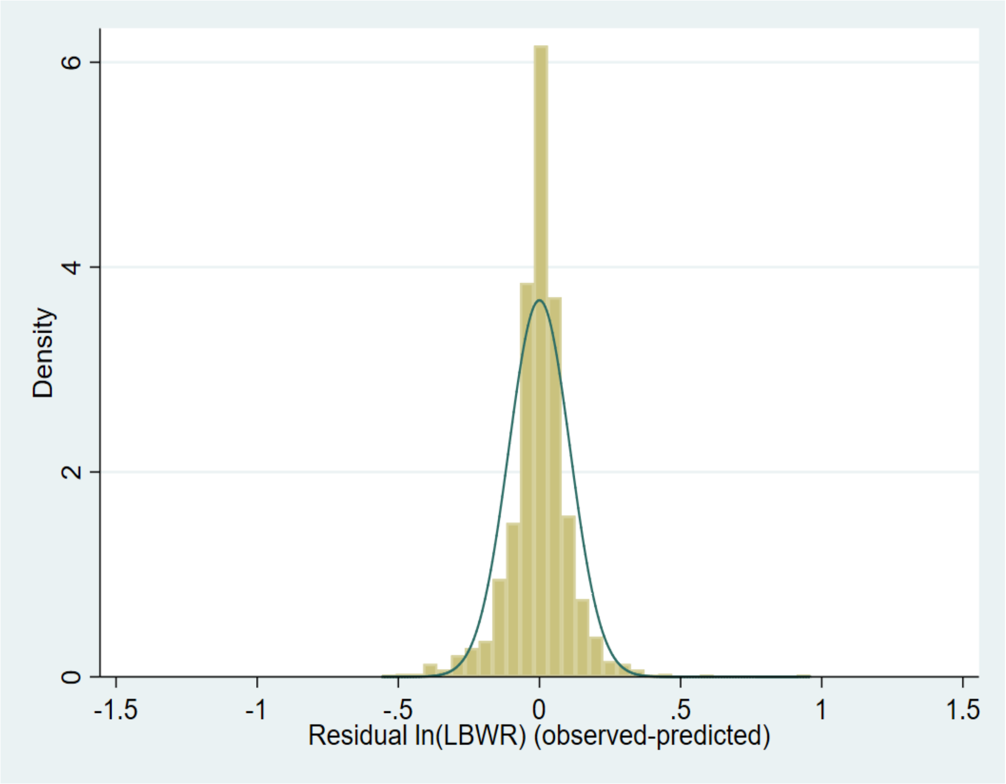

Table 6.2: Model coefficients

		Coefficient (95% Confidence Interval)
Neonatal Mortality Rate		0.009 (0.005 – 0.012)
Child underweight		0.615 (-0.031 – 1.26)
Region	Other regions	-
	Sub-Saharan Africa	0.3 (0.169 – 0.432)
	Southern Asia	0.635 (0.355 – 0.915)
Data type	High quality admin data	-
	Moderate quality admin data	-0.008 (-0.036 – 0.002)
	Nationally representative survey	0.165 (0.132 – 0.198)

Table 6.2 shows the estimated coefficients for the predictors retained in the final model. Each unit increase in NMR is associated with a 0.01-unit increase in the natural log LBW rate. Unit increases in prevalence of child underweight are associated with a 0.62-unit increase in natural log LBW rate. Little difference was observed between LBW rates from high and moderate quality administrative data sources. However, adjusted nationally representative household survey data LBW rates were systematically higher than administrative data sources. As expected, low birthweight rates for Southern Asia and sub-Saharan Africa were higher than those from other regions.

Calculation of low birthweight numbers

Live birth estimates from the World Population Prospects: the 2017 revision[7] were used to convert the low birthweight rate to the number of low birthweights using the formula:
Number of low birthweights = (low birthweight rate*number of livebirths)/100

Uncertainty estimation

For countries with higher quality administrative data we used a bspline regression model to predict the standard error and calculate 95% confidence intervals for the country-level LBW estimates. Using the calculated standard errors and assuming a normal distribution, we randomly sampled 1000 times from the distribution to generate 1000 point estimates for LBW for each country with higher quality administrative data for each year.

For all other countries, estimates of uncertainty were generated by drawing 1000 bootstrap samples from the estimation dataset and using the parameter estimates obtained from each bootstrap sample to generate a new set of predictions including the estimate of the variance of the country-specific random effects. We used this estimate of the between country variance to draw, at random, a random effect for countries not included in the bootstrap sample assuming that the random effects are normally distributed with mean zero and variance equal to that estimated from the model.

To obtain worldwide and regional level estimates of uncertainty, we summed the 1000 LBW point estimates for each country for each year from the two approaches detailed above at worldwide or regional level and used the 2.5th and 97.5th centiles of the resulting distributions. Because about half of the modelled countries had a country-specific random effect generated at random for each bootstrap prediction, some of which were positive and others negative, the relative uncertainty at the regional and worldwide level tends to be less than that at the individual country level.

Annual Average Rate of Reduction (AARR)

The Annual Average Rate of Reduction (AARR) is the average relative percent decrease per year in prevalence or rate and is given by

$$1 - \left(\frac{\mathrm{LBWr}_1}{\mathrm{LBWr}_0}\right)^{\frac{1}{t}}$$

where LBWr_1 is the end year LBW rate, LBWr_0 is the starting year LBW rate, and t is the number of years between start and end years of the relevant period.

7. Results

Table 7.1: Estimated low birthweight rate by country for 2000 and 2015 for 148 countries[2] with data

Country	Low birthweight rate 2000	Number of low birthweights 2000	Low birthweight rate 2015	Number of low birthweights 2015	2000-2015 ARR %	Source of estimate
Albania	4.9	2482	4.6	1594	0.4	hierarchical regression
Algeria	7.7	47080	7.3	68846	0.4	hierarchical regression
Andorra	7.5	52	7.4	57	0.1	hierarchical regression
Angola	18.0	146719	15.3	180176	1.1	hierarchical regression
Antigua and Barbuda	9.4	161	9.1	148	0.3	hierarchical regression
Argentina	7.4	53097	7.3	55369	0.0	b-spline regression
Armenia	8.2	3228	9.0	3606	-0.6	b-spline regression
Australia	6.3	15575	6.5	20256	-0.2	b-spline regression
Austria	6.4	5117	6.5	5419	-0.2	b-spline regression
Azerbaijan	6.5	9185	7.3	12819	-0.7	b-spline regression
Bahamas	13.4	707	13.1	732	0.1	hierarchical regression
Bahrain	8.5	1257	11.9	2559	-2.3	b-spline regression
Bangladesh	36.2	1318013	27.8	864807	1.7	hierarchical regression
Belarus	5.0	4468	5.1	5790	0.0	b-spline regression
Belgium	7.2	8196	7.3	9355	-0.1	b-spline regression
Belize	9.0	659	8.6	703	0.3	hierarchical regression
Benin	18.7	54612	16.9	66992	0.7	hierarchical regression
Bhutan	13.8	2215	11.7	1694	1.1	hierarchical regression
Bolivia (Plurinational State of)	8.0	20549	7.2	18256	0.7	hierarchical regression
Bosnia and Herzegovina	3.5	1454	3.4	1104	0.2	hierarchical regression
Botswana	16.3	7762	15.6	8337	0.3	hierarchical regression
Brazil	7.8	276914	8.4	248646	-0.5	b-spline regression
Brunei Darussalam	10.3	732	10.8	724	-0.3	b-spline regression
Bulgaria	8.6	5633	9.6	6397	-0.7	b-spline regression
Burkina Faso	15.8	85144	13.1	94015	1.2	hierarchical regression
Burundi	17.4	47609	15.1	66158	0.9	hierarchical regression
Cambodia	15.4	52387	12.1	44558	1.6	hierarchical regression
Cameroon	13.1	83123	12.0	100727	0.6	hierarchical regression
Canada	5.5	18358	6.4	24684	-1.0	b-spline regression
Central African Republic	15.4	23172	14.5	24124	0.4	hierarchical regression
Chile	5.2	13570	6.2	14910	-1.2	b-spline regression
China	5.8	940617	5.0	846892	1.0	hierarchical regression
Cote D'Ivoire	17.5	118883	15.5	132695	0.8	hierarchical regression
Colombia	10.5	89037	10.0	74309	0.4	hierarchical regression
Comoros	25.9	5271	23.7	6143	0.6	hierarchical regression
Congo	13.5	17109	11.6	20485	1.0	hierarchical regression
Cook Islands	3.7	15	3.5	12	0.5	hierarchical regression

[2] India is counted among these 148 countries but as it only had partial data for the most recent survey, estimates are not reported. These data have been used in regional and global estimates. (India was labelled as partial data as no HMIS/ admin data met the inclusion criteria; one survey (National Family Health Survey, 2005-2006) was included, however for the latest survey (National Family Health Survey, 2015-16) only data for a single year met inclusion criteria and these partial data were used).

Country	Low birthweight rate 2000	Number of low birthweights 2000	Low birthweight rate 2015	Number of low birthweights 2015	2000-2015 ARR %	Source of estimate
Costa Rica	7.0	5336	7.5	5222	-0.4	b-spline regression
Croatia	5.4	2454	5.1	2014	0.4	b-spline regression
Cuba	6.1	8862	5.3	6604	1.0	b-spline regression
Czechia	5.8	5146	7.8	8539	-2.0	b-spline regression
Democratic Republic of the Congo	12.4	269628	10.8	354423	0.9	hierarchical regression
Denmark	5.1	3384	5.3	3160	-0.2	b-spline regression
Dominican Republic	11.6	25121	11.3	24386	0.2	hierarchical regression
Ecuador	12.0	37786	11.2	36967	0.5	hierarchical regression
El Salvador	11.0	15983	10.3	12156	0.4	hierarchical regression
Estonia	4.3	551	4.3	612	0.0	b-spline regression
Finland	4.3	2473	4.1	2441	0.3	b-spline regression
France	7.5	56693	7.4	56972	0.1	hierarchical regression
Gabon	15.3	6303	14.2	8190	0.5	hierarchical regression
Gambia	19.2	10743	16.8	13268	0.9	hierarchical regression
Georgia	6.1	3464	6.1	3372	0.0	b-spline regression
Germany	6.5	48505	6.6	47226	-0.2	b-spline regression
Ghana	16.1	108083	14.2	123251	0.8	hierarchical regression
Greece	9.0	9971	8.7	8244	0.2	hierarchical regression
Guatemala	12.2	50363	11.0	45698	0.7	hierarchical regression
Guinea-Bissau	25.3	13029	21.1	13866	1.2	hierarchical regression
Guyana	16.3	3156	15.6	2486	0.3	hierarchical regression
Honduras	11.9	25353	10.9	21557	0.6	hierarchical regression
Hungary	8.6	8234	8.8	7727	-0.1	b-spline regression
Iceland	3.5	142	4.2	187	-1.3	b-spline regression
Indonesia	11.2	515897	10.0	497589	0.8	hierarchical regression
Ireland	4.9	2817	5.9	4036	-1.3	b-spline regression
Israel	8.3	10622	7.8	12958	0.5	b-spline regression
Italy	7.1	37820	7.0	34486	0.1	hierarchical regression
Jamaica	15.3	8676	14.6	7039	0.3	hierarchical regression
Japan	8.6	100044	9.5	99957	-0.7	b-spline regression
Jordan	14.6	23242	13.8	33504	0.4	hierarchical regression
Kazakhstan	6.1	14507	5.4	20897	0.8	b-spline regression
Kenya	12.3	154672	11.5	172422	0.5	hierarchical regression
Kuwait	10.2	4535	9.9	6384	0.2	hierarchical regression
Kyrgyzstan	6.8	7191	5.5	8414	1.4	b-spline regression
Lao People's Democratic Republic	20.4	34663	17.3	28150	1.1	hierarchical regression
Latvia	5.1	992	4.5	909	0.9	b-spline regression
Lebanon	9.8	6095	9.2	7937	0.4	hierarchical regression
Lesotho	15.4	9128	14.6	8947	0.4	hierarchical regression
Lithuania	4.8	1582	4.5	1405	0.4	hierarchical regression
Luxembourg	6.6	357	6.5	416	0.1	b-spline regression
Madagascar	19.8	128925	17.1	139080	1.0	hierarchical regression
Malawi	17.2	86670	14.5	94488	1.2	hierarchical regression
Malaysia	10.0	50519	11.3	59463	-0.9	b-spline regression
Maldives	15.6	996	11.7	916	1.9	hierarchical regression

Country	Low birthweight rate 2000	Number of low birthweights 2000	Low birthweight rate 2015	Number of low birthweights 2015	2000-2015 ARR %	Source of estimate
Malta	5.9	264	6.3	266	-0.5	b-spline regression
Mauritius	12.7	2550	17.1	2301	-2.0	b-spline regression
Mexico	8.2	200551	7.9	183328	0.3	hierarchical regression
Monaco	5.5	14	5.4	31	0.1	hierarchical regression
Mongolia	6.3	2903	5.4	3934	1.0	hierarchical regression
Montenegro	5.3	443	5.5	394	-0.2	b-spline regression
Morocco	18.8	120339	17.3	122514	0.5	hierarchical regression
Mozambique	16.7	136272	13.8	152668	1.2	hierarchical regression
Myanmar	13.9	156773	12.3	116164	0.8	hierarchical regression
Namibia	16.5	9816	15.5	11129	0.4	hierarchical regression
Nepal	27.2	206633	21.8	124951	1.5	hierarchical regression
Netherlands	7.1	13998	6.2	10982	0.9	b-spline regression
New Zealand	6.6	3658	5.7	3507	0.9	b-spline regression
Nicaragua	11.5	15564	10.7	12934	0.5	hierarchical regression
Norway	4.9	2853	4.5	2765	0.6	b-spline regression
Oman	10.9	6079	10.5	8527	0.2	hierarchical regression
Panama	10.6	7547	10.1	7944	0.3	hierarchical regression
Paraguay	8.6	12335	8.1	11364	0.4	hierarchical regression
Peru	10.1	63271	9.4	57845	0.5	hierarchical regression
Philippines	21.5	498118	20.1	480695	0.4	hierarchical regression
Poland	5.7	21495	5.9	21711	-0.3	b-spline regression
Portugal	7.4	8406	8.9	7382	-1.2	b-spline regression
Qatar	9.1	1073	7.3	1841	1.4	b-spline regression
Republic of Korea	3.8	21878	5.8	25903	-2.7	b-spline regression
Republic of Moldova	5.5	2606	5.0	2137	0.6	hierarchical regression
Romania	8.8	19556	8.2	15710	0.5	hierarchical regression
Russian Federation	7.4	97741	5.8	107536	1.6	b-spline regression
Rwanda	10.3	31773	7.9	29128	1.8	hierarchical regression
San Marino	3.3	6	3.3	8	0.0	hierarchical regression
Sao Tome and Principe	7.2	402	6.6	443	0.6	hierarchical regression
Senegal	22.0	85504	18.5	100096	1.1	hierarchical regression
Serbia	4.7	5563	4.5	4252	0.3	hierarchical regression
Seychelles	9.0	140	11.7	182	-1.7	b-spline regression
Sierra Leone	17.2	36833	14.4	37220	1.2	hierarchical regression
Singapore	9.8	4867	9.6	4779	0.1	hierarchical regression
Slovakia	6.8	3676	7.6	4331	-0.7	b-spline regression
Slovenia	5.6	984	6.1	1315	-0.6	b-spline regression
South Africa	15.0	166887	14.2	167050	0.4	hierarchical regression
Spain	7.0	28181	8.3	34336	-1.1	b-spline regression
Sri Lanka	16.3	56878	15.9	51314	0.2	b-spline regression
Suriname	16.0	1756	14.7	1496	0.6	hierarchical regression
Swaziland	11.1	3846	10.3	3987	0.5	hierarchical regression
Sweden	4.5	4078	2.4	2861	4.0	b-spline regression
Switzerland	6.0	4588	6.5	5589	-0.5	b-spline regression
Tajikistan	6.2	11577	5.6	14101	0.6	hierarchical regression
Thailand	13.5	123614	10.5	76312	1.7	b-spline regression

Country	Low birthweight rate 2000	Number of low birthweights 2000	Low birthweight rate 2015	Number of low birthweights 2015	2000-2015 ARR %	Source of estimate
The former Yugoslav Republic of Macedonia	8.9	2341	9.1	2127	-0.1	b-spline regression
Togo	17.8	34880	16.1	41230	0.7	hierarchical regression
Trinidad and Tobago	13.1	2447	12.4	2348	0.4	hierarchical regression
Tunisia	8.2	13772	7.5	15693	0.6	hierarchical regression
Turkey	12.9	176788	11.4	147483	0.8	hierarchical regression
Turkmenistan	5.4	5753	4.9	7113	0.6	hierarchical regression
Ukraine	5.4	21861	5.6	26996	-0.3	b-spline regression
United Arab Emirates	13.0	6930	12.7	11613	0.2	hierarchical regression
United Kingdom	7.3	50741	7.0	56001	0.3	b-spline regression
United Republic of Tanzania	12.4	179397	10.5	219037	1.1	hierarchical regression
United States of America	7.5	297327	8.0	321060	-0.5	b-spline regression
Uruguay	7.7	4186	7.6	3698	0.1	b-spline regression
Uzbekistan	5.8	32601	5.3	34881	0.6	hierarchical regression
Vanuatu	11.1	670	10.9	756	0.1	hierarchical regression
Venezuela (Bolivarian Republic of)	8.6	50252	9.1	54820	-0.4	b-spline regression
Viet Nam	9.2	130071	8.2	129885	0.8	hierarchical regression
West Bank and Gaza Strip	8.8	10604	8.4	12584	0.3	hierarchical regression
Zambia	13.5	63593	11.6	71942	1.0	hierarchical regression
Zimbabwe	12.4	50797	12.6	67607	-0.1	hierarchical regression

Table 7.2: Estimated low birthweight number and rate globally and by region for low birthweight regions (2000-2015)

Region	Year	Number of live births	Number low birthweight	Uncertainty range		Low birthweight rate	Uncertainty range	
				Lower	Upper		Lower	Upper
Global	2000	130,555,200	22,902,400	18,405,800	27,798,400	17.5	14.1	21.3
	2001	131,054,500	22,716,500	18,275,400	27,729,000	17.3	13.9	21.2
	2002	131,737,600	22,536,700	18,396,400	27,714,600	17.1	14.0	21.0
	2003	132,549,400	22,362,300	18,545,100	26,719,900	16.9	14.0	20.2
	2004	133,444,700	22,197,500	18,043,200	27,027,100	16.6	13.5	20.3
	2005	134,381,800	22,042,100	18,043,200	27,027,100	16.4	13.4	20.1
	2006	135,327,700	21,880,000	17,961,100	26,252,100	16.2	13.3	19.4
	2007	136,258,700	21,724,200	17,981,600	25,662,100	15.9	13.2	18.8
	2008	137,147,100	21,569,700	17,875,900	25,692,100	15.7	13.0	18.7
	2009	137,960,600	21,400,500	17,780,600	25,456,100	15.5	12.9	18.5
	2010	138,670,500	21,231,900	17,917,600	25,347,400	15.3	12.9	18.3
	2011	139,260,300	21,066,100	17,786,500	25,209,400	15.1	12.8	18.1
	2012	139,737,600	20,900,300	17,685,600	24,825,000	15.0	12.7	17.8
	2013	140,111,500	20,744,500	17,457,800	24,500,500	14.8	12.5	17.5
	2014	140,383,200	20,603,300	17,457,800	24,500,500	14.7	12.4	17.5
	2015	140,554,200	20,469,700	17,375,000	24,017,900	14.6	12.4	17.1
North America, Europe, Australia and New Zealand	2000	11,919,500	832,900	813,800	856,600	7.0	6.8	7.2
	2001	11,956,300	839,600	823,100	860,500	7.0	6.9	7.2
	2002	12,037,800	848,400	833,600	870,200	7.0	6.9	7.2
	2003	12,152,400	858,600	842,200	880,700	7.1	6.9	7.2
	2004	12,288,300	869,500	853,400	889,900	7.1	6.9	7.2
	2005	12,429,200	879,900	864,200	900,300	7.1	7.0	7.2
	2006	12,558,100	888,700	873,900	910,400	7.1	7.0	7.2
	2007	12,663,500	895,300	878,800	916,600	7.1	6.9	7.2
	2008	12,738,000	899,300	883,400	918,900	7.1	6.9	7.2
	2009	12,777,700	900,600	885,000	920,600	7.0	6.9	7.2
	2010	12,784,800	899,500	884,200	920,400	7.0	6.9	7.2
	2011	12,767,700	896,700	880,800	918,000	7.0	6.9	7.2
	2012	12,740,200	893,300	879,300	913,200	7.0	6.9	7.2
	2013	12,713,400	890,000	874,700	911,300	7.0	6.9	7.2
	2014	12,691,600	887,000	872,000	908,100	7.0	6.9	7.2
	2015	12,676,500	884,400	866,900	905,600	7.0	6.8	7.1
Northern Africa	2000	4,391,300	602,400	458,800	846,700	13.7	10.4	19.3
	2001	4,432,400	604,200	462,100	852,400	13.6	10.4	19.2
	2002	4,486,700	607,200	461,000	872,400	13.5	10.3	19.4
	2003	4,551,700	610,900	471,600	880,800	13.4	10.4	19.4
	2004	4,627,300	615,500	481,500	885,500	13.3	10.4	19.1
	2005	4,717,800	622,100	480,300	888,700	13.2	10.2	18.8
	2006	4,829,400	630,800	478,100	903,200	13.1	9.9	18.7
	2007	4,963,200	642,000	494,300	946,700	12.9	10.0	19.1
	2008	5,115,500	655,200	496,300	935,000	12.8	9.7	18.3
	2009	5,279,100	670,100	503,800	949,500	12.7	9.5	18.0
	2010	5,440,800	684,800	517,500	1,011,700	12.6	9.5	18.6
	2011	5,585,400	697,900	533,700	987,100	12.5	9.6	17.7
	2012	5,701,200	707,500	543,900	1,015,900	12.4	9.5	17.8
	2013	5,780,800	713,300	551,000	1,031,600	12.3	9.5	17.8

Region	Year	Number of live births	Number low birthweight	Uncertainty range		Low birthweight rate	Uncertainty range	
				Lower	Upper		Lower	Upper
	2014	5,821,700	715,000	521,200	1,059,900	12.3	9.0	18.2
	2015	5,826,100	712,600	546,300	1,043,500	12.2	9.4	17.9
Sub-Saharan Africa (Southern/East/West/Middle Africa)	2000	26,994,800	4,436,000	3,729,700	5,499,000	16.4	13.8	20.4
	2001	27,569,600	4,480,800	3,777,600	5,492,300	16.3	13.7	19.9
	2002	28,151,800	4,522,900	3,782,700	5,559,400	16.1	13.4	19.7
	2003	28,742,100	4,562,500	3,856,800	5,624,600	15.9	13.4	19.6
	2004	29,339,000	4,599,300	3,906,800	5,692,200	15.7	13.3	19.4
	2005	29,941,100	4,634,400	3,927,000	5,800,500	15.5	13.1	19.4
	2006	30,546,900	4,669,600	3,950,900	5,654,500	15.3	12.9	18.5
	2007	31,153,800	4,706,300	4,022,600	5,720,500	15.1	12.9	18.4
	2008	31,758,900	4,743,200	4,066,500	5,833,900	14.9	12.8	18.4
	2009	32,359,400	4,780,400	4,093,100	5,903,000	14.8	12.6	18.2
	2010	32,951,700	4,819,400	4,165,100	5,940,500	14.6	12.6	18.0
	2011	33,532,700	4,857,800	4,208,600	5,979,200	14.5	12.6	17.8
	2012	34,102,000	4,893,900	4,229,100	5,994,600	14.4	12.4	17.6
	2013	34,660,200	4,929,900	4,256,100	6,028,000	14.2	12.3	17.4
	2014	35,208,400	4,965,300	4,328,600	6,155,600	14.1	12.3	17.5
	2015	35,748,700	5,000,100	4,349,600	6,146,300	14.0	12.2	17.2
Central Asia	2000	1,203,800	71,700	62,000	83,500	6.0	5.1	6.9
	2001	1,194,800	69,600	60,200	82,800	5.8	5.0	6.9
	2002	1,199,800	68,700	59,300	80,200	5.7	4.9	6.7
	2003	1,217,700	68,800	59,600	80,900	5.6	4.9	6.6
	2004	1,247,200	69,800	61,200	81,500	5.6	4.9	6.5
	2005	1,286,700	71,700	63,000	82,600	5.6	4.9	6.4
	2006	1,333,800	74,200	65,400	85,700	5.6	4.9	6.4
	2007	1,384,700	77,200	67,500	89,800	5.6	4.9	6.5
	2008	1,435,500	80,200	71,200	92,900	5.6	5.0	6.5
	2009	1,482,900	83,000	73,200	96,100	5.6	4.9	6.5
	2010	1,523,600	85,500	76,100	97,000	5.6	5.0	6.4
	2011	1,555,600	87,200	77,700	98,400	5.6	5.0	6.3
	2012	1,578,600	88,100	78,600	100,800	5.6	5.0	6.4
	2013	1,592,900	88,200	78,500	100,300	5.5	4.9	6.3
	2014	1,598,400	87,300	78,300	100,300	5.5	4.9	6.3
	2015	1,595,200	85,500	76,200	96,700	5.4	4.8	6.1
Southern Asia	2000	39,312,400	12,694,600	8,800,300	17,292,700	32.3	22.4	44.0
	2001	39,300,300	12,493,000	8,552,900	17,199,800	31.8	21.8	43.8
	2002	39,287,700	12,291,400	8,537,900	16,610,700	31.3	21.7	42.3
	2003	39,273,300	12,091,400	8,362,100	16,374,700	30.8	21.3	41.7
	2004	39,251,300	11,898,600	8,252,700	16,435,300	30.3	21.0	41.9
	2005	39,205,000	11,710,400	7,902,800	16,027,800	29.9	20.2	40.9
	2006	39,113,700	11,512,300	8,143,700	15,406,000	29.4	20.8	39.4
	2007	38,966,500	11,313,100	7,818,700	15,214,900	29.0	20.1	39.0
	2008	38,762,300	11,112,600	7,849,500	14,926,300	28.7	20.3	38.5
	2009	38,509,000	10,893,800	7,632,900	14,782,900	28.3	19.8	38.4
	2010	38,227,700	10,676,200	7,573,100	14,701,100	27.9	19.8	38.5
	2011	37,946,800	10,467,000	7,397,200	14,190,900	27.6	19.5	37.4
	2012	37,692,900	10,270,500	7,123,400	13,991,700	27.2	18.9	37.1
	2013	37,483,600	10,093,900	7,131,100	13,914,700	26.9	19.0	37.1
	2014	37,323,900	9,941,900	7,148,800	13,649,600	26.6	19.2	36.6

Region	Year	Number of live births	Number low birthweight	Uncertainty range		Low birthweight rate	Uncertainty range	
				Lower	Upper		Lower	Upper
	2015	37,208,500	9,807,400	6,913,700	13,104,600	26.4	18.6	35.2
Eastern Asia	2000	18,513,800	1,111,000	900,100	1,364,100	6.0	4.9	7.4
	2001	18,385,100	1,094,400	891,600	1,375,400	6.0	4.8	7.5
	2002	18,353,100	1,080,300	871,500	1,328,400	5.9	4.7	7.2
	2003	18,385,300	1,069,600	873,200	1,313,200	5.8	4.7	7.1
	2004	18,458,300	1,060,500	868,600	1,313,100	5.7	4.7	7.1
	2005	18,558,400	1,054,300	862,200	1,286,800	5.7	4.6	6.9
	2006	18,681,600	1,049,500	856,200	1,313,000	5.6	4.6	7.0
	2007	18,827,700	1,047,700	854,000	1,306,700	5.6	4.5	6.9
	2008	18,988,100	1,046,900	863,800	1,304,600	5.5	4.5	6.9
	2009	19,144,600	1,047,100	865,100	1,304,300	5.5	4.5	6.8
	2010	19,274,100	1,046,200	849,600	1,295,300	5.4	4.4	6.7
	2011	19,355,700	1,044,900	843,300	1,271,300	5.4	4.4	6.6
	2012	19,378,900	1,040,500	853,800	1,278,300	5.4	4.4	6.6
	2013	19,337,200	1,033,400	848,600	1,259,000	5.3	4.4	6.5
	2014	19,226,900	1,023,400	838,200	1,270,500	5.3	4.4	6.6
	2015	19,047,600	1,010,600	822,600	1,264,800	5.3	4.3	6.6
Western Asia	2000	4,877,700	532,300	437,400	667,200	10.9	9.0	13.7
	2001	4,902,100	531,100	440,100	650,300	10.8	9.0	13.3
	2002	4,934,100	530,700	436,100	655,300	10.8	8.8	13.3
	2003	4,974,700	530,700	436,800	670,700	10.7	8.8	13.5
	2004	5,023,900	531,400	439,800	662,200	10.6	8.8	13.2
	2005	5,082,100	533,000	439,200	669,600	10.5	8.6	13.2
	2006	5,148,900	535,700	440,000	682,100	10.4	8.5	13.2
	2007	5,221,600	539,200	442,500	679,500	10.3	8.5	13.0
	2008	5,296,900	543,200	450,200	674,600	10.3	8.5	12.7
	2009	5,371,600	547,300	441,700	696,800	10.2	8.2	13.0
	2010	5,441,700	551,100	447,900	689,300	10.1	8.2	12.7
	2011	5,503,200	554,700	452,800	701,400	10.1	8.2	12.7
	2012	5,554,300	557,200	457,700	696,200	10.0	8.2	12.5
	2013	5,594,000	559,000	457,400	712,600	10.0	8.2	12.7
	2014	5,622,100	560,000	461,000	715,100	10.0	8.2	12.7
	2015	5,639,400	560,200	456,400	703,000	9.9	8.1	12.5
South-east Asia and Oceania (excluding Australia and New Zealand)	2000	11,735,300	1,598,600	1,190,300	1,947,200	13.6	10.1	16.6
	2001	11,788,700	1,586,900	1,201,000	1,908,000	13.5	10.2	16.2
	2002	11,850,800	1,577,300	1,198,900	1,894,100	13.3	10.1	16.0
	2003	11,910,100	1,568,000	1,192,000	1,863,400	13.2	10.0	15.6
	2004	11,961,200	1,559,400	1,187,500	1,862,900	13.0	9.9	15.6
	2005	12,001,900	1,550,900	1,194,100	1,864,200	12.9	9.9	15.5
	2006	12,033,400	1,541,900	1,198,600	1,827,600	12.8	10.0	15.2
	2007	12,060,300	1,534,200	1,184,100	1,831,900	12.7	9.8	15.2
	2008	12,085,200	1,526,600	1,196,000	1,825,500	12.6	9.9	15.1
	2009	12,107,300	1,520,100	1,183,800	1,818,900	12.6	9.8	15.0
	2010	12,124,200	1,513,700	1,181,000	1,815,000	12.5	9.7	15.0
	2011	12,133,000	1,507,200	1,170,200	1,797,500	12.4	9.6	14.8
	2012	12,131,400	1,499,600	1,178,000	1,792,100	12.4	9.7	14.8
	2013	12,118,600	1,491,100	1,182,700	1,811,900	12.3	9.8	15.0
	2014	12,094,900	1,481,600	1,154,900	1,760,300	12.2	9.5	14.6
	2015	12,061,800	1,471,000	1,151,700	1,763,800	12.2	9.5	14.6

Region	Year	Number of live births	Number low birthweight	Uncertainty range		Low birthweight rate	Uncertainty range	
				Lower	Upper		Lower	Upper
Latin America and the Caribbean	2000	11,606,900	1,023,300	945,800	1,113,500	8.8	8.1	9.6
	2001	11,525,600	1,017,300	942,300	1,115,900	8.8	8.2	9.7
	2002	11,436,100	1,010,100	933,500	1,106,000	8.8	8.2	9.7
	2003	11,342,500	1,002,200	929,200	1,090,100	8.8	8.2	9.6
	2004	11,248,600	993,900	926,000	1,088,800	8.8	8.2	9.7
	2005	11,160,000	985,800	912,000	1,076,300	8.8	8.2	9.6
	2006	11,082,300	977,600	903,700	1,069,200	8.8	8.2	9.6
	2007	11,017,800	969,600	898,400	1,063,900	8.8	8.2	9.7
	2008	10,967,100	963,100	893,700	1,053,200	8.8	8.1	9.6
	2009	10,929,500	958,600	887,500	1,050,900	8.8	8.1	9.6
	2010	10,902,200	955,700	885,100	1,053,200	8.8	8.1	9.7
	2011	10,880,500	953,200	888,100	1,042,000	8.8	8.2	9.6
	2012	10,858,500	949,900	870,800	1,040,300	8.7	8.0	9.6
	2013	10,831,100	946,100	879,700	1,046,100	8.7	8.1	9.7
	2014	10,795,700	942,200	876,000	1,036,700	8.7	8.1	9.6
	2015	10,751,000	938,300	871,500	1,032,100	8.7	8.1	9.6

Figure 7.3: Graphs by country of data inputs and low birthweight rate estimates with associated uncertainty

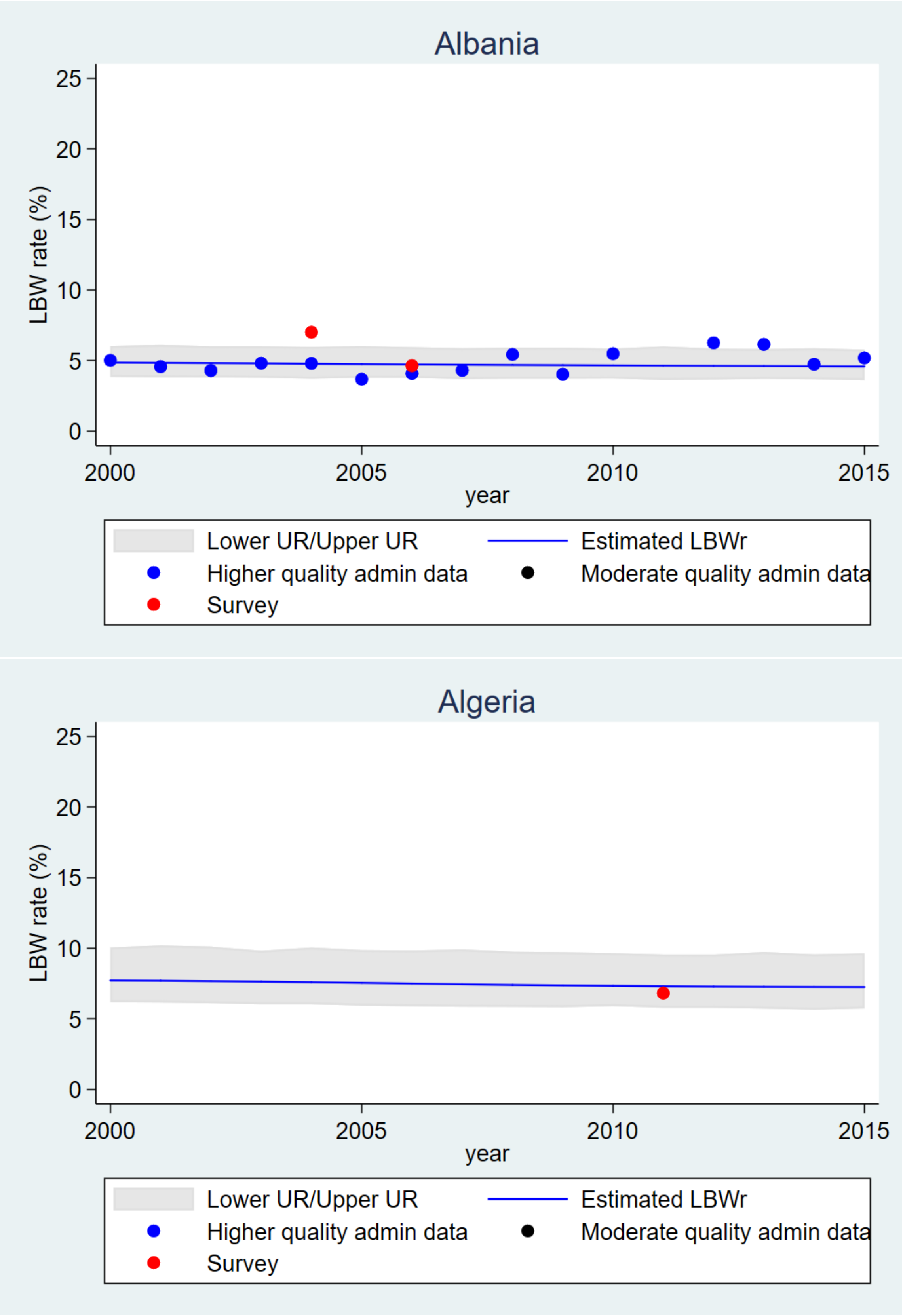

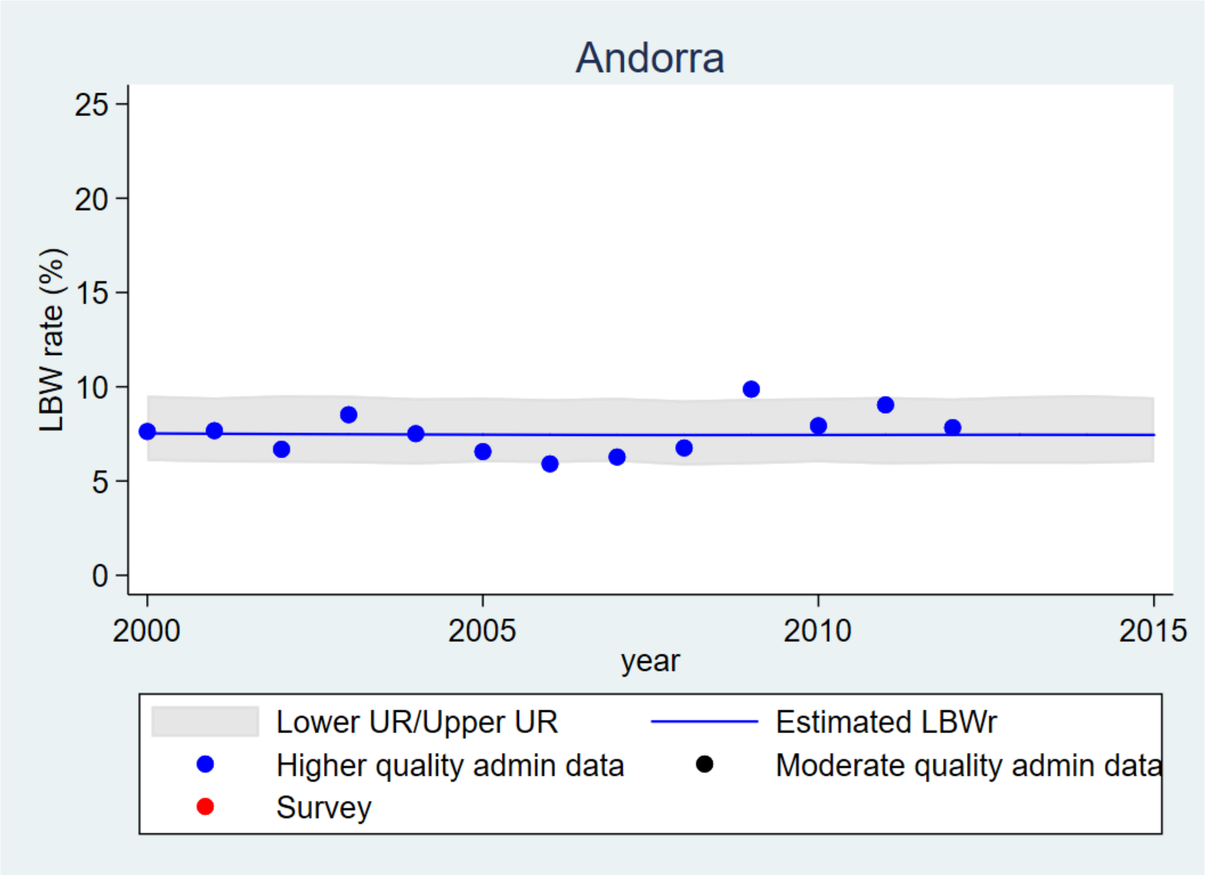

Andorra
LBW rate (%)
25
20
15
10
5
0
2000
2005
2010
2015
year
Lower UR/Upper UR
Estimated LBWr
Higher quality admin data
Moderate quality admin data
Survey

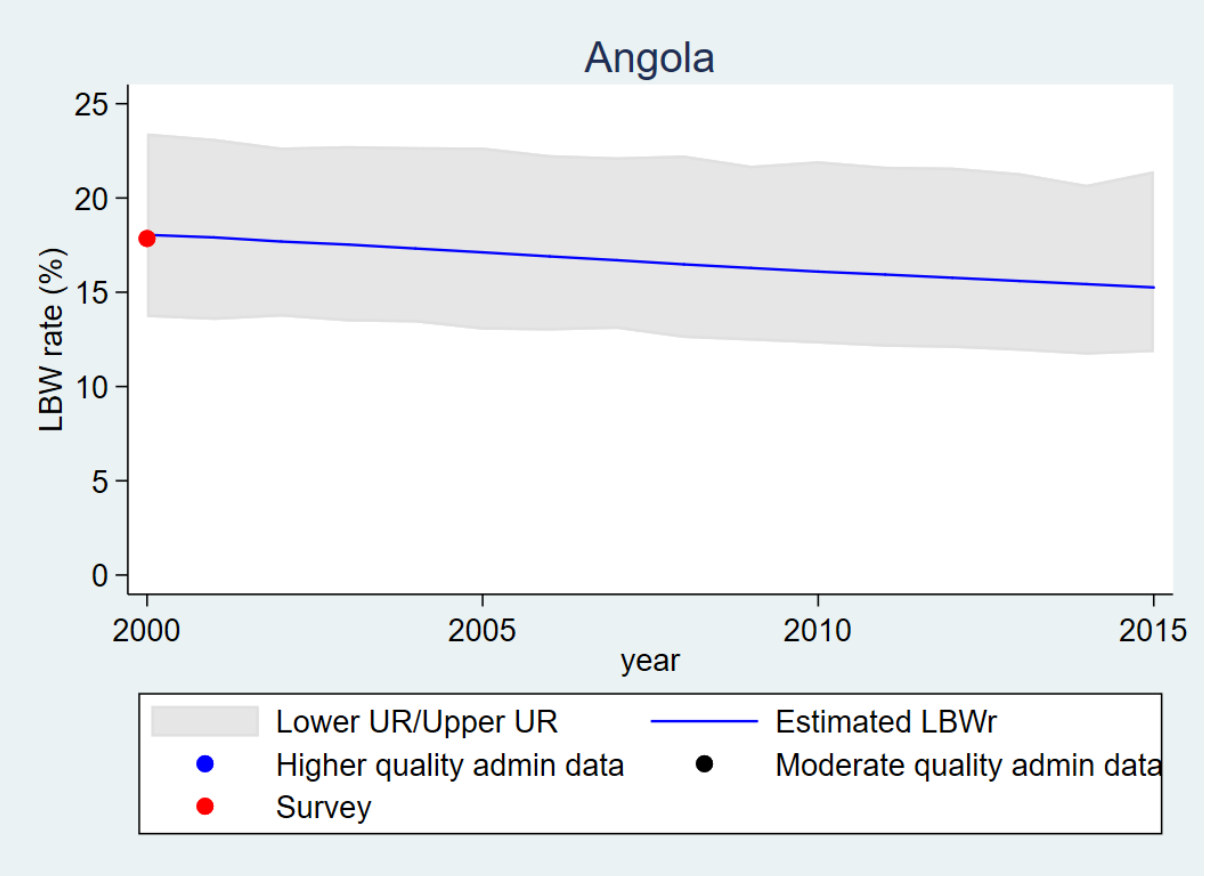

Angola
LBW rate (%)
25
20
15
10
5
0
2000
2005
2010
2015
year
Lower UR/Upper UR
Estimated LBWr
Higher quality admin data
Moderate quality admin data
Survey

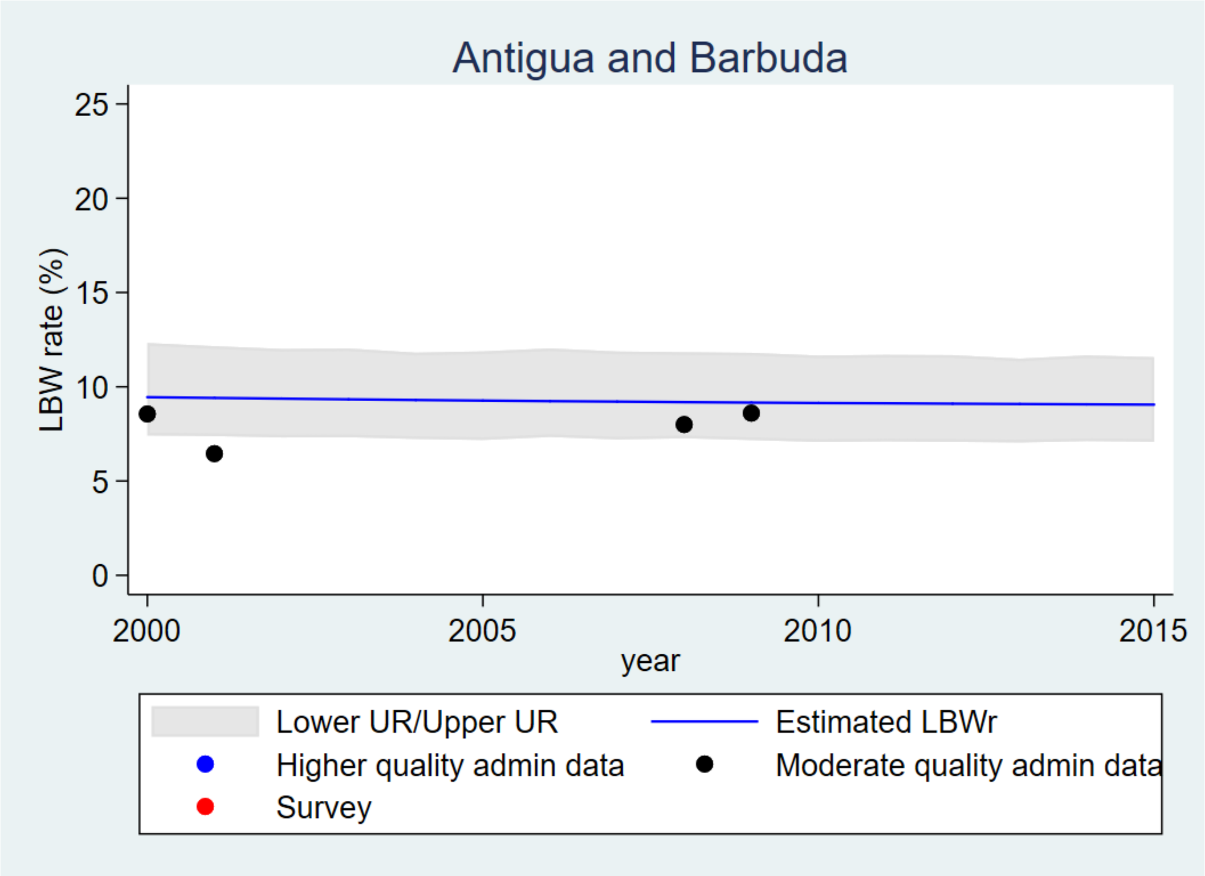

Antigua and Barbuda
LBW rate (%)
0
5
10
15
20
25
2000
2005
2010
2015
year
Lower UR/Upper UR
Estimated LBWr
Higher quality admin data
Moderate quality admin data
Survey

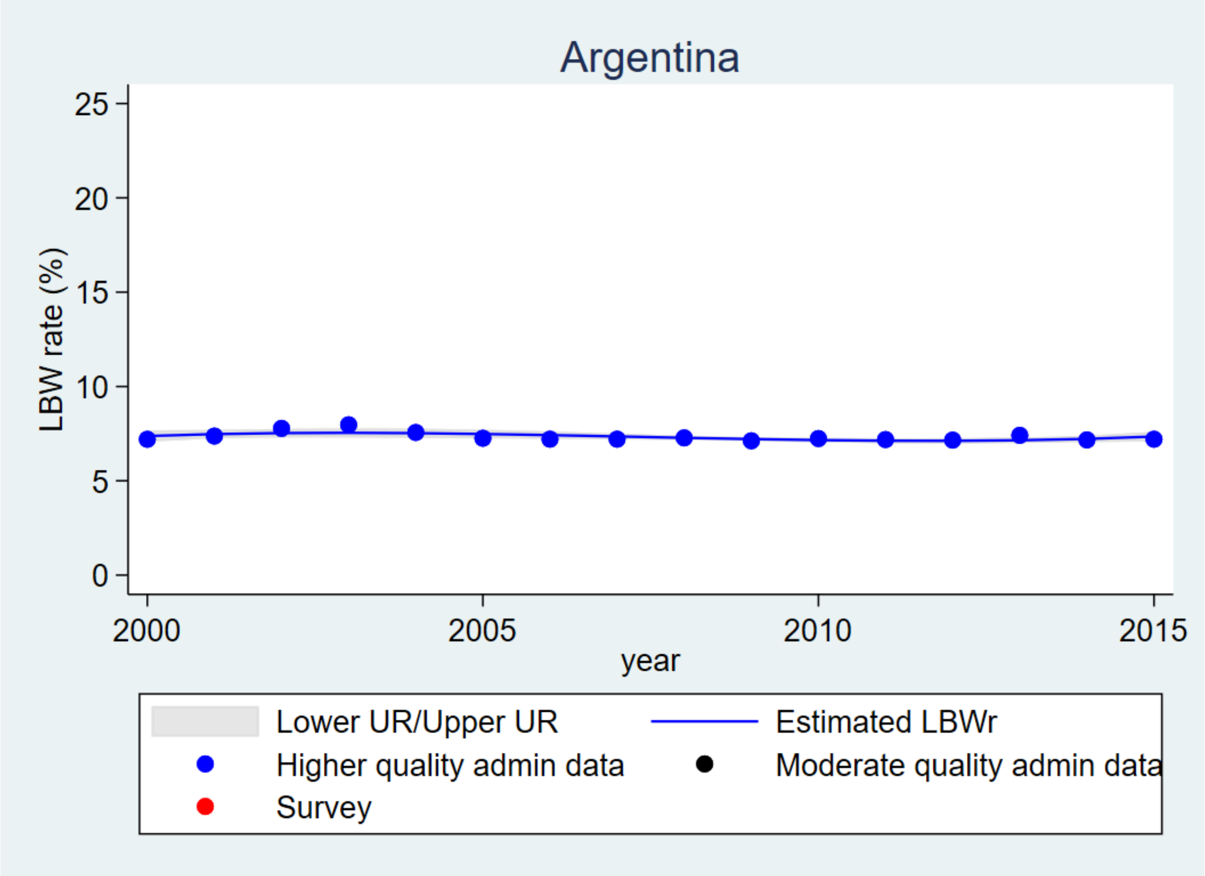

Argentina
LBW rate (%)
0
5
10
15
20
25
2000
2005
2010
2015
year
Lower UR/Upper UR
Estimated LBWr
Higher quality admin data
Moderate quality admin data
Survey

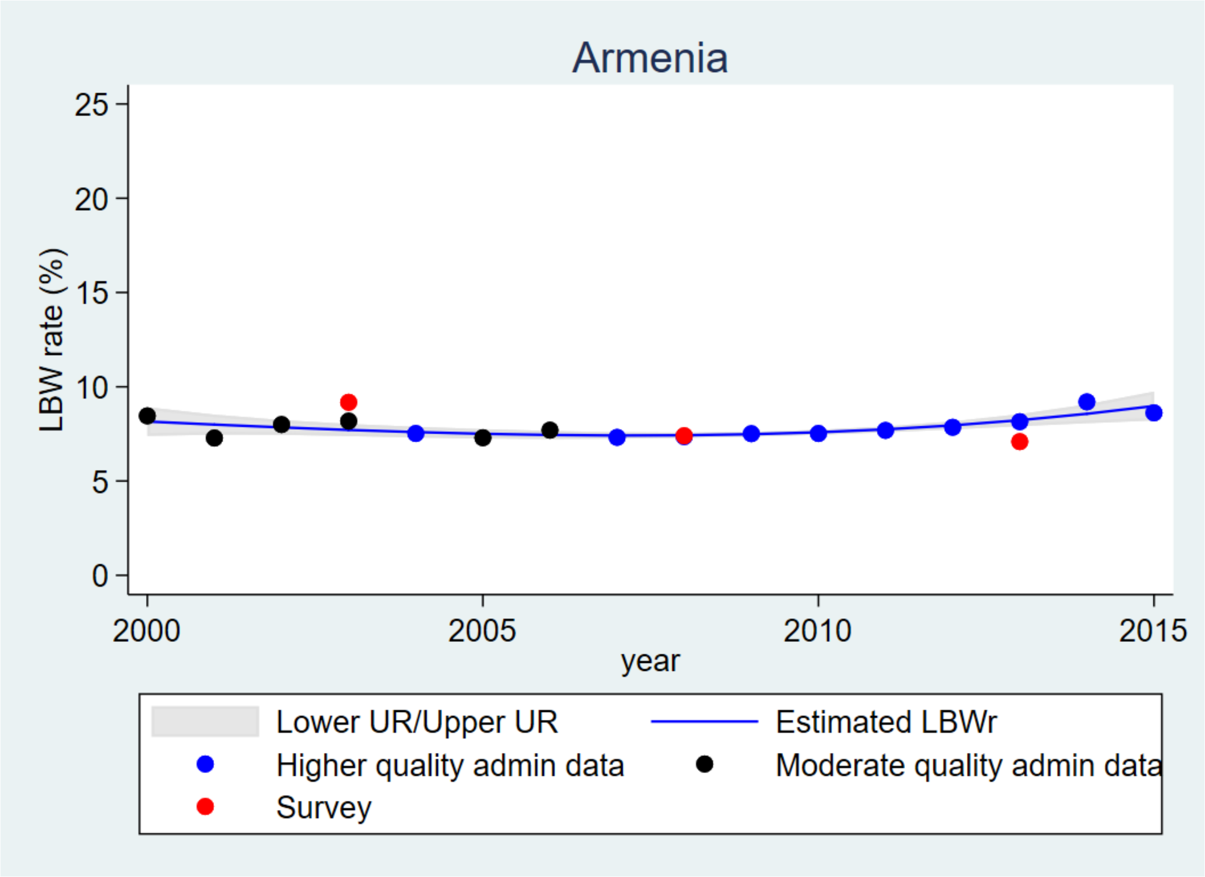

Armenia
LBW rate (%)
25
20
15
10
5
0
2000
2005
2010
2015
year
Lower UR/Upper UR
Estimated LBWr
Higher quality admin data
Moderate quality admin data
Survey

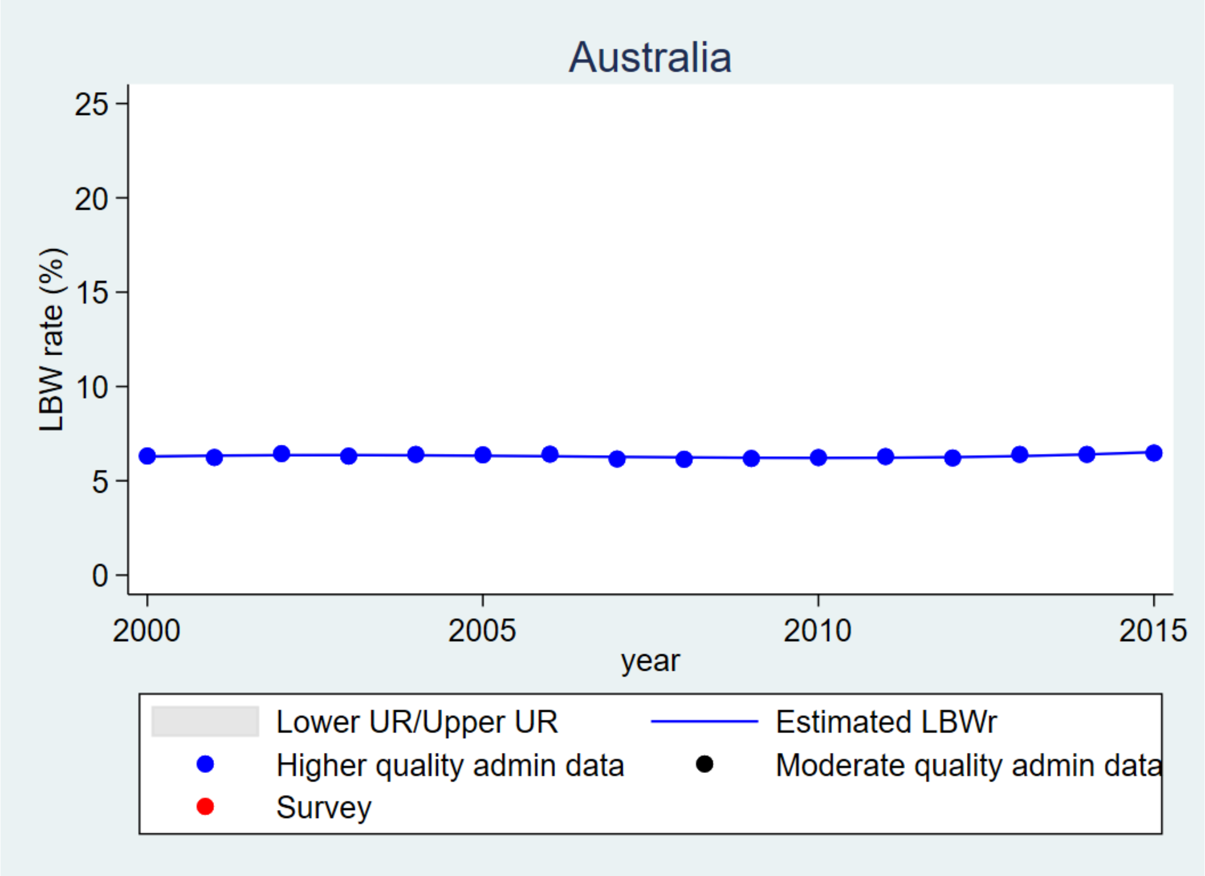

Australia
LBW rate (%)
25
20
15
10
5
0
2000
2005
2010
2015
year
Lower UR/Upper UR
Estimated LBWr
Higher quality admin data
Moderate quality admin data
Survey

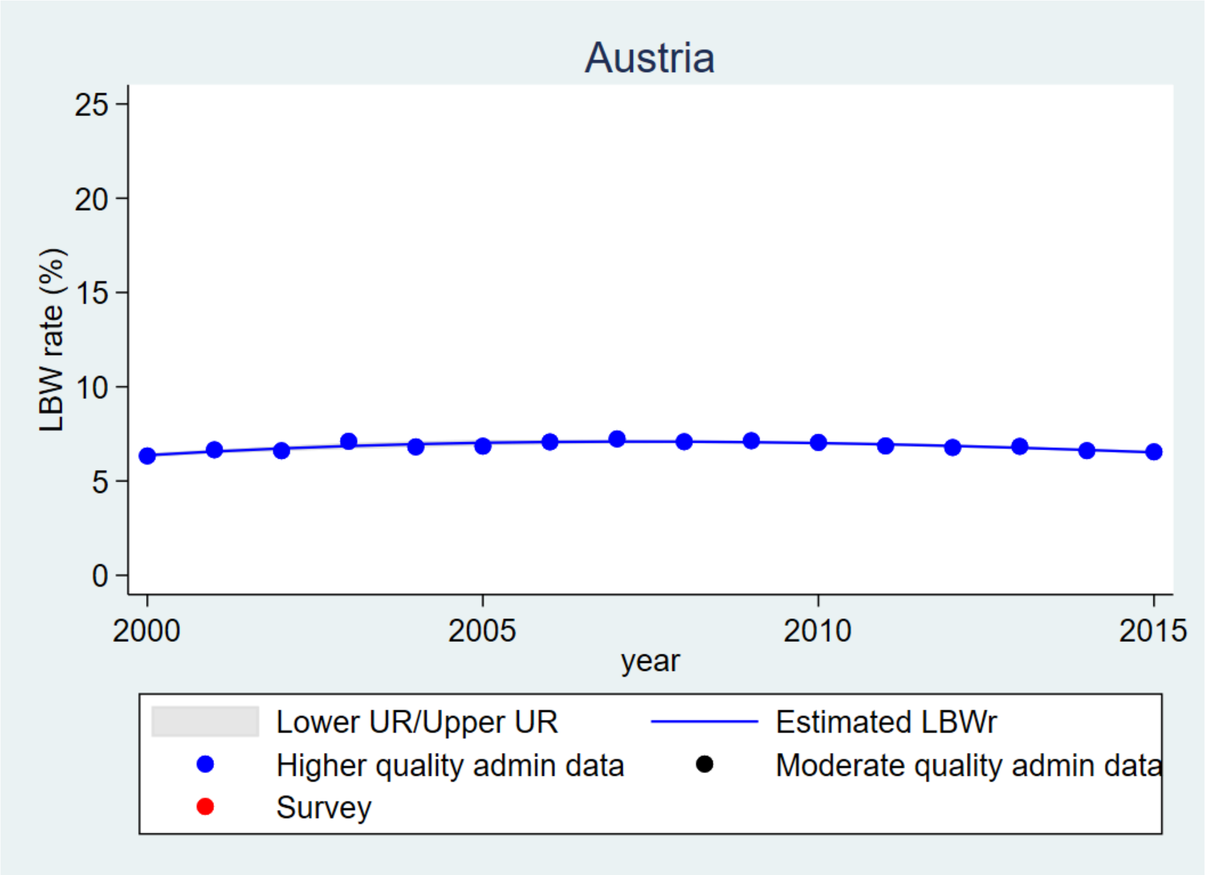
Austria
LBW rate (%)
25
20
15
10
5
0
2000
2005
2010
2015
year
Lower UR/Upper UR
Estimated LBWr
Higher quality admin data
Moderate quality admin data
Survey

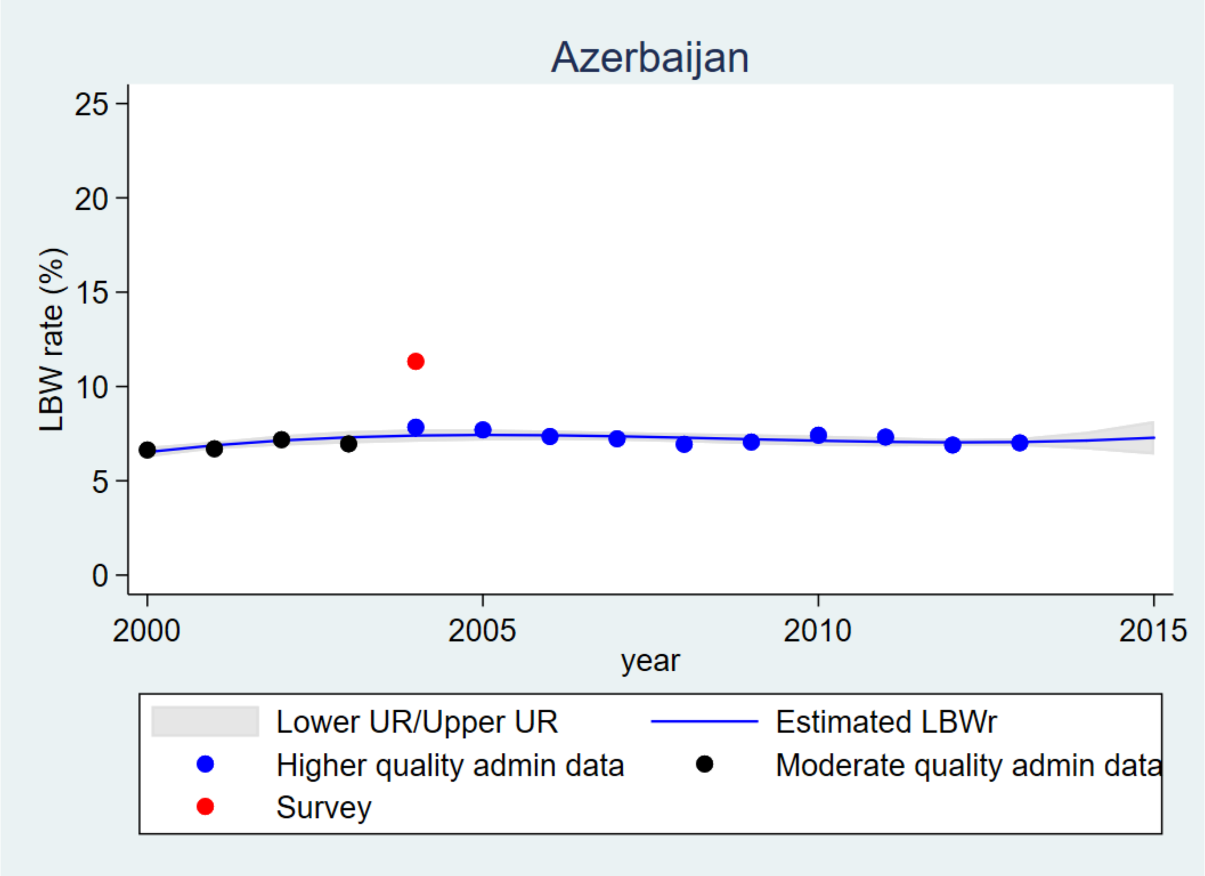
Azerbaijan
LBW rate (%)
25
20
15
10
5
0
2000
2005
2010
2015
year
Lower UR/Upper UR
Estimated LBWr
Higher quality admin data
Moderate quality admin data
Survey

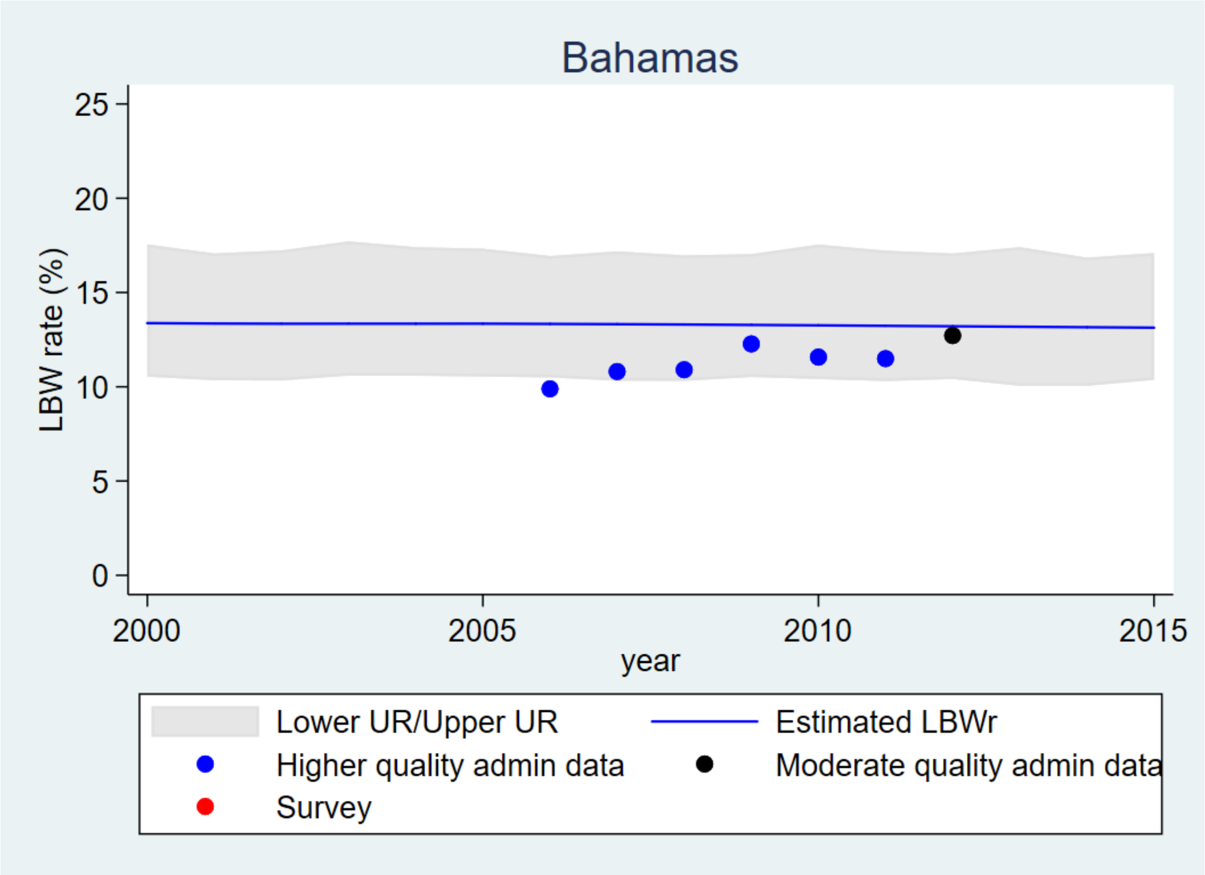
Bahamas
LBW rate (%)
25
20
15
10
5
0
2000
2005
2010
2015
year
Lower UR/Upper UR
Estimated LBWr
Higher quality admin data
Moderate quality admin data
Survey

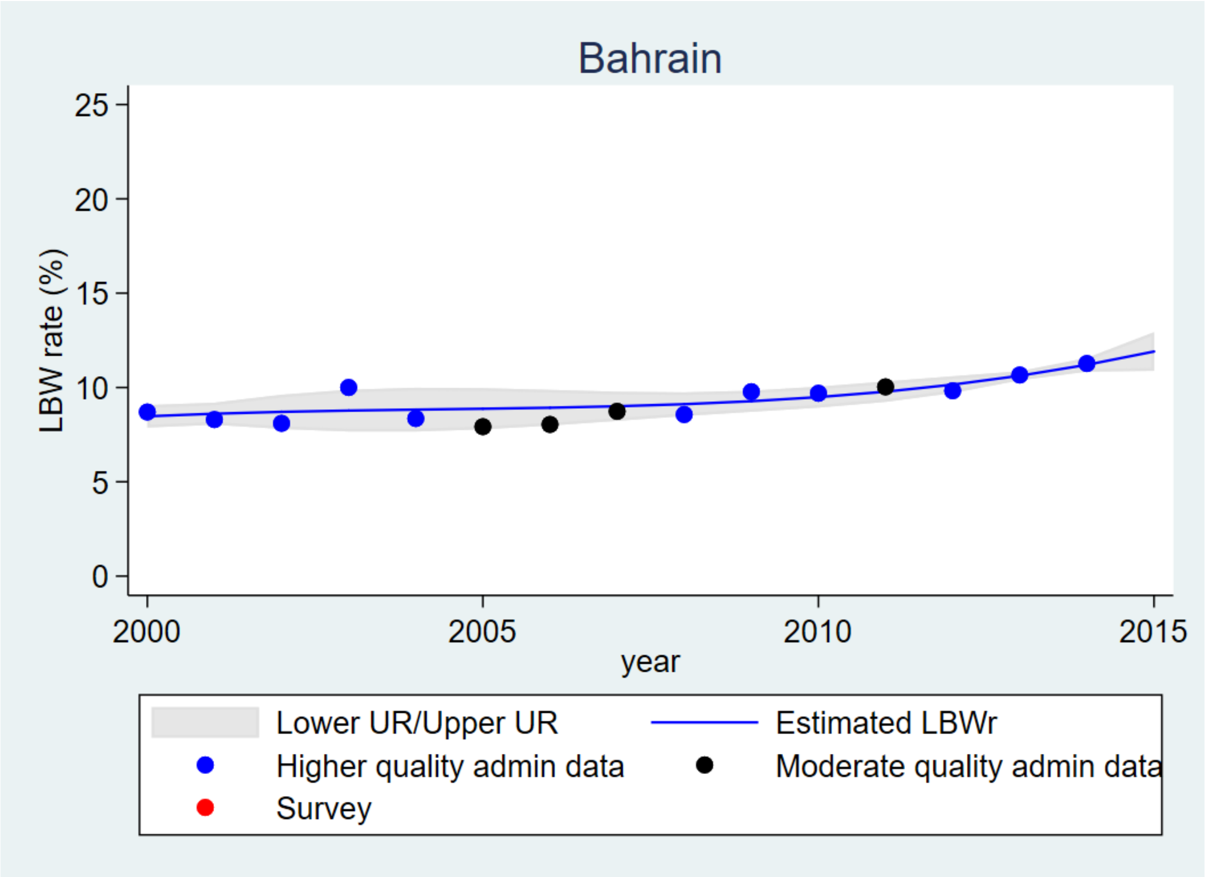
Bahrain
LBW rate (%)
25
20
15
10
5
0
2000
2005
2010
2015
year
Lower UR/Upper UR
Estimated LBWr
Higher quality admin data
Moderate quality admin data
Survey

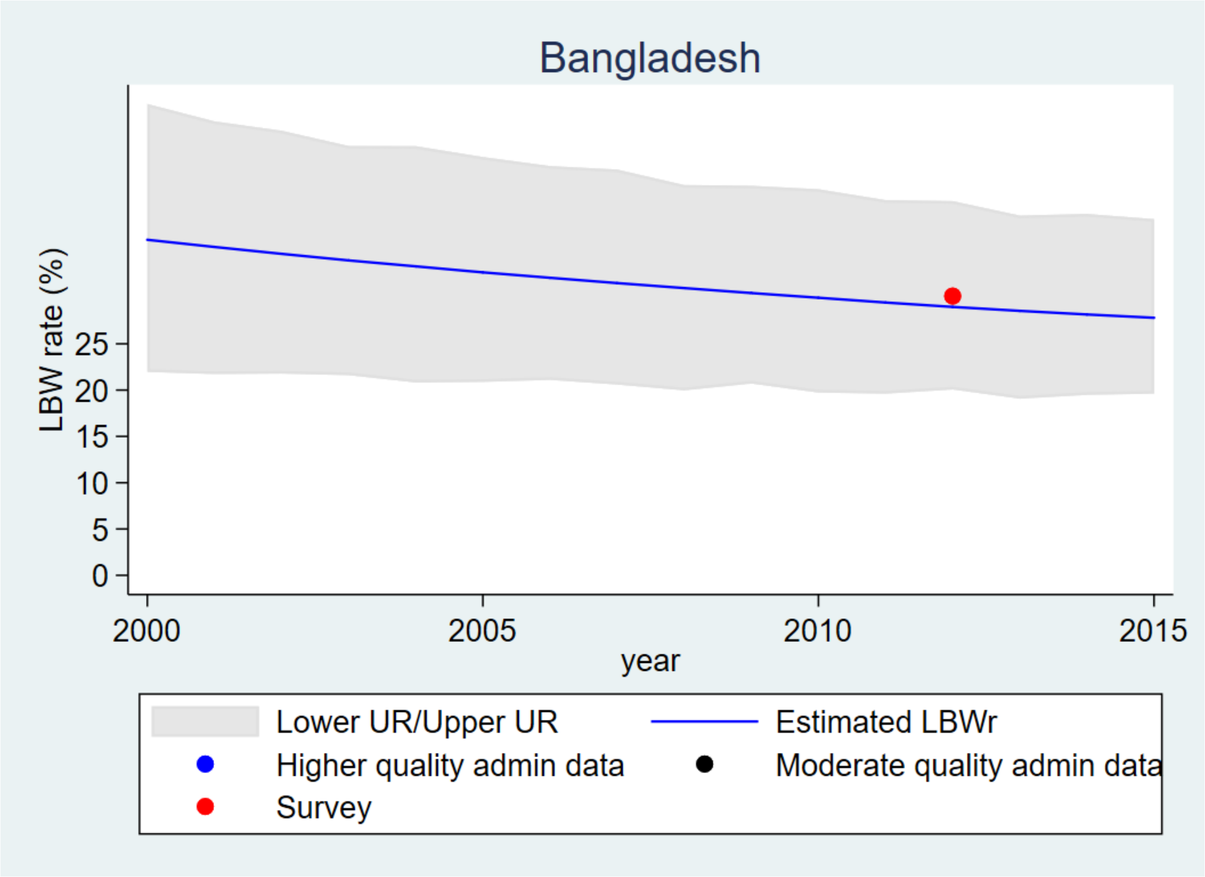
Bangladesh
LBW rate (%)
0
5
10
15
20
25
2000
2005
2010
2015
year
Lower UR/Upper UR
Estimated LBWr
Higher quality admin data
Moderate quality admin data
Survey

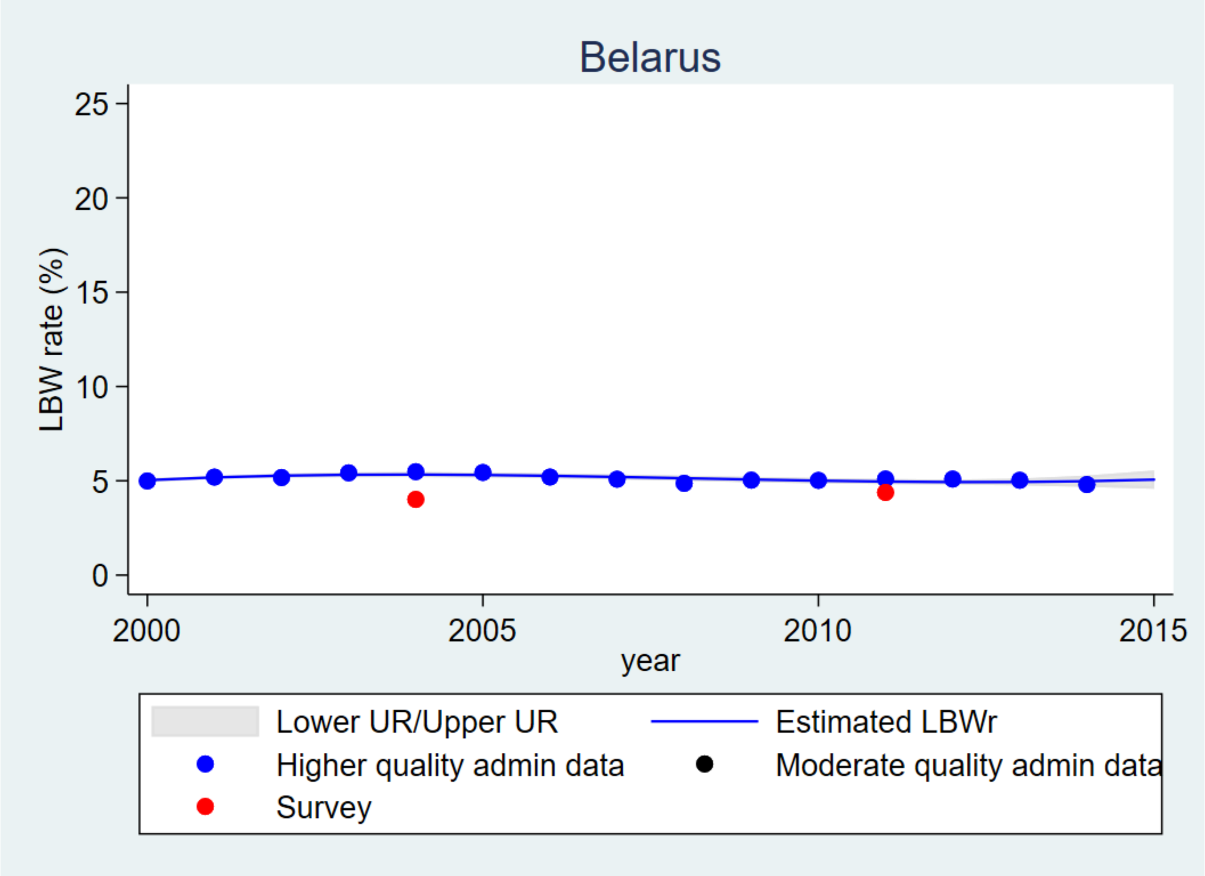
Belarus
LBW rate (%)
0
5
10
15
20
25
2000
2005
2010
2015
year
Lower UR/Upper UR
Estimated LBWr
Higher quality admin data
Moderate quality admin data
Survey

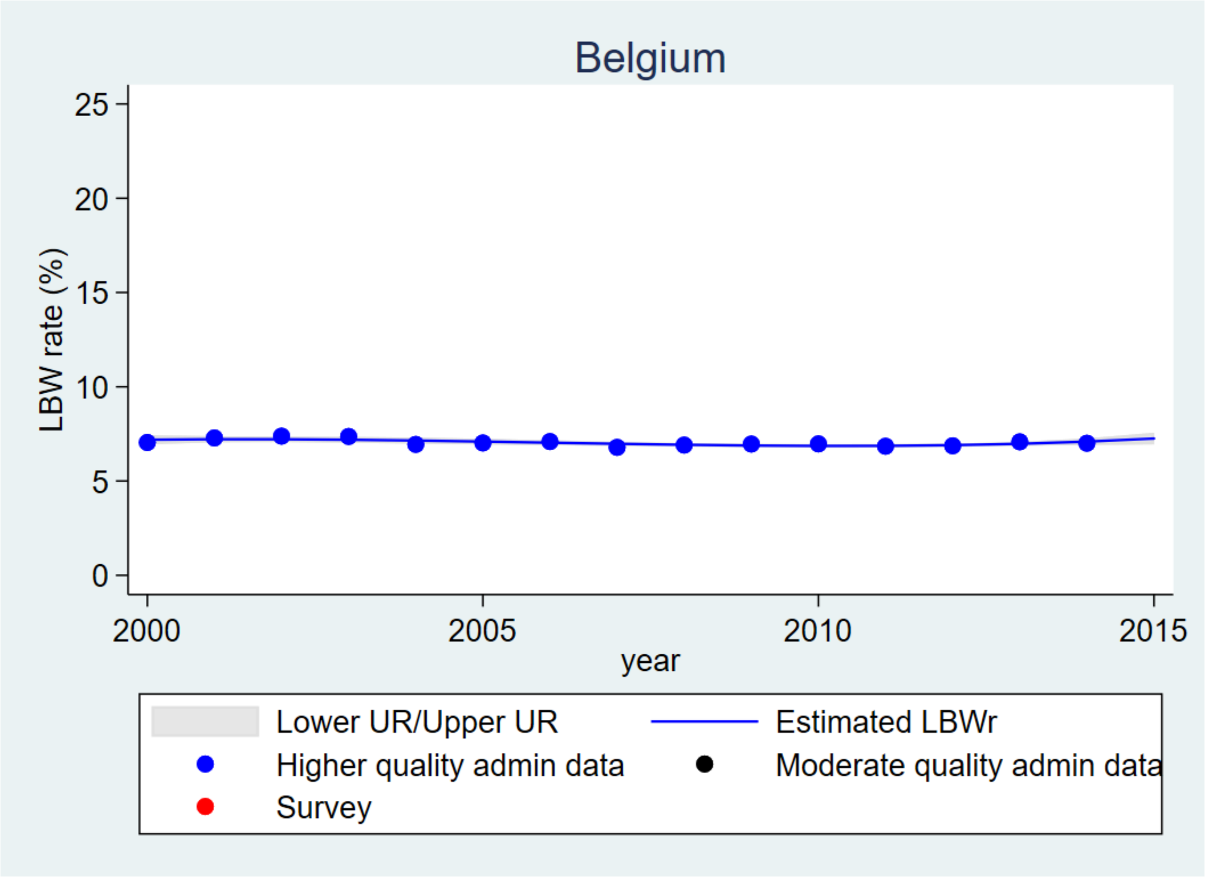
Belgium
LBW rate (%)
25
20
15
10
5
0
2000
2005
2010
2015
year
Lower UR/Upper UR
Estimated LBWr
Higher quality admin data
Moderate quality admin data
Survey

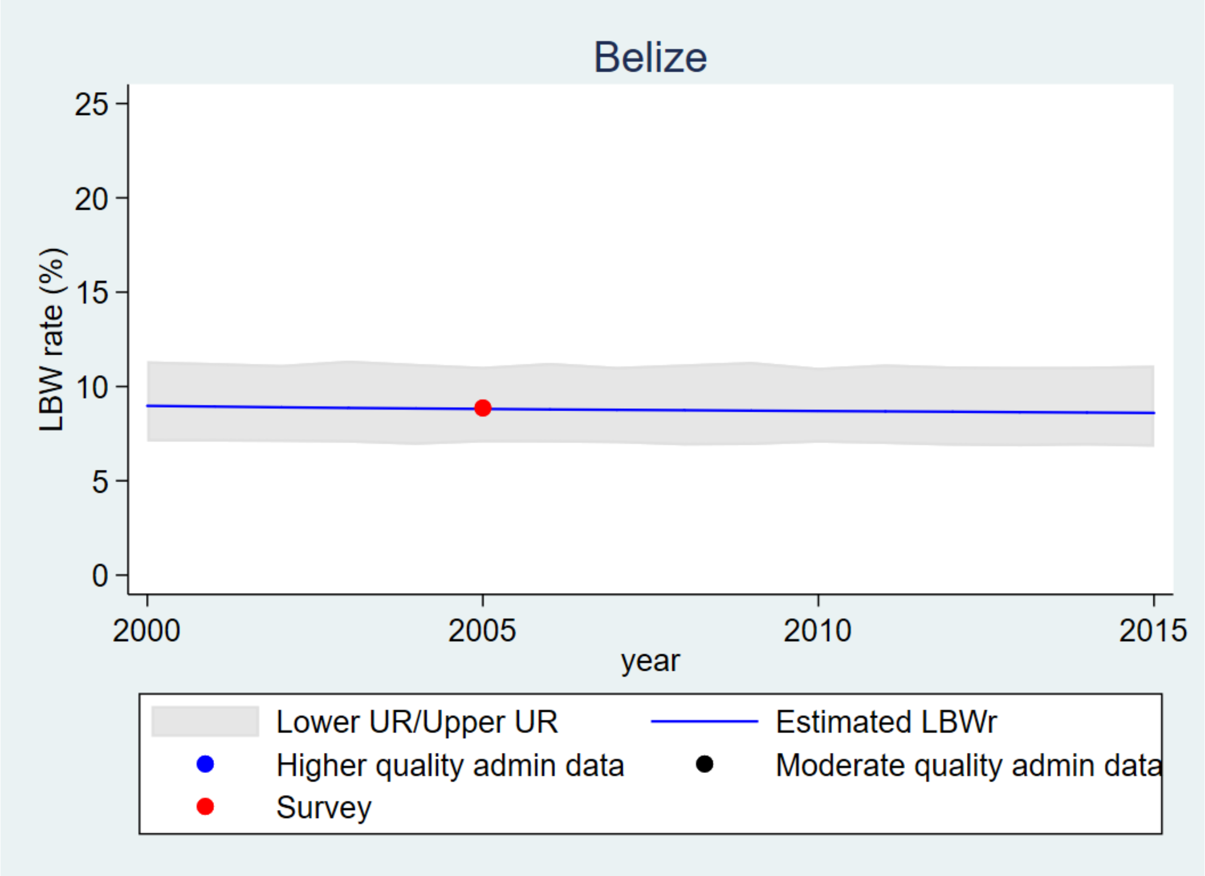
Belize
LBW rate (%)
25
20
15
10
5
0
2000
2005
2010
2015
year
Lower UR/Upper UR
Estimated LBWr
Higher quality admin data
Moderate quality admin data
Survey

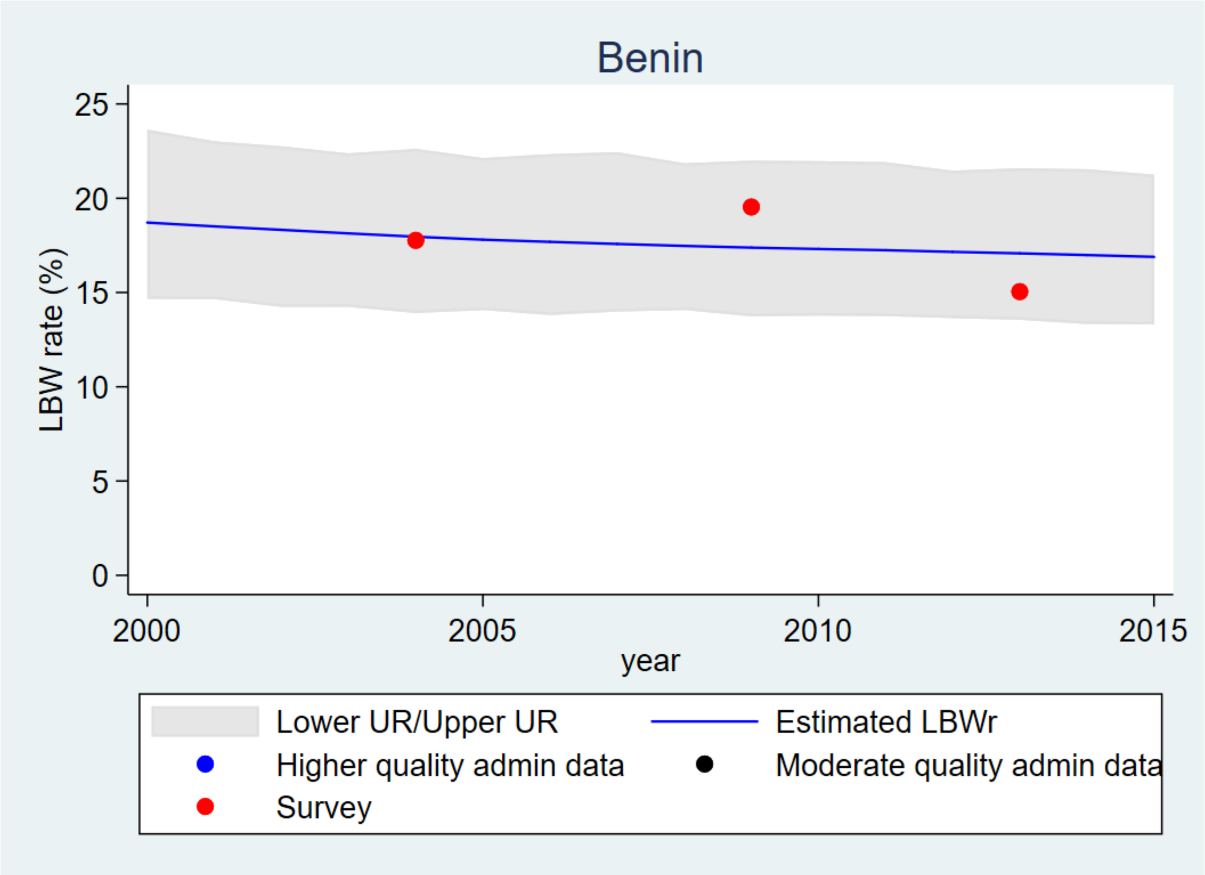
Benin
LBW rate (%)
25
20
15
10
5
0
2000
2005
2010
2015
year
Lower UR/Upper UR
Estimated LBWr
Higher quality admin data
Moderate quality admin data
Survey

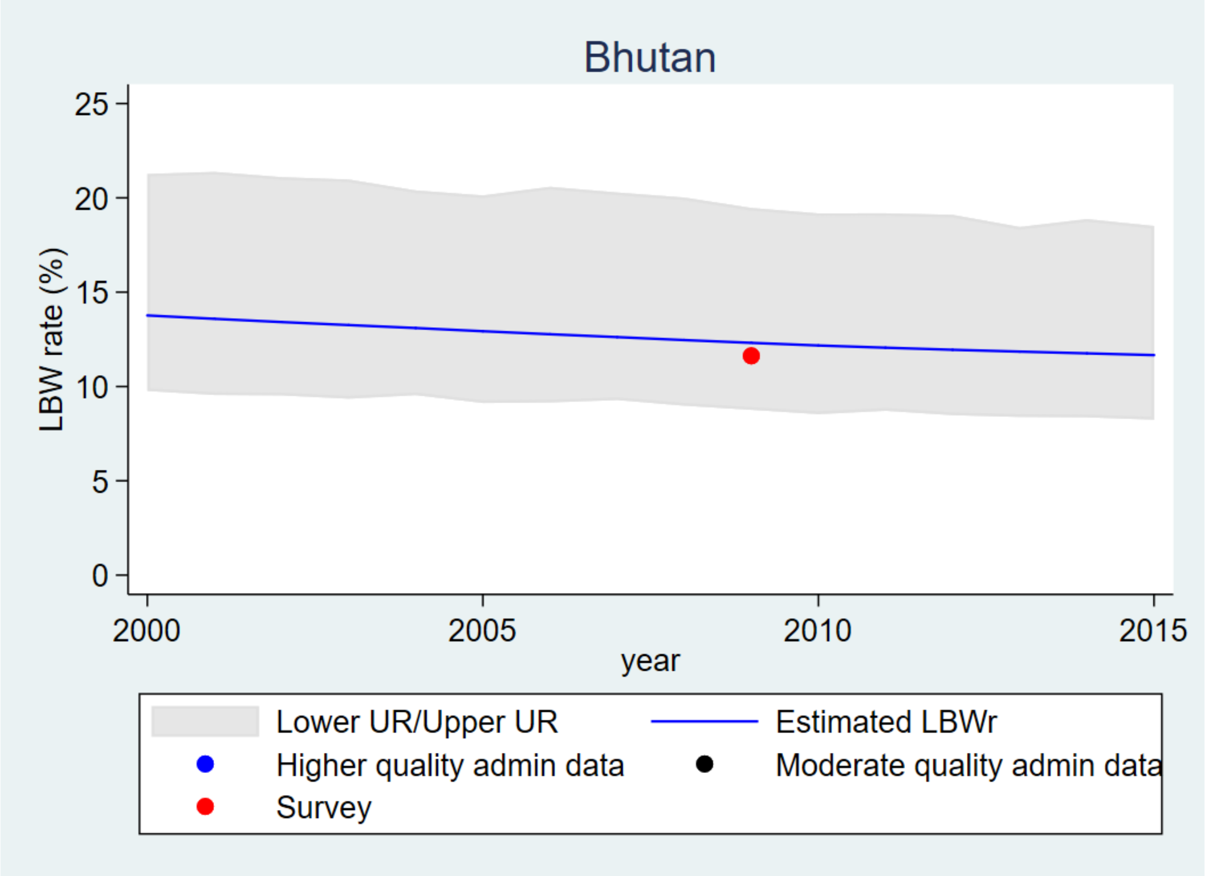
Bhutan
LBW rate (%)
25
20
15
10
5
0
2000
2005
2010
2015
year
Lower UR/Upper UR
Estimated LBWr
Higher quality admin data
Moderate quality admin data
Survey

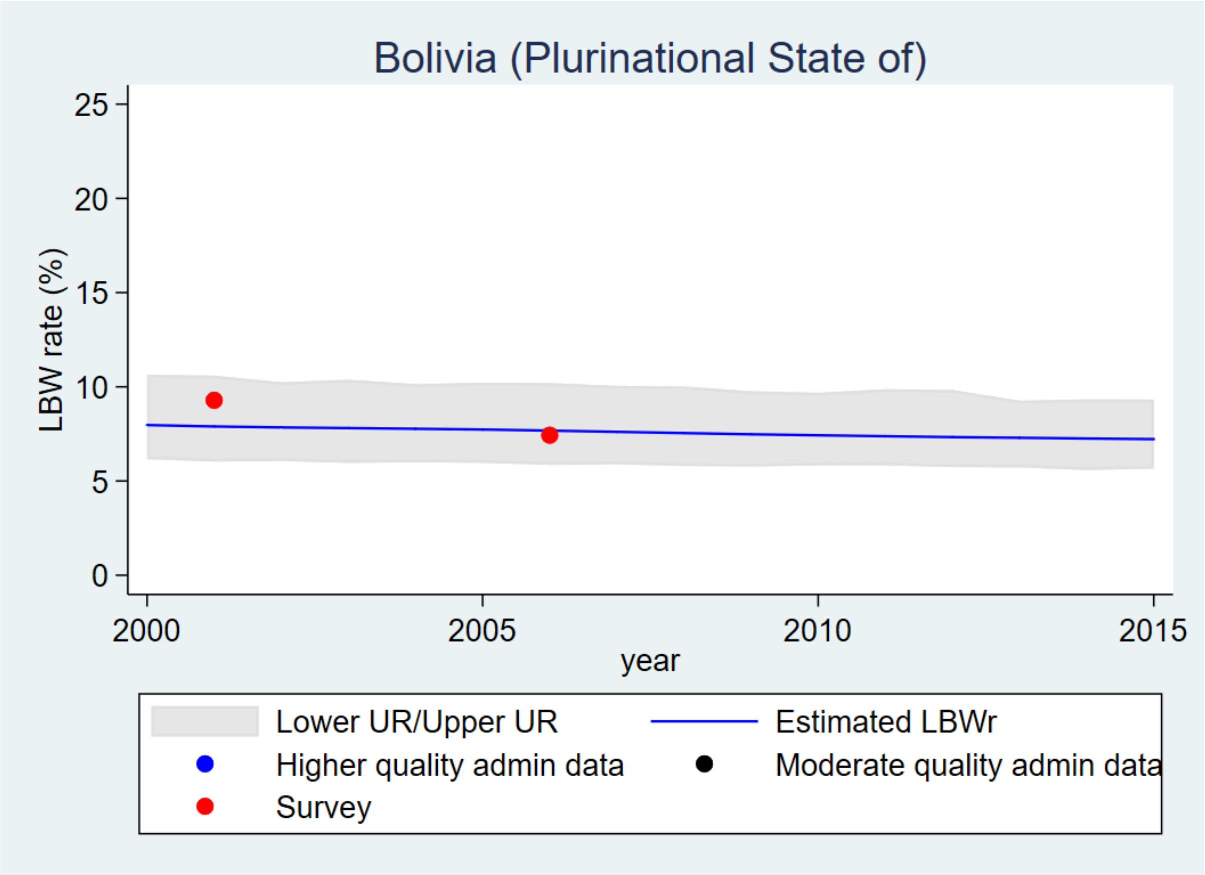
Bolivia (Plurinational State of)
LBW rate (%)
0
5
10
15
20
25
2000
2005
2010
2015
year
Lower UR/Upper UR
Estimated LBWr
Higher quality admin data
Moderate quality admin data
Survey

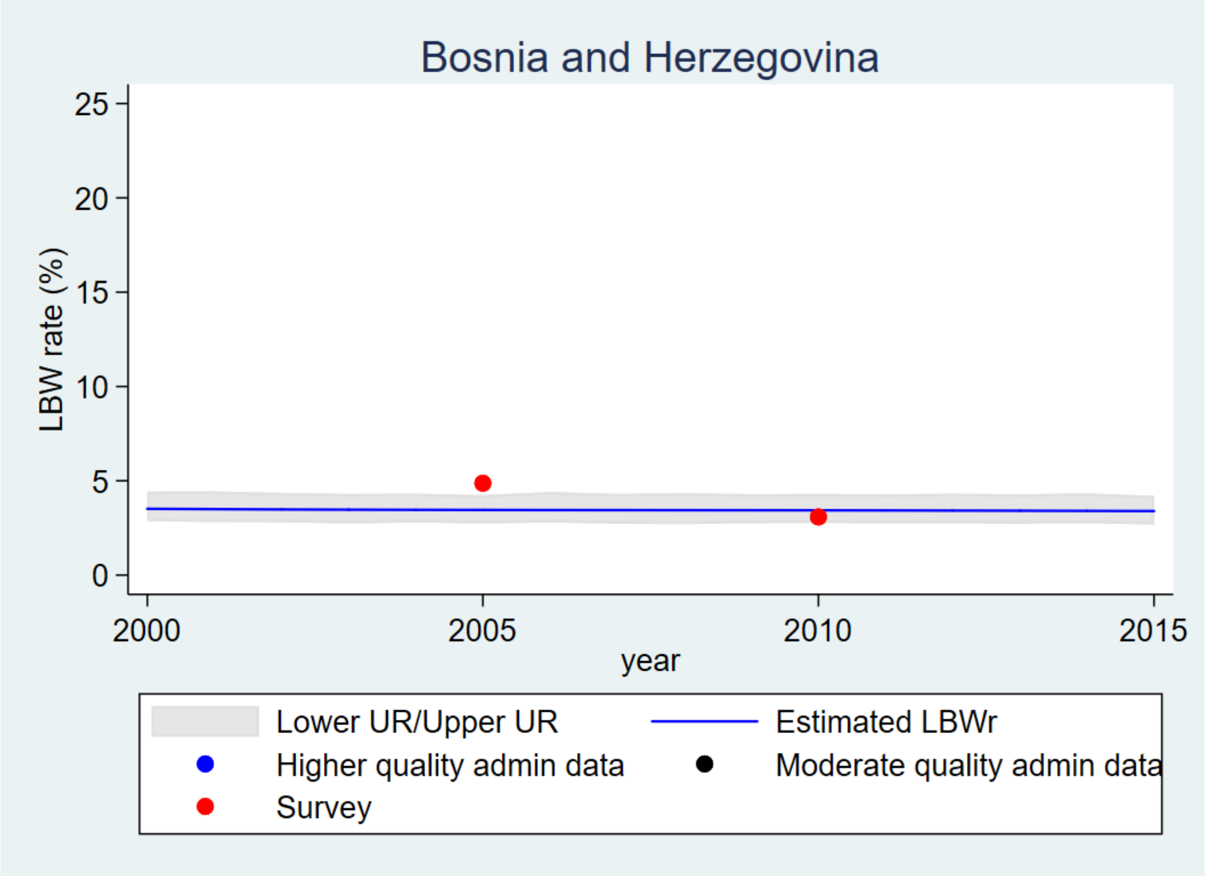
Bosnia and Herzegovina
LBW rate (%)
0
5
10
15
20
25
2000
2005
2010
2015
year
Lower UR/Upper UR
Estimated LBWr
Higher quality admin data
Moderate quality admin data
Survey

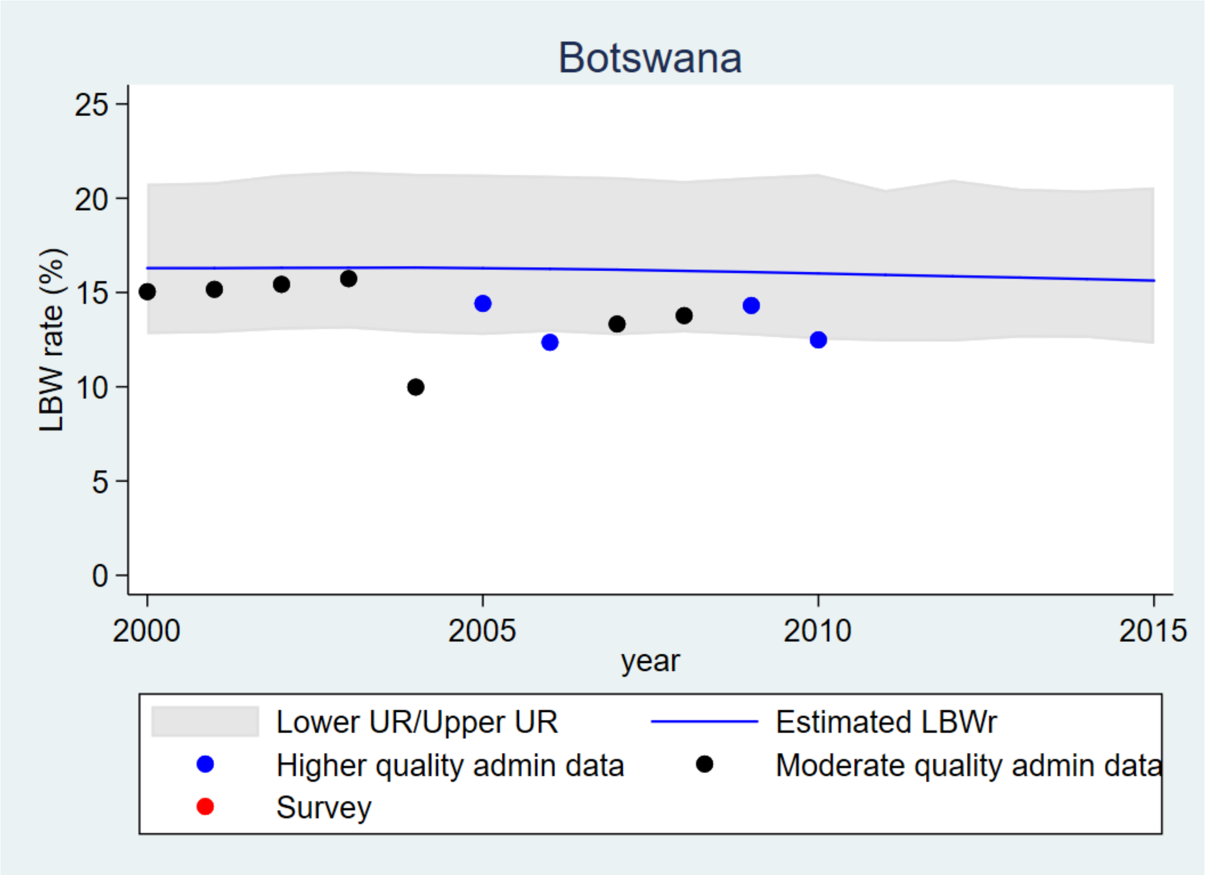

Botswana
LBW rate (%)
25
20
15
10
5
0
2000
2005
2010
2015
year
Lower UR/Upper UR
Estimated LBWr
Higher quality admin data
Moderate quality admin data
Survey

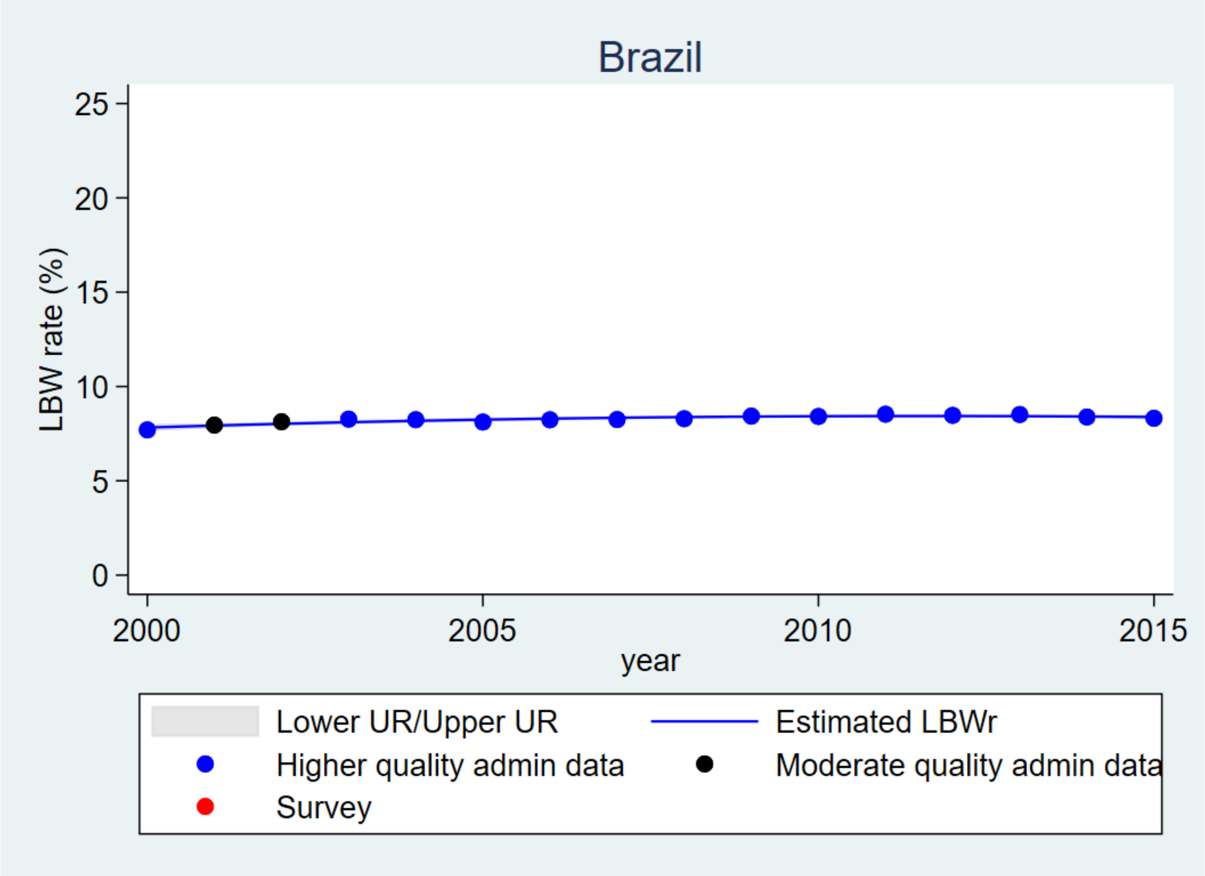

Brazil
LBW rate (%)
25
20
15
10
5
0
2000
2005
2010
2015
year
Lower UR/Upper UR
Estimated LBWr
Higher quality admin data
Moderate quality admin data
Survey

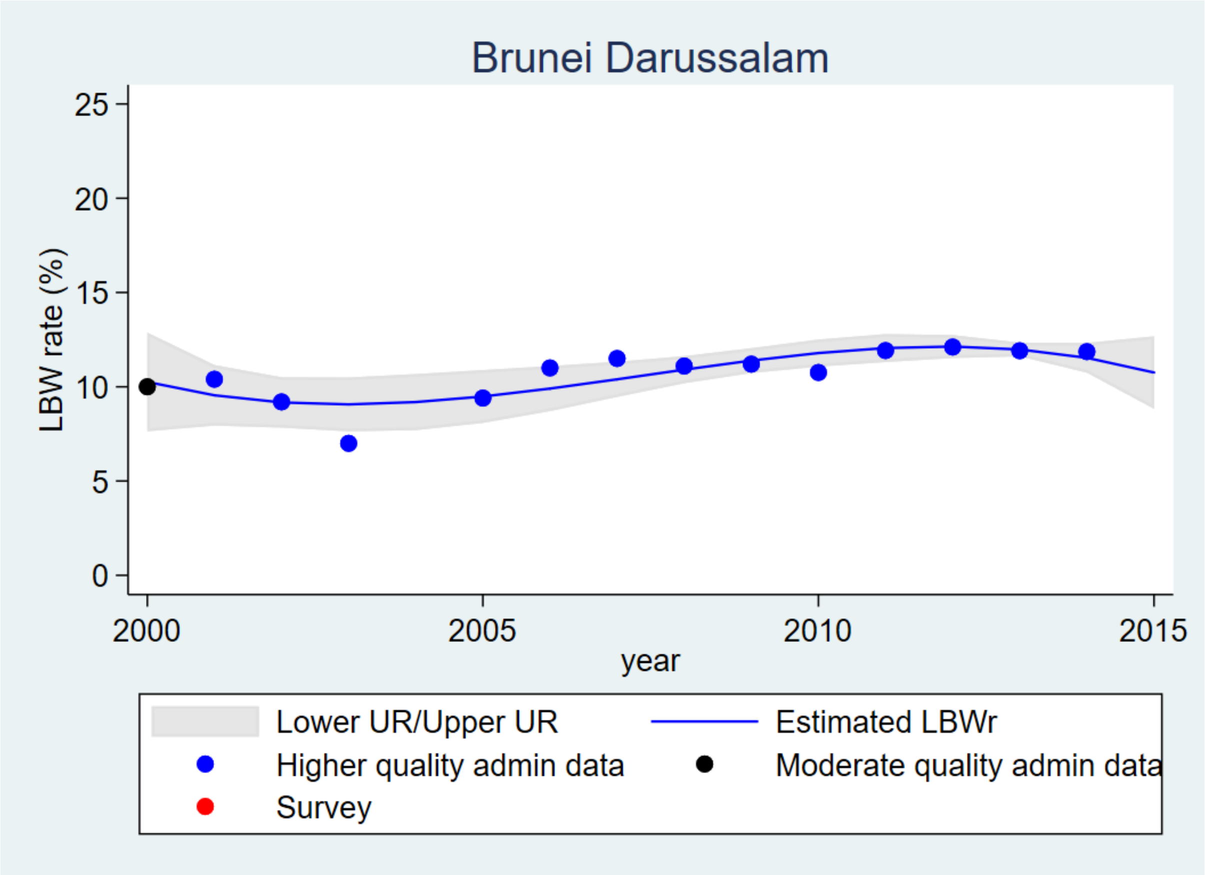
Brunei Darussalam
LBW rate (%)
25
20
15
10
5
0
2000
2005
2010
2015
year
Lower UR/Upper UR
Estimated LBWr
Higher quality admin data
Moderate quality admin data
Survey

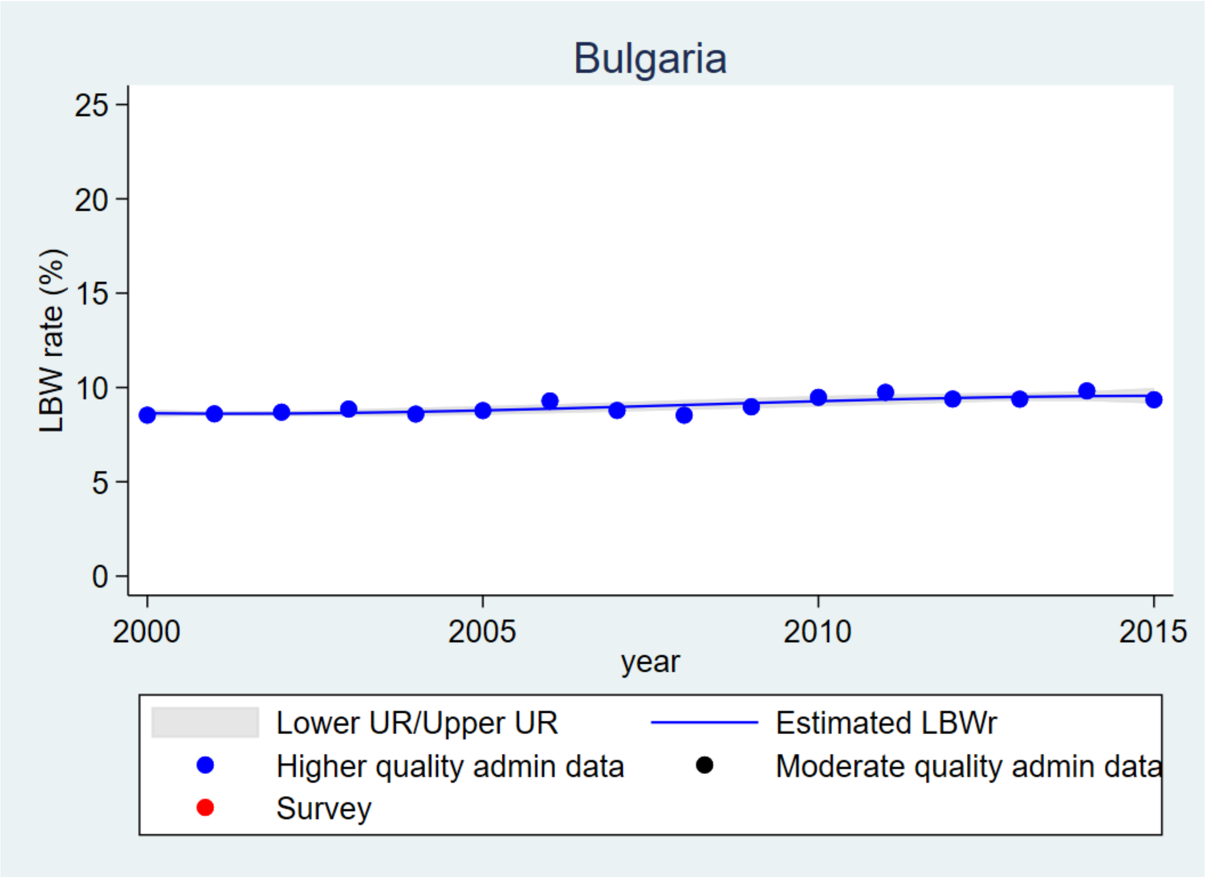
Bulgaria
LBW rate (%)
25
20
15
10
5
0
2000
2005
2010
2015
year
Lower UR/Upper UR
Estimated LBWr
Higher quality admin data
Moderate quality admin data
Survey

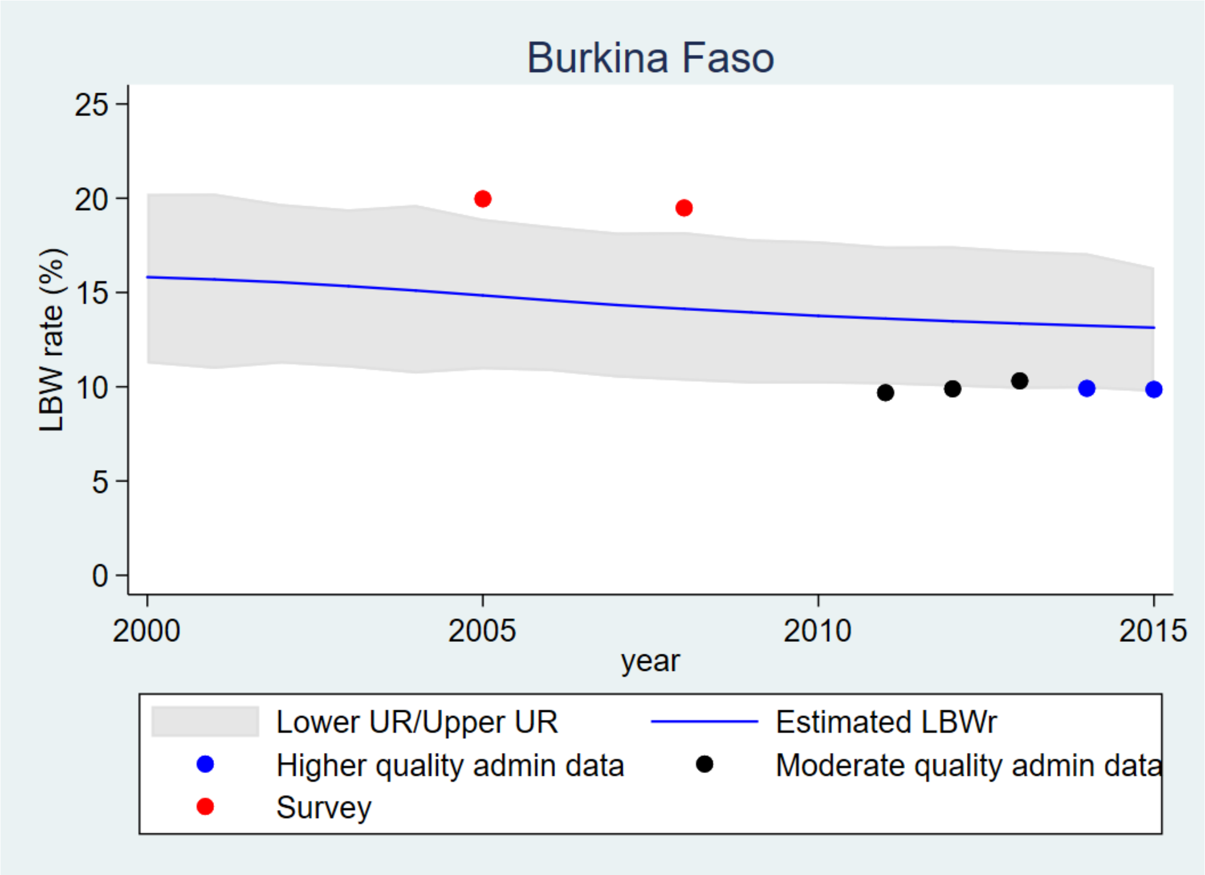

Burkina Faso
LBW rate (%)
25
20
15
10
5
0
2000
2005
2010
2015
year
Lower UR/Upper UR
Estimated LBWr
Higher quality admin data
Moderate quality admin data
Survey

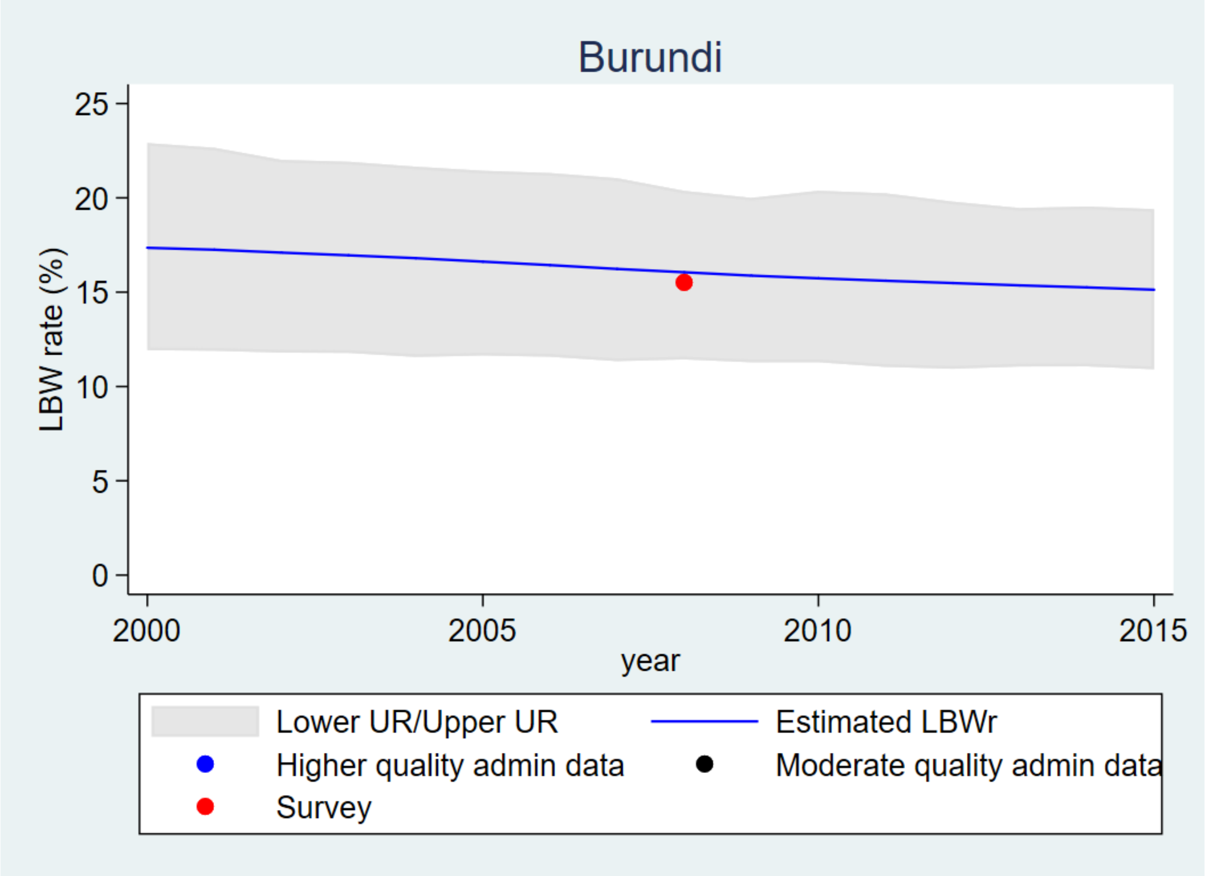

Burundi
LBW rate (%)
25
20
15
10
5
0
2000
2005
2010
2015
year
Lower UR/Upper UR
Estimated LBWr
Higher quality admin data
Moderate quality admin data
Survey

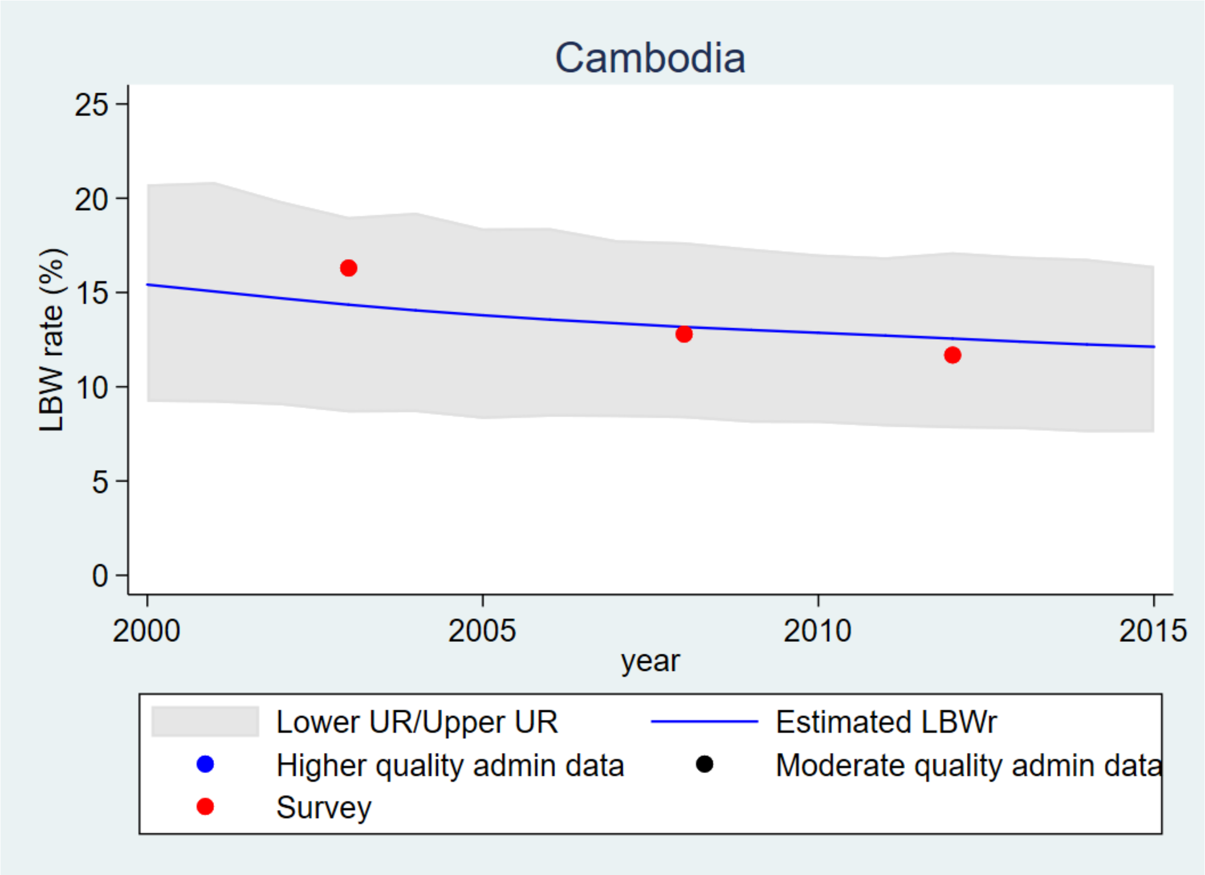
Cambodia
LBW rate (%)
25
20
15
10
5
0
2000
2005
2010
2015
year
Lower UR/Upper UR
Estimated LBWr
Higher quality admin data
Moderate quality admin data
Survey

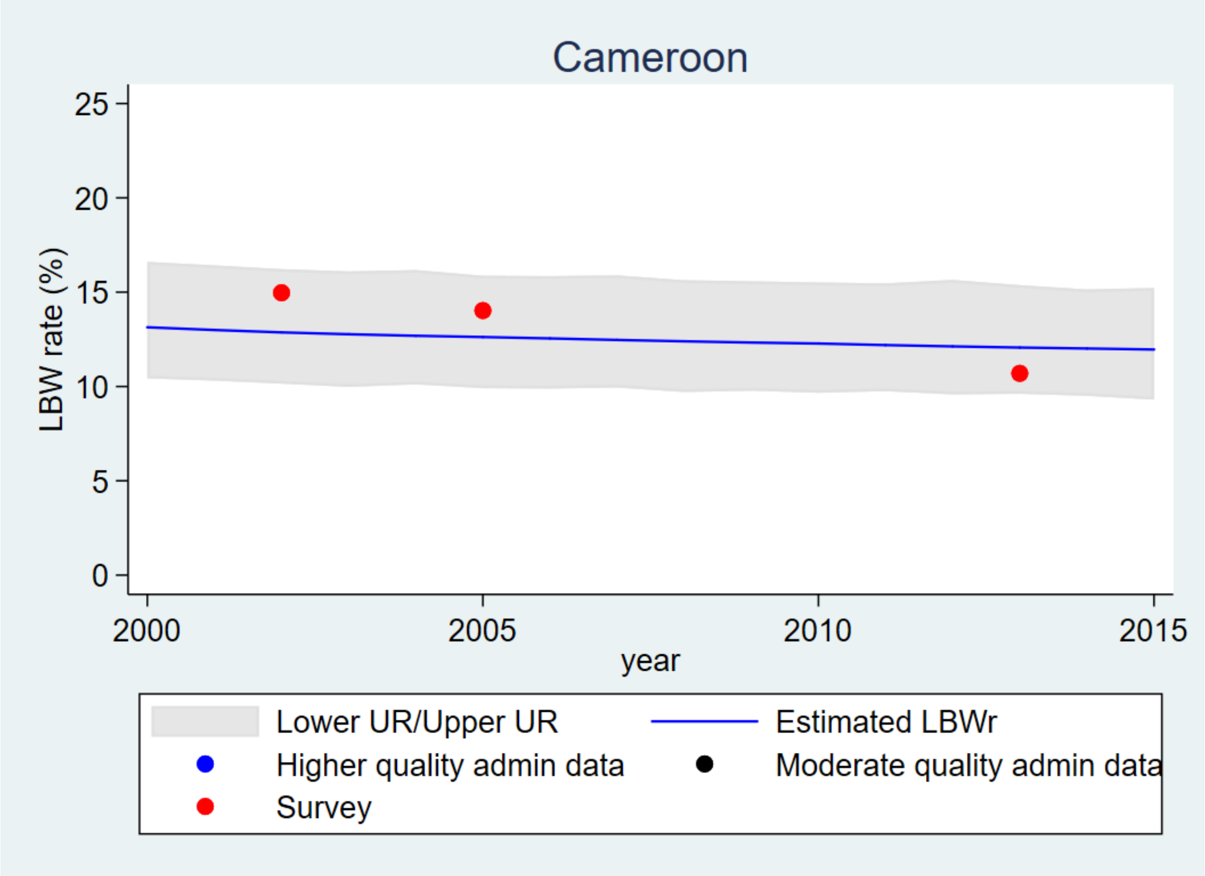
Cameroon
LBW rate (%)
25
20
15
10
5
0
2000
2005
2010
2015
year
Lower UR/Upper UR
Estimated LBWr
Higher quality admin data
Moderate quality admin data
Survey

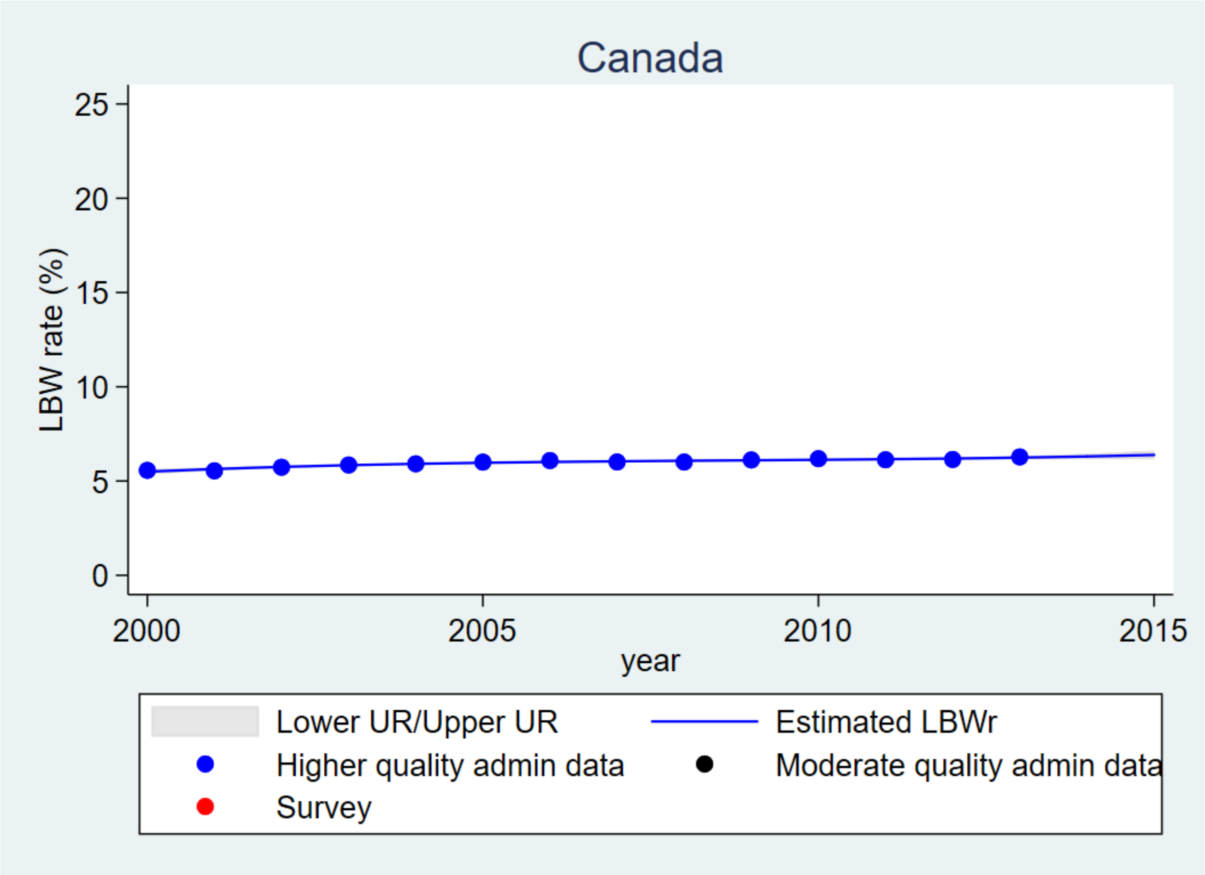
Canada
LBW rate (%)
25
20
15
10
5
0
2000
2005
2010
2015
year
Lower UR/Upper UR
Estimated LBWr
Higher quality admin data
Moderate quality admin data
Survey

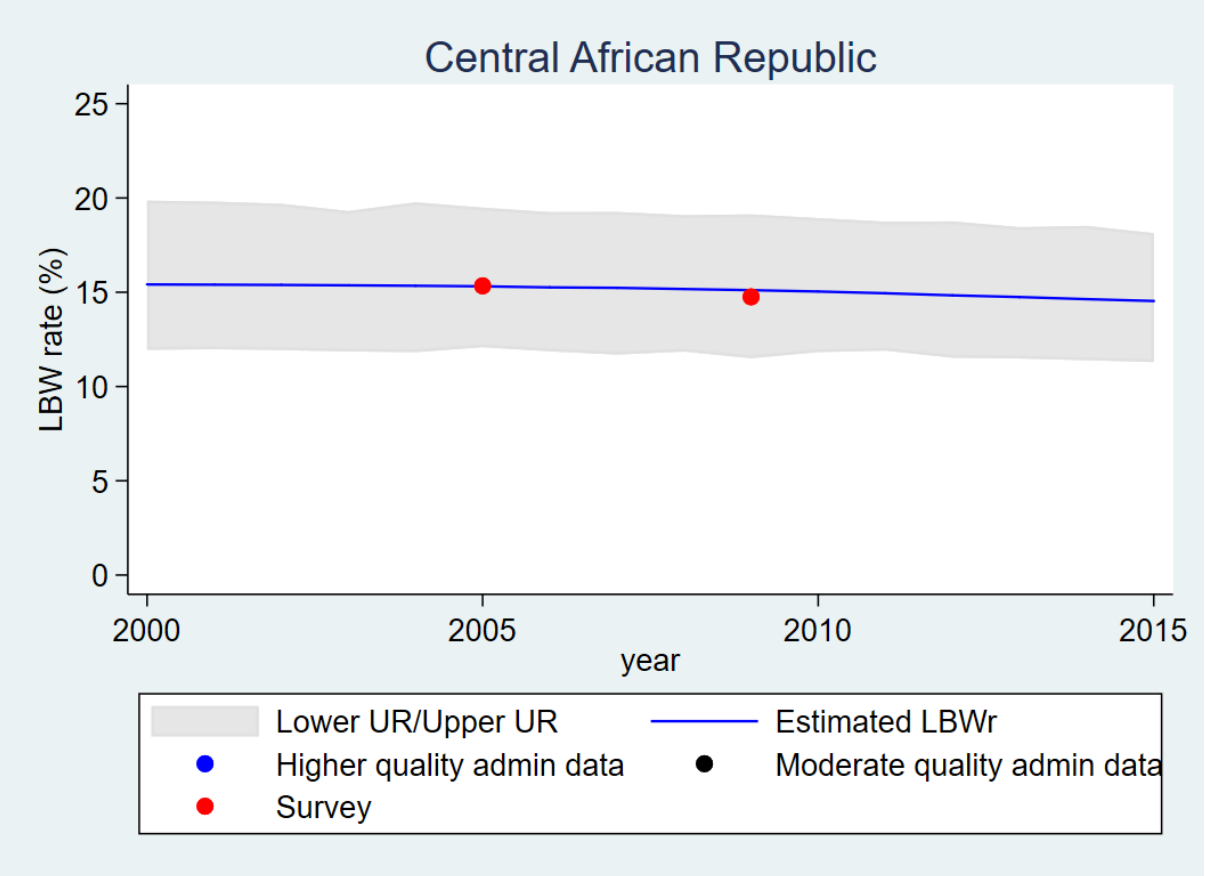
Central African Republic
LBW rate (%)
25
20
15
10
5
0
2000
2005
2010
2015
year
Lower UR/Upper UR
Estimated LBWr
Higher quality admin data
Moderate quality admin data
Survey

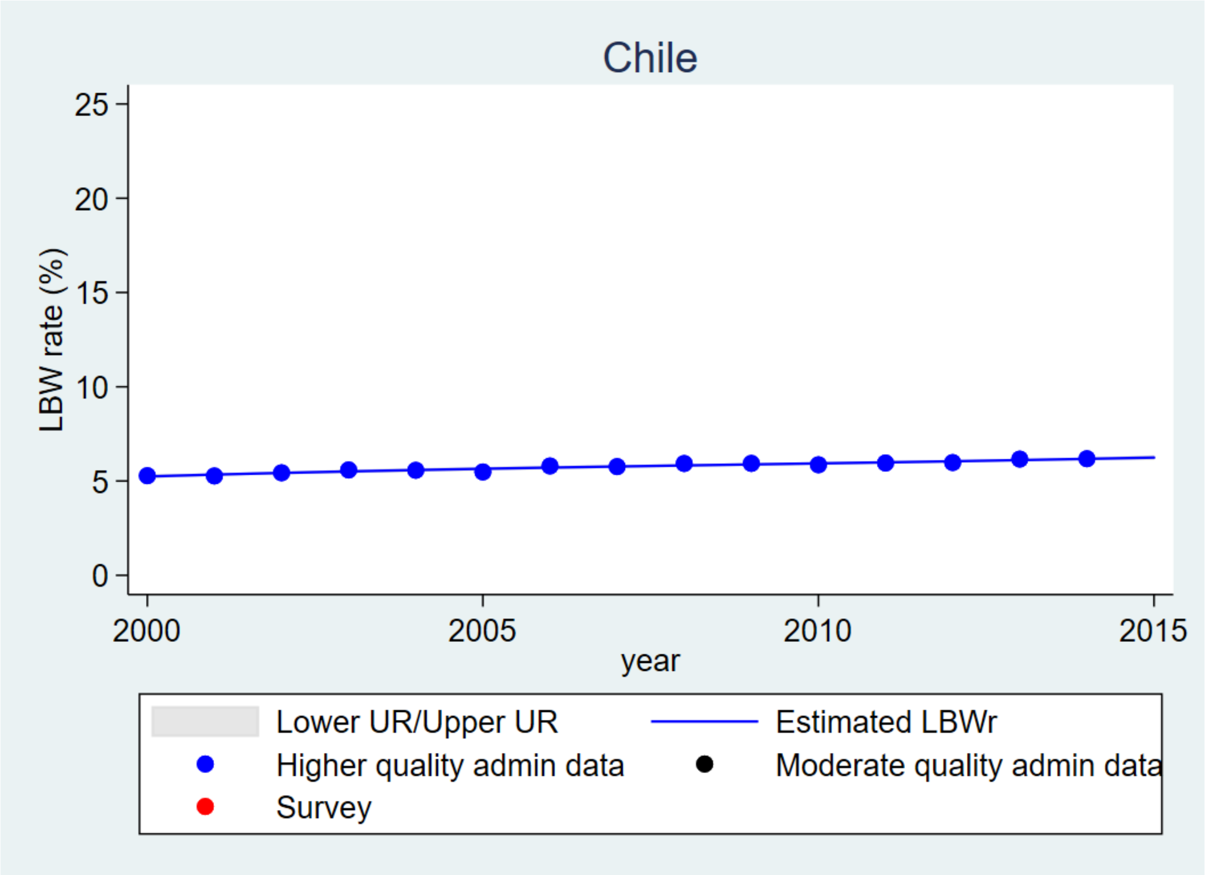

Chile
LBW rate (%)
25
20
15
10
5
0
2000
2005
2010
2015
year
Lower UR/Upper UR
Estimated LBWr
Higher quality admin data
Moderate quality admin data
Survey

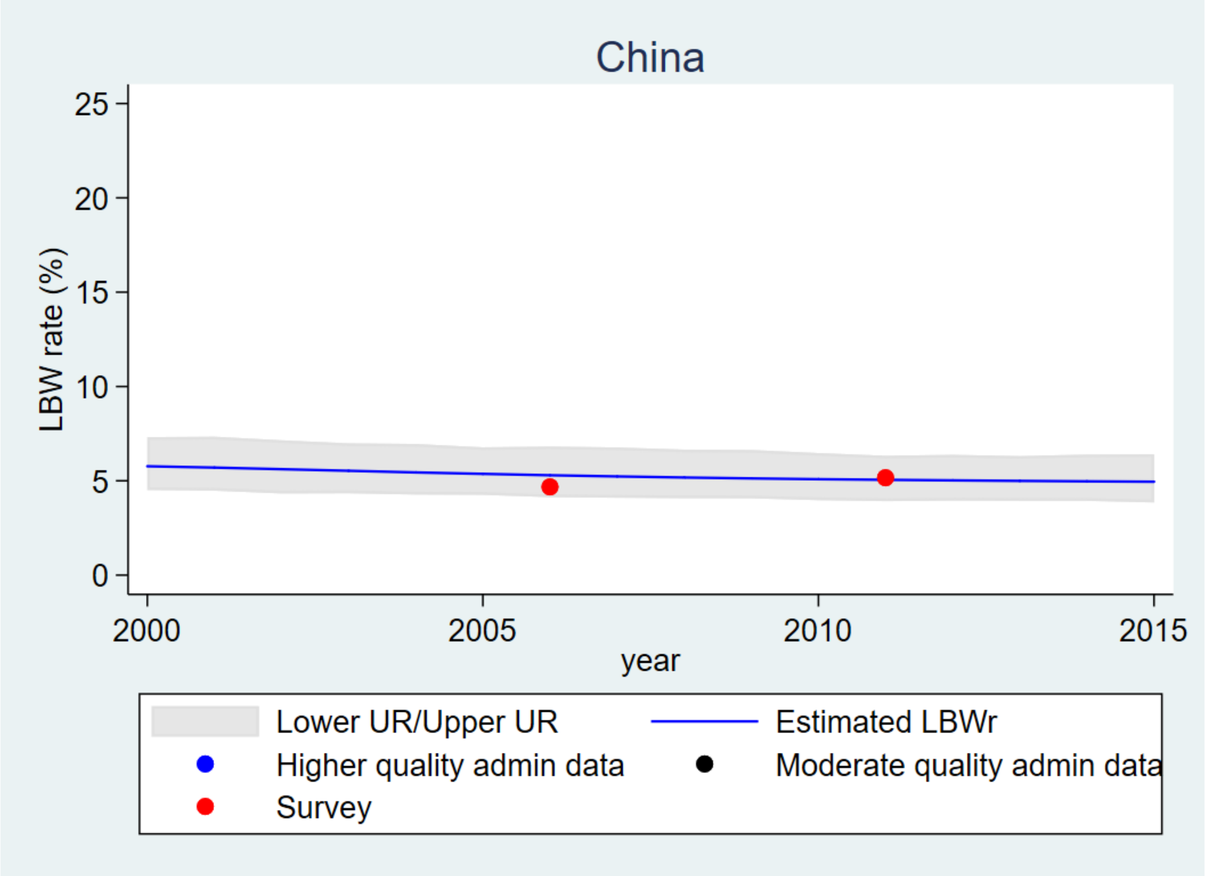

China
LBW rate (%)
25
20
15
10
5
0
2000
2005
2010
2015
year
Lower UR/Upper UR
Estimated LBWr
Higher quality admin data
Moderate quality admin data
Survey

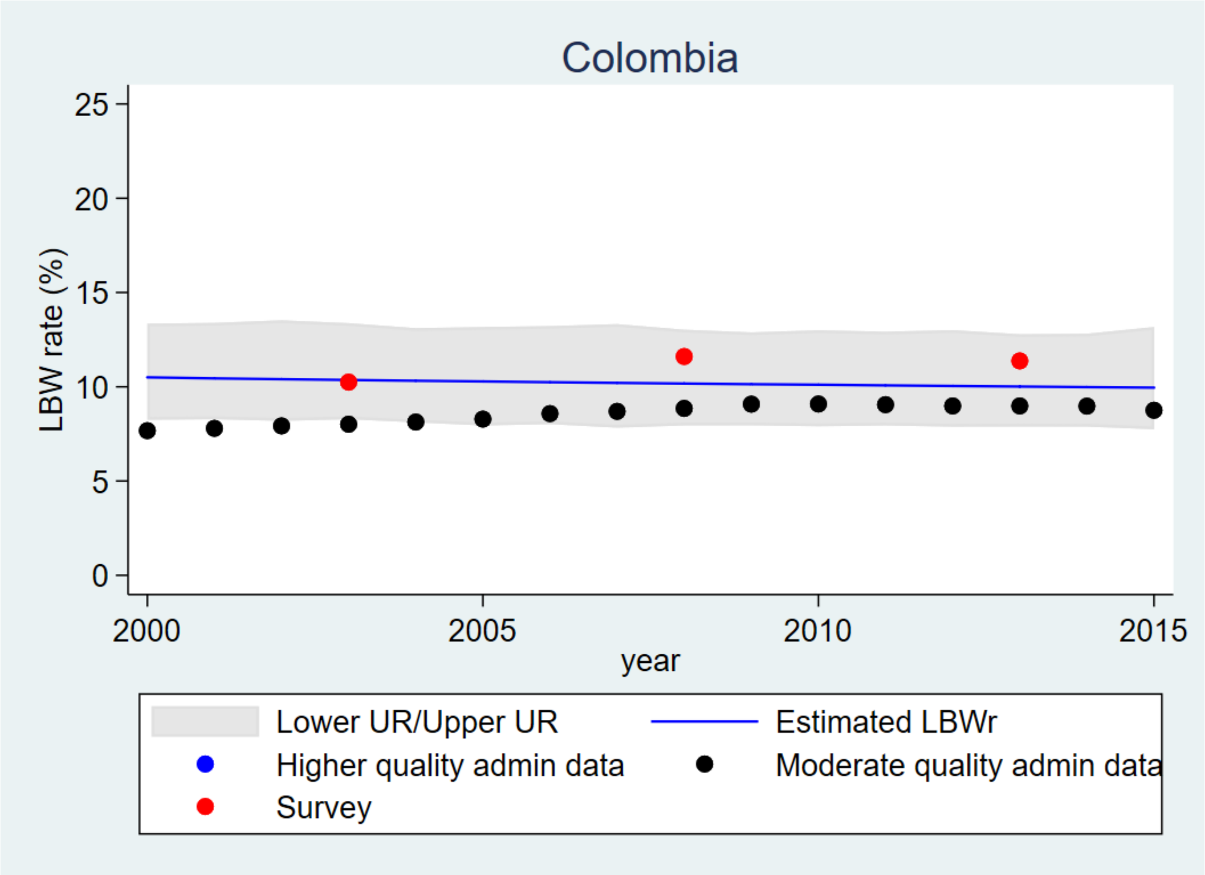
Colombia
LBW rate (%)
25
20
15
10
5
0
2000
2005
2010
2015
year
Lower UR/Upper UR
Estimated LBWr
Higher quality admin data
Moderate quality admin data
Survey

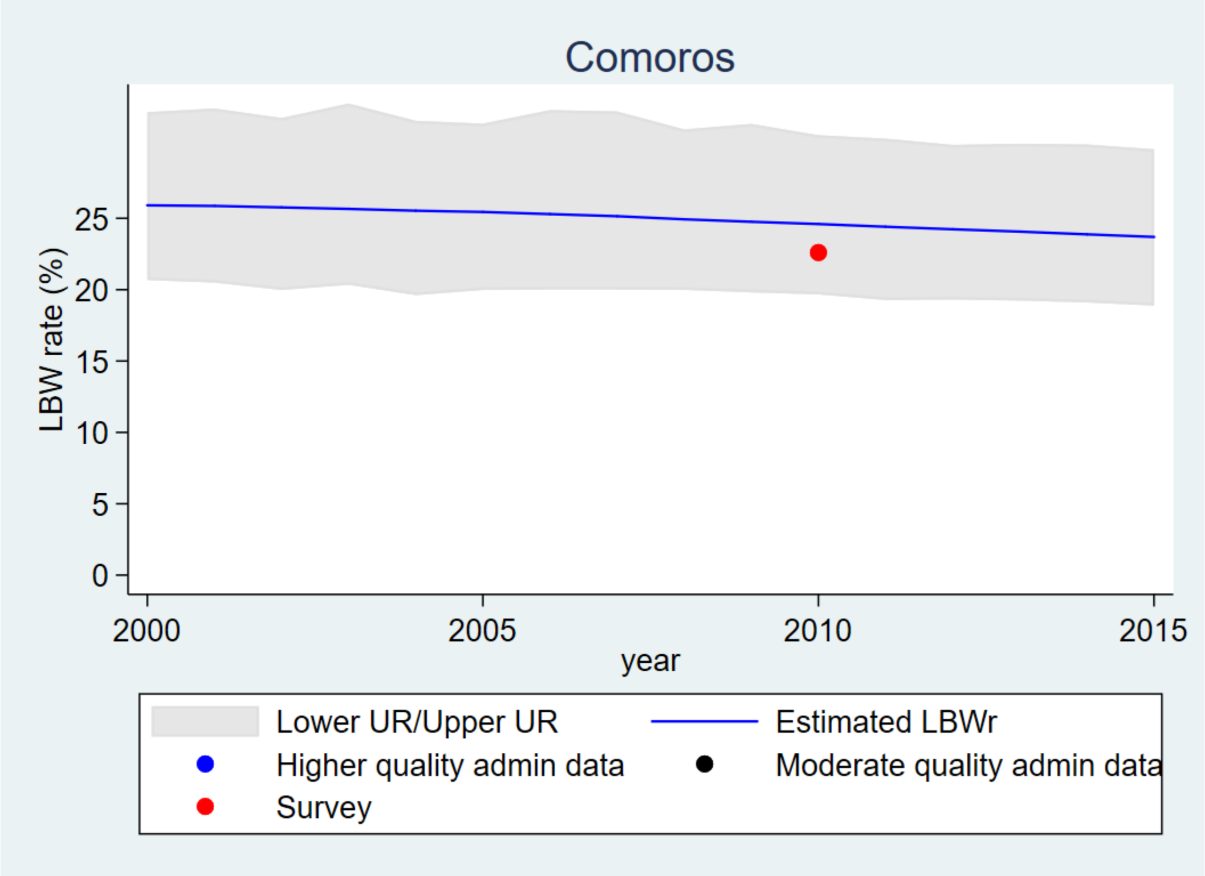
Comoros
LBW rate (%)
25
20
15
10
5
0
2000
2005
2010
2015
year
Lower UR/Upper UR
Estimated LBWr
Higher quality admin data
Moderate quality admin data
Survey

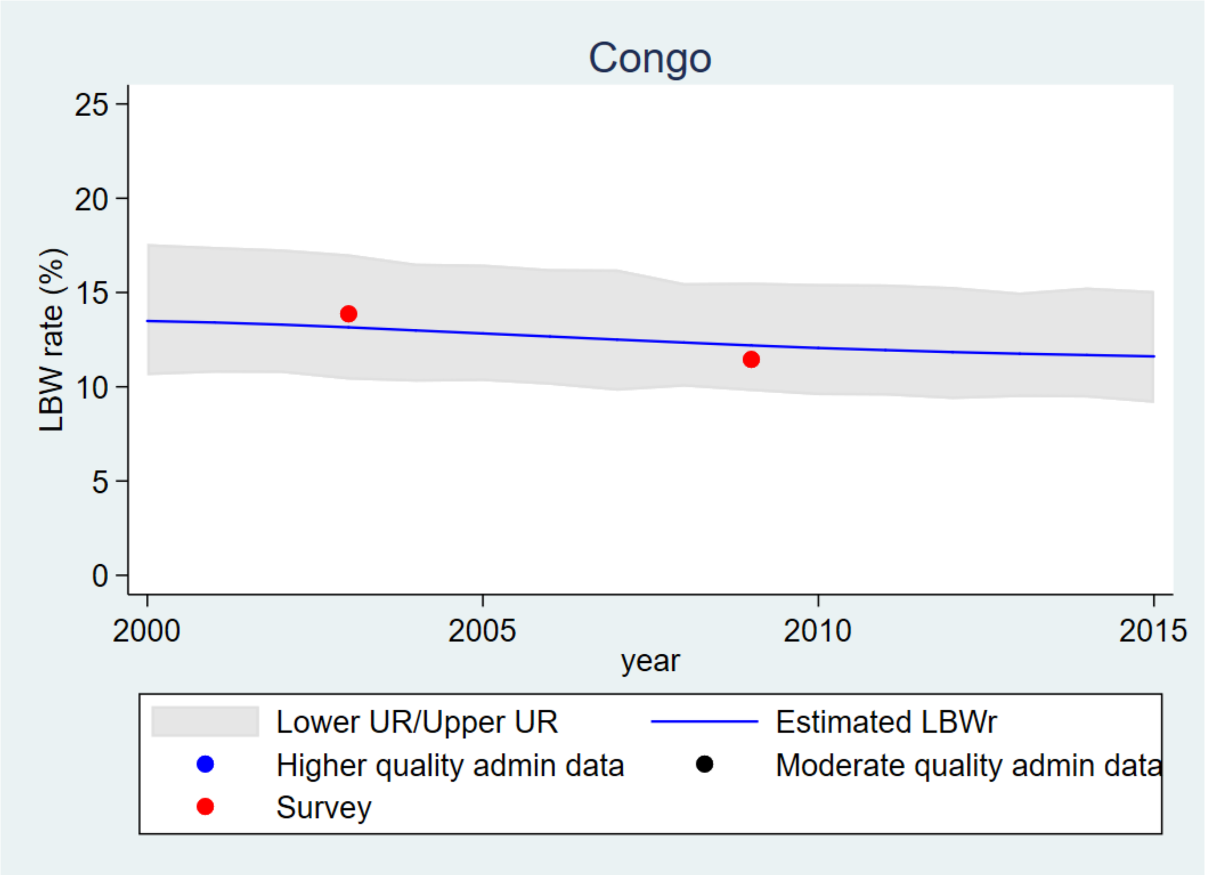
Congo
LBW rate (%)
25
20
15
10
5
0
2000
2005
2010
2015
year
Lower UR/Upper UR
Estimated LBWr
Higher quality admin data
Moderate quality admin data
Survey

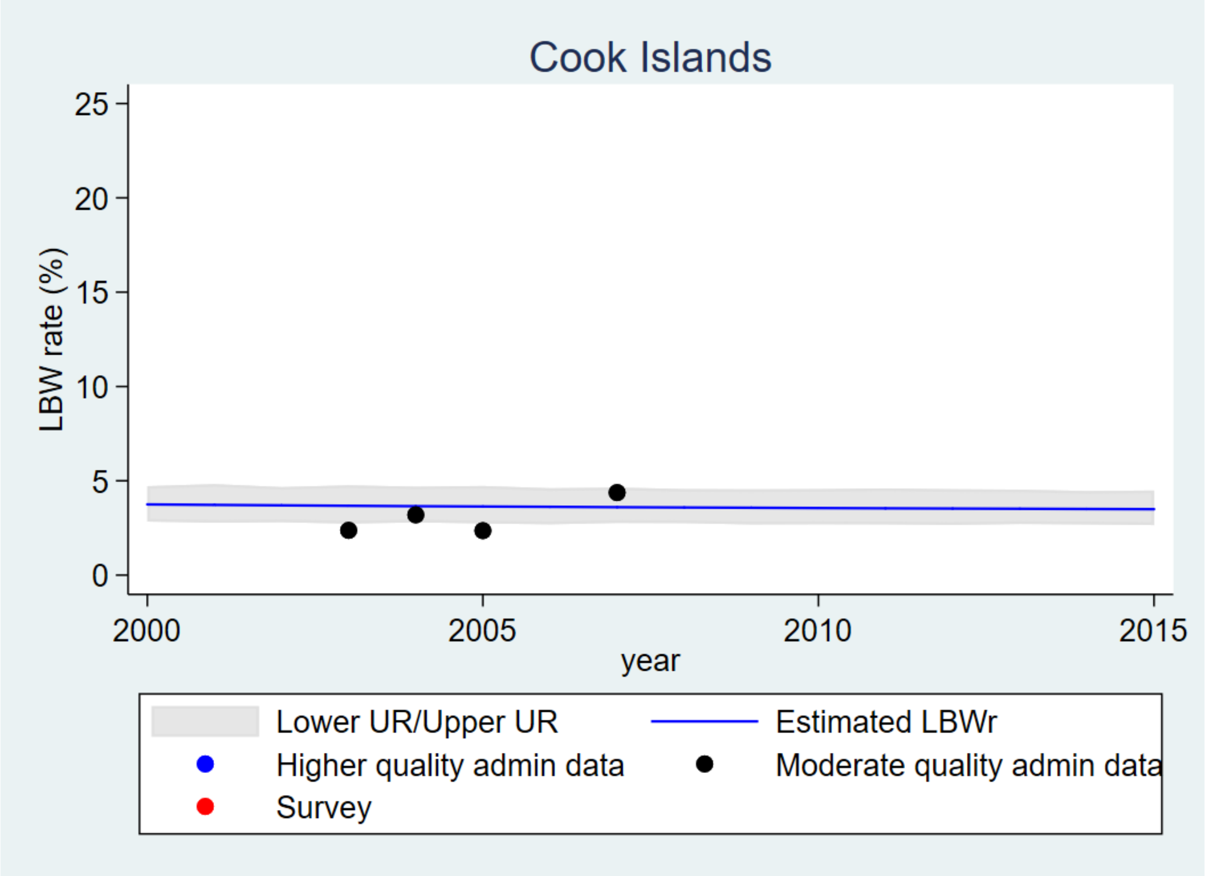
Cook Islands
LBW rate (%)
25
20
15
10
5
0
2000
2005
2010
2015
year
Lower UR/Upper UR
Estimated LBWr
Higher quality admin data
Moderate quality admin data
Survey

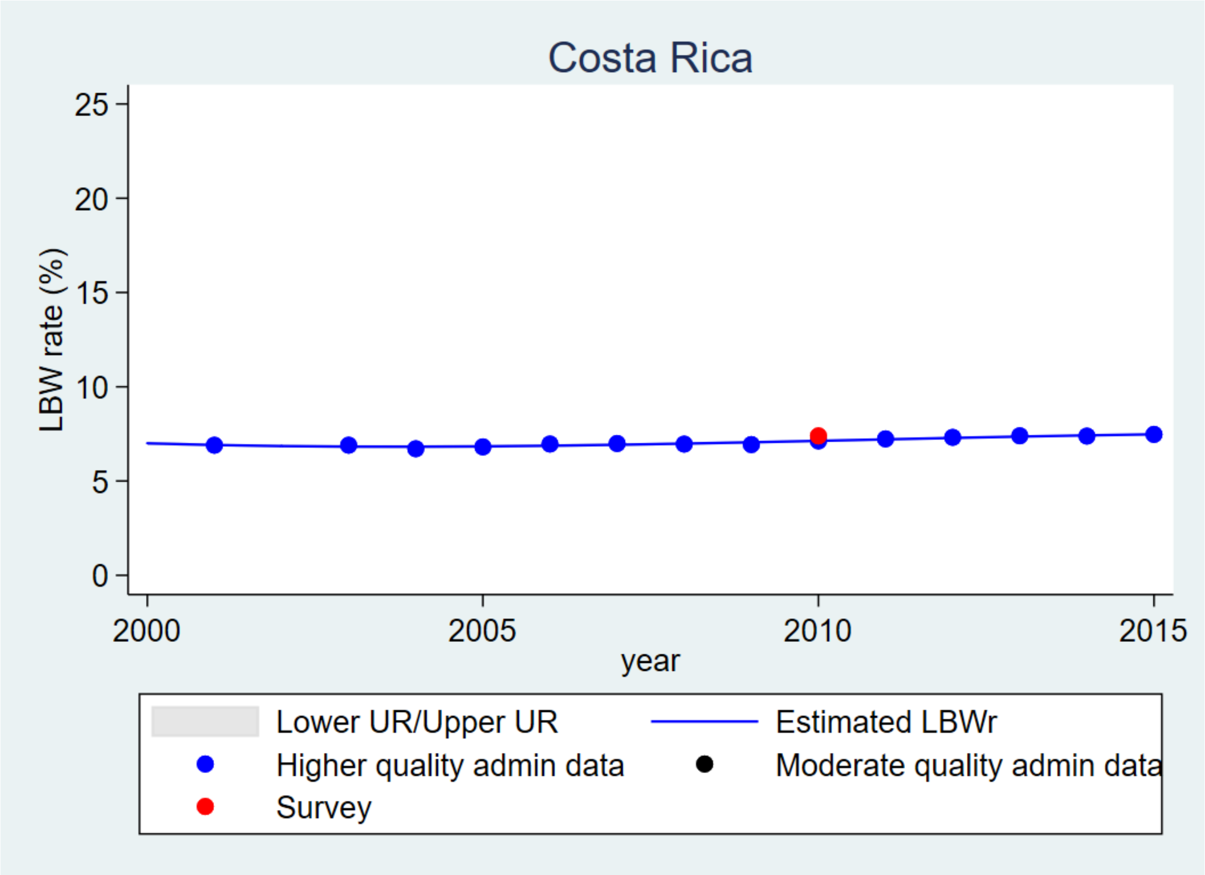
Costa Rica
LBW rate (%)
25
20
15
10
5
0
2000
2005
2010
2015
year
Lower UR/Upper UR
Estimated LBWr
Higher quality admin data
Moderate quality admin data
Survey

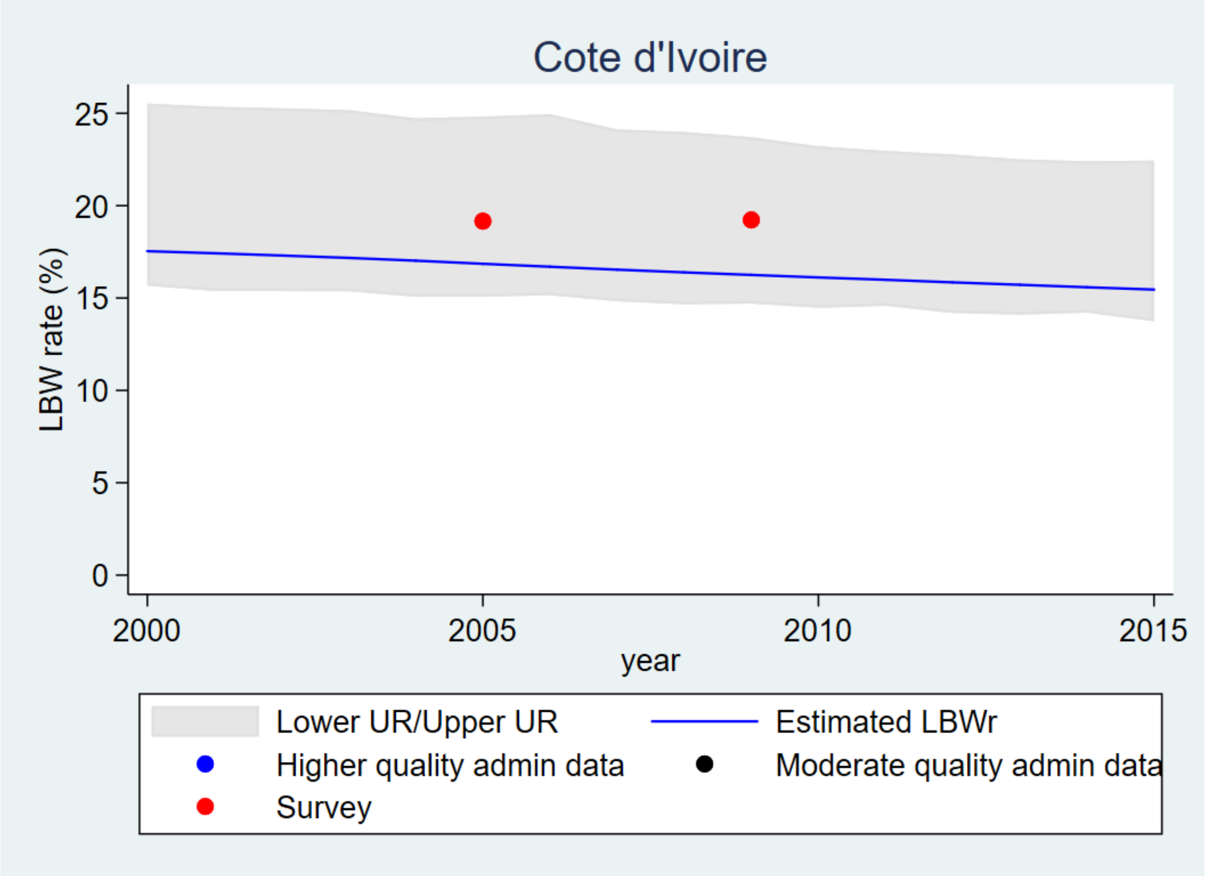
Cote d'Ivoire
LBW rate (%)
25
20
15
10
5
0
2000
2005
2010
2015
year
Lower UR/Upper UR
Estimated LBWr
Higher quality admin data
Moderate quality admin data
Survey

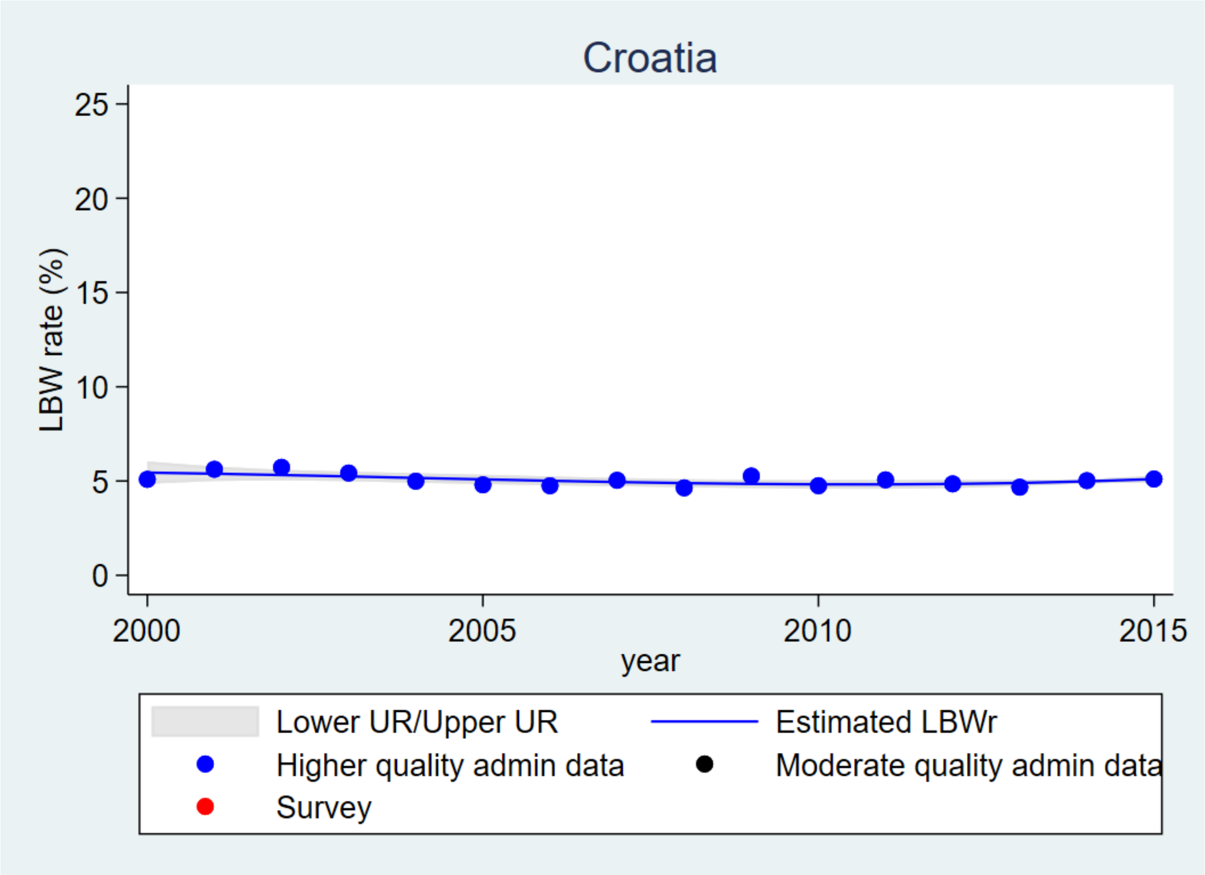
Croatia
LBW rate (%)
25
20
15
10
5
0
2000
2005
2010
2015
year
Lower UR/Upper UR
Estimated LBWr
Higher quality admin data
Moderate quality admin data
Survey

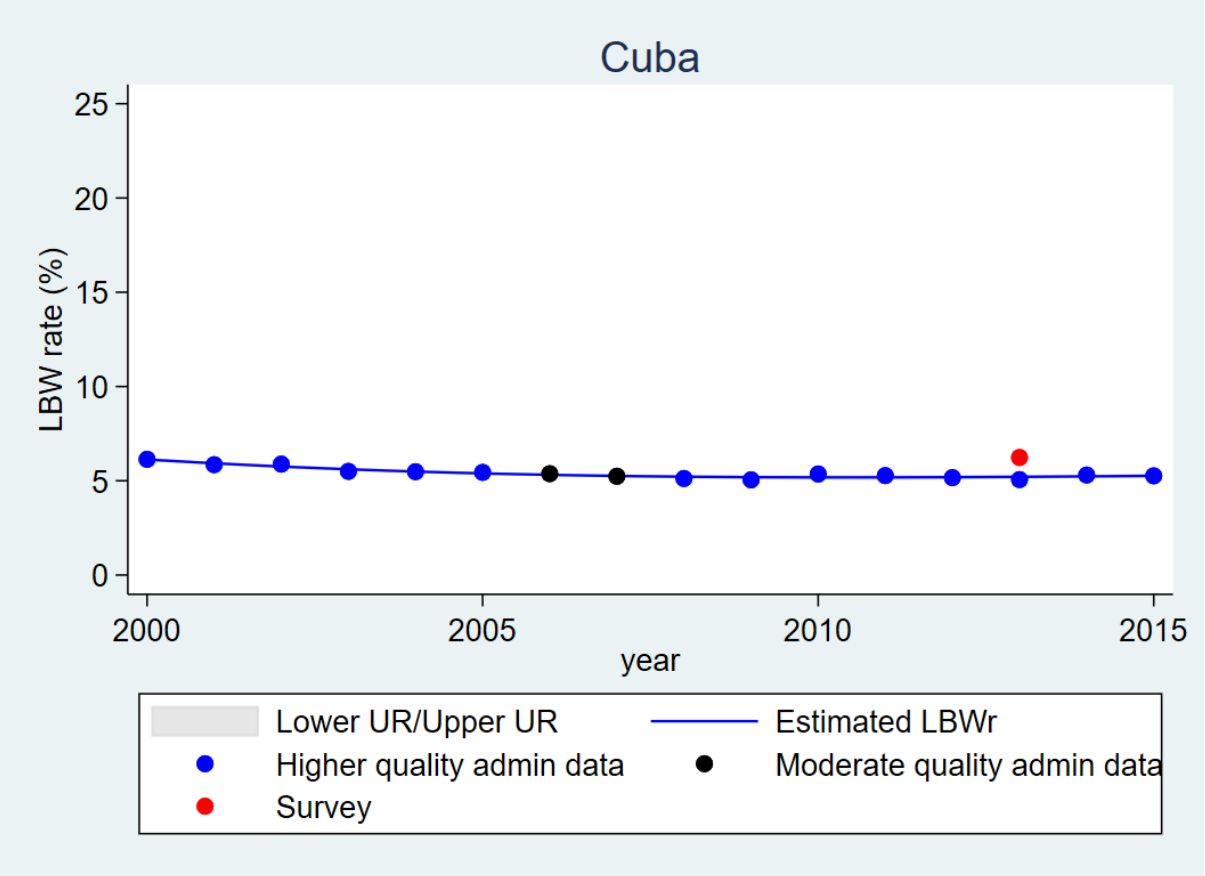
Cuba
LBW rate (%)
25
20
15
10
5
0
2000
2005
2010
2015
year
Lower UR/Upper UR
Estimated LBWr
Higher quality admin data
Moderate quality admin data
Survey

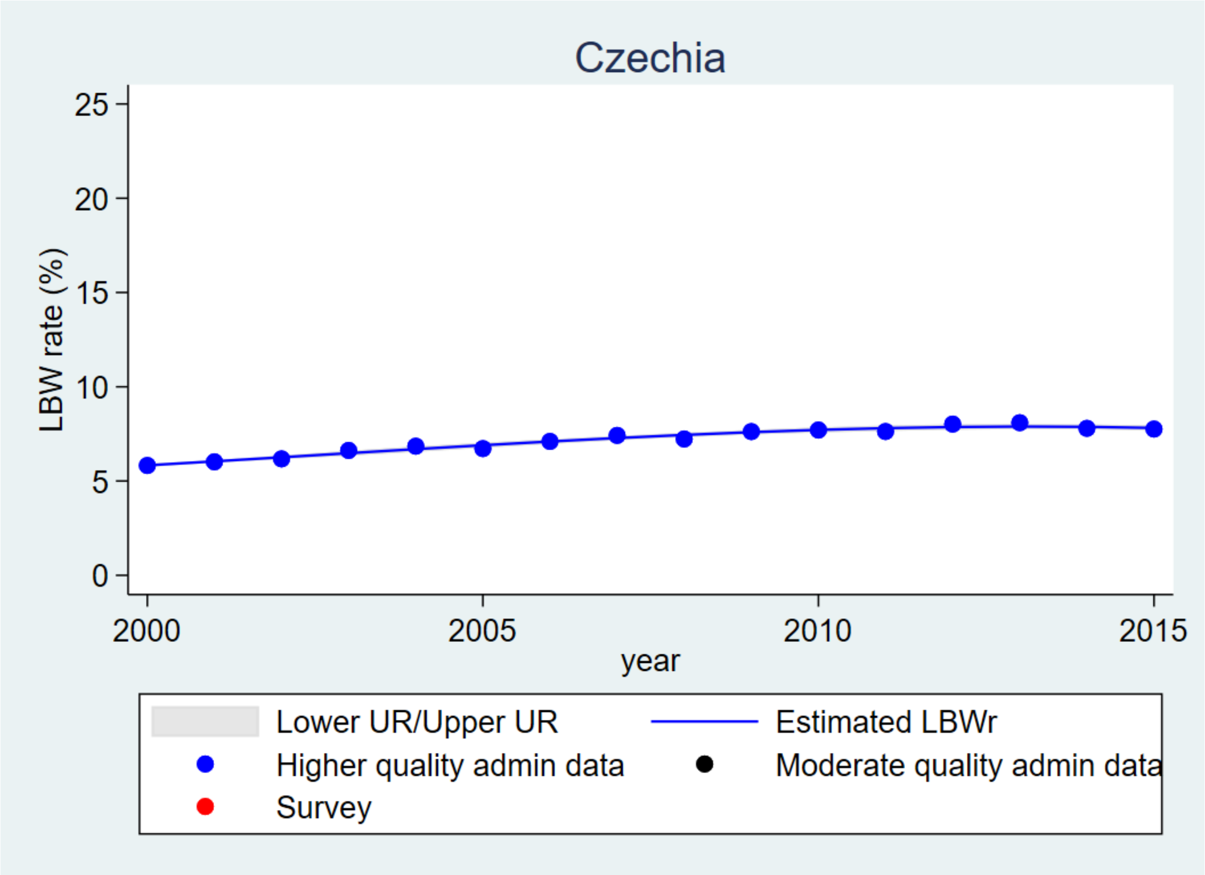

Czechia
LBW rate (%)
0
5
10
15
20
25
2000
2005
2010
2015
year
Lower UR/Upper UR
Estimated LBWr
Higher quality admin data
Moderate quality admin data
Survey

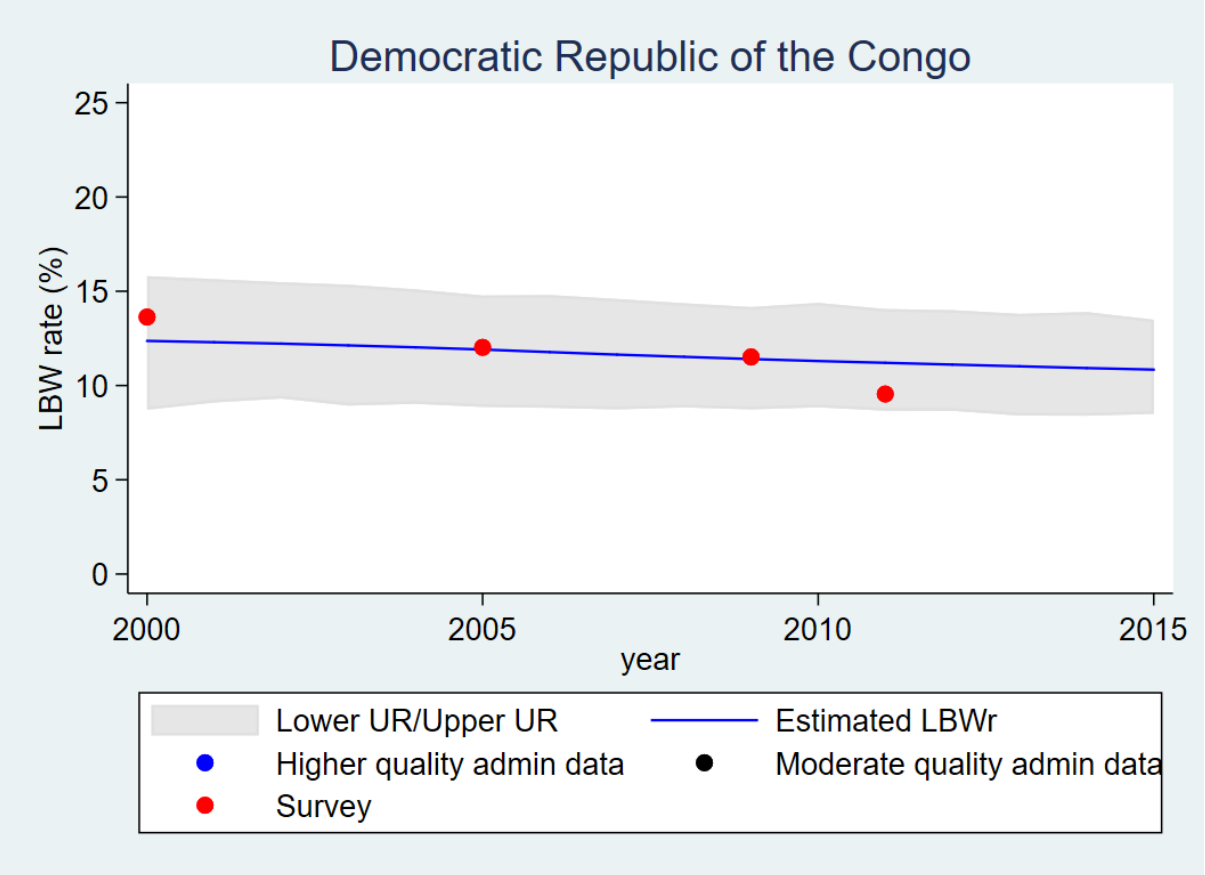

Democratic Republic of the Congo
LBW rate (%)
0
5
10
15
20
25
2000
2005
2010
2015
year
Lower UR/Upper UR
Estimated LBWr
Higher quality admin data
Moderate quality admin data
Survey

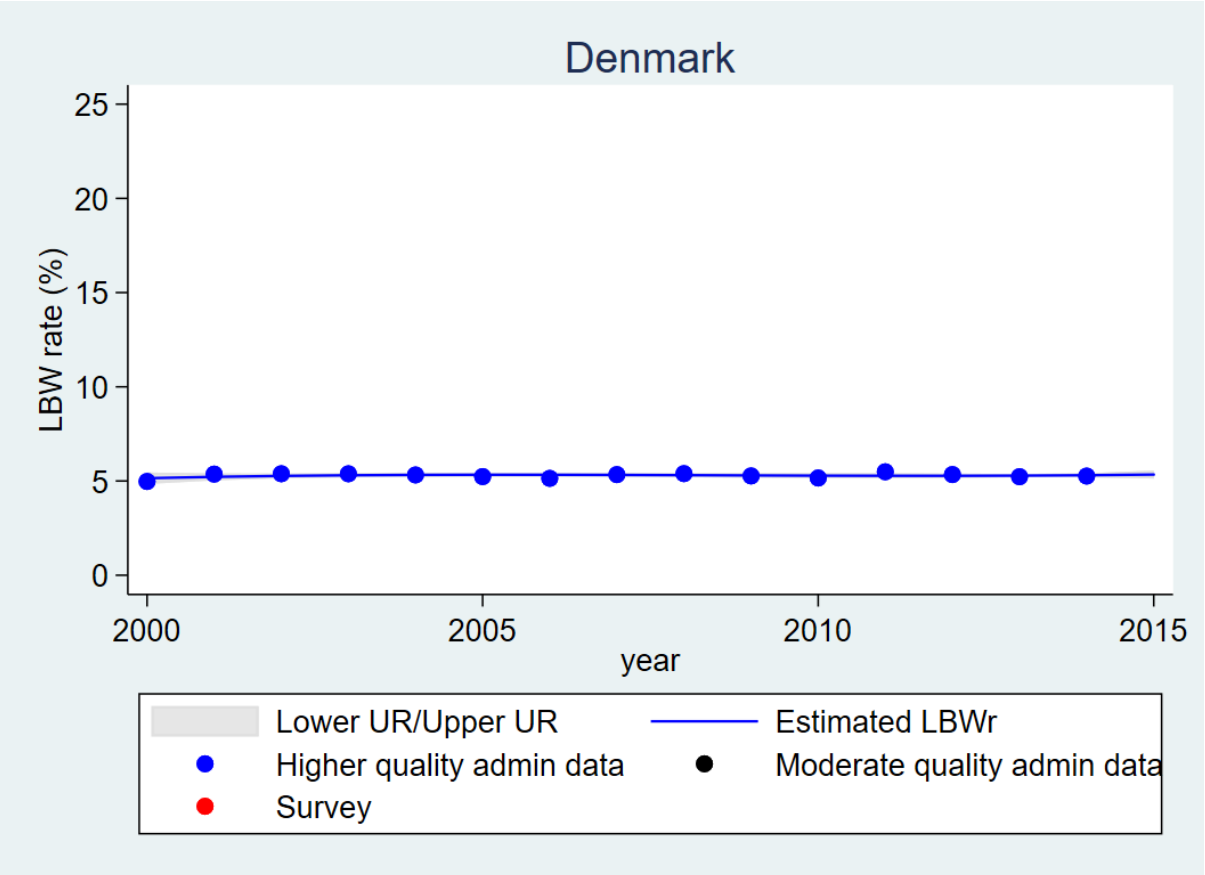
Denmark
LBW rate (%)
25
20
15
10
5
0
2000
2005
2010
2015
year
Lower UR/Upper UR
Estimated LBWr
Higher quality admin data
Moderate quality admin data
Survey

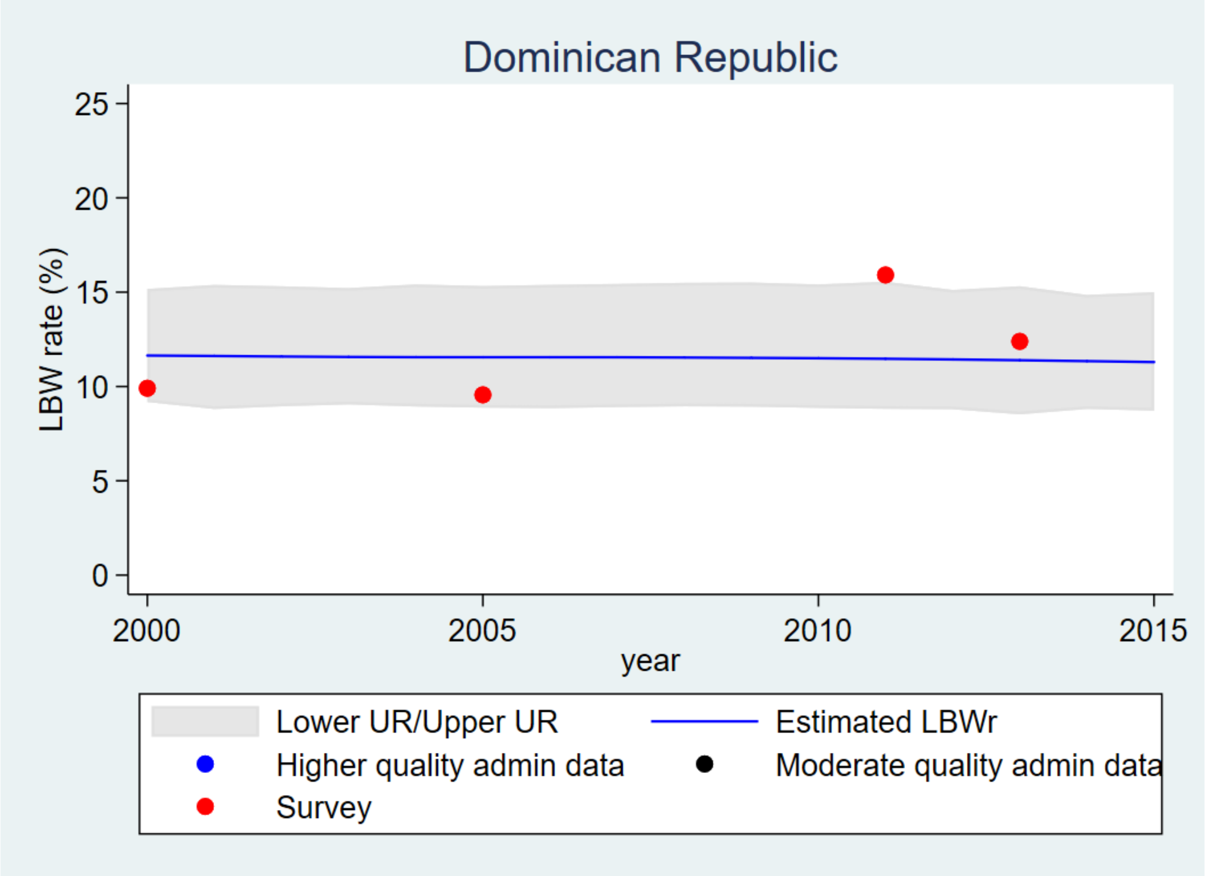
Dominican Republic
LBW rate (%)
25
20
15
10
5
0
2000
2005
2010
2015
year
Lower UR/Upper UR
Estimated LBWr
Higher quality admin data
Moderate quality admin data
Survey

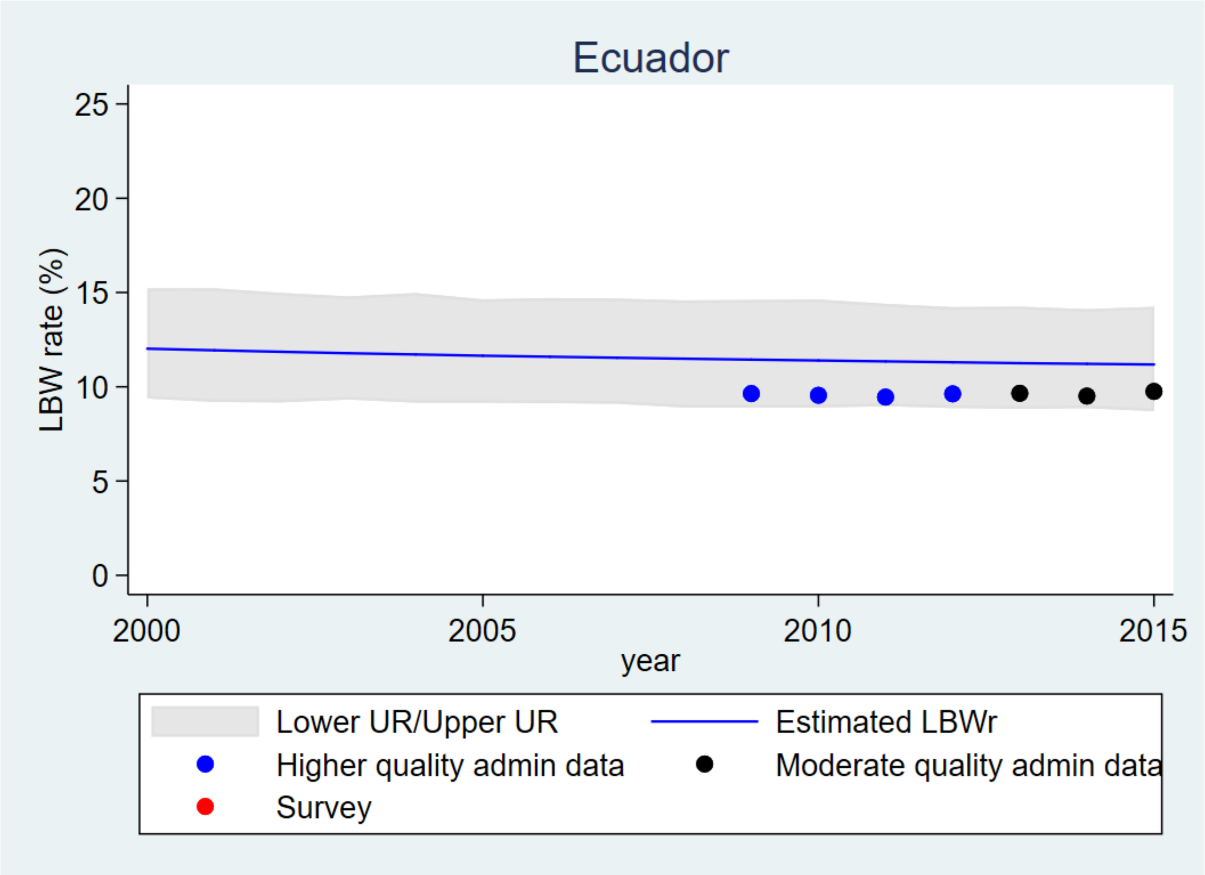
Ecuador
LBW rate (%)
25
20
15
10
5
0
2000
2005
2010
2015
year
Lower UR/Upper UR
Estimated LBWr
Higher quality admin data
Moderate quality admin data
Survey

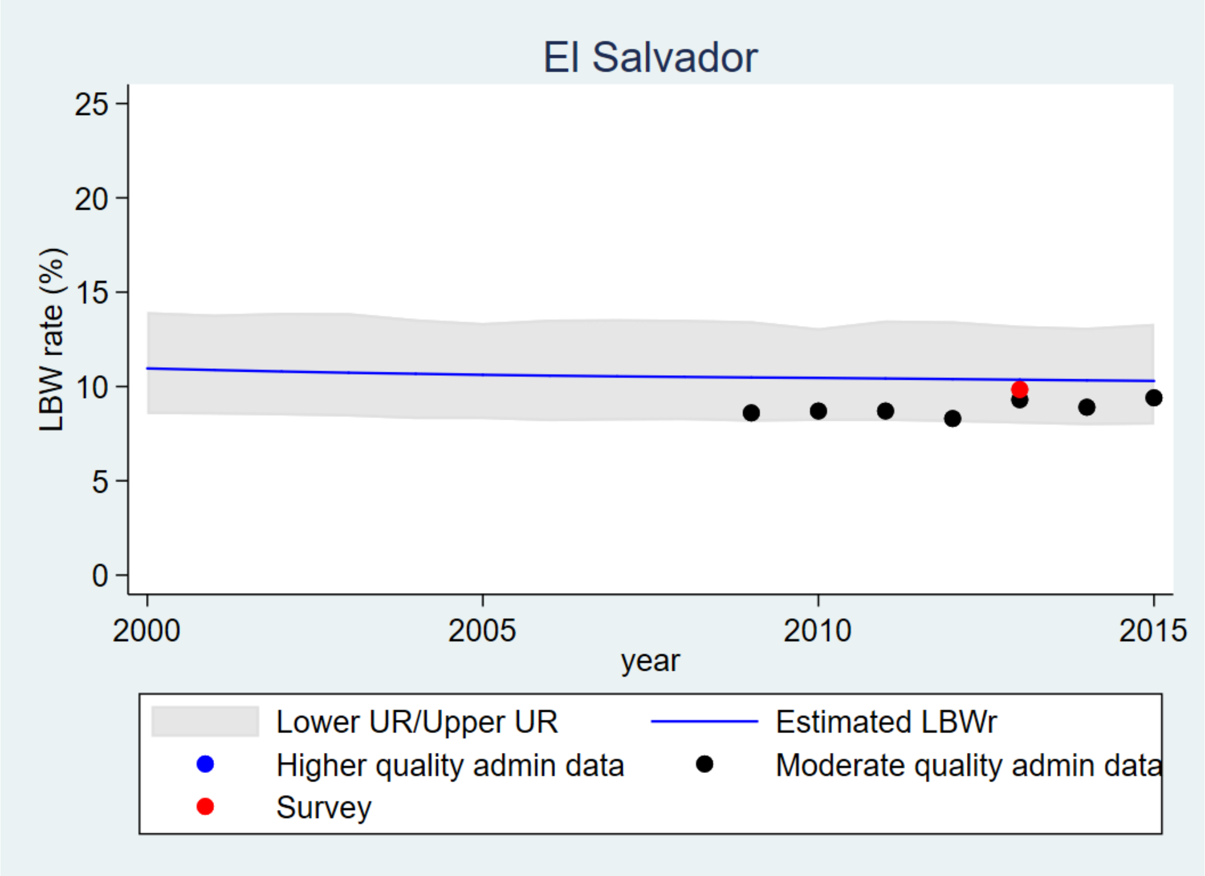
El Salvador
LBW rate (%)
25
20
15
10
5
0
2000
2005
2010
2015
year
Lower UR/Upper UR
Estimated LBWr
Higher quality admin data
Moderate quality admin data
Survey

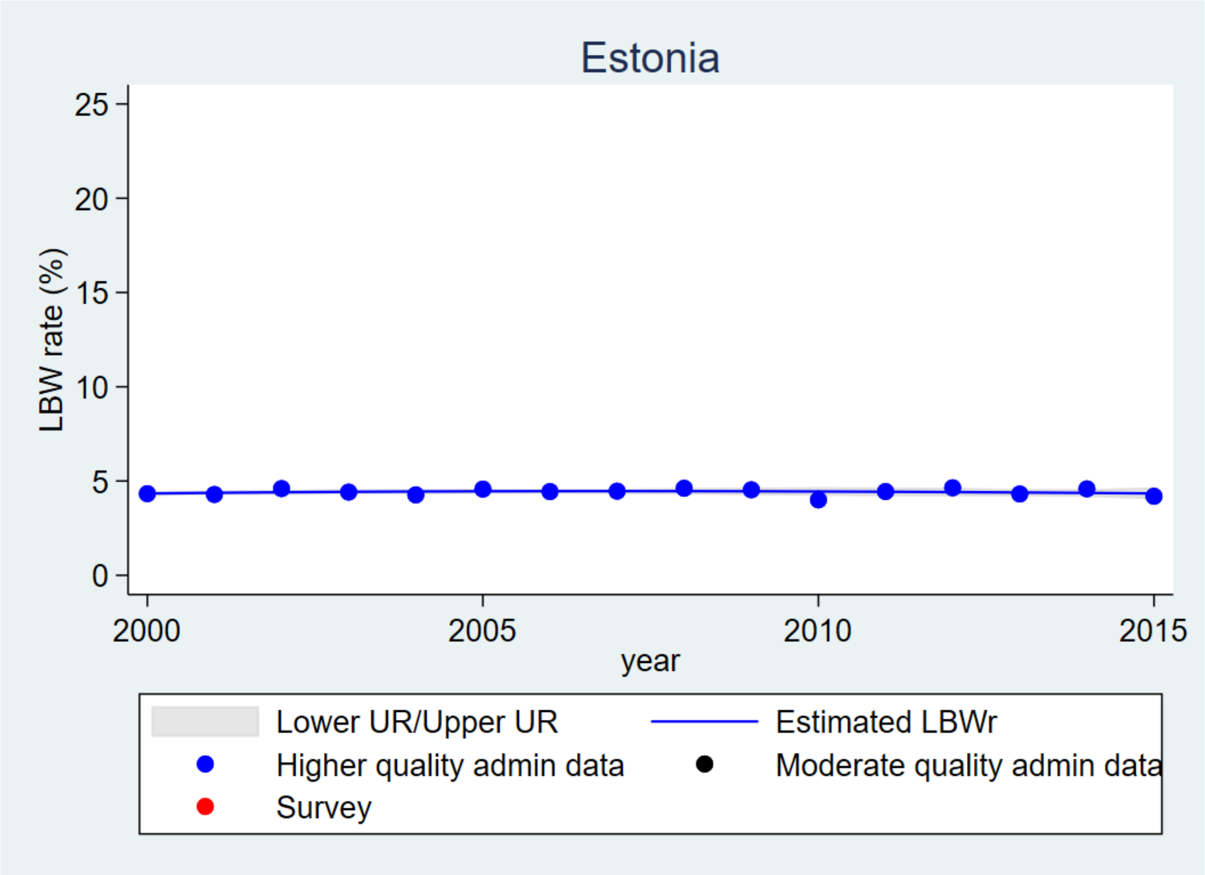
Estonia
LBW rate (%)
25
20
15
10
5
0
2000
2005
2010
2015
year
Lower UR/Upper UR
Estimated LBWr
Higher quality admin data
Moderate quality admin data
Survey

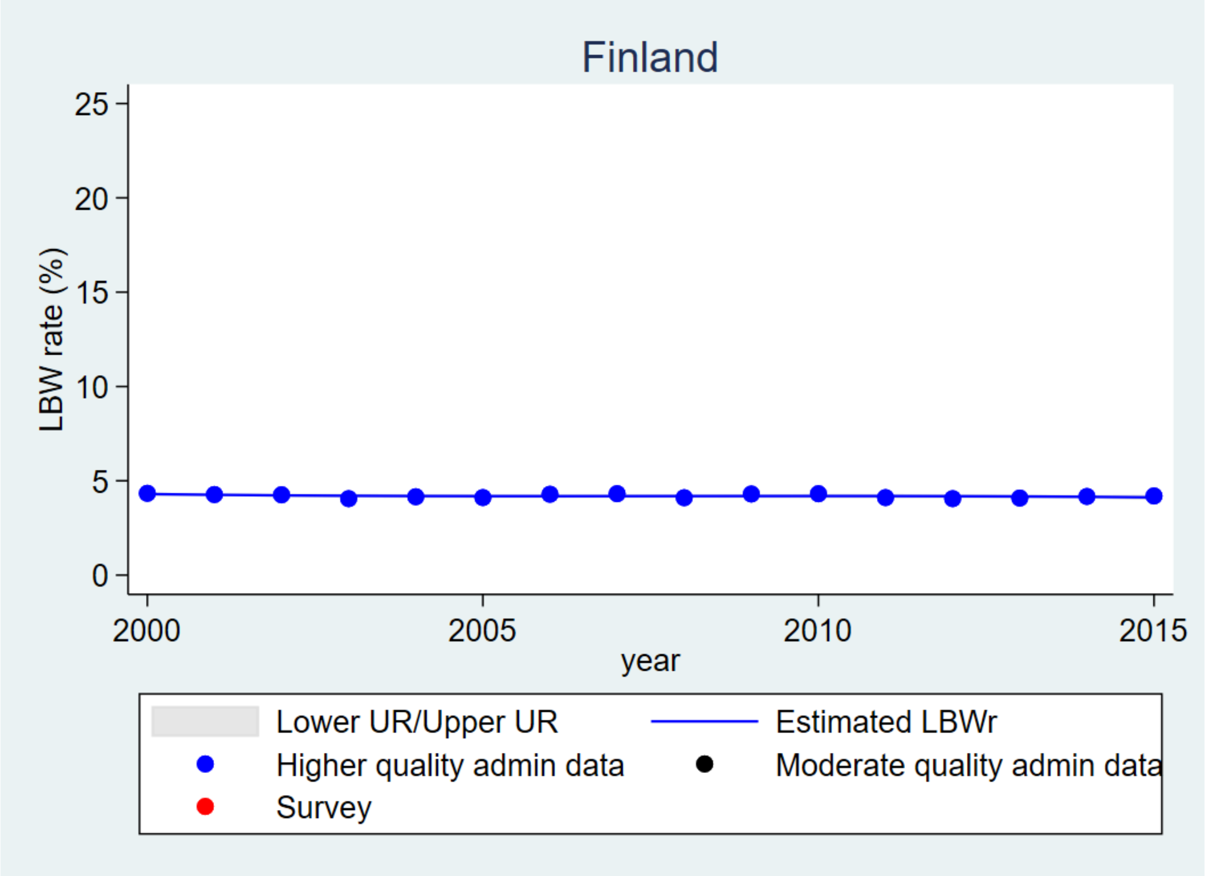
Finland
LBW rate (%)
25
20
15
10
5
0
2000
2005
2010
2015
year
Lower UR/Upper UR
Estimated LBWr
Higher quality admin data
Moderate quality admin data
Survey

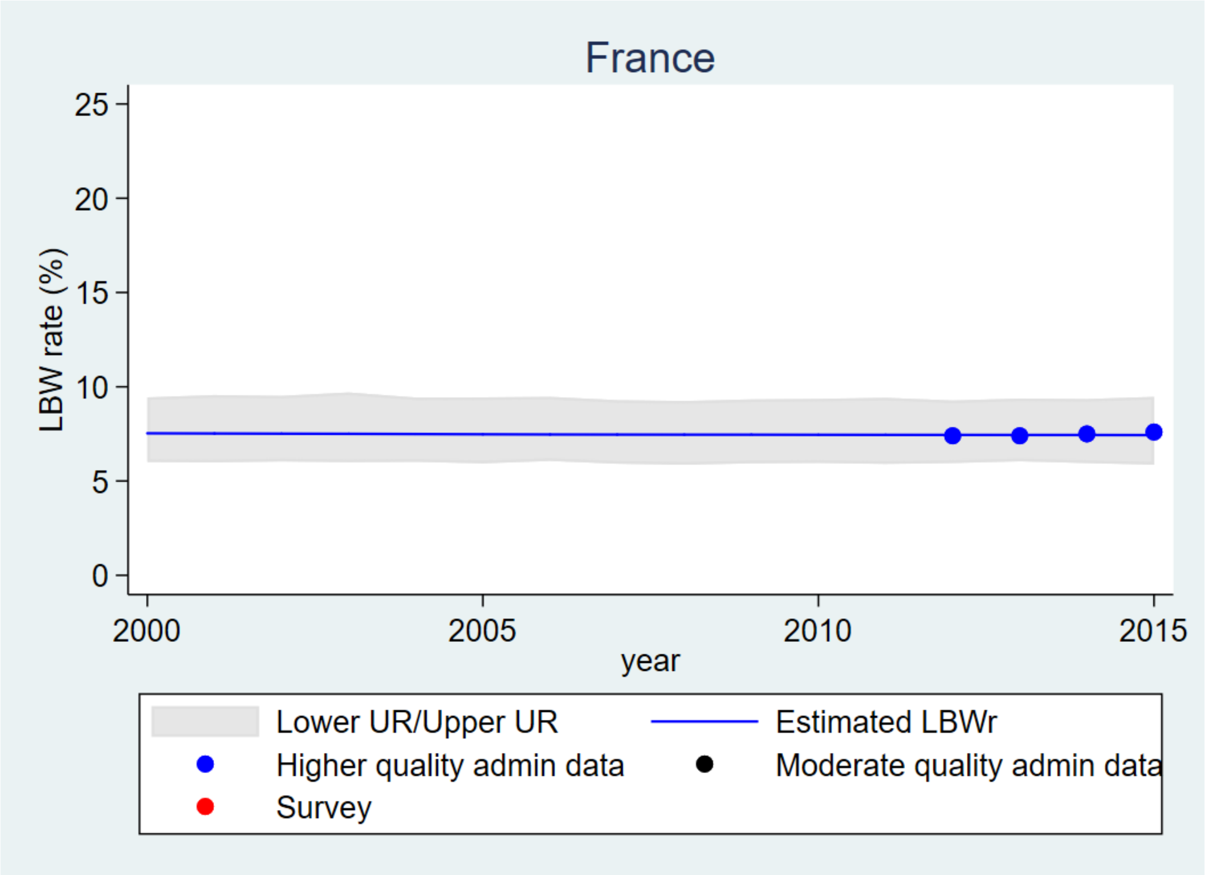
France
LBW rate (%)
25
20
15
10
5
0
2000
2005
2010
2015
year
Lower UR/Upper UR
Estimated LBWr
Higher quality admin data
Moderate quality admin data
Survey

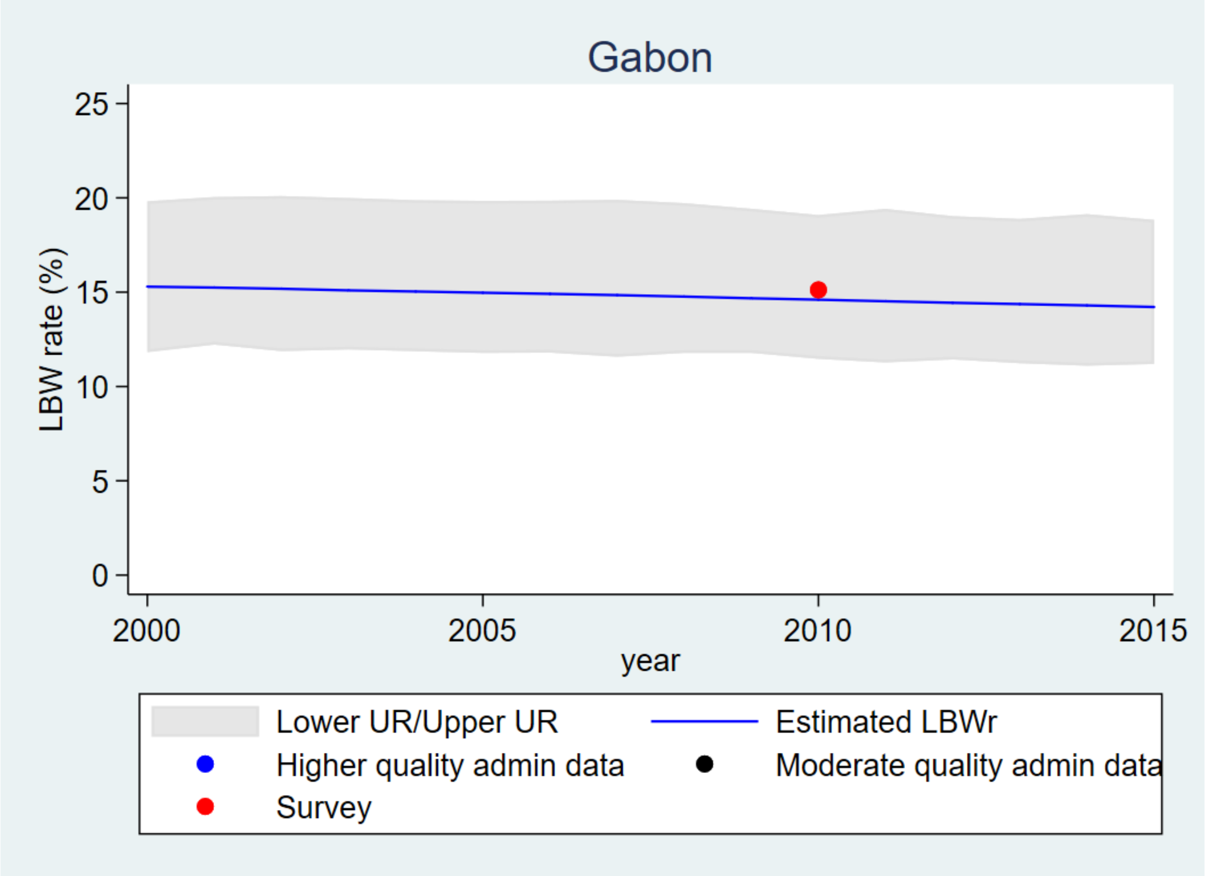
Gabon
LBW rate (%)
25
20
15
10
5
0
2000
2005
2010
2015
year
Lower UR/Upper UR
Estimated LBWr
Higher quality admin data
Moderate quality admin data
Survey

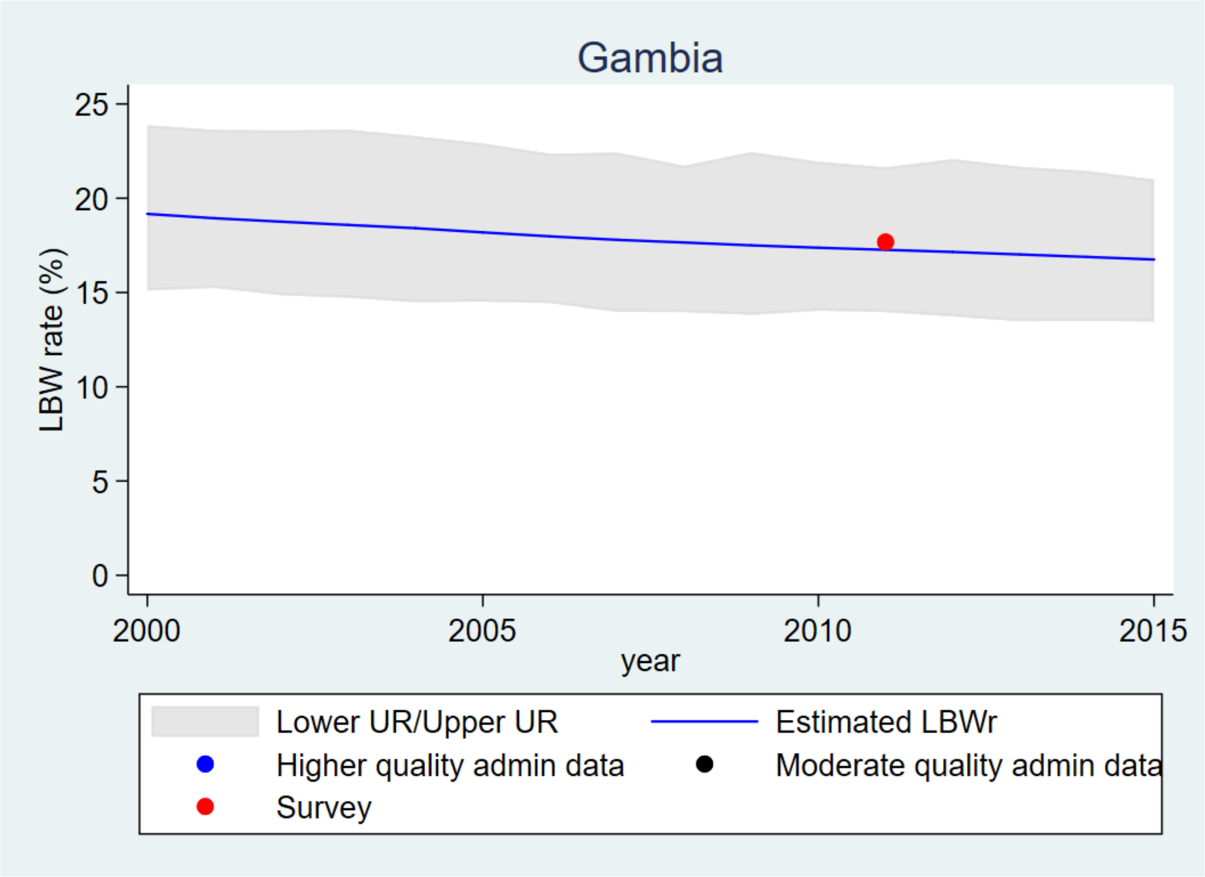
Gambia
LBW rate (%)
25
20
15
10
5
0
2000
2005
2010
2015
year
Lower UR/Upper UR
Estimated LBWr
Higher quality admin data
Moderate quality admin data
Survey

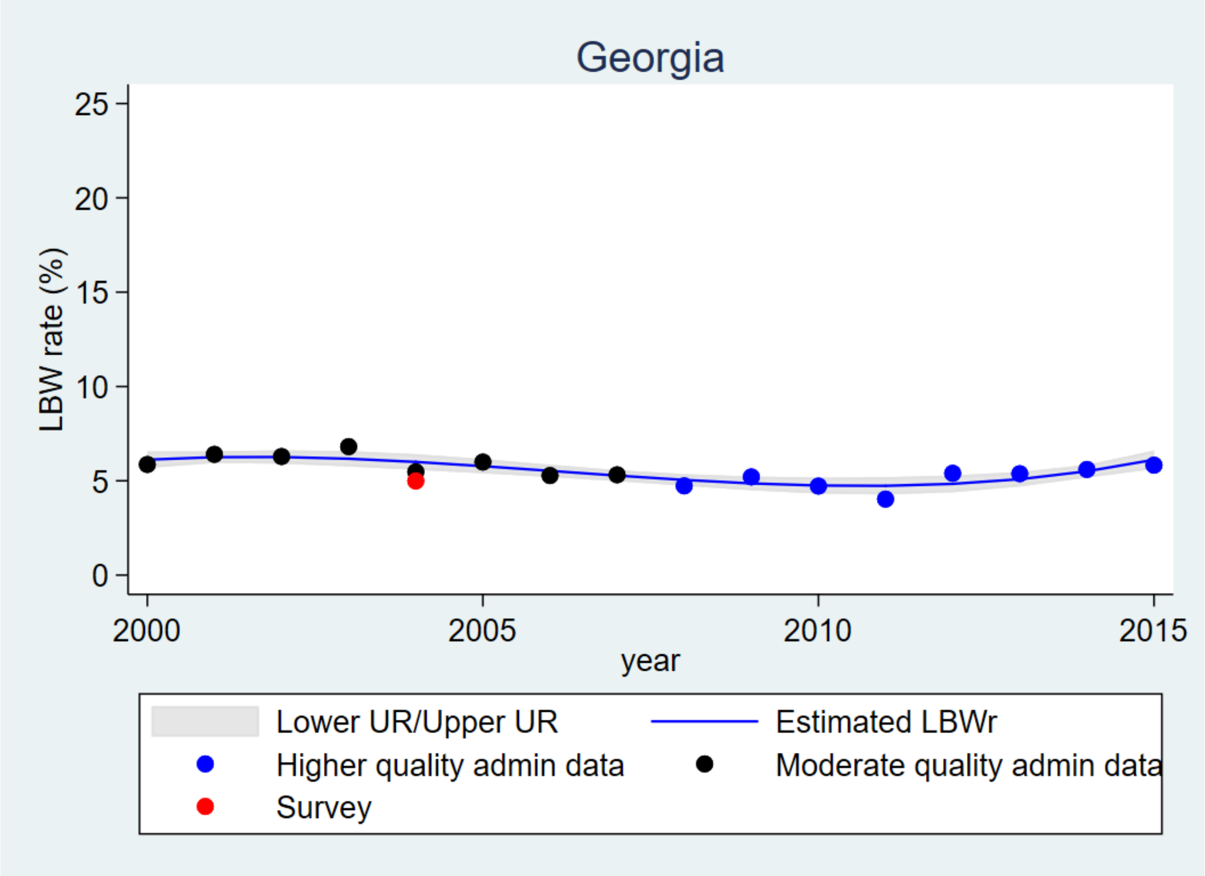
Georgia
LBW rate (%)
25
20
15
10
5
0
2000
2005
2010
2015
year
Lower UR/Upper UR
Estimated LBWr
Higher quality admin data
Moderate quality admin data
Survey

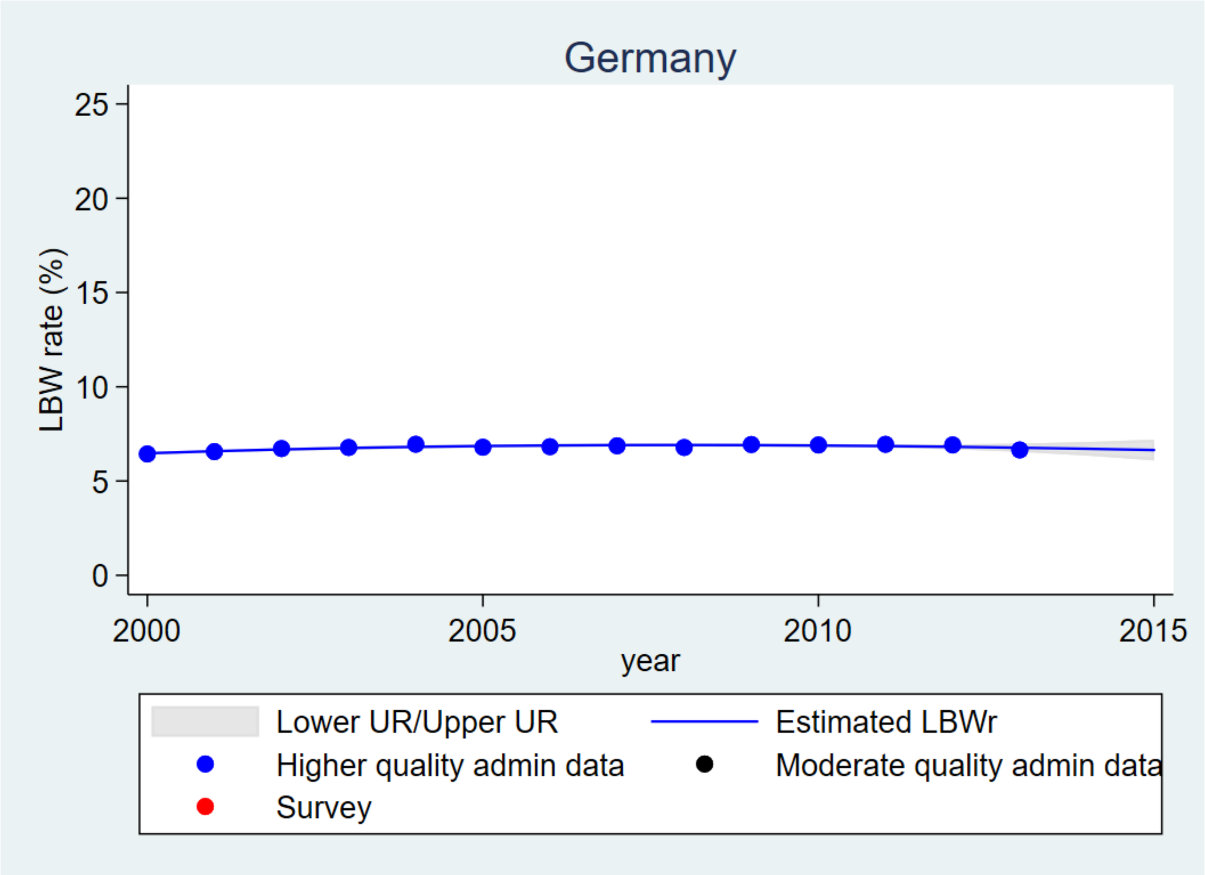
Germany
LBW rate (%)
25
20
15
10
5
0
2000
2005
2010
2015
year
Lower UR/Upper UR
Estimated LBWr
Higher quality admin data
Moderate quality admin data
Survey

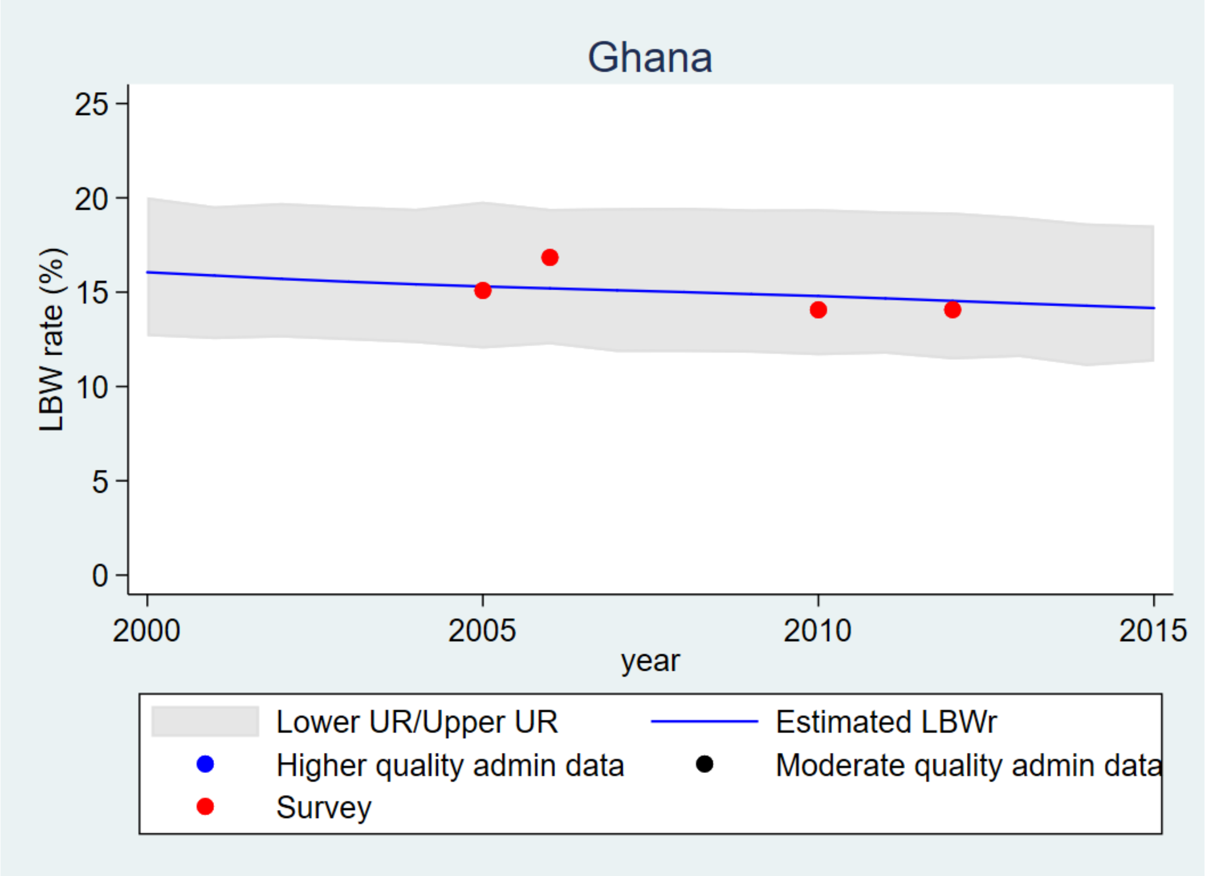
Ghana
LBW rate (%)
25
20
15
10
5
0
2000
2005
2010
2015
year
Lower UR/Upper UR
Estimated LBWr
Higher quality admin data
Moderate quality admin data
Survey

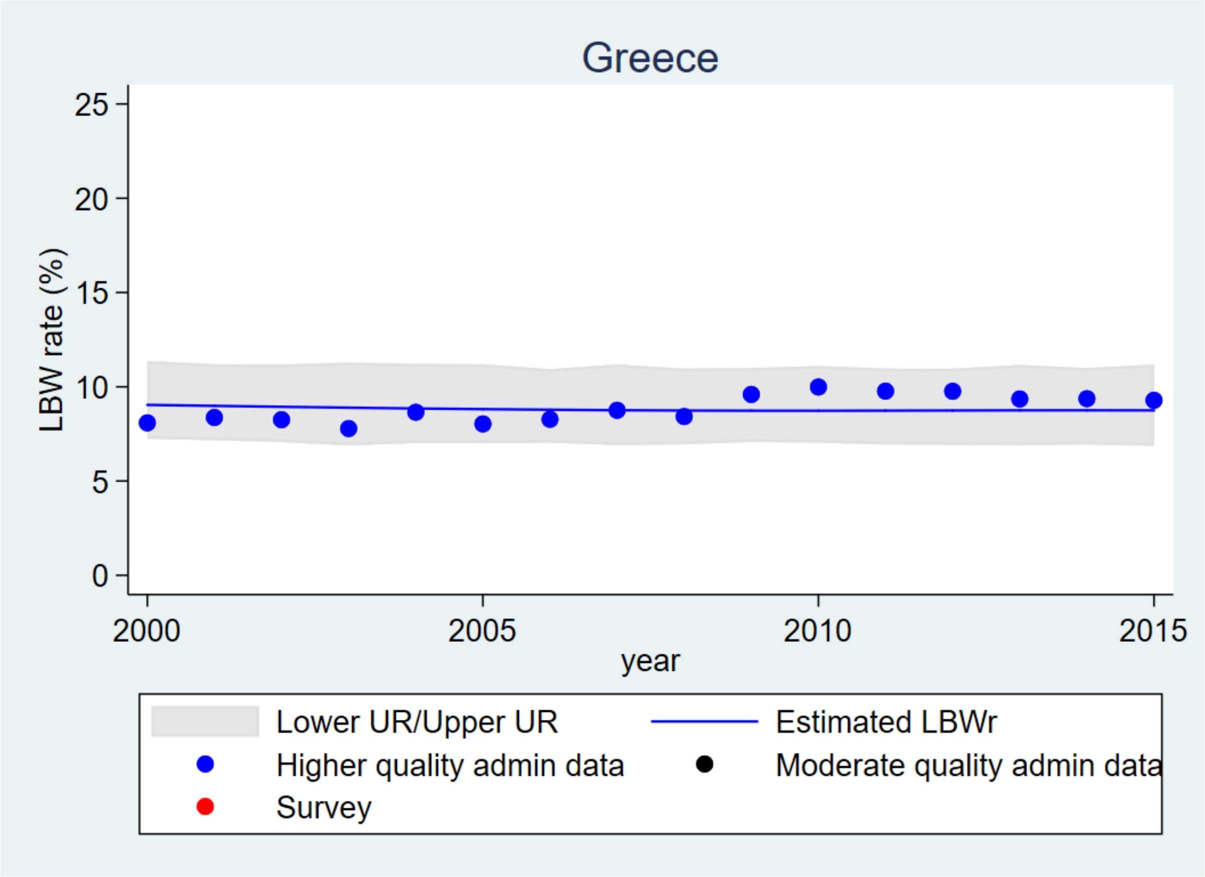
Greece
LBW rate (%)
25
20
15
10
5
0
2000
2005
2010
2015
year
Lower UR/Upper UR
Estimated LBWr
Higher quality admin data
Moderate quality admin data
Survey

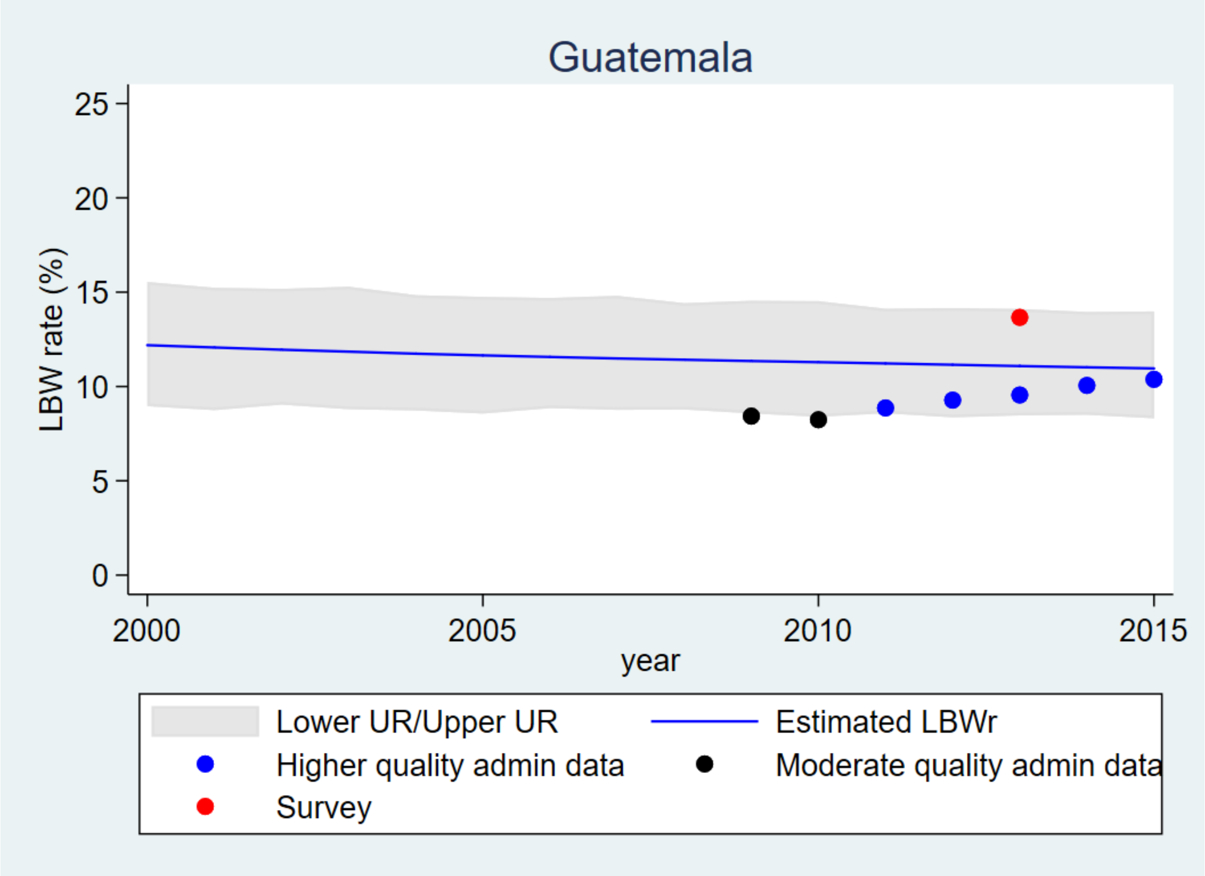
Guatemala
LBW rate (%)
25
20
15
10
5
0
2000
2005
2010
2015
year
Lower UR/Upper UR
Estimated LBWr
Higher quality admin data
Moderate quality admin data
Survey

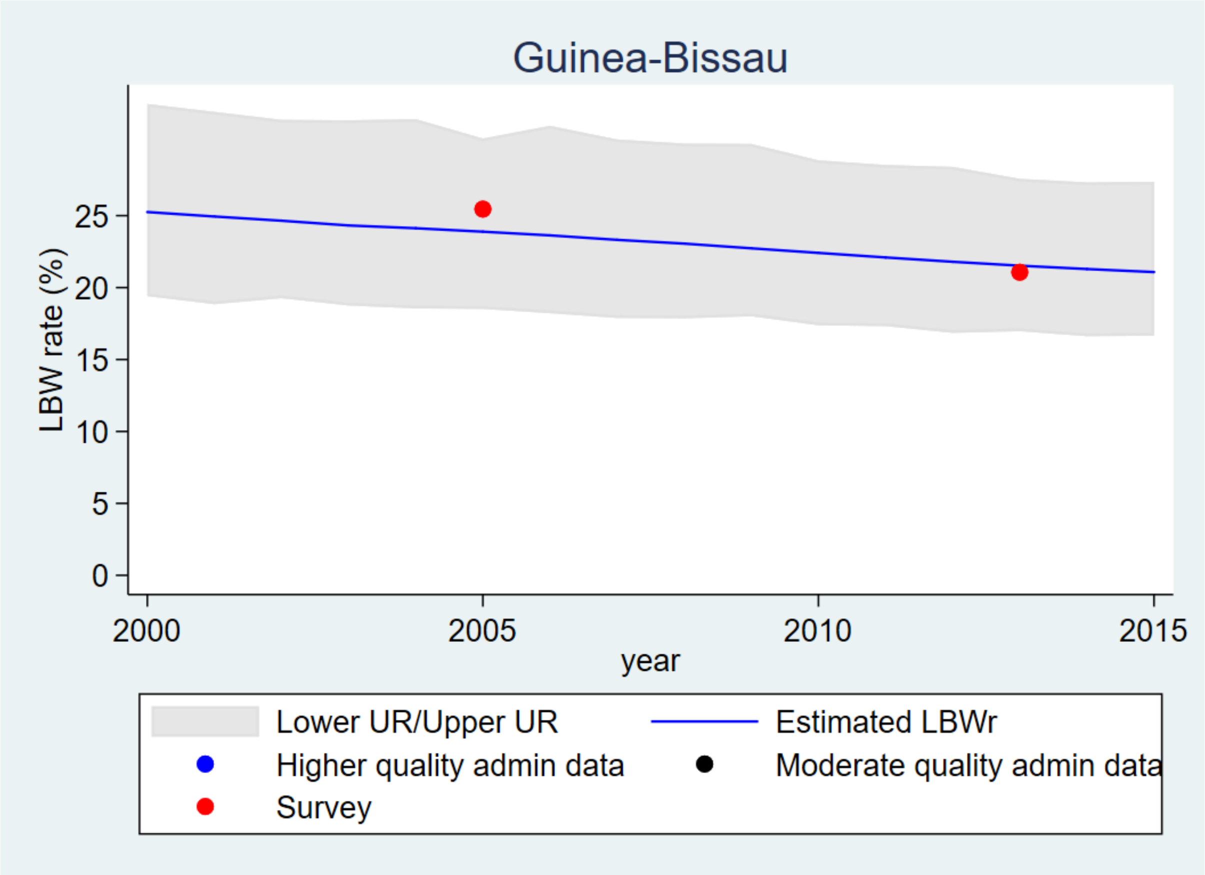
Guinea-Bissau
LBW rate (%)
0
5
10
15
20
25
2000
2005
2010
2015
year
Lower UR/Upper UR
Estimated LBWr
Higher quality admin data
Moderate quality admin data
Survey

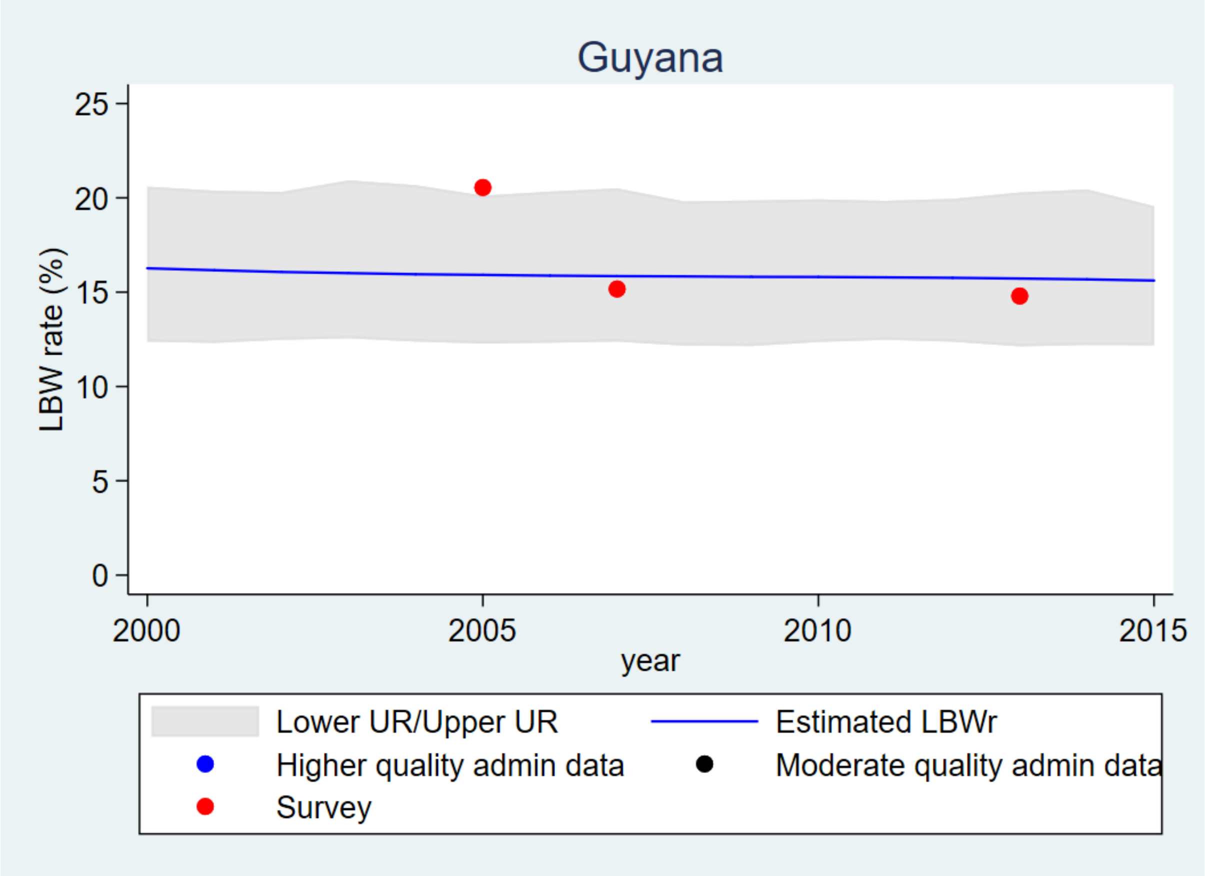
Guyana
LBW rate (%)
0
5
10
15
20
25
2000
2005
2010
2015
year
Lower UR/Upper UR
Estimated LBWr
Higher quality admin data
Moderate quality admin data
Survey

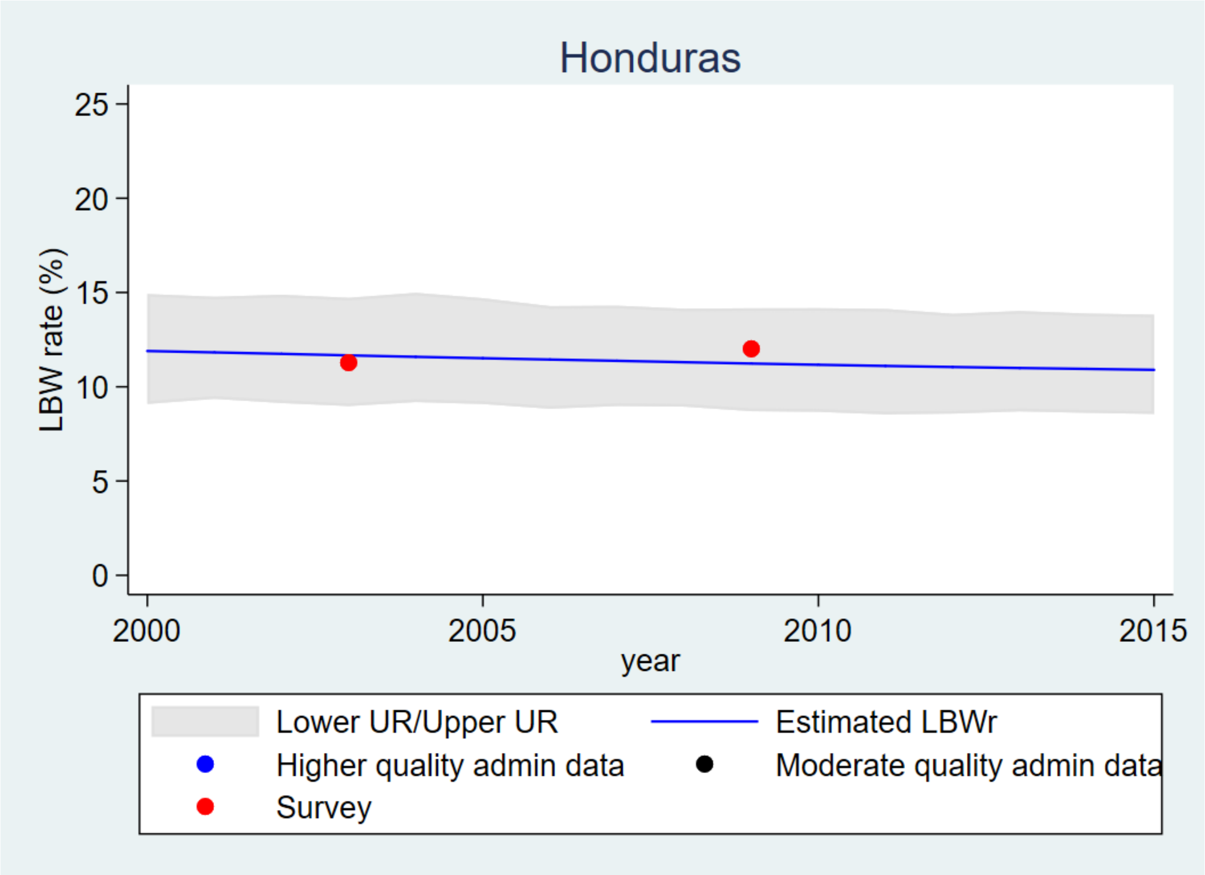
Honduras
LBW rate (%)
25
20
15
10
5
0
2000
2005
2010
2015
year
Lower UR/Upper UR
Estimated LBWr
Higher quality admin data
Moderate quality admin data
Survey

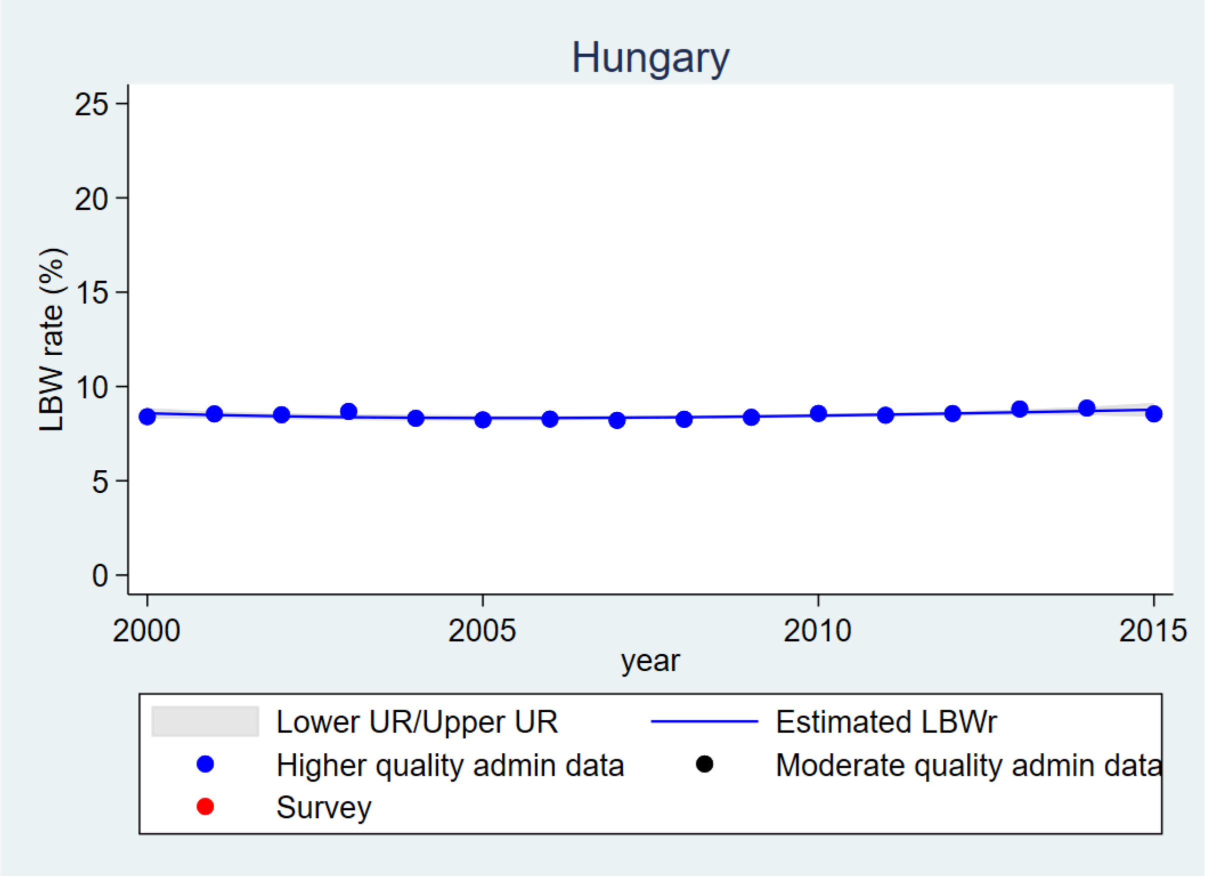
Hungary
LBW rate (%)
25
20
15
10
5
0
2000
2005
2010
2015
year
Lower UR/Upper UR
Estimated LBWr
Higher quality admin data
Moderate quality admin data
Survey

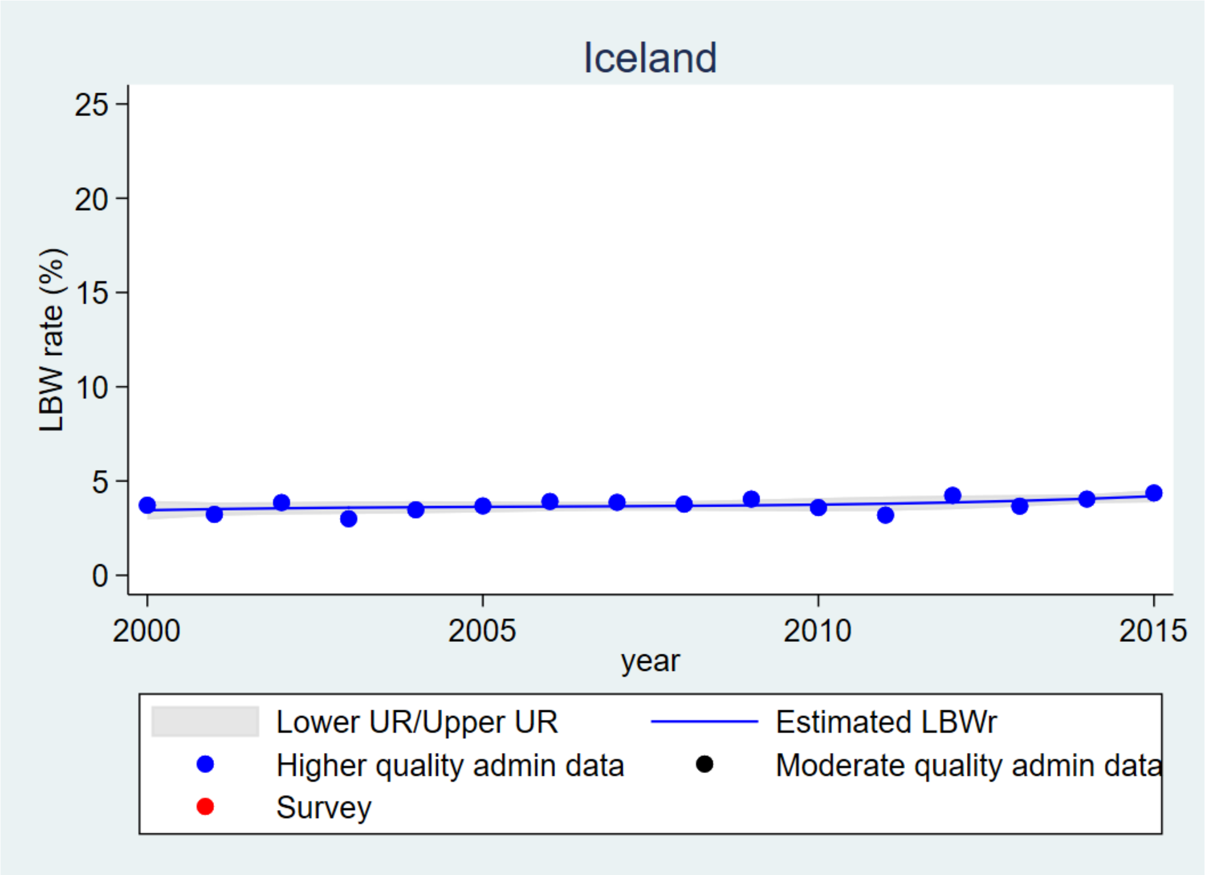

Iceland
LBW rate (%)
25
20
15
10
5
0
2000
2005
2010
2015
year
Lower UR/Upper UR
Estimated LBWr
Higher quality admin data
Moderate quality admin data
Survey

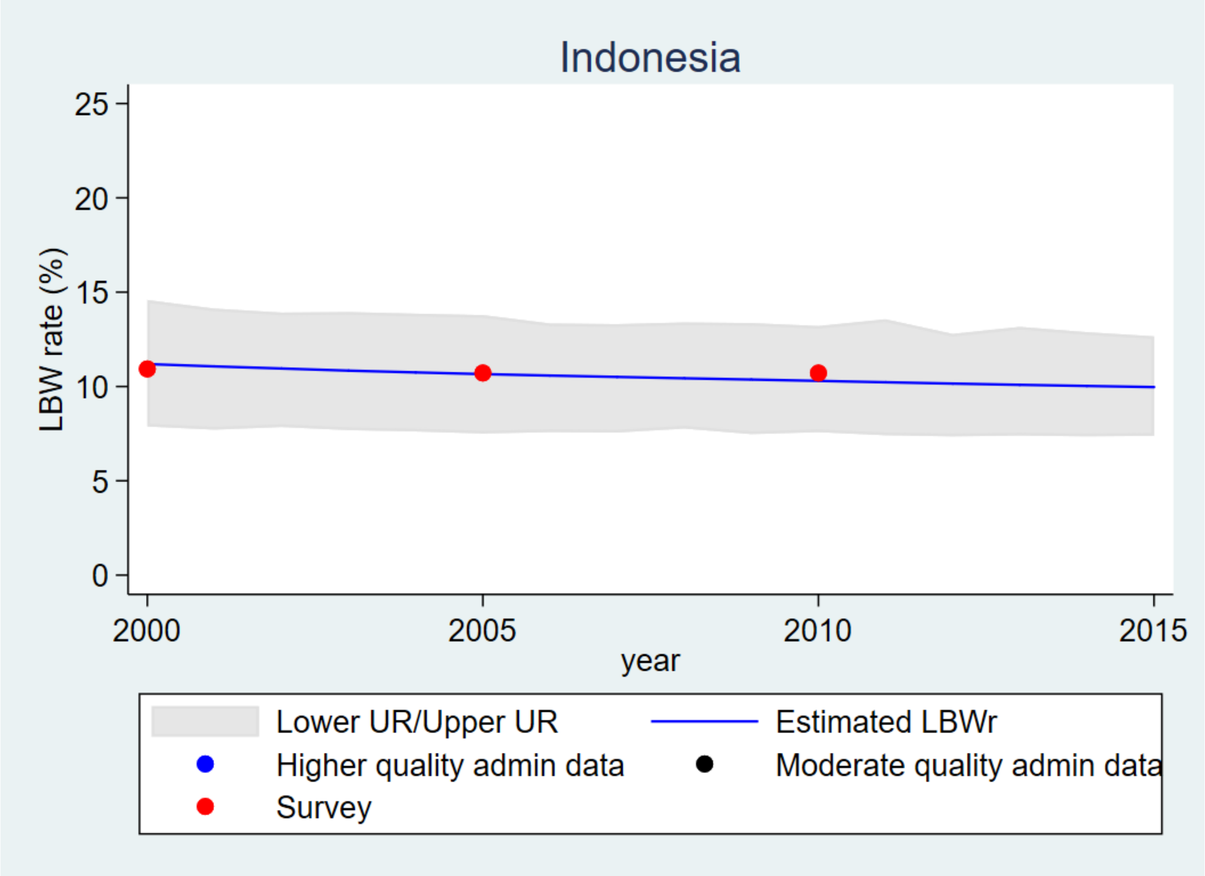

Indonesia
LBW rate (%)
25
20
15
10
5
0
2000
2005
2010
2015
year
Lower UR/Upper UR
Estimated LBWr
Higher quality admin data
Moderate quality admin data
Survey

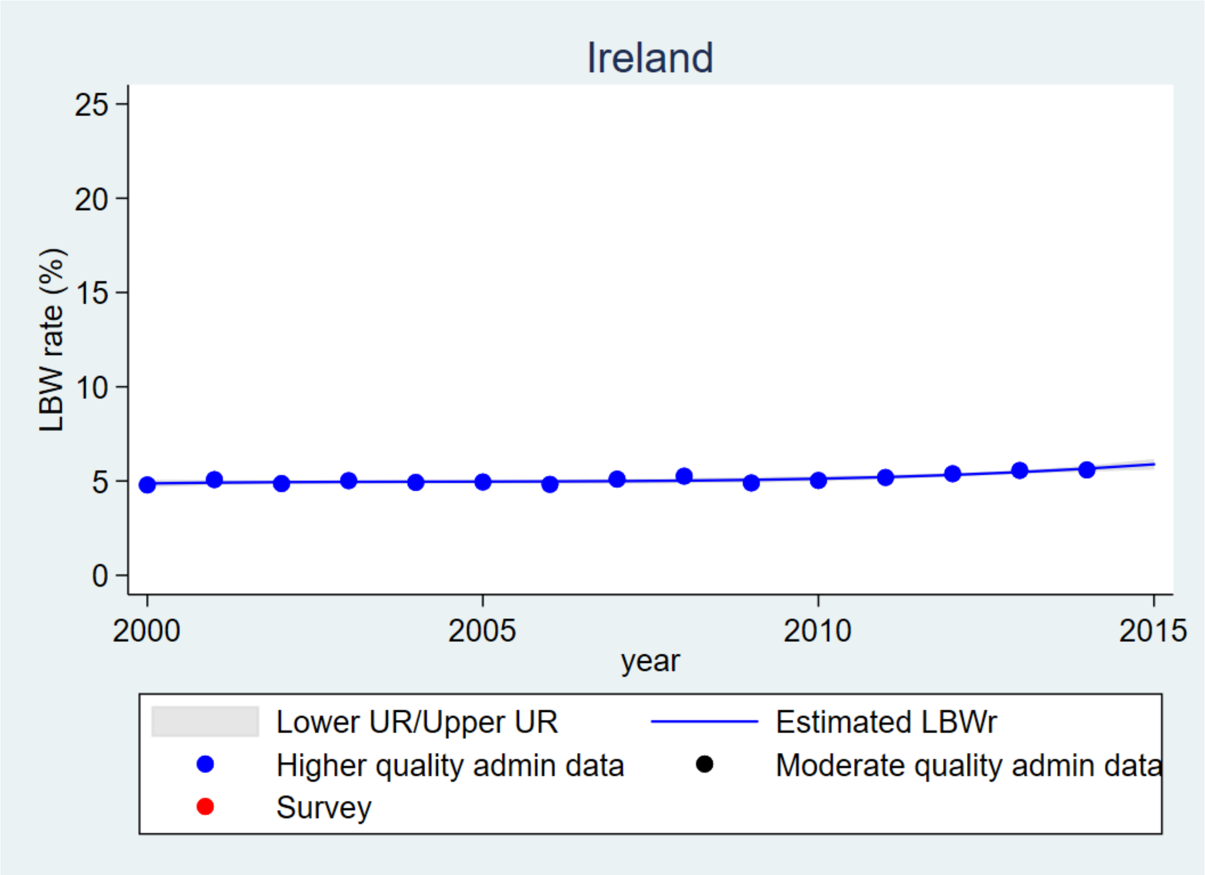
Ireland
LBW rate (%)
25
20
15
10
5
0
2000
2005
2010
2015
year
Lower UR/Upper UR
Estimated LBWr
Higher quality admin data
Moderate quality admin data
Survey

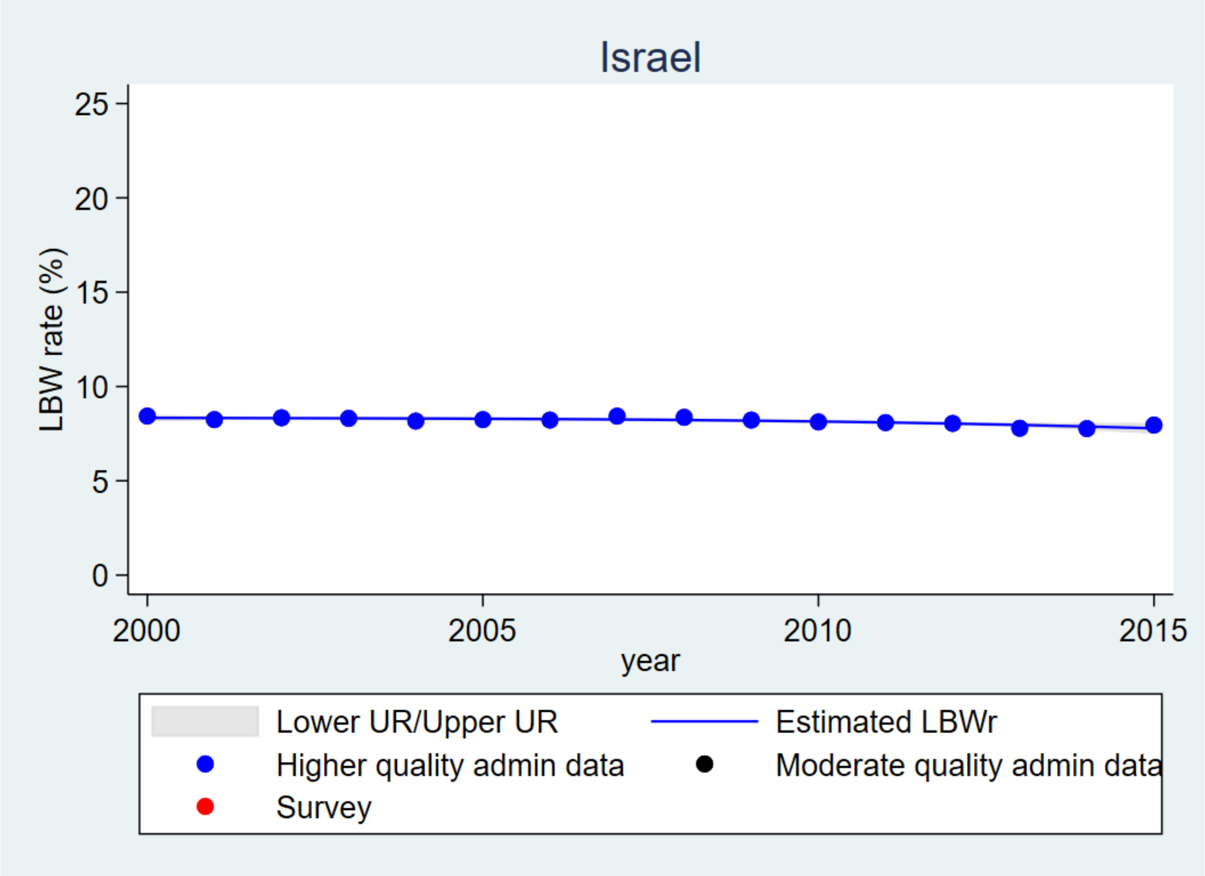
Israel
LBW rate (%)
25
20
15
10
5
0
2000
2005
2010
2015
year
Lower UR/Upper UR
Estimated LBWr
Higher quality admin data
Moderate quality admin data
Survey

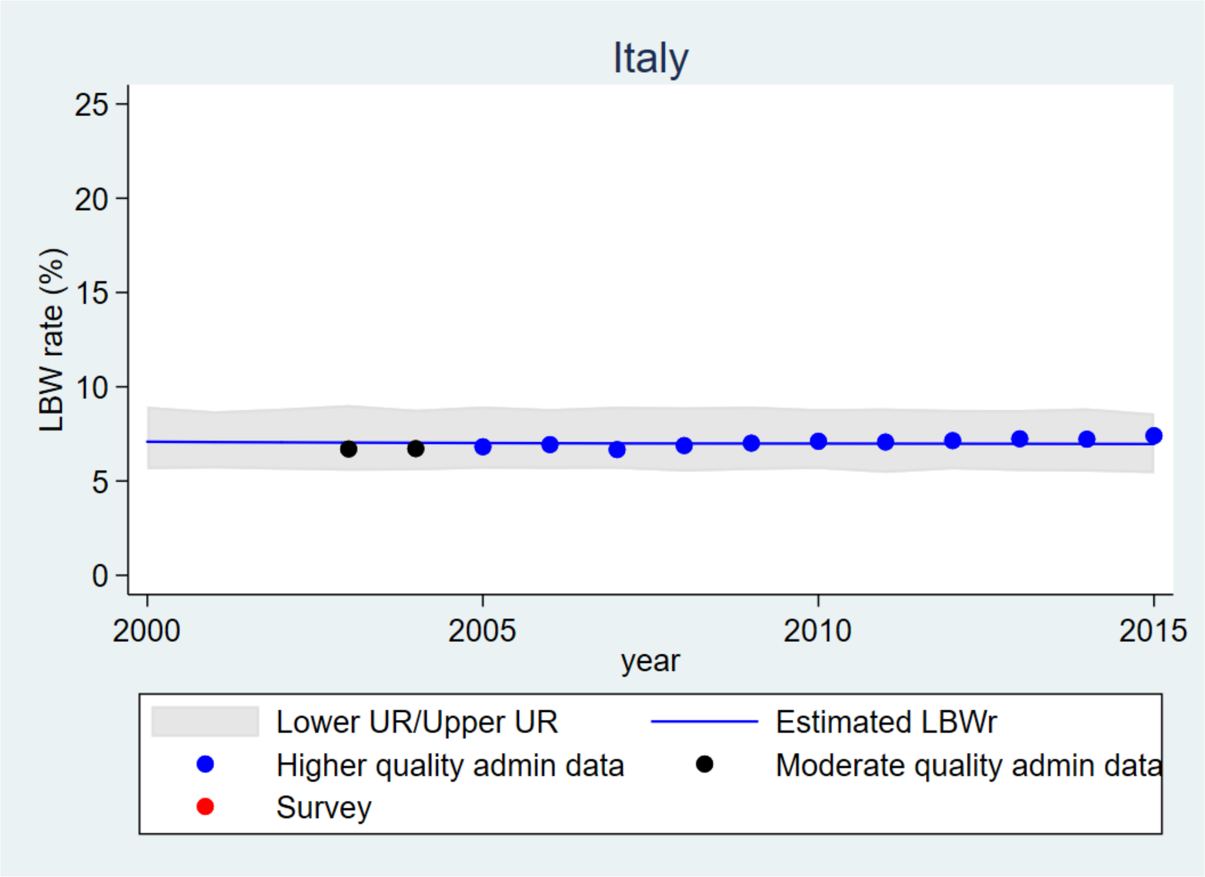
Italy
LBW rate (%)
25
20
15
10
5
0
2000
2005
2010
2015
year
Lower UR/Upper UR
Estimated LBWr
Higher quality admin data
Moderate quality admin data
Survey

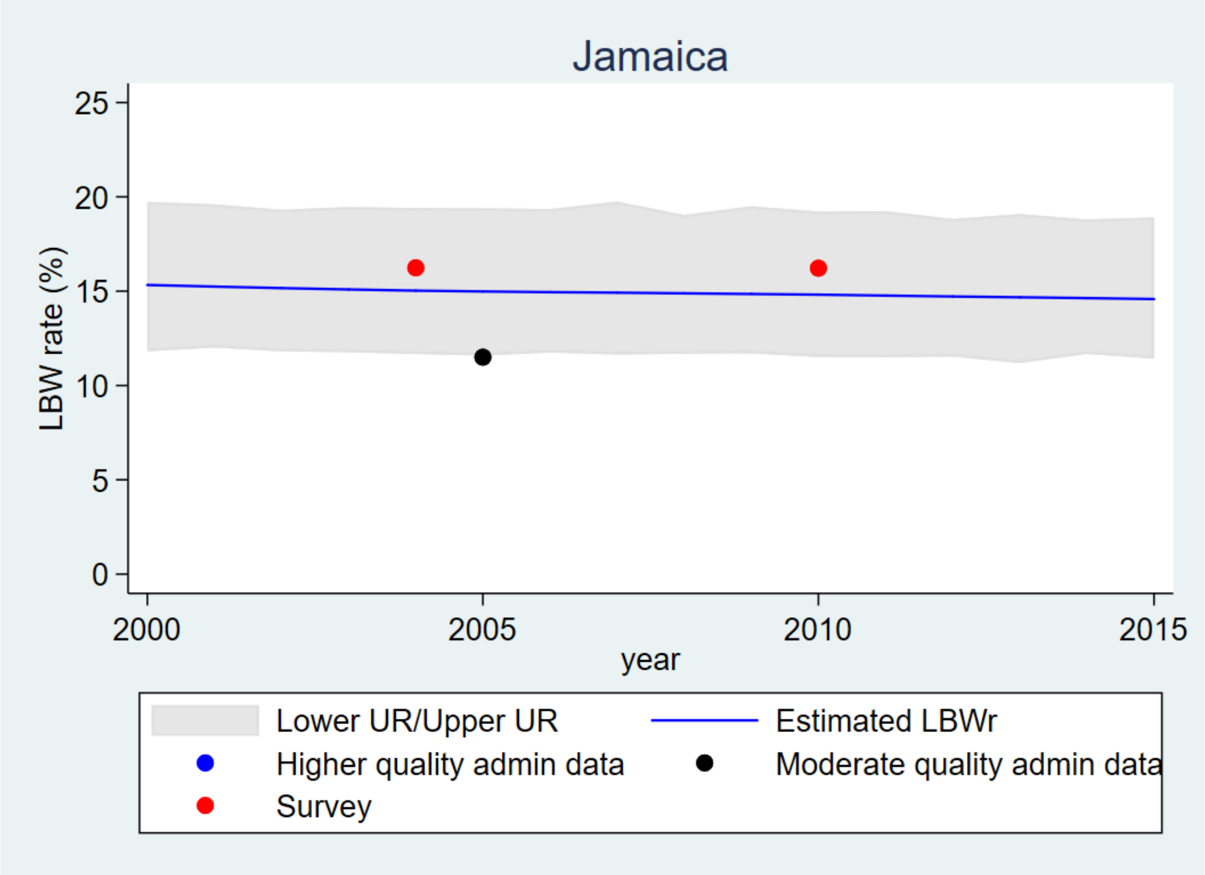
Jamaica
LBW rate (%)
25
20
15
10
5
0
2000
2005
2010
2015
year
Lower UR/Upper UR
Estimated LBWr
Higher quality admin data
Moderate quality admin data
Survey

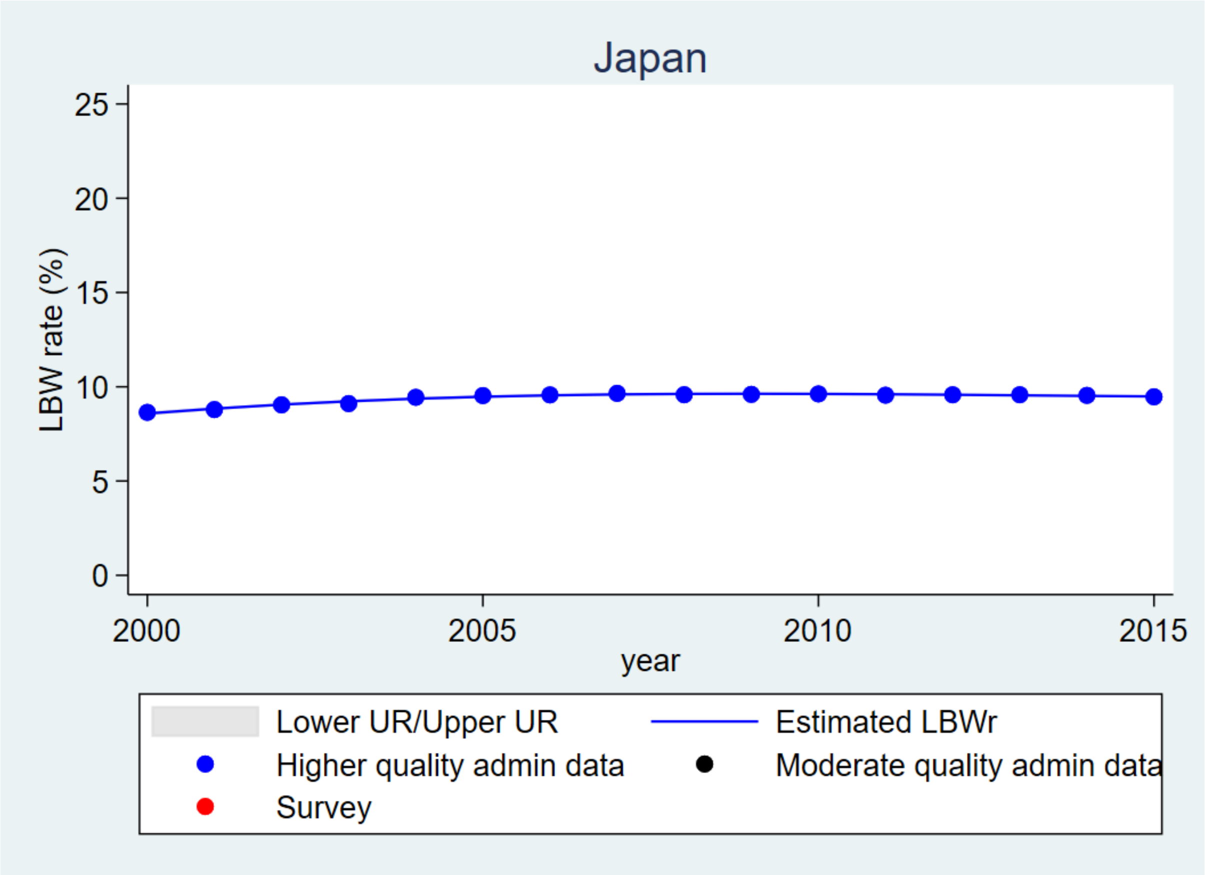

Japan
LBW rate (%)
25
20
15
10
5
0
2000
2005
2010
2015
year
Lower UR/Upper UR
Estimated LBWr
Higher quality admin data
Moderate quality admin data
Survey

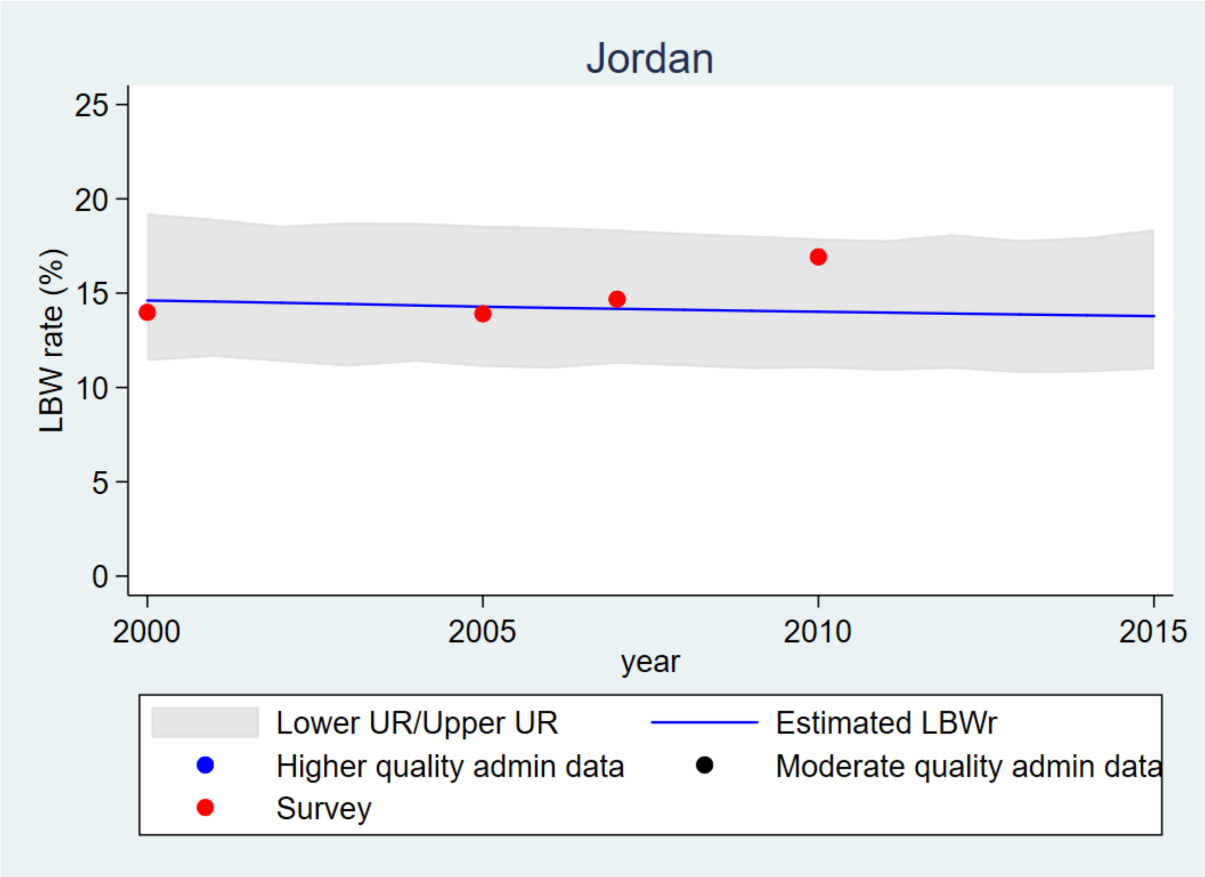

Jordan
LBW rate (%)
25
20
15
10
5
0
2000
2005
2010
2015
year
Lower UR/Upper UR
Estimated LBWr
Higher quality admin data
Moderate quality admin data
Survey

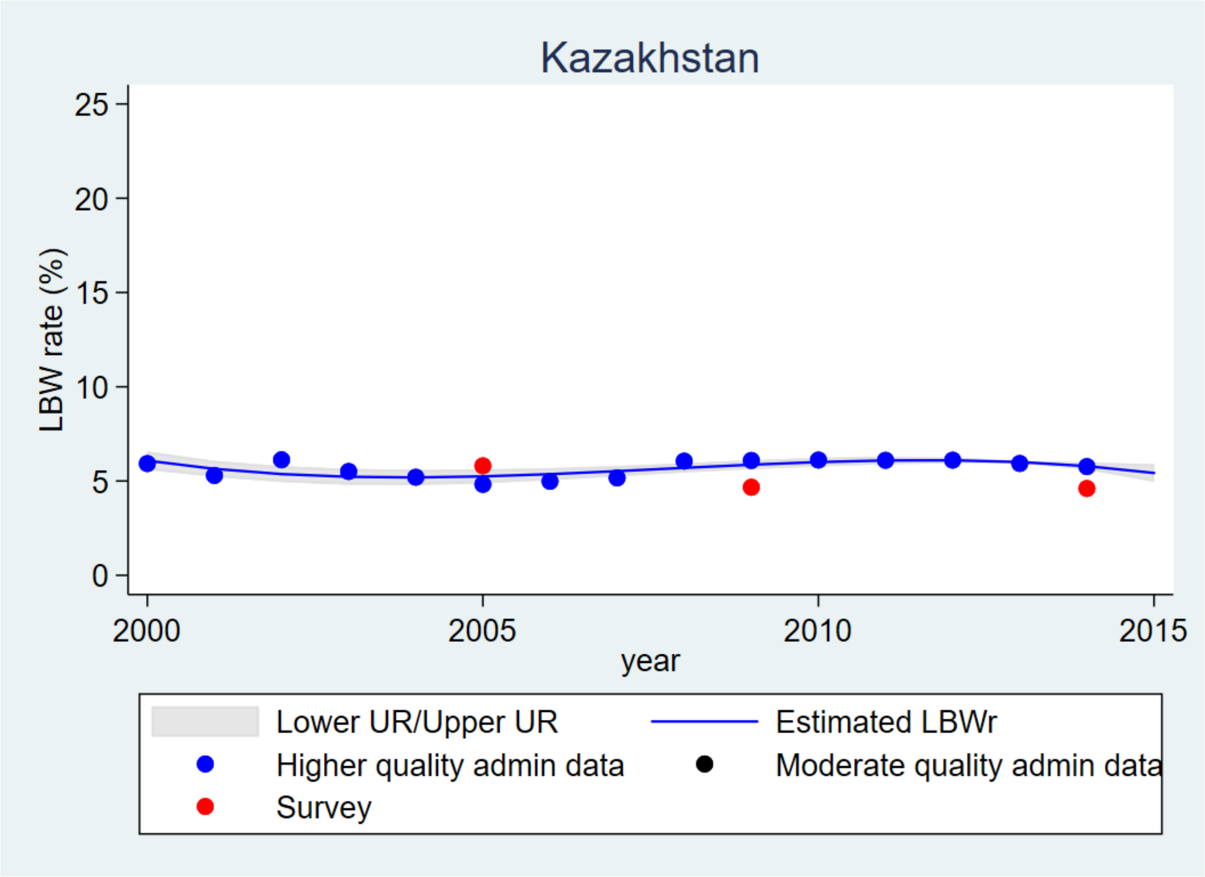
Kazakhstan
LBW rate (%)
25
20
15
10
5
0
2000
2005
2010
2015
year
Lower UR/Upper UR
Estimated LBWr
Higher quality admin data
Moderate quality admin data
Survey

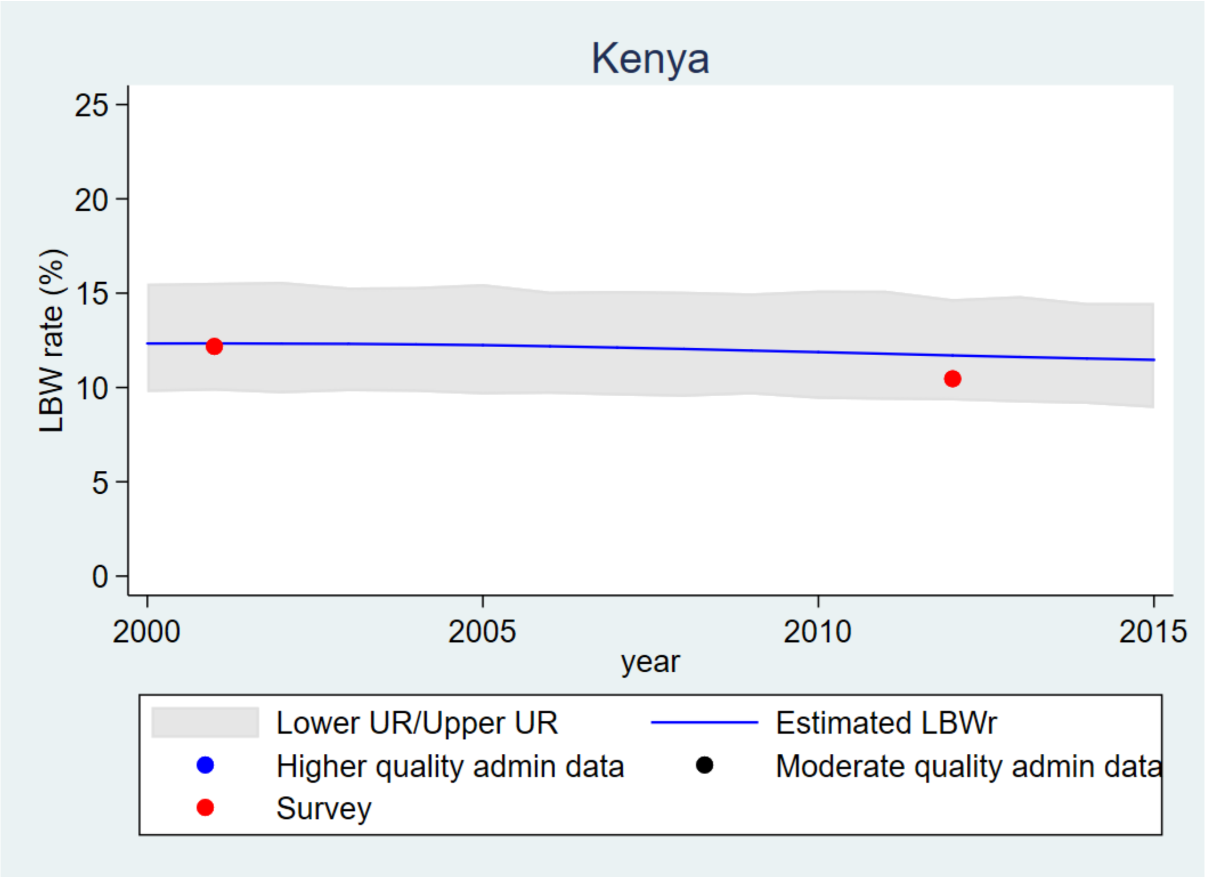
Kenya
LBW rate (%)
25
20
15
10
5
0
2000
2005
2010
2015
year
Lower UR/Upper UR
Estimated LBWr
Higher quality admin data
Moderate quality admin data
Survey

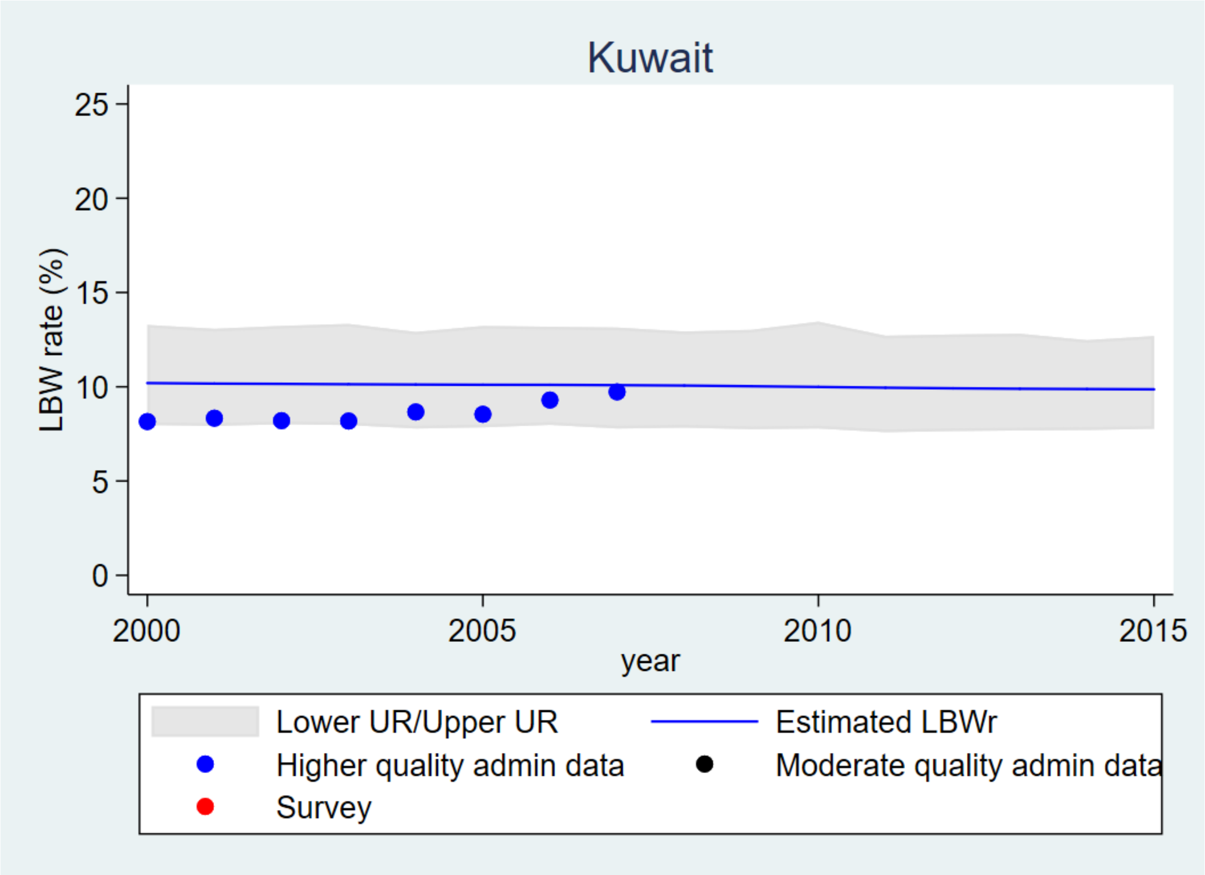
Kuwait
LBW rate (%)
25
20
15
10
5
0
2000
2005
2010
2015
year
Lower UR/Upper UR
Estimated LBWr
Higher quality admin data
Moderate quality admin data
Survey

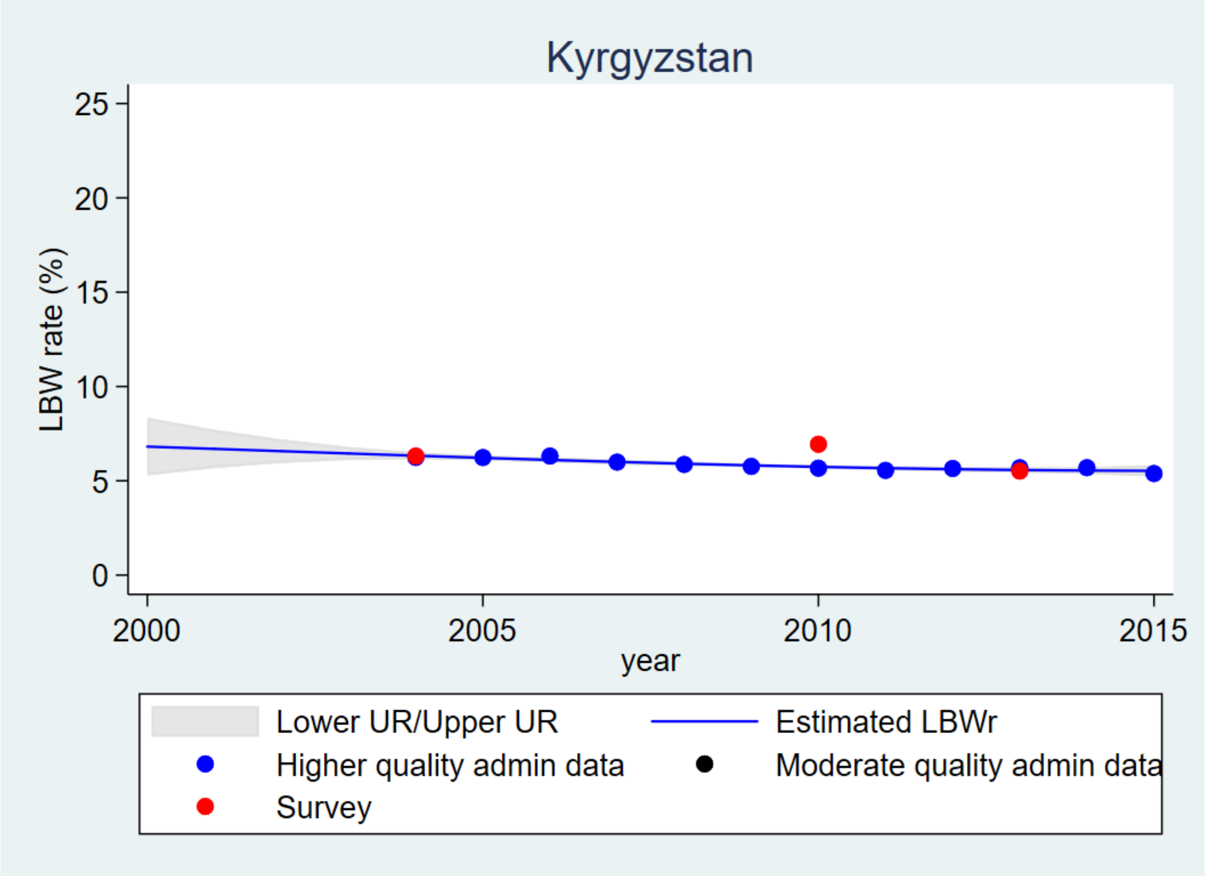
Kyrgyzstan
LBW rate (%)
25
20
15
10
5
0
2000
2005
2010
2015
year
Lower UR/Upper UR
Estimated LBWr
Higher quality admin data
Moderate quality admin data
Survey

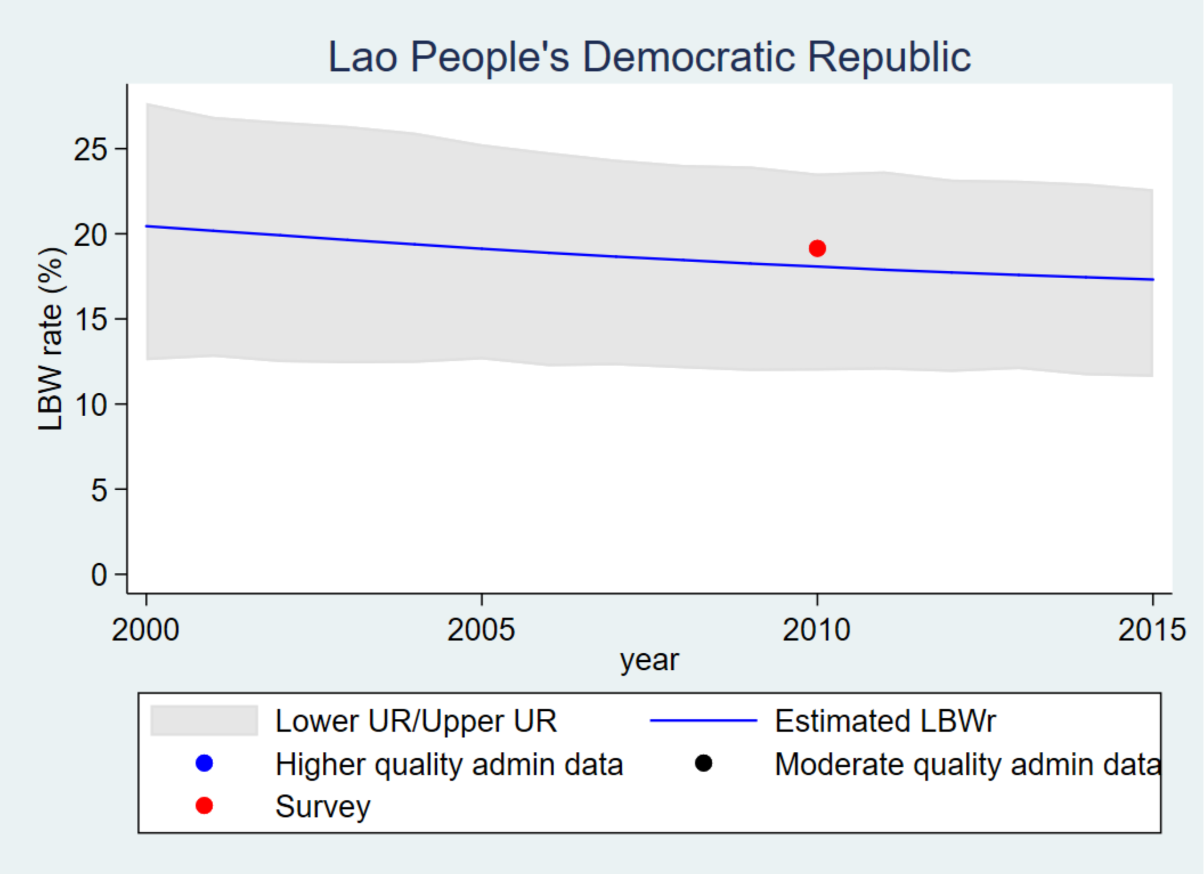
Lao People's Democratic Republic
LBW rate (%)
0
5
10
15
20
25
2000
2005
2010
2015
year
Lower UR/Upper UR
Estimated LBWr
Higher quality admin data
Moderate quality admin data
Survey

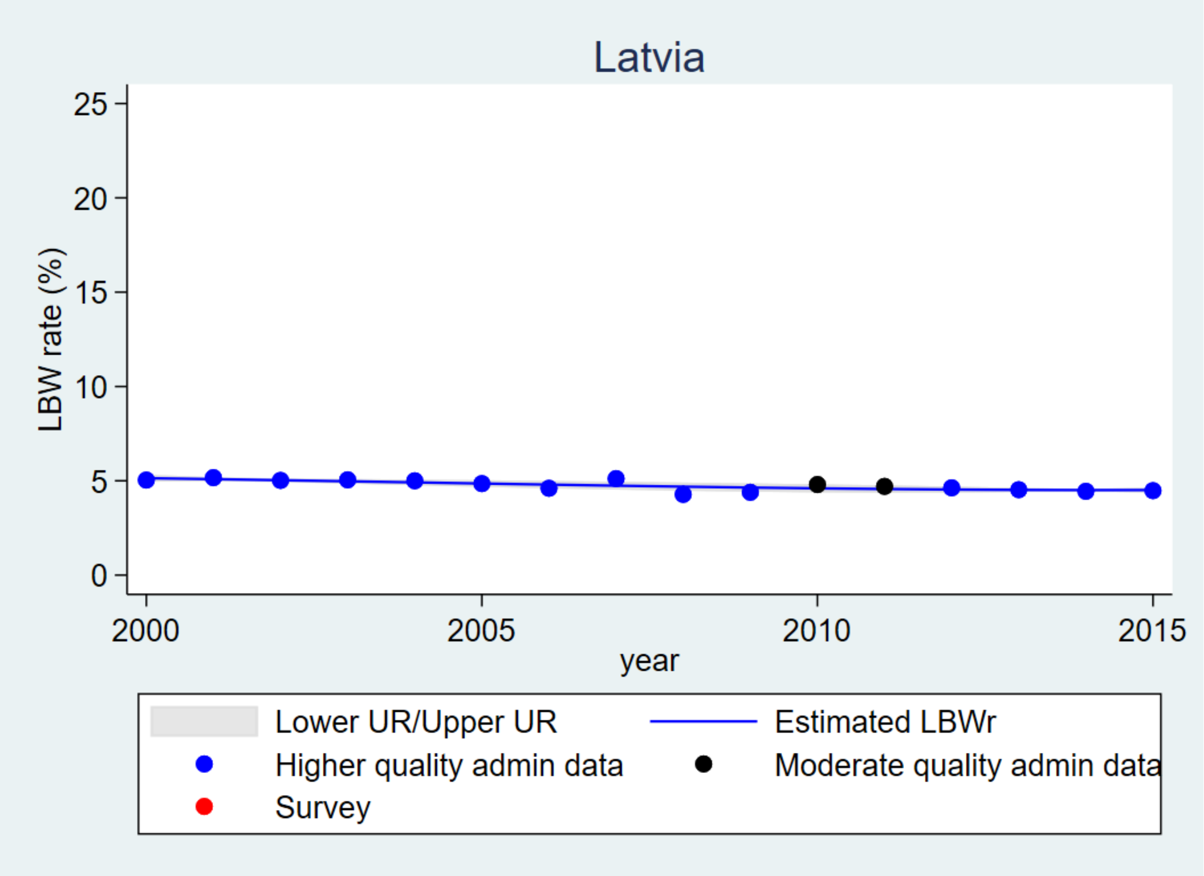
Latvia
LBW rate (%)
0
5
10
15
20
25
2000
2005
2010
2015
year
Lower UR/Upper UR
Estimated LBWr
Higher quality admin data
Moderate quality admin data
Survey

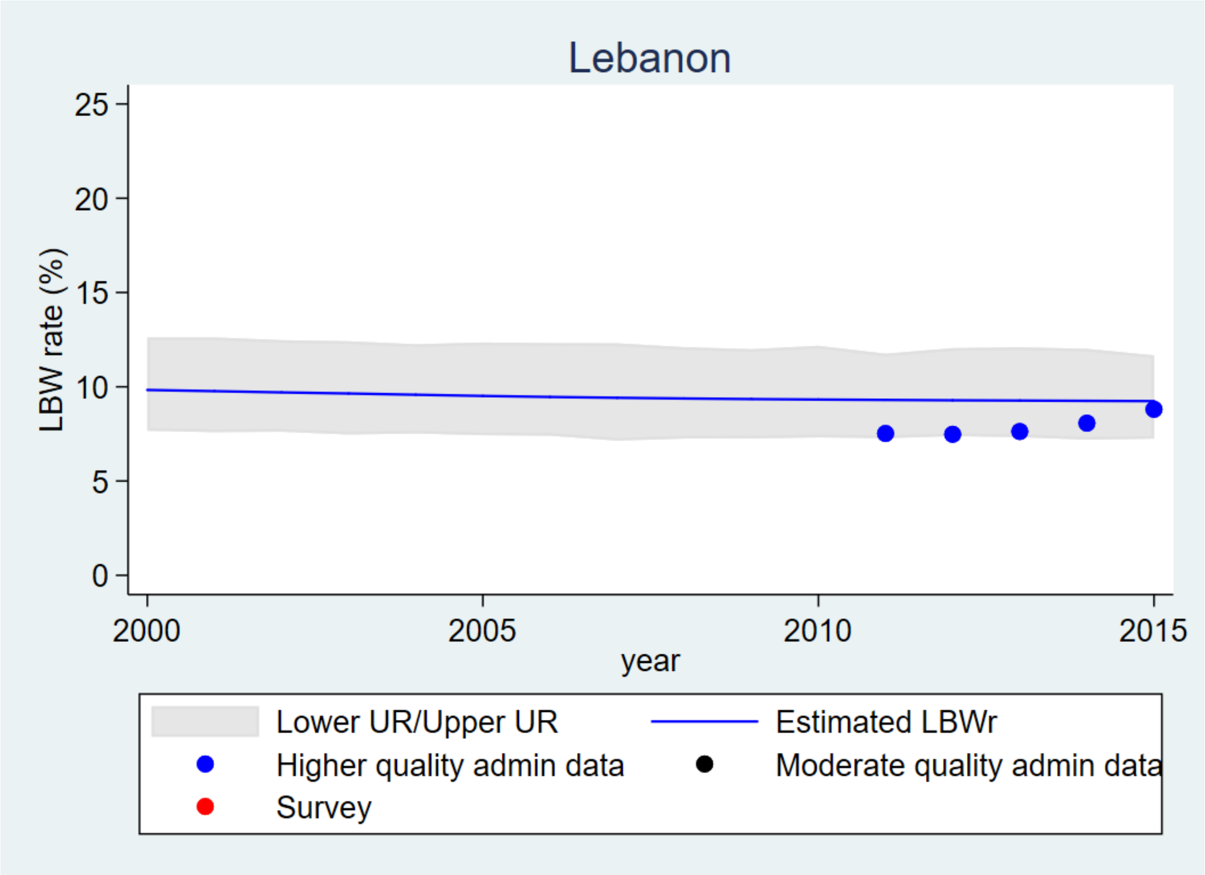
Lebanon
LBW rate (%)
25
20
15
10
5
0
2000
2005
2010
2015
year
Lower UR/Upper UR
Estimated LBWr
Higher quality admin data
Moderate quality admin data
Survey

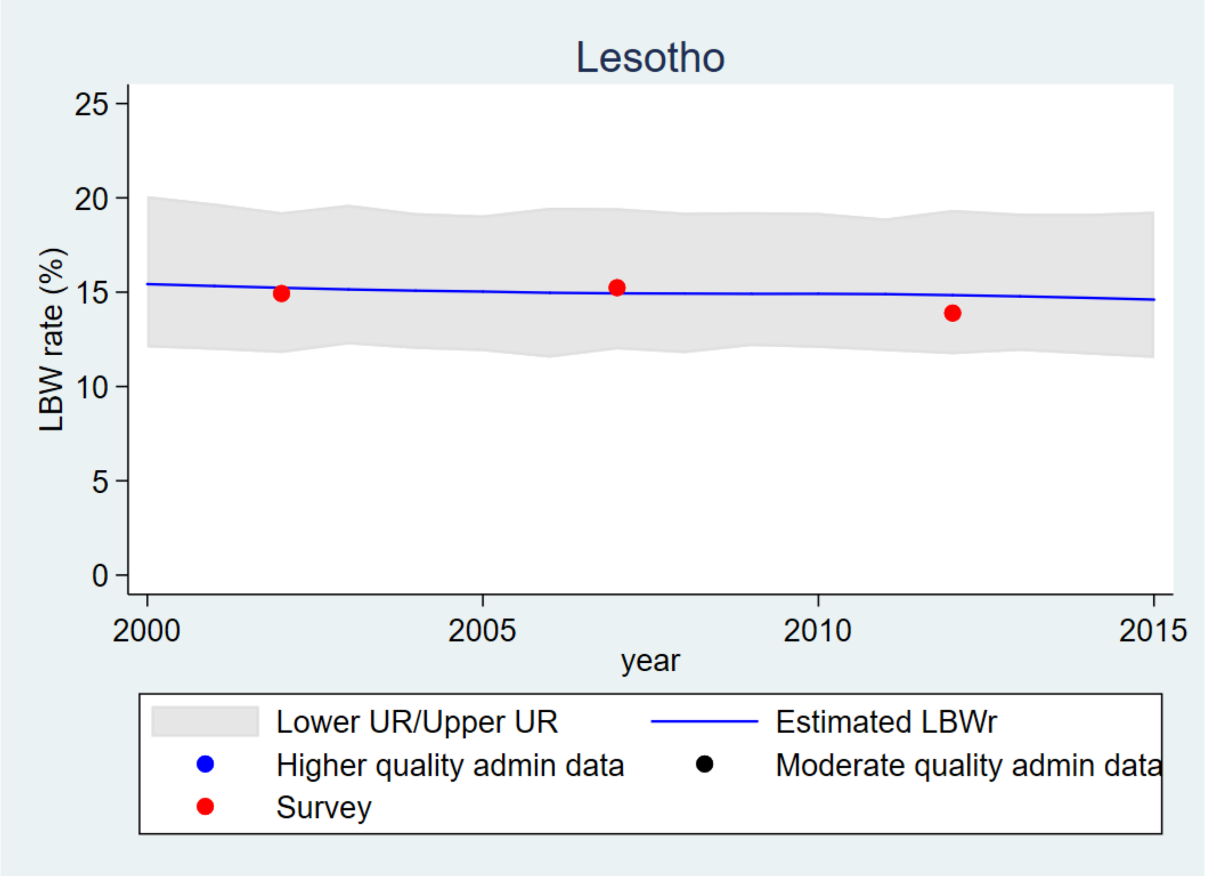
Lesotho
LBW rate (%)
25
20
15
10
5
0
2000
2005
2010
2015
year
Lower UR/Upper UR
Estimated LBWr
Higher quality admin data
Moderate quality admin data
Survey

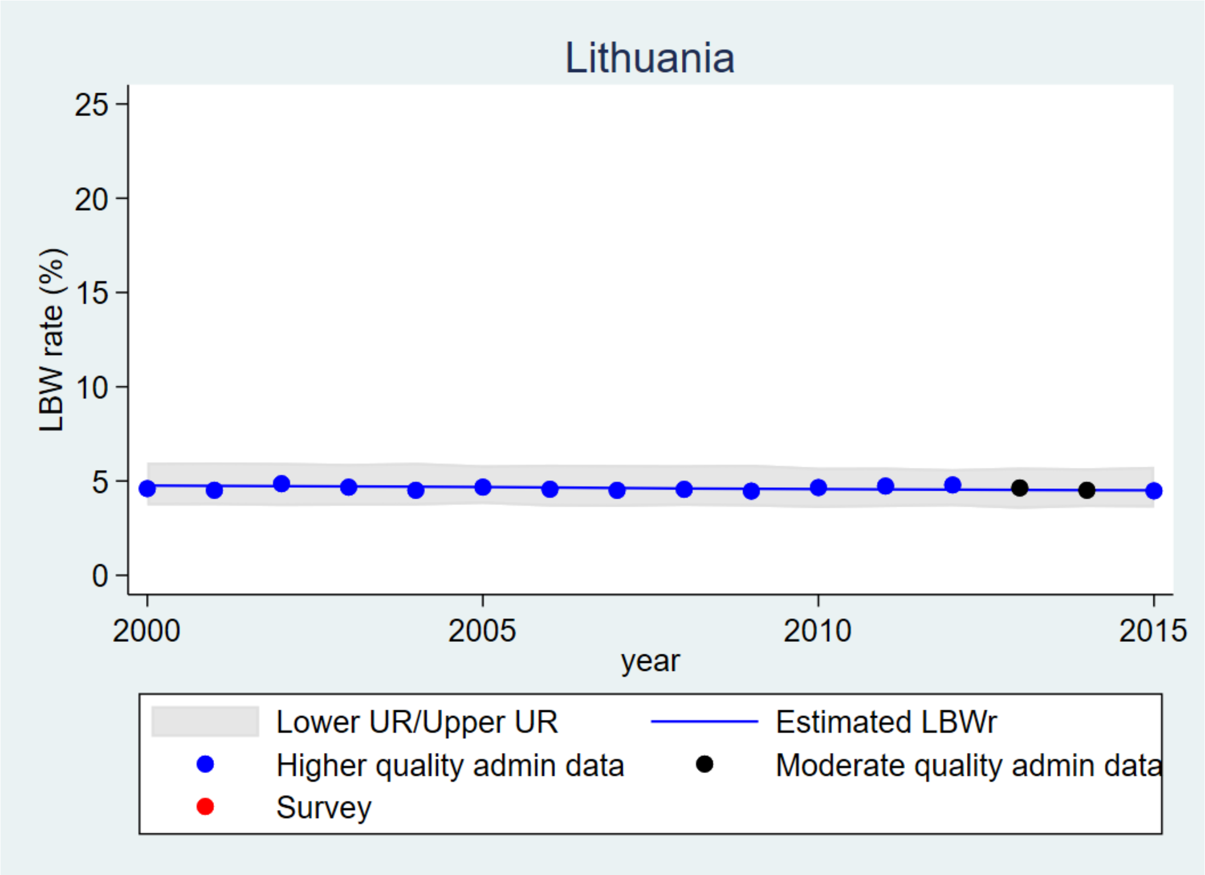
Lithuania
LBW rate (%)
25
20
15
10
5
0
2000
2005
2010
2015
year
Lower UR/Upper UR
Estimated LBWr
Higher quality admin data
Moderate quality admin data
Survey

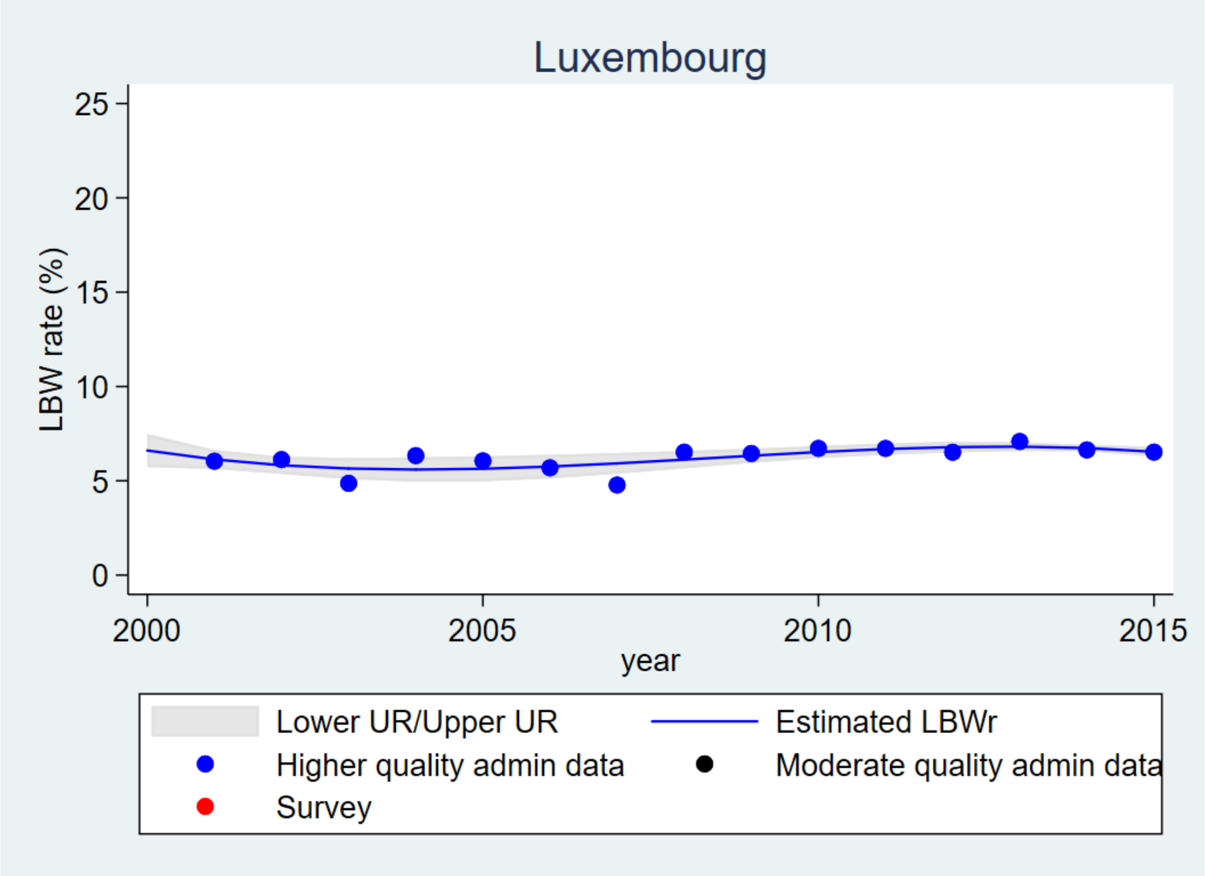
Luxembourg
LBW rate (%)
25
20
15
10
5
0
2000
2005
2010
2015
year
Lower UR/Upper UR
Estimated LBWr
Higher quality admin data
Moderate quality admin data
Survey

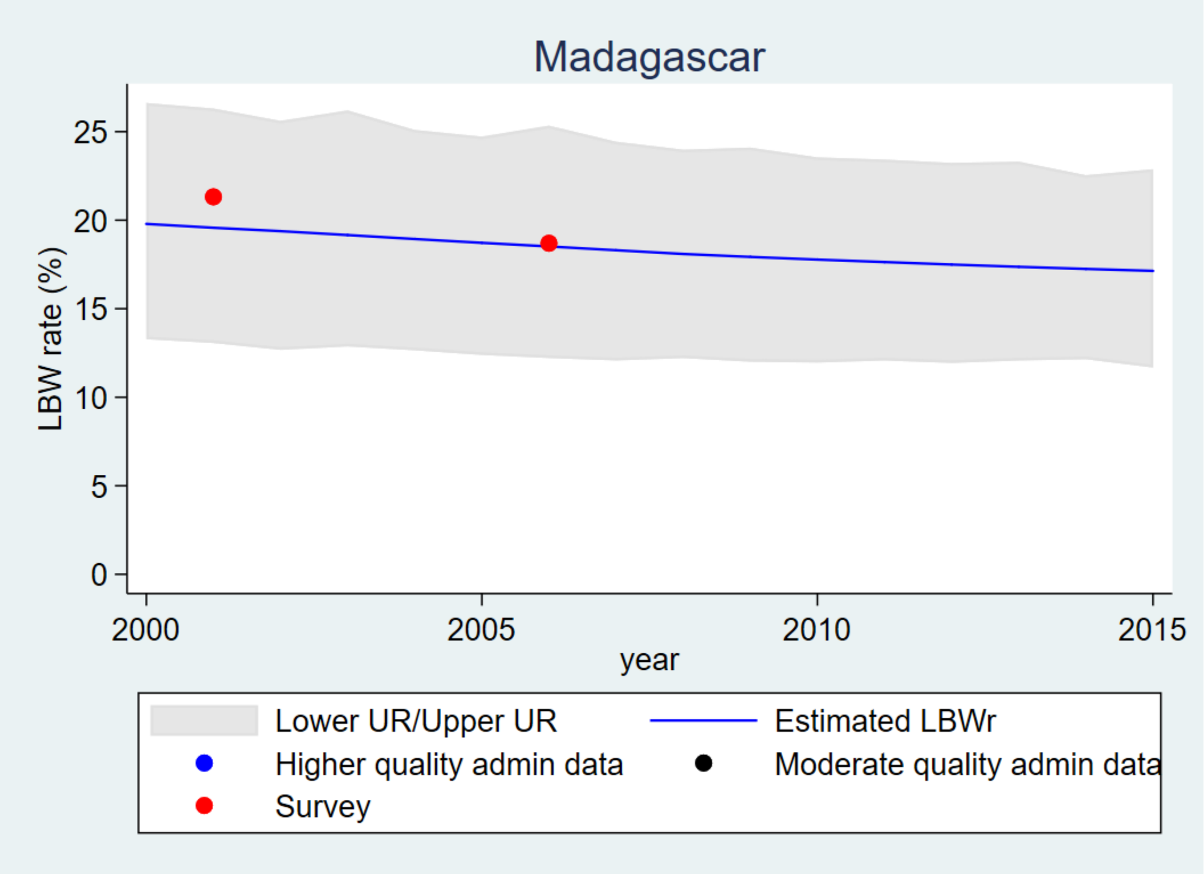

Madagascar
LBW rate (%)
0
5
10
15
20
25
2000
2005
2010
2015
year
Lower UR/Upper UR
Estimated LBWr
Higher quality admin data
Moderate quality admin data
Survey

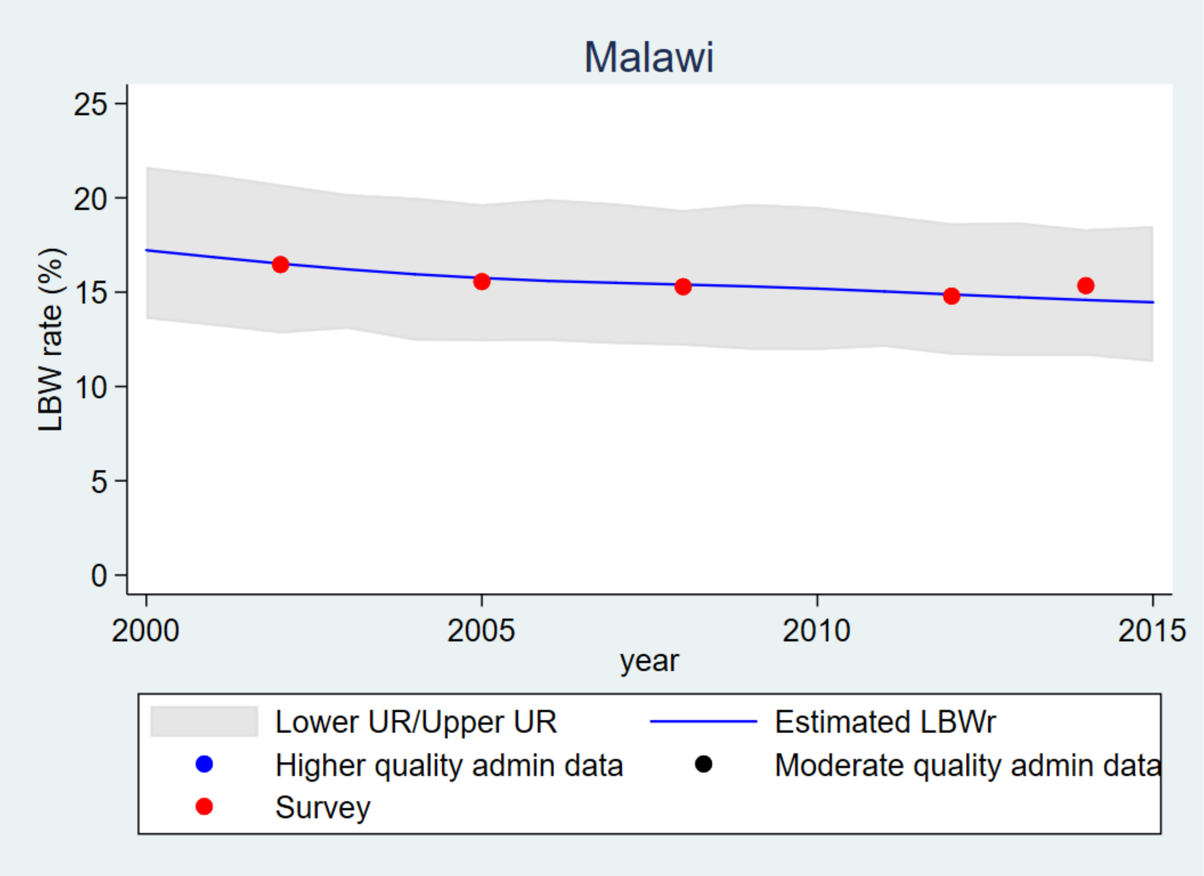

Malawi
LBW rate (%)
0
5
10
15
20
25
2000
2005
2010
2015
year
Lower UR/Upper UR
Estimated LBWr
Higher quality admin data
Moderate quality admin data
Survey

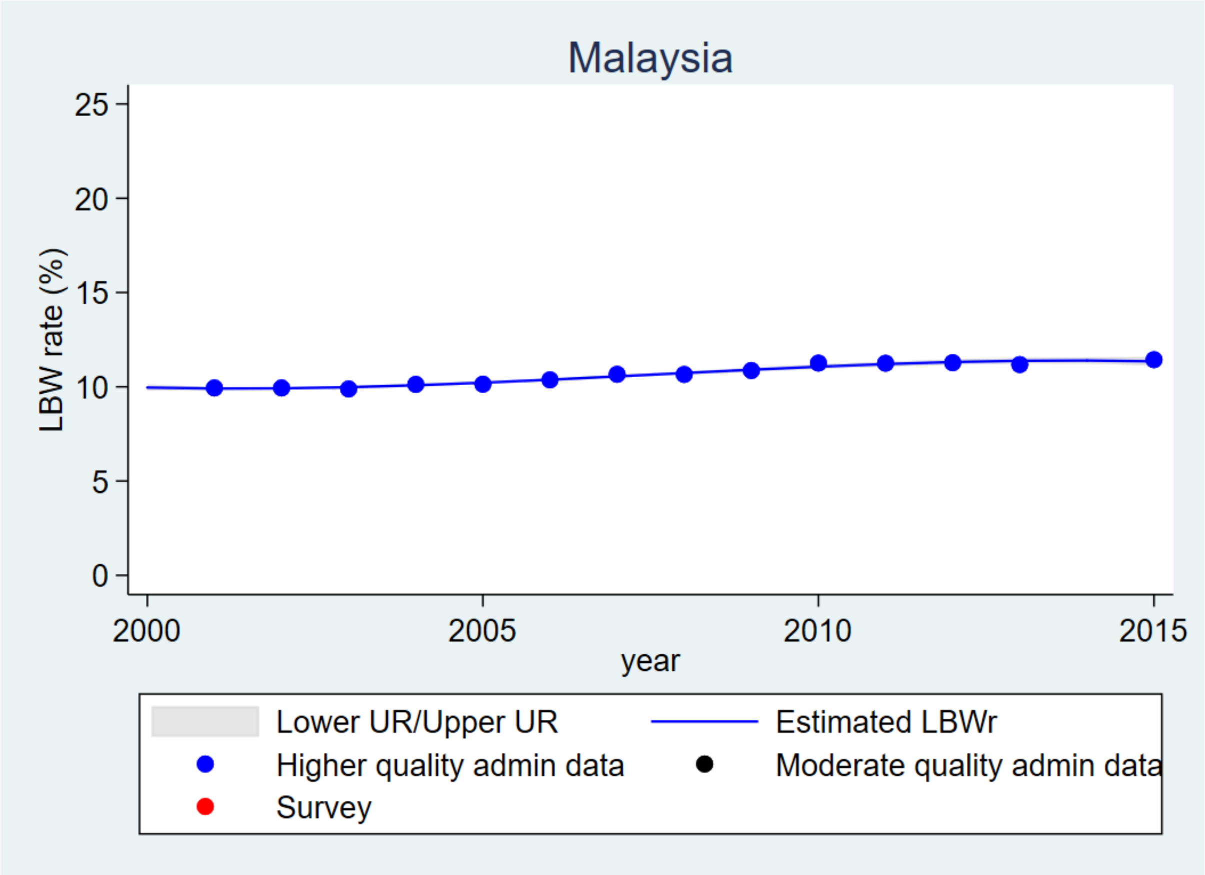
Malaysia
LBW rate (%)
25
20
15
10
5
0
2000
2005
2010
2015
year
Lower UR/Upper UR
Estimated LBWr
Higher quality admin data
Moderate quality admin data
Survey

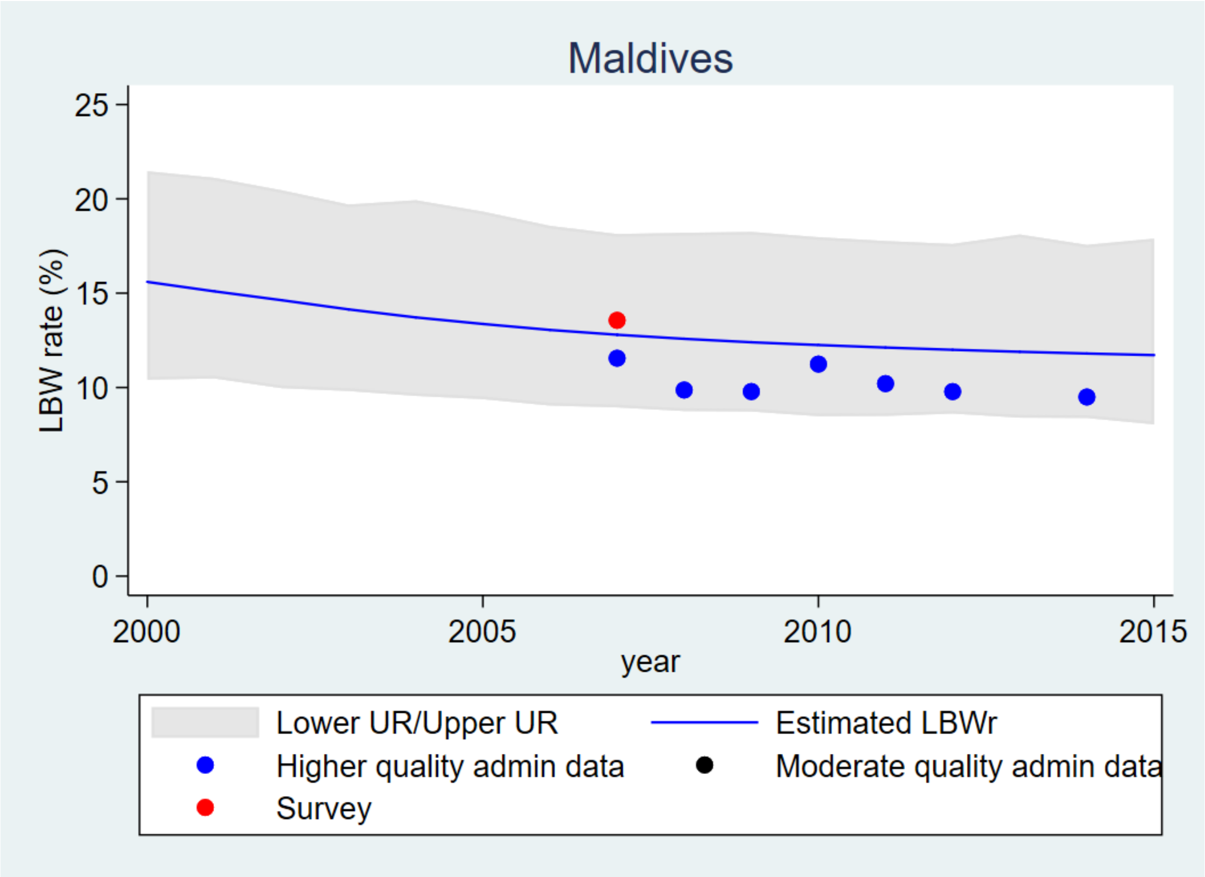
Maldives
LBW rate (%)
25
20
15
10
5
0
2000
2005
2010
2015
year
Lower UR/Upper UR
Estimated LBWr
Higher quality admin data
Moderate quality admin data
Survey

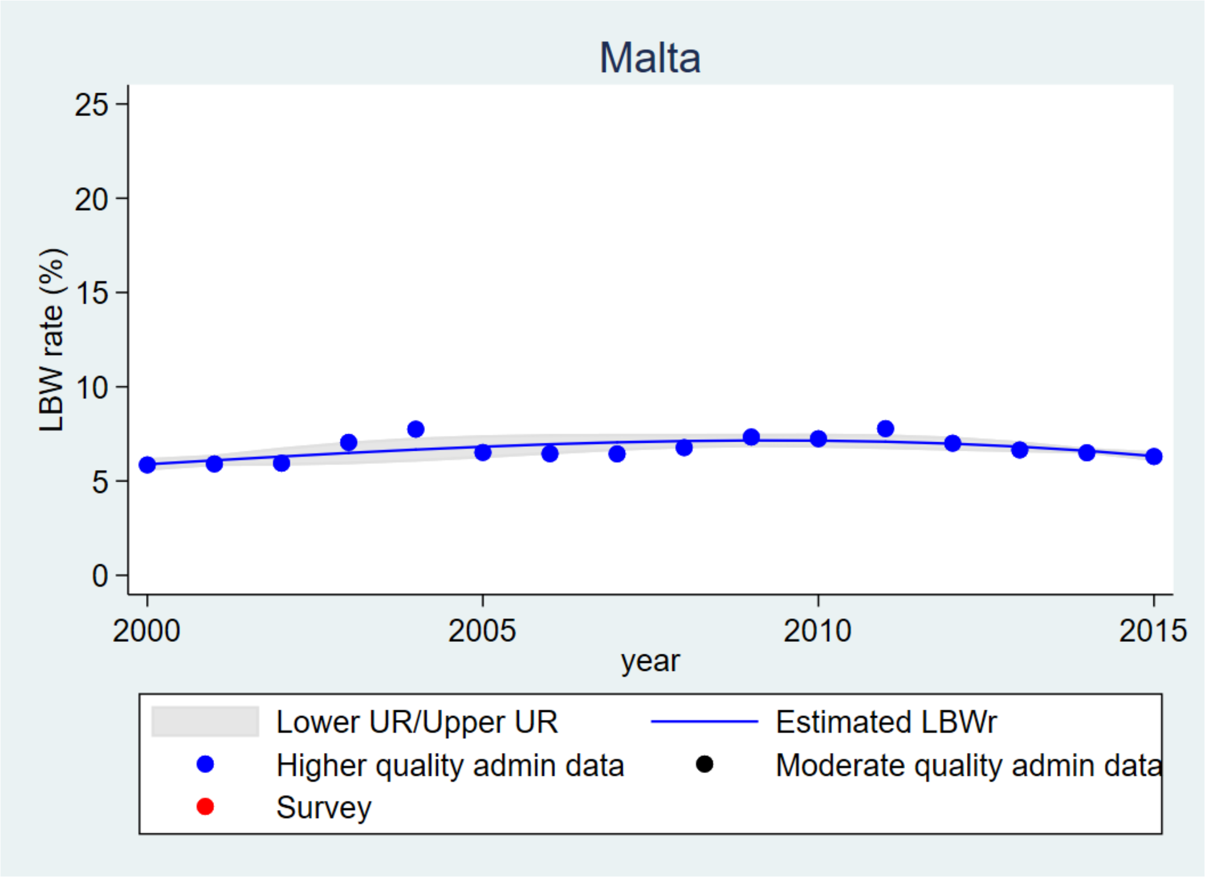
Malta
LBW rate (%)
25
20
15
10
5
0
2000
2005
2010
2015
year
Lower UR/Upper UR
Estimated LBWr
Higher quality admin data
Moderate quality admin data
Survey

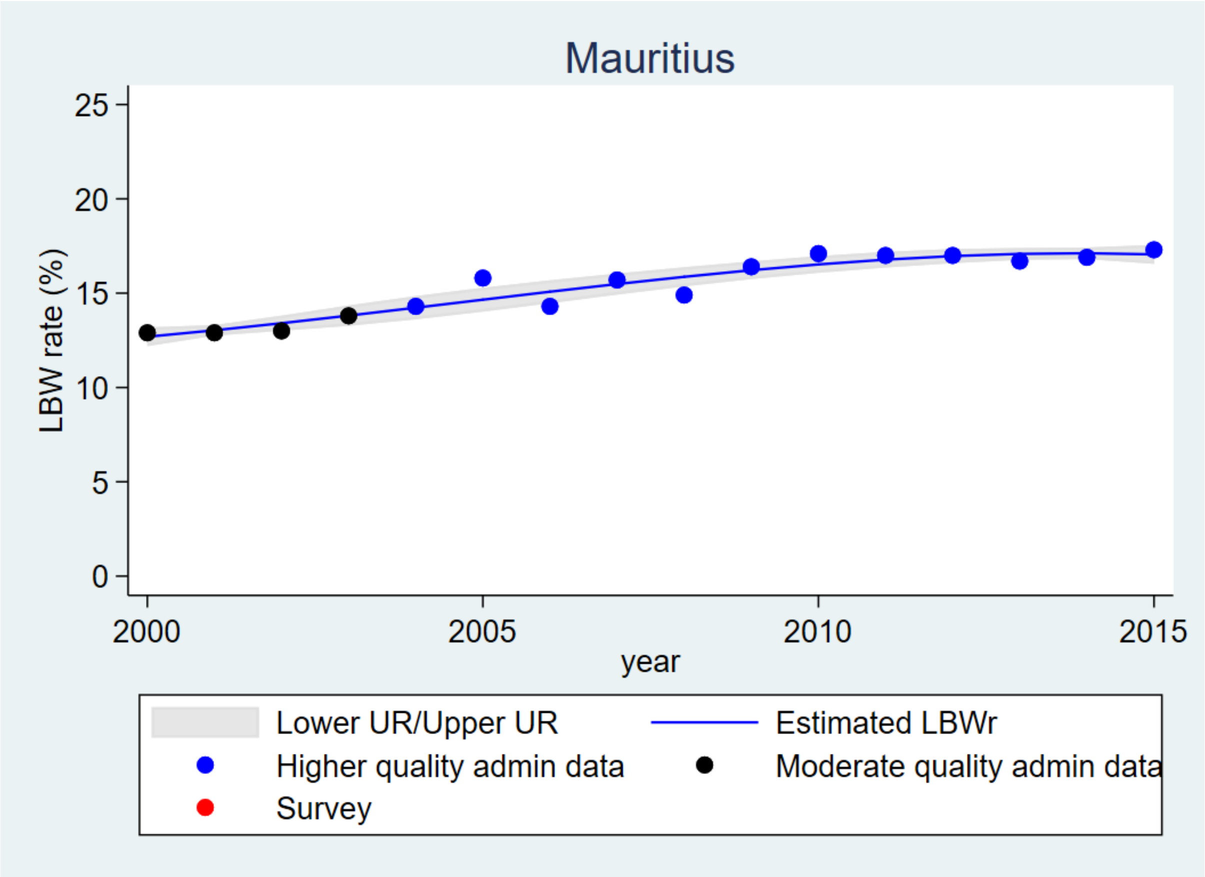
Mauritius
LBW rate (%)
25
20
15
10
5
0
2000
2005
2010
2015
year
Lower UR/Upper UR
Estimated LBWr
Higher quality admin data
Moderate quality admin data
Survey

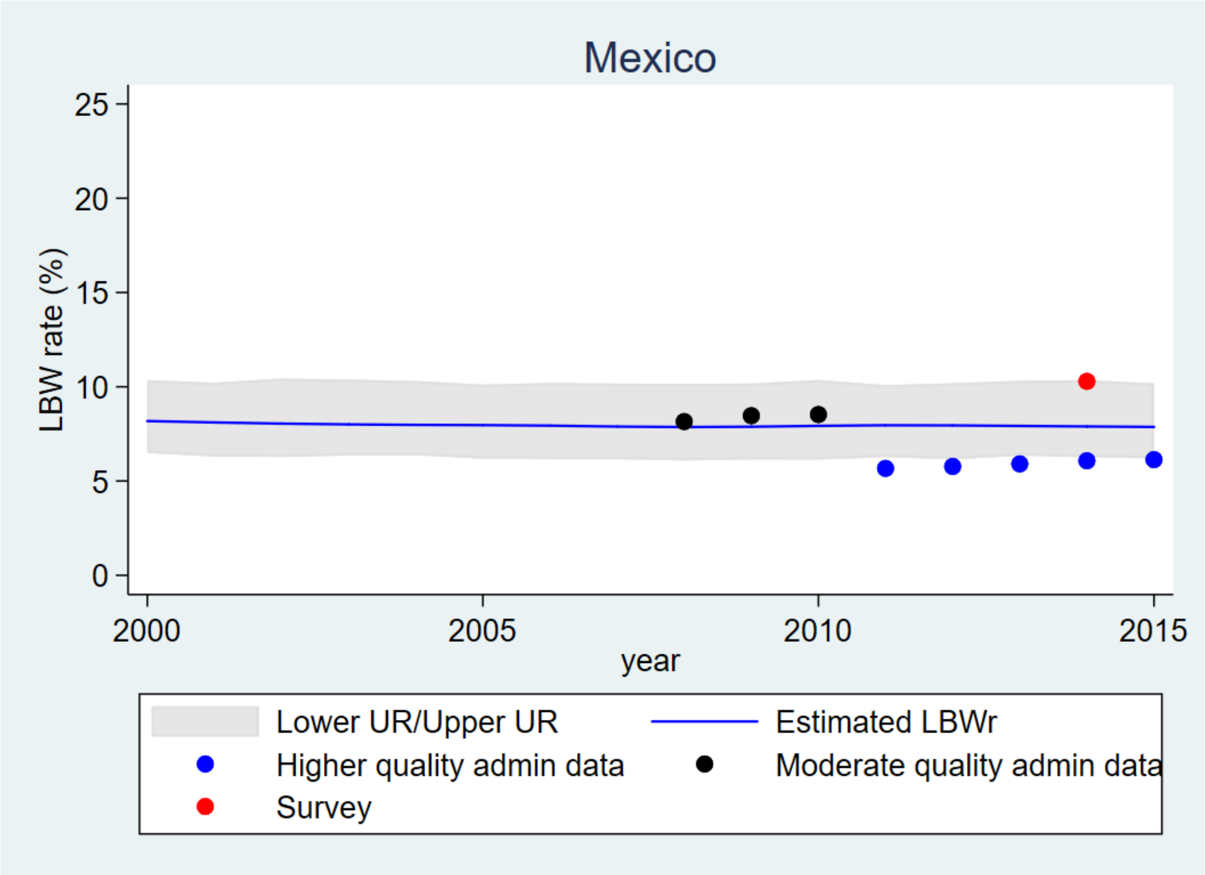
Mexico
LBW rate (%)
0
5
10
15
20
25
2000
2005
2010
2015
year
Lower UR/Upper UR
Estimated LBWr
Higher quality admin data
Moderate quality admin data
Survey

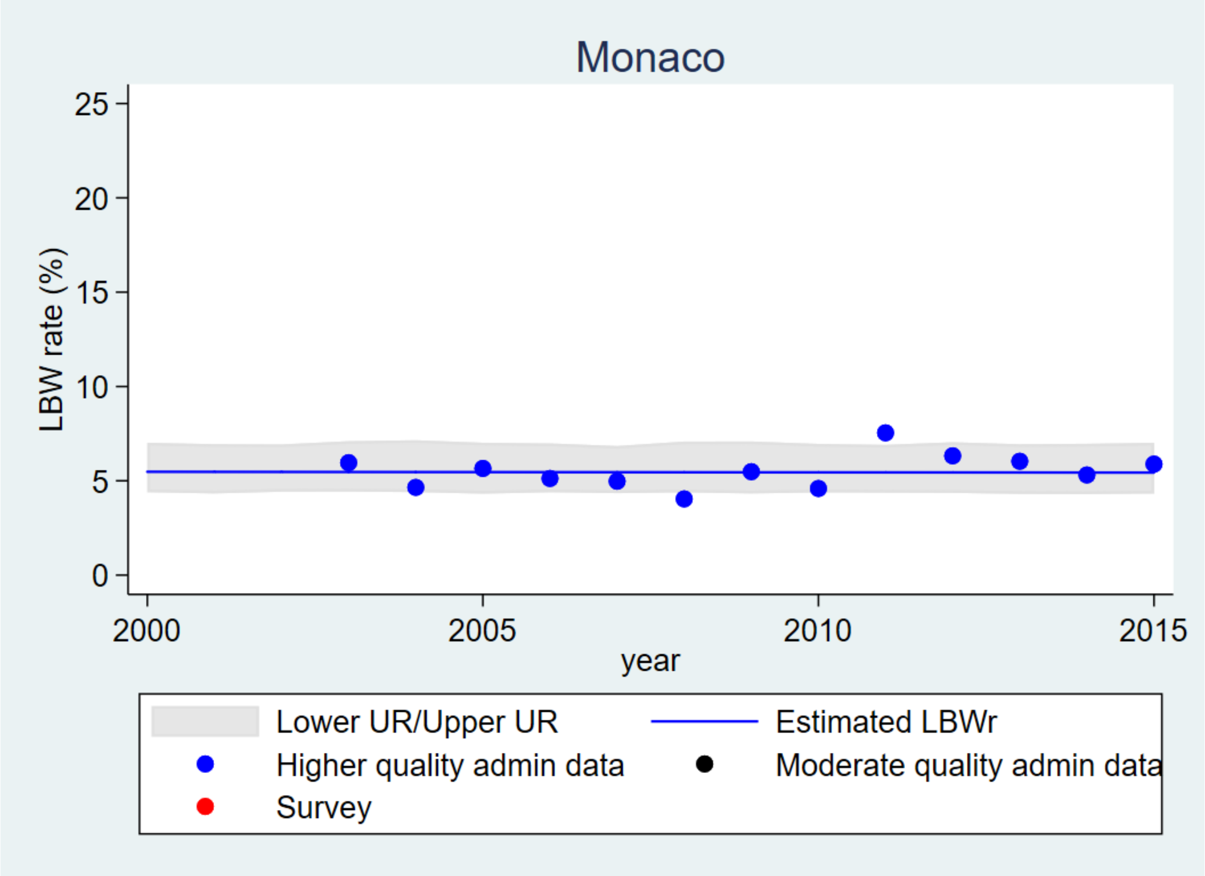
Monaco
LBW rate (%)
0
5
10
15
20
25
2000
2005
2010
2015
year
Lower UR/Upper UR
Estimated LBWr
Higher quality admin data
Moderate quality admin data
Survey

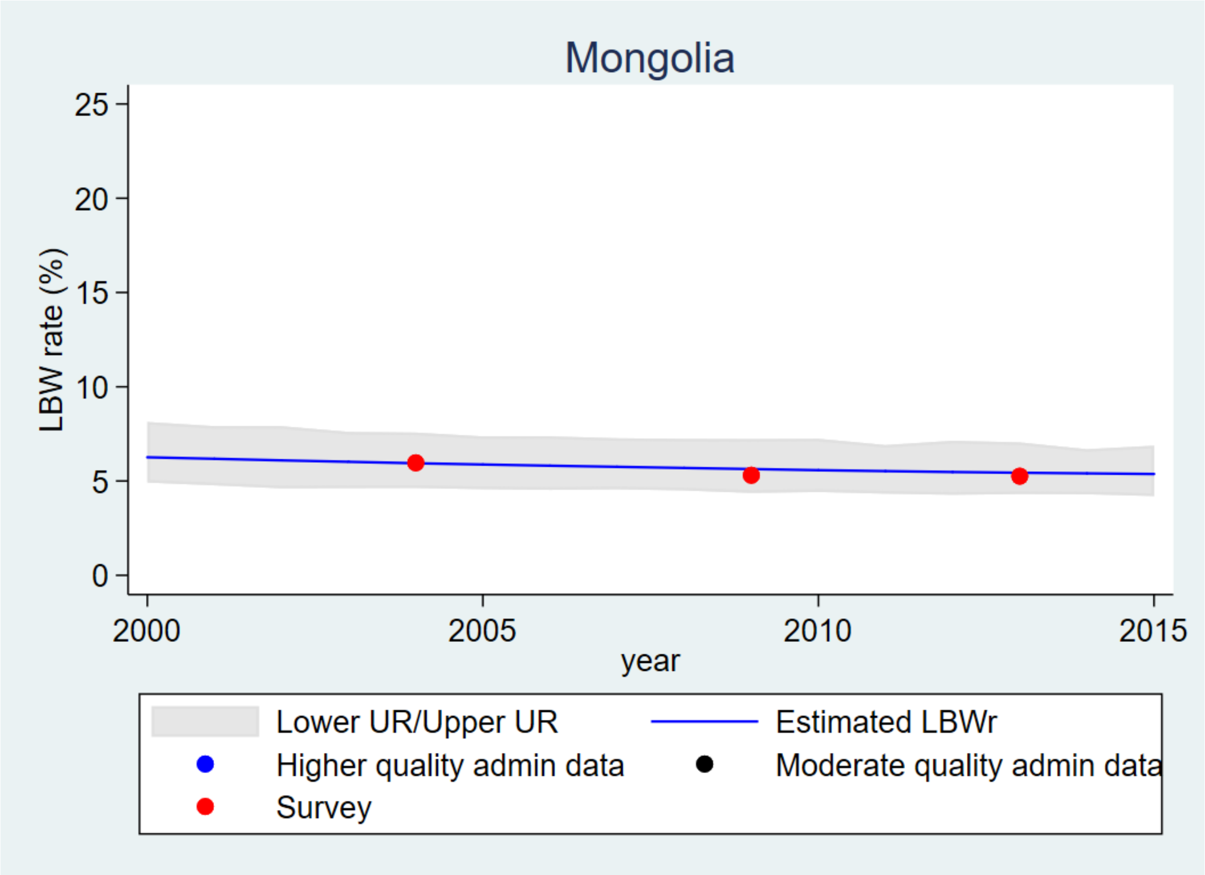
Mongolia
LBW rate (%)
25
20
15
10
5
0
2000
2005
2010
2015
year
Lower UR/Upper UR
Estimated LBWr
Higher quality admin data
Moderate quality admin data
Survey

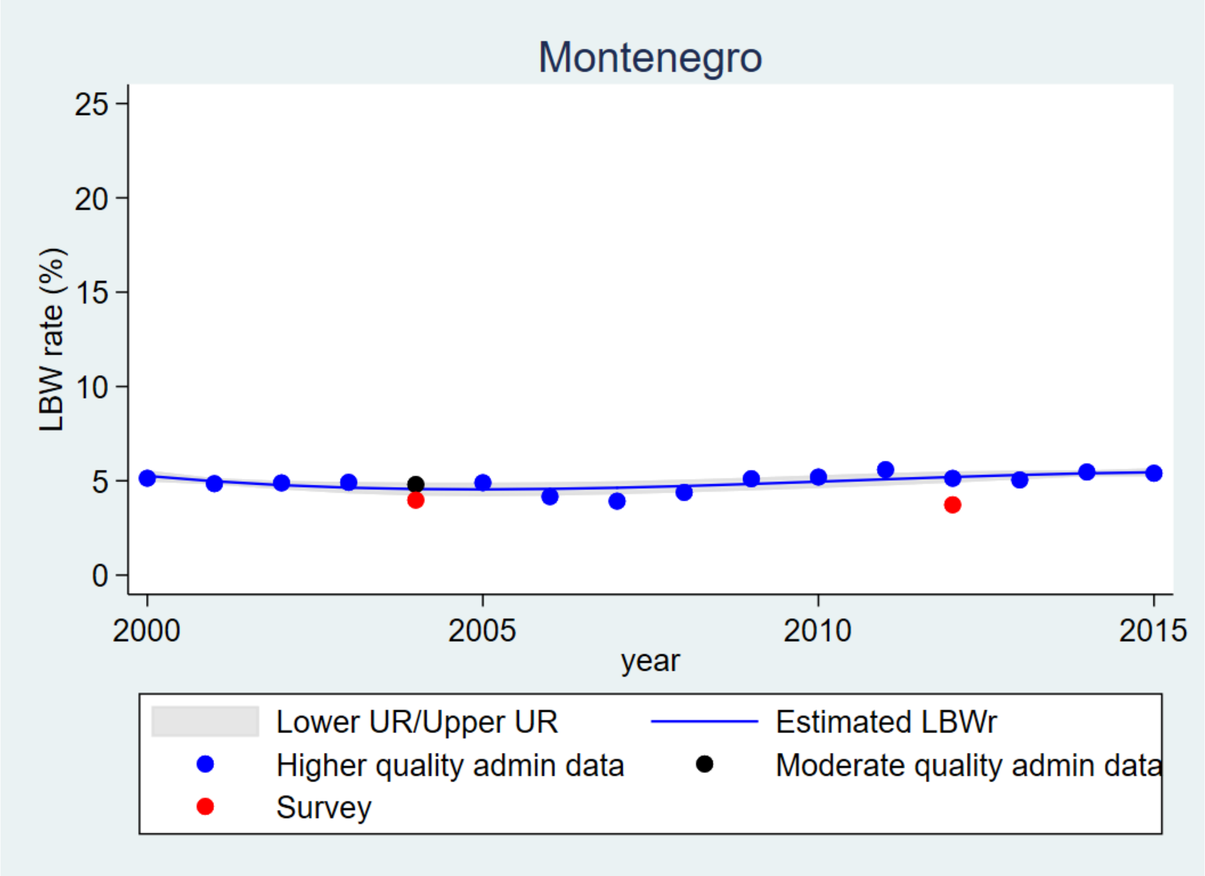
Montenegro
LBW rate (%)
25
20
15
10
5
0
2000
2005
2010
2015
year
Lower UR/Upper UR
Estimated LBWr
Higher quality admin data
Moderate quality admin data
Survey

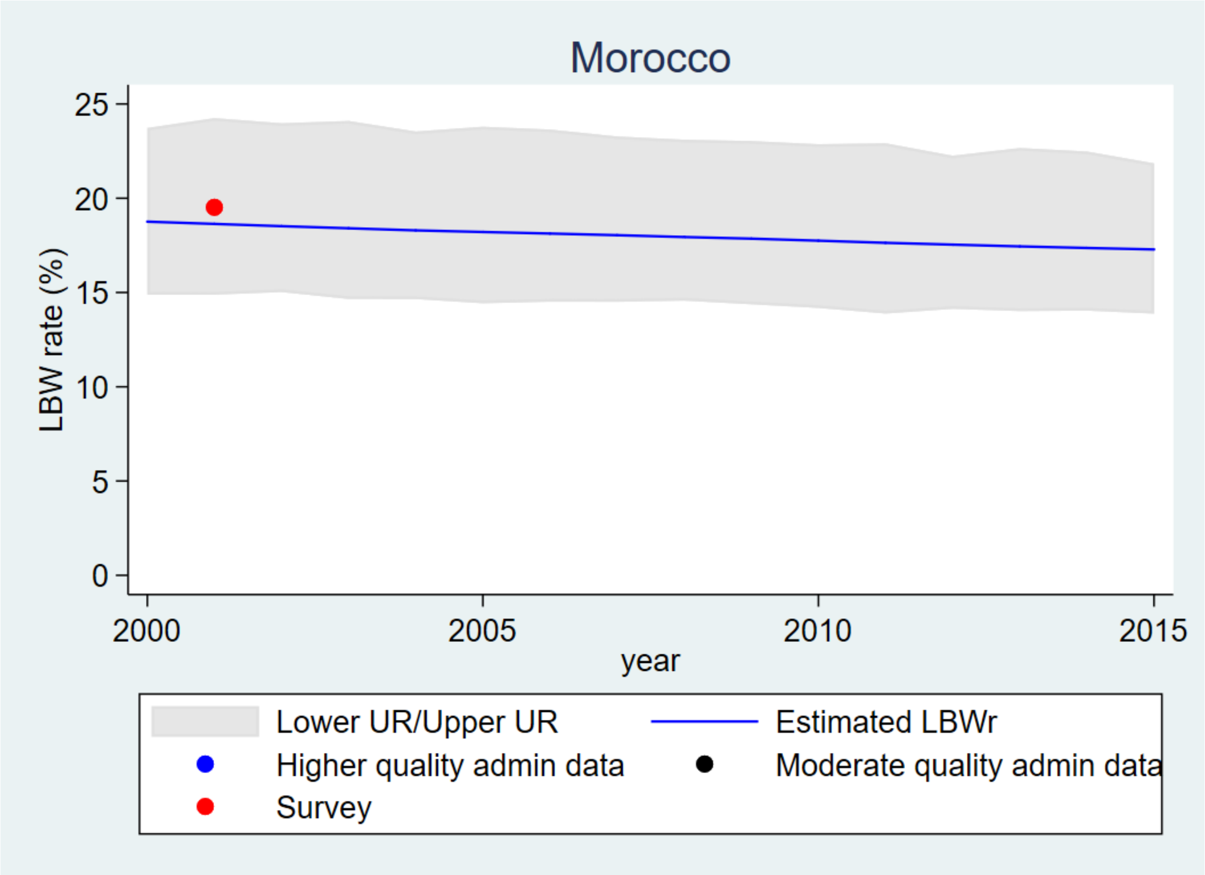
Morocco
LBW rate (%)
25
20
15
10
5
0
2000
2005
2010
2015
year
Lower UR/Upper UR
Estimated LBWr
Higher quality admin data
Moderate quality admin data
Survey

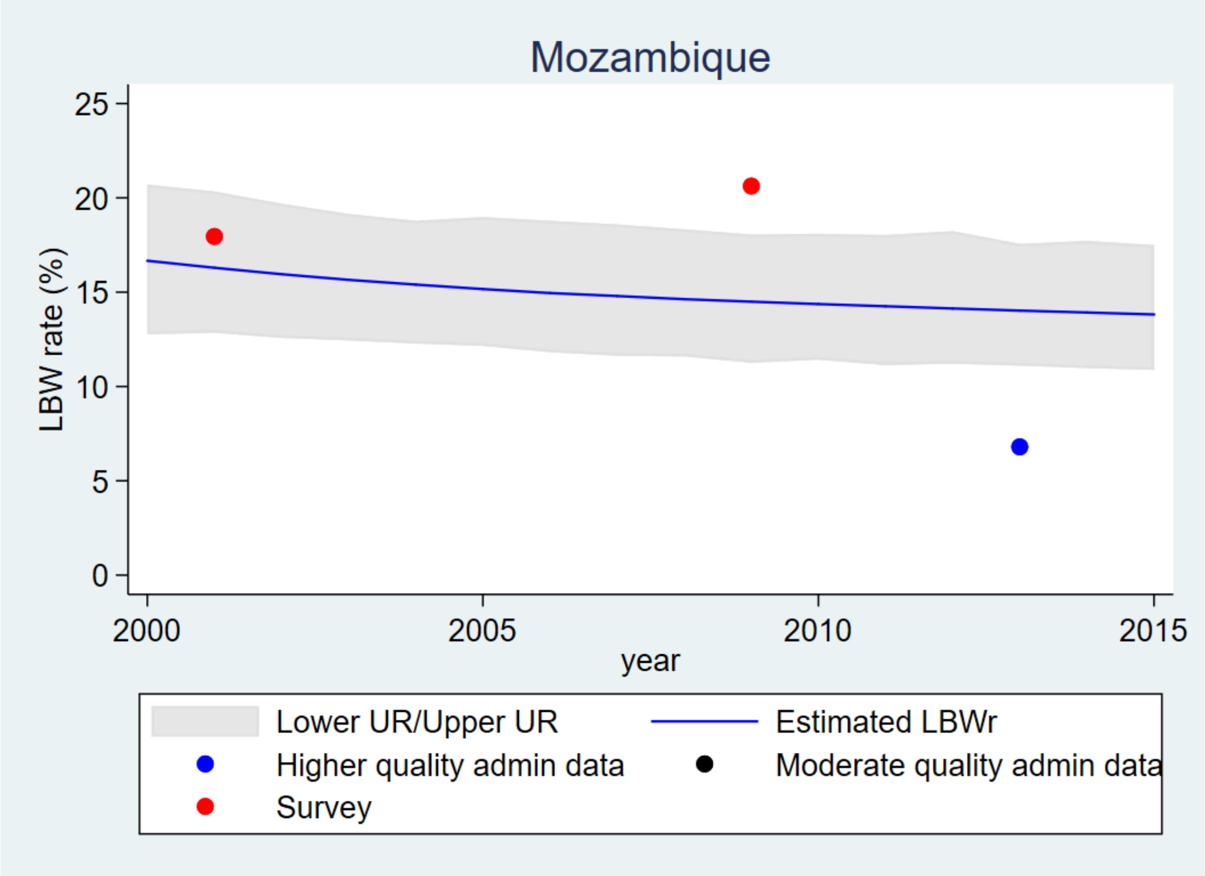
Mozambique
LBW rate (%)
25
20
15
10
5
0
2000
2005
2010
2015
year
Lower UR/Upper UR
Estimated LBWr
Higher quality admin data
Moderate quality admin data
Survey

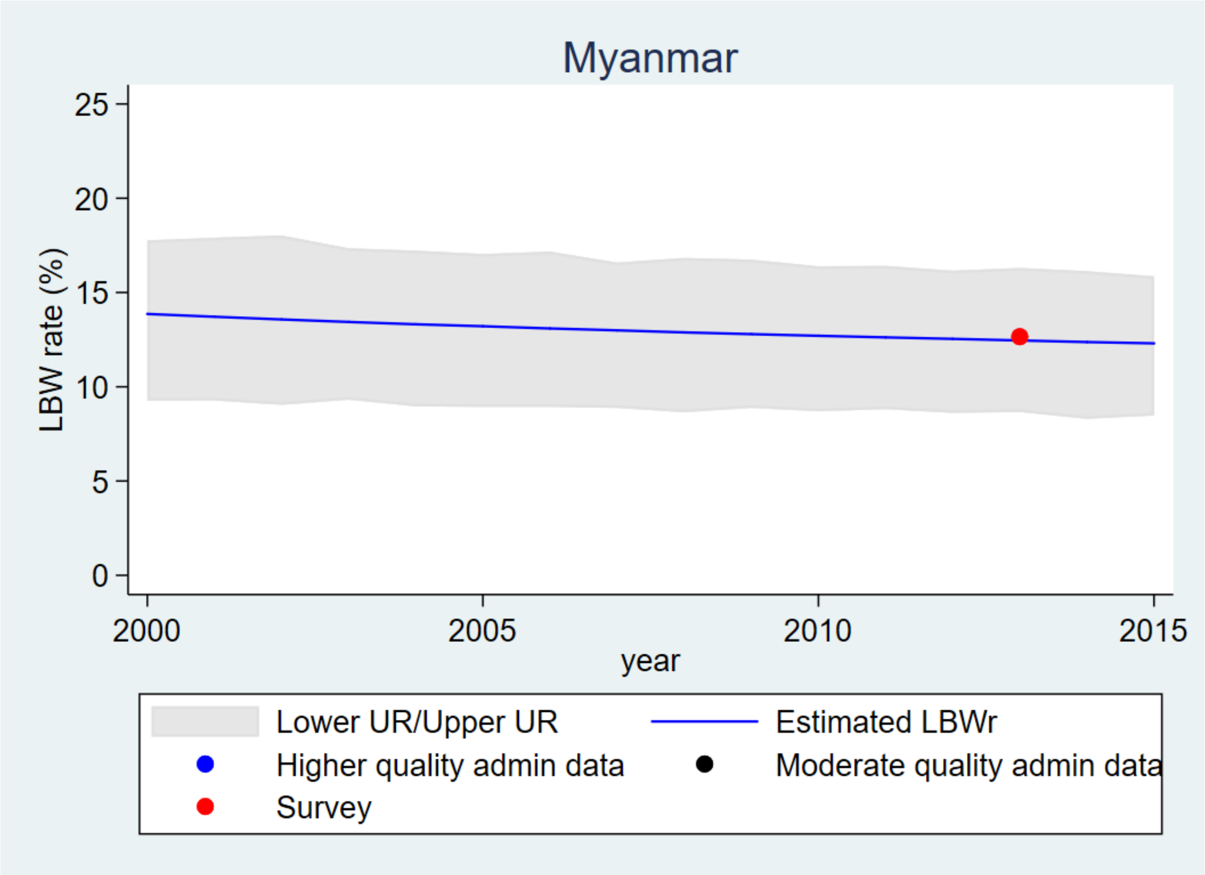
Myanmar
LBW rate (%)
25
20
15
10
5
0
2000
2005
2010
2015
year
Lower UR/Upper UR
Estimated LBWr
Higher quality admin data
Moderate quality admin data
Survey

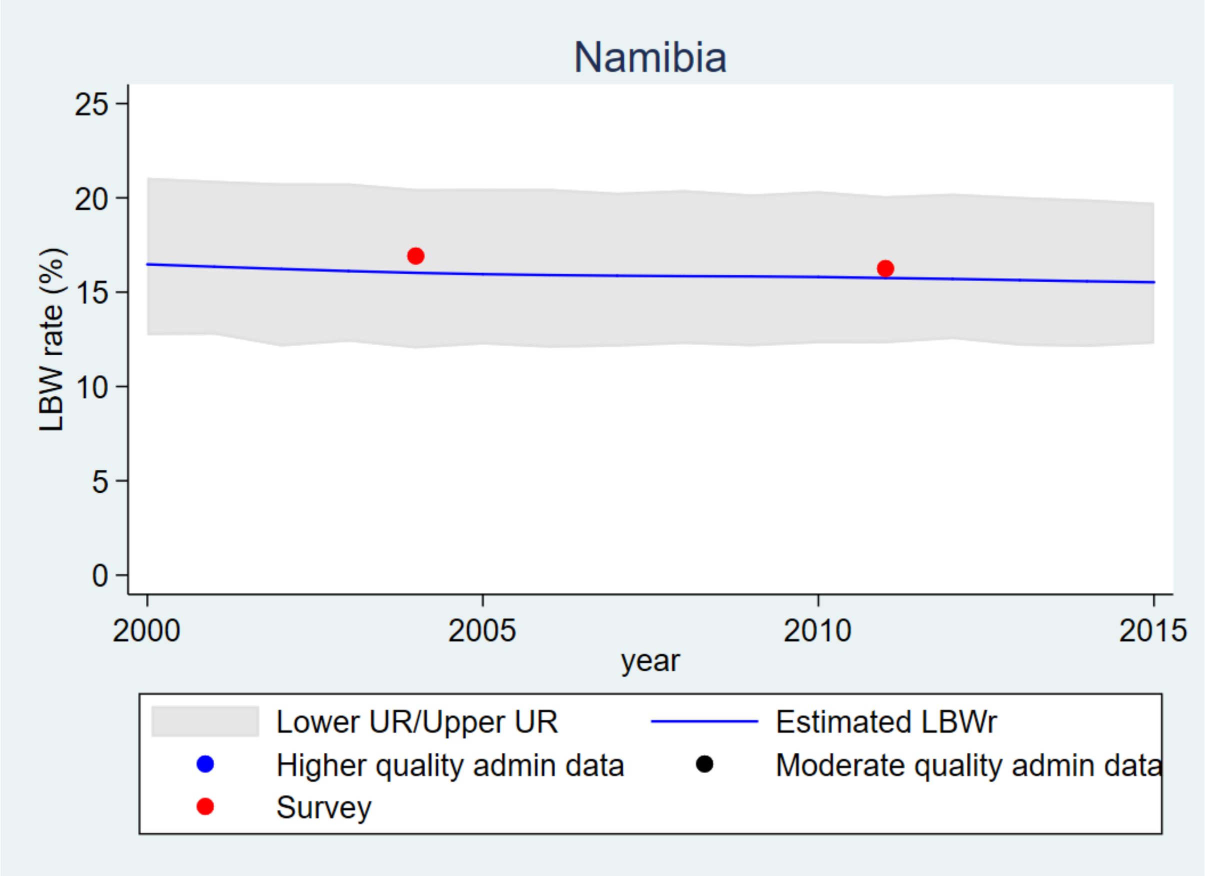
Namibia
LBW rate (%)
25
20
15
10
5
0
2000
2005
2010
2015
year
Lower UR/Upper UR
Estimated LBWr
Higher quality admin data
Moderate quality admin data
Survey

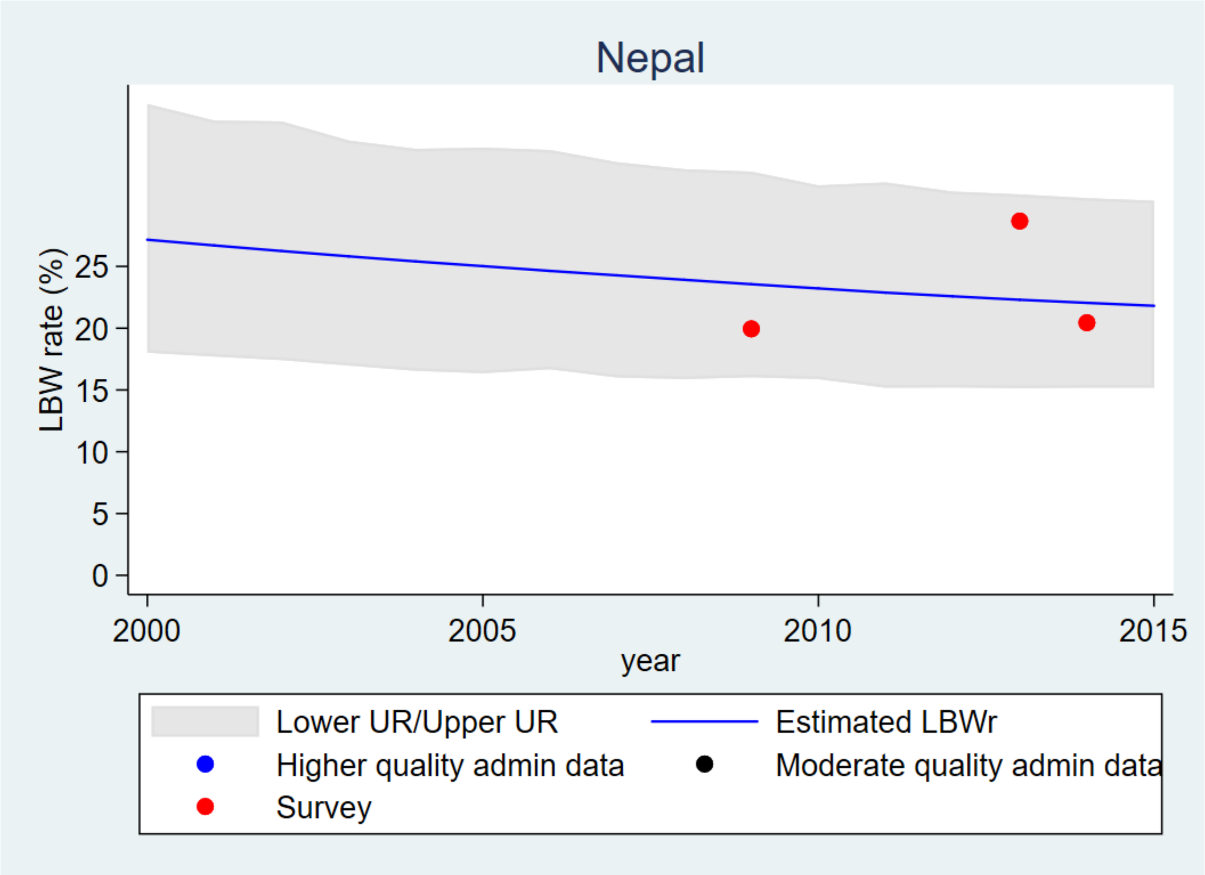
Nepal
LBW rate (%)
25
20
15
10
5
0
2000
2005
2010
2015
year
Lower UR/Upper UR
Estimated LBWr
Higher quality admin data
Moderate quality admin data
Survey

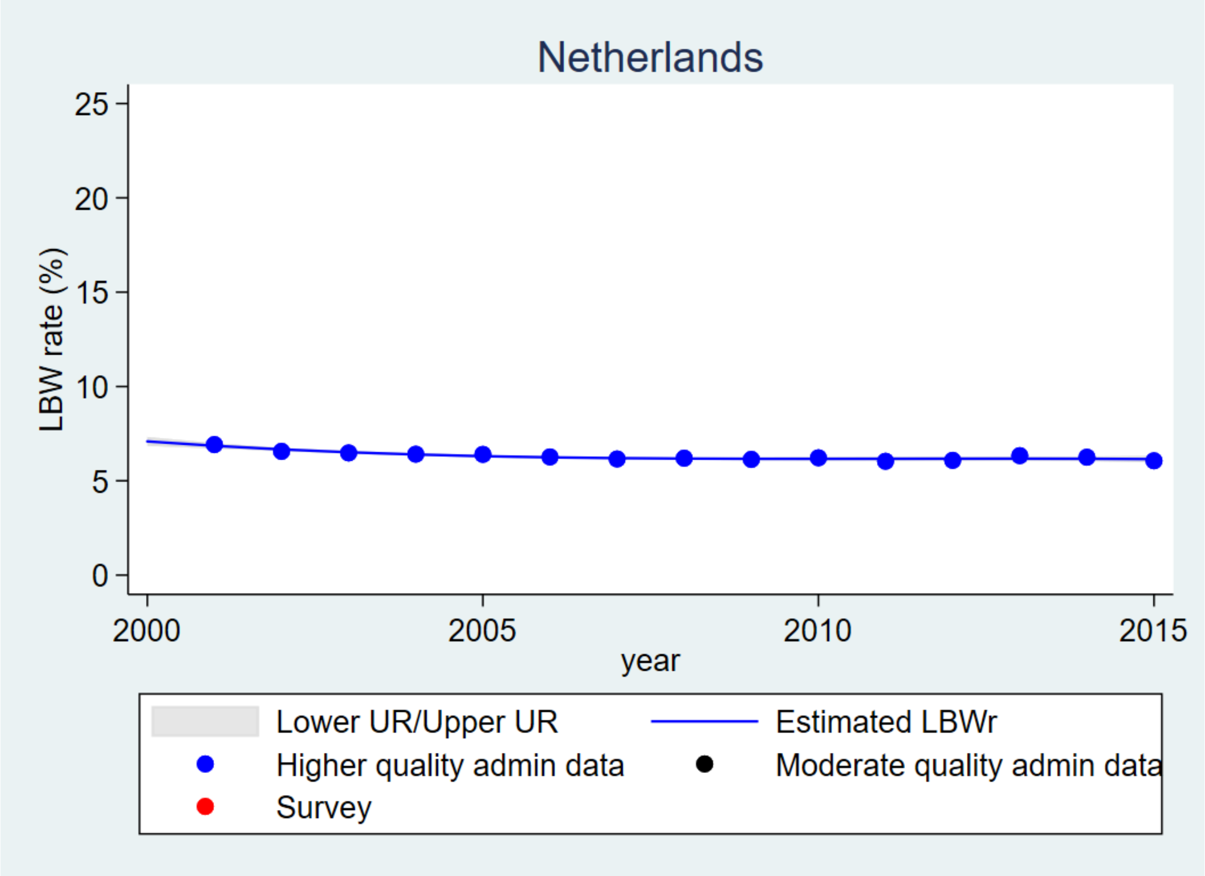
Netherlands
LBW rate (%)
25
20
15
10
5
0
2000
2005
2010
2015
year
Lower UR/Upper UR
Estimated LBWr
Higher quality admin data
Moderate quality admin data
Survey

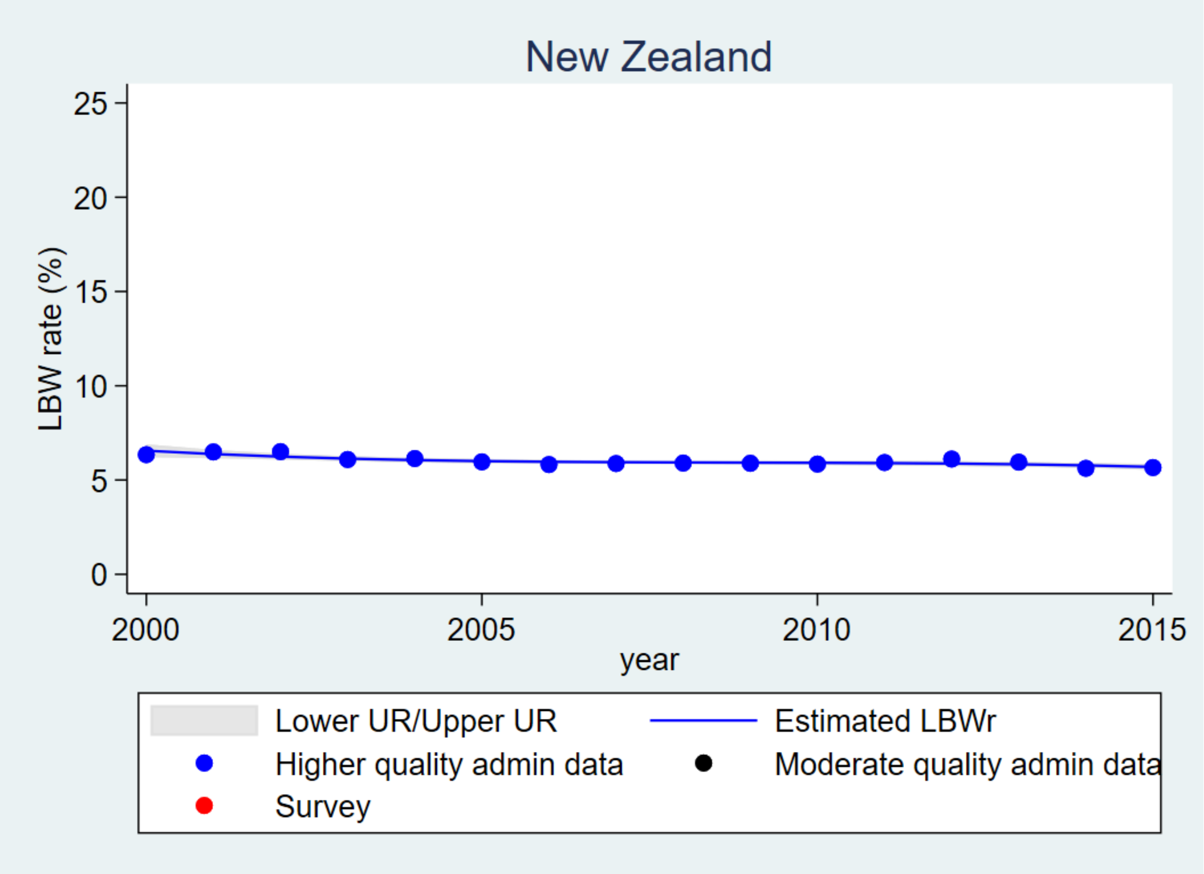
New Zealand
LBW rate (%)
25
20
15
10
5
0
2000
2005
2010
2015
year
Lower UR/Upper UR
Estimated LBWr
Higher quality admin data
Moderate quality admin data
Survey

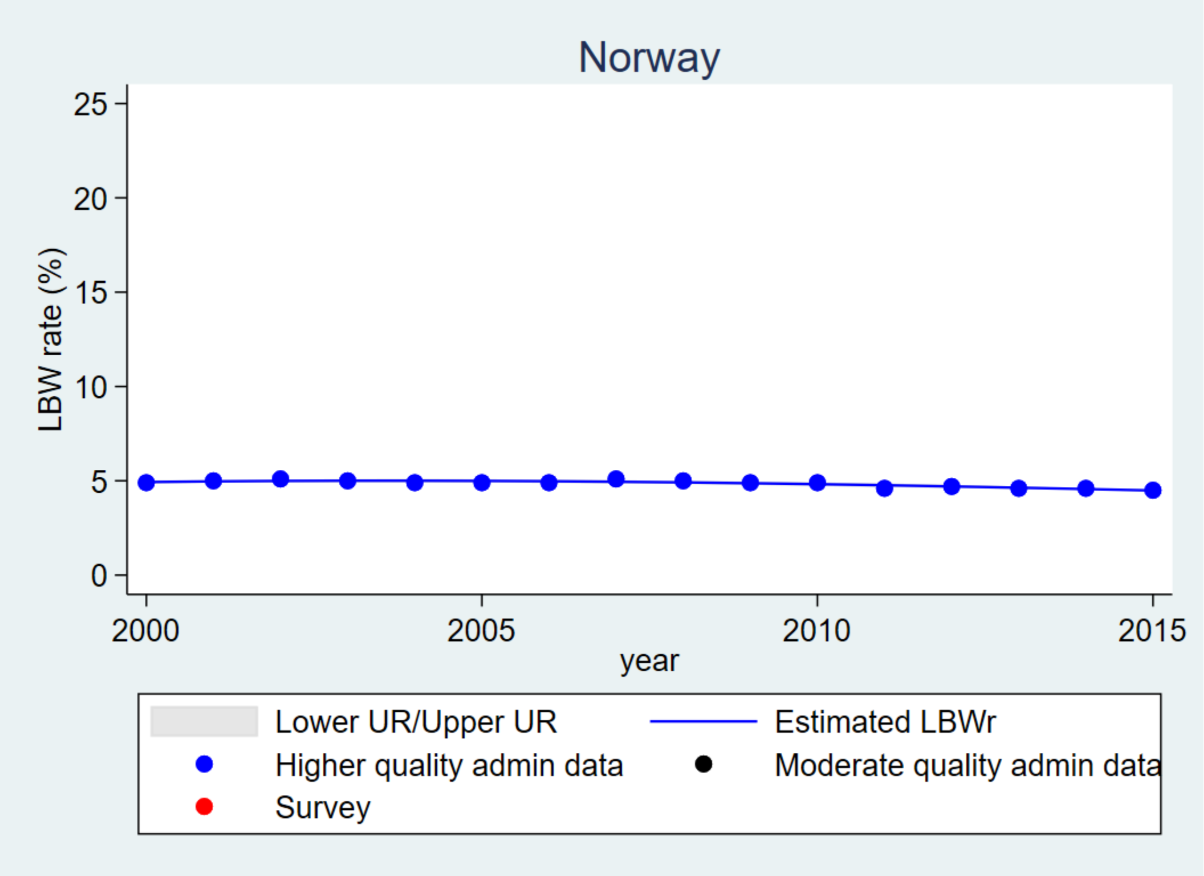
Norway
LBW rate (%)
25
20
15
10
5
0
2000
2005
2010
2015
year
Lower UR/Upper UR
Estimated LBWr
Higher quality admin data
Moderate quality admin data
Survey

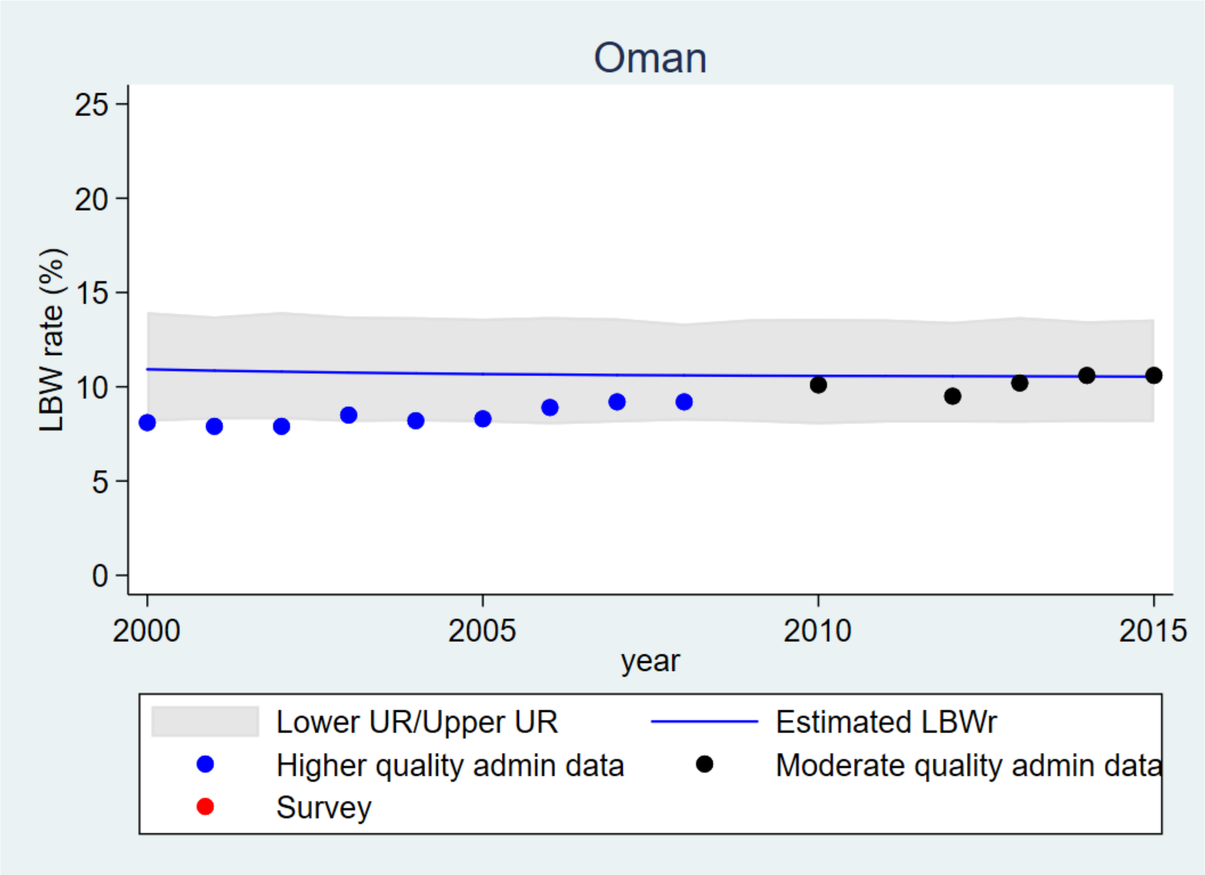
Oman
25
20
15
10
5
0
LBW rate (%)
2000
2005
2010
2015
year
Lower UR/Upper UR
Estimated LBWr
Higher quality admin data
Moderate quality admin data
Survey

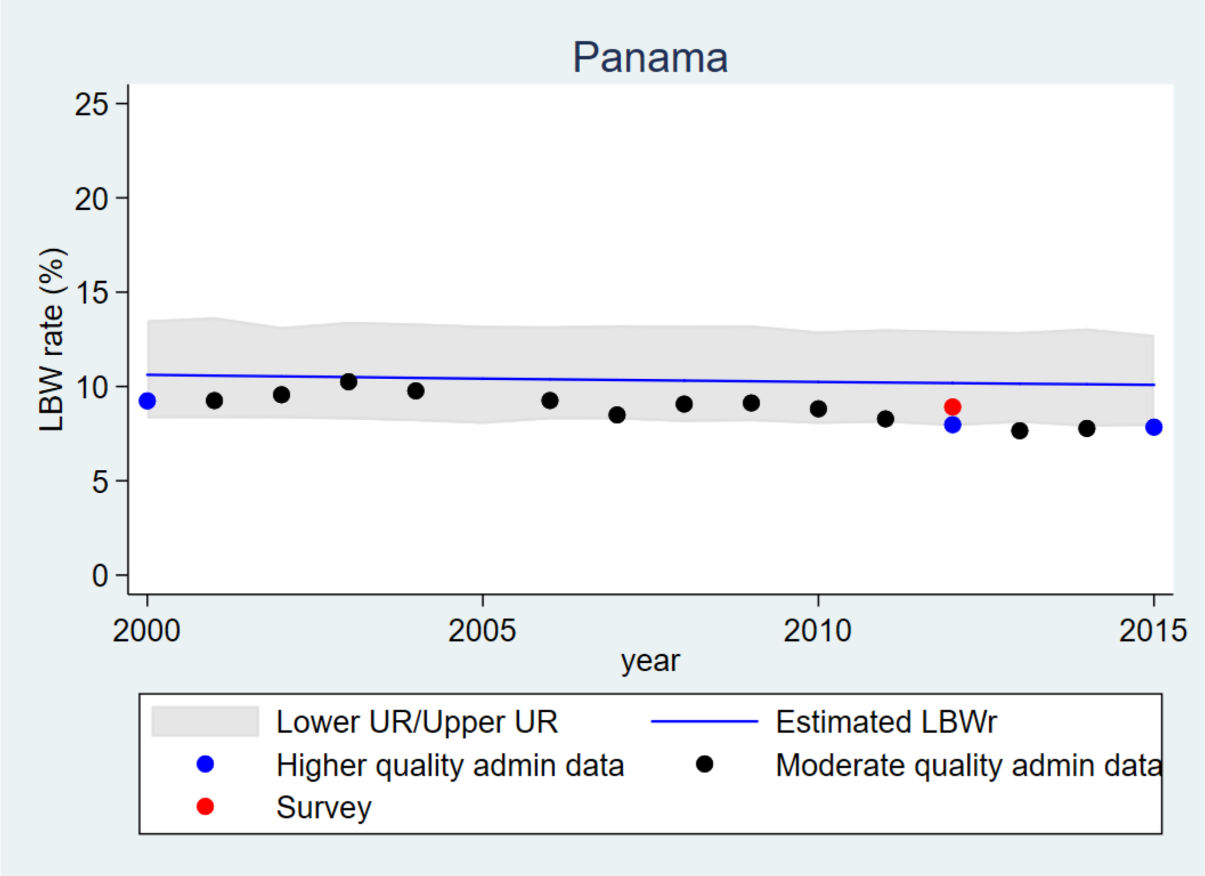
Panama
25
20
15
10
5
0
LBW rate (%)
2000
2005
2010
2015
year
Lower UR/Upper UR
Estimated LBWr
Higher quality admin data
Moderate quality admin data
Survey

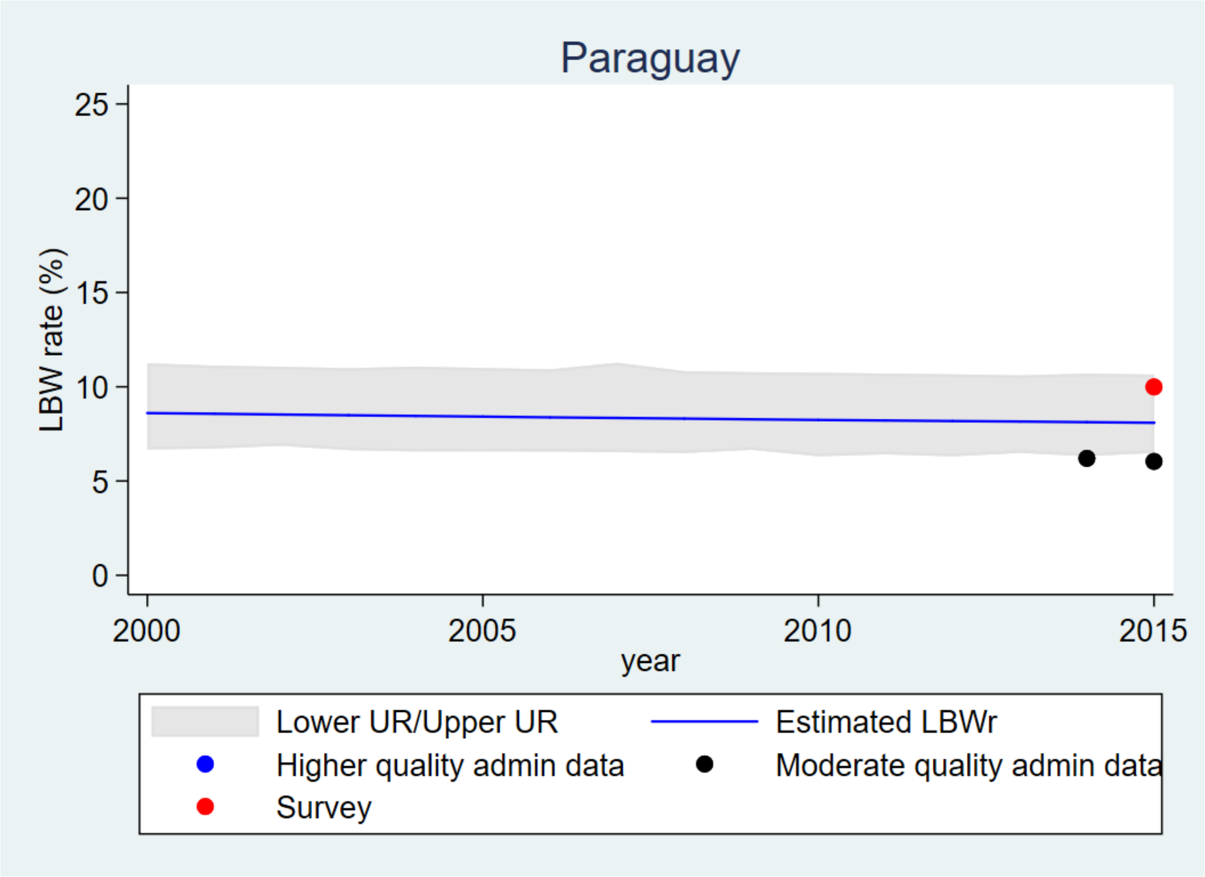
Paraguay
LBW rate (%)
25
20
15
10
5
0
2000
2005
2010
2015
year
Lower UR/Upper UR
Estimated LBWr
Higher quality admin data
Moderate quality admin data
Survey

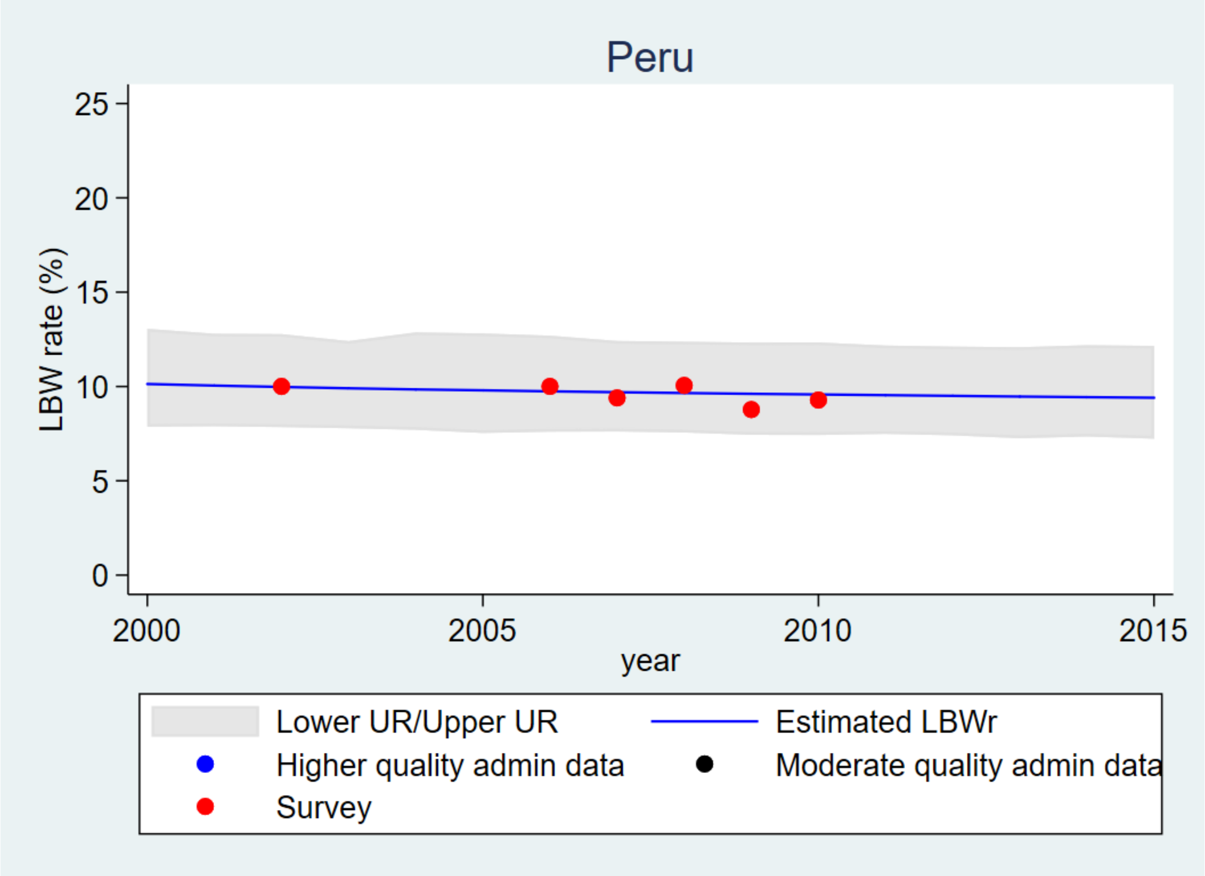
Peru
LBW rate (%)
25
20
15
10
5
0
2000
2005
2010
2015
year
Lower UR/Upper UR
Estimated LBWr
Higher quality admin data
Moderate quality admin data
Survey

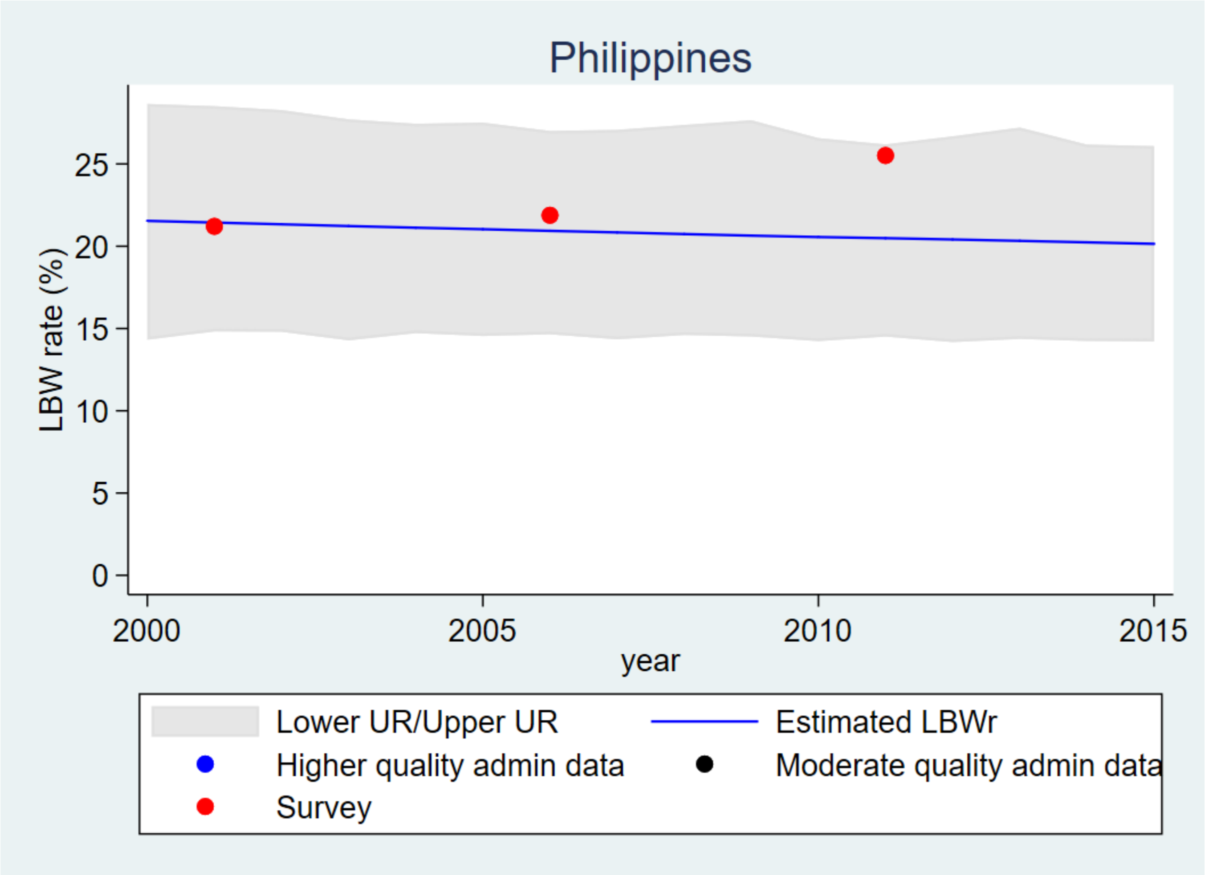
Philippines
LBW rate (%)
year
Lower UR/Upper UR
Estimated LBWr
Higher quality admin data
Moderate quality admin data
Survey

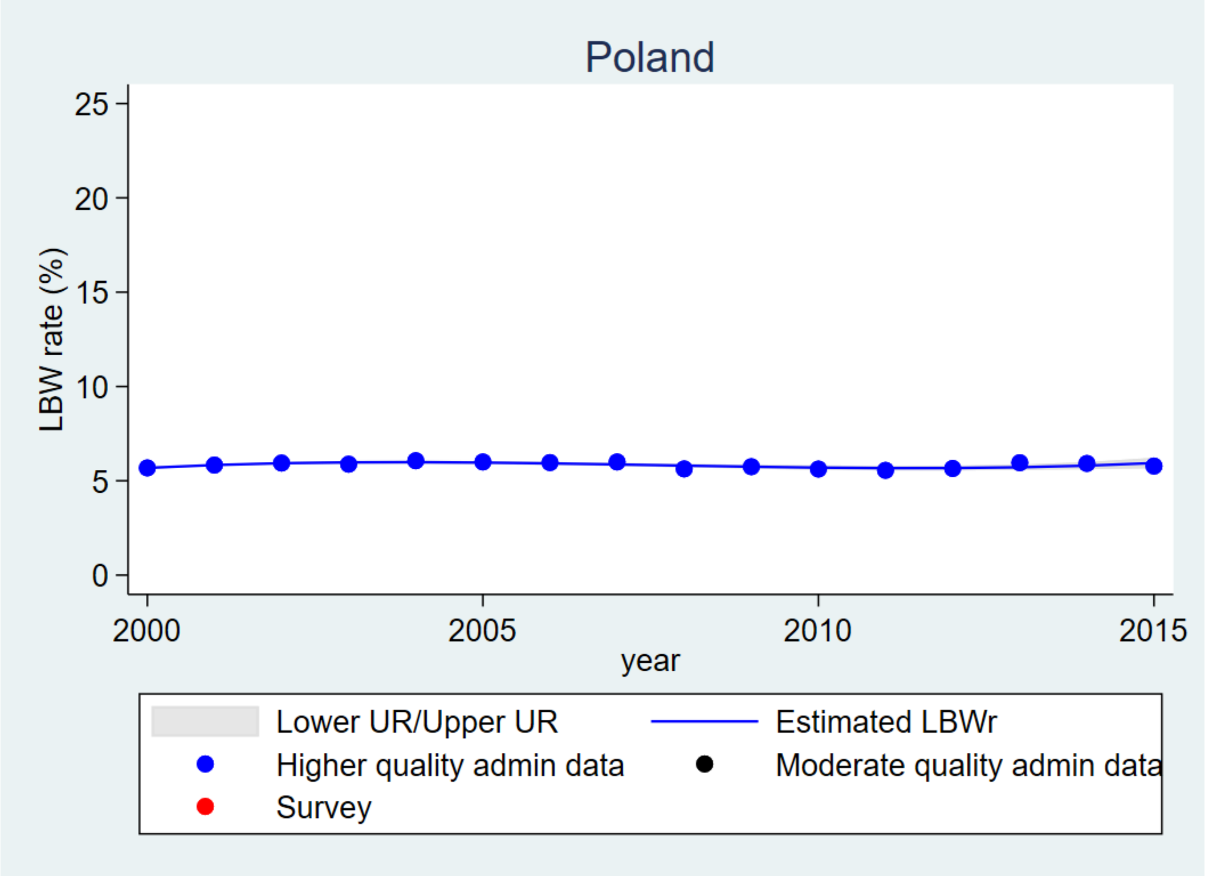
Poland
LBW rate (%)
year
Lower UR/Upper UR
Estimated LBWr
Higher quality admin data
Moderate quality admin data
Survey

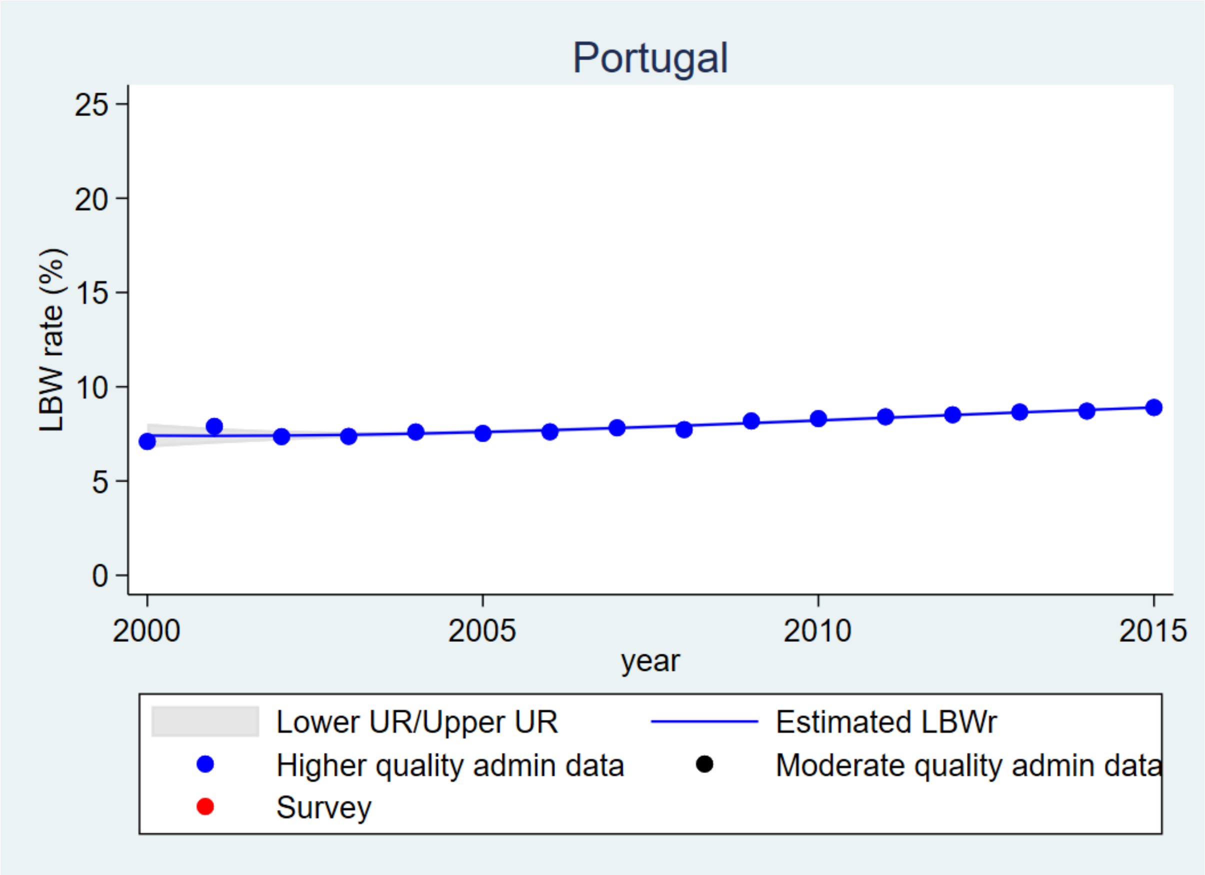
Portugal
LBW rate (%)
0
5
10
15
20
25
2000
2005
2010
2015
year
Lower UR/Upper UR
Estimated LBWr
Higher quality admin data
Moderate quality admin data
Survey

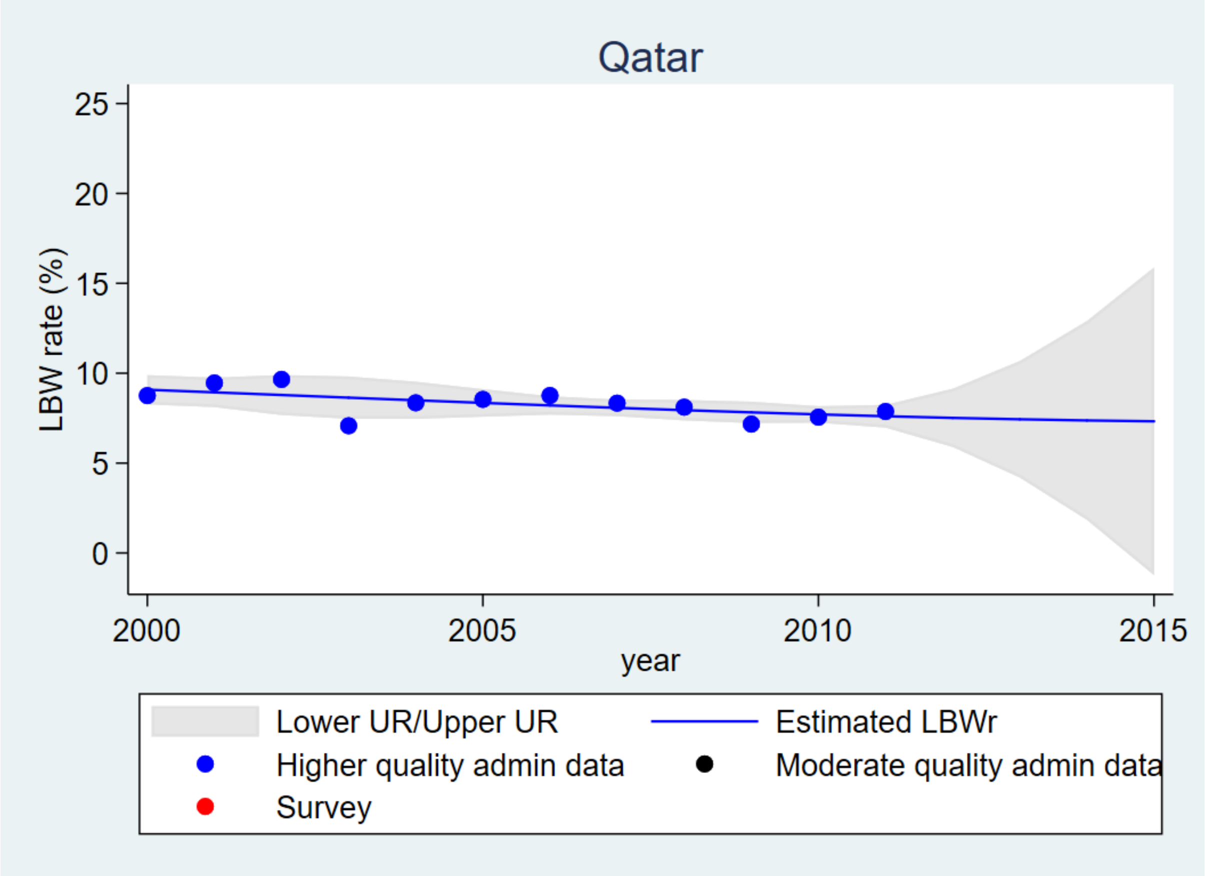
Qatar
LBW rate (%)
0
5
10
15
20
25
2000
2005
2010
2015
year
Lower UR/Upper UR
Estimated LBWr
Higher quality admin data
Moderate quality admin data
Survey

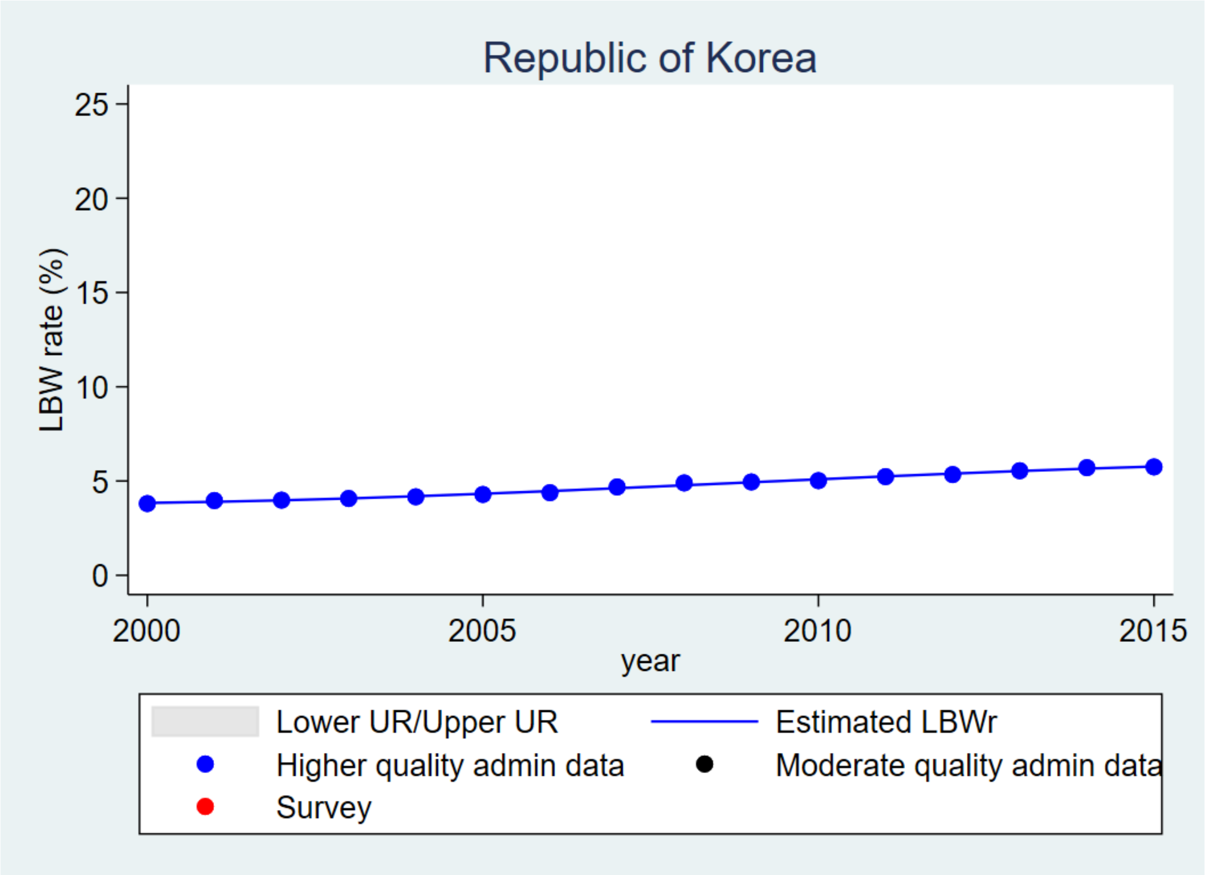

Republic of Korea
LBW rate (%)
25
20
15
10
5
0
2000
2005
2010
2015
year
Lower UR/Upper UR
Estimated LBWr
Higher quality admin data
Moderate quality admin data
Survey

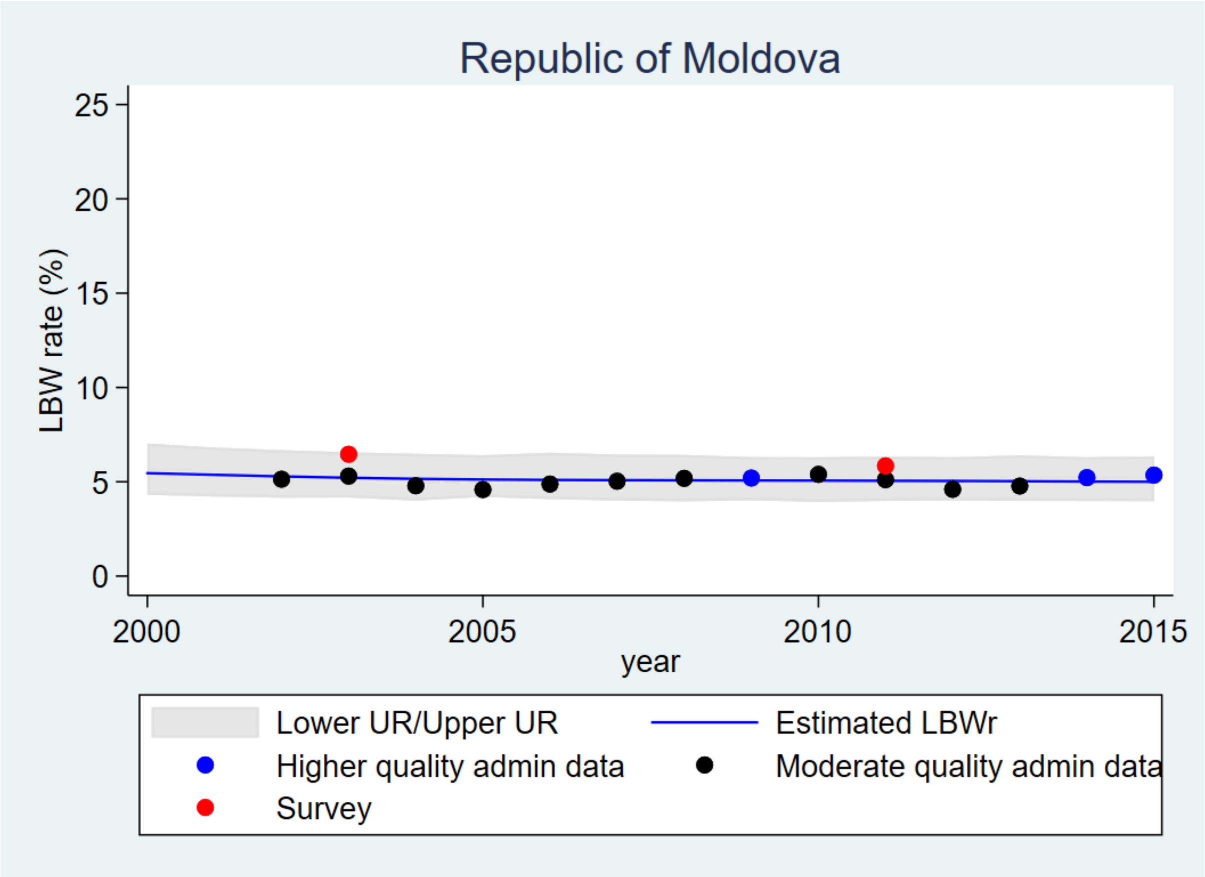

Republic of Moldova
LBW rate (%)
25
20
15
10
5
0
2000
2005
2010
2015
year
Lower UR/Upper UR
Estimated LBWr
Higher quality admin data
Moderate quality admin data
Survey

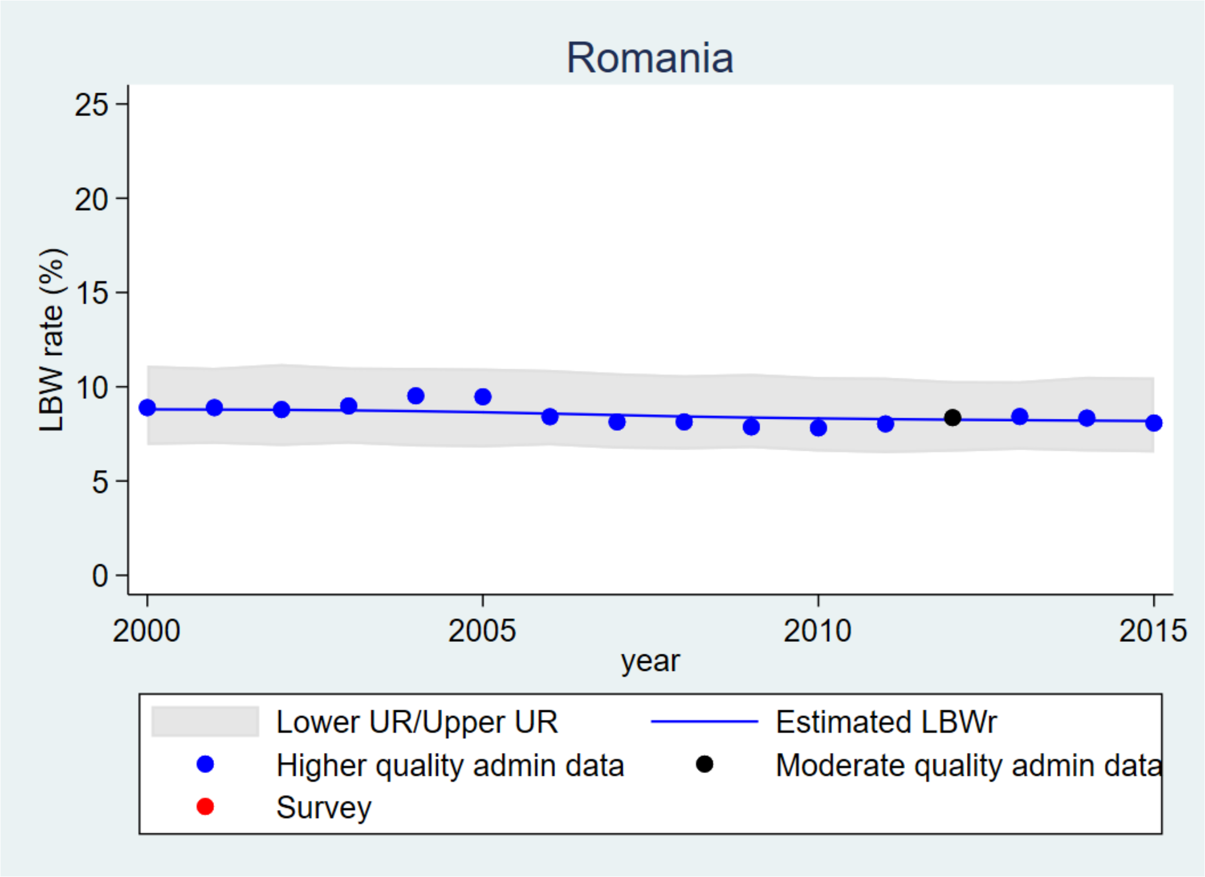
Romania
LBW rate (%)
25
20
15
10
5
0
2000
2005
2010
2015
year
Lower UR/Upper UR
Estimated LBWr
Higher quality admin data
Moderate quality admin data
Survey

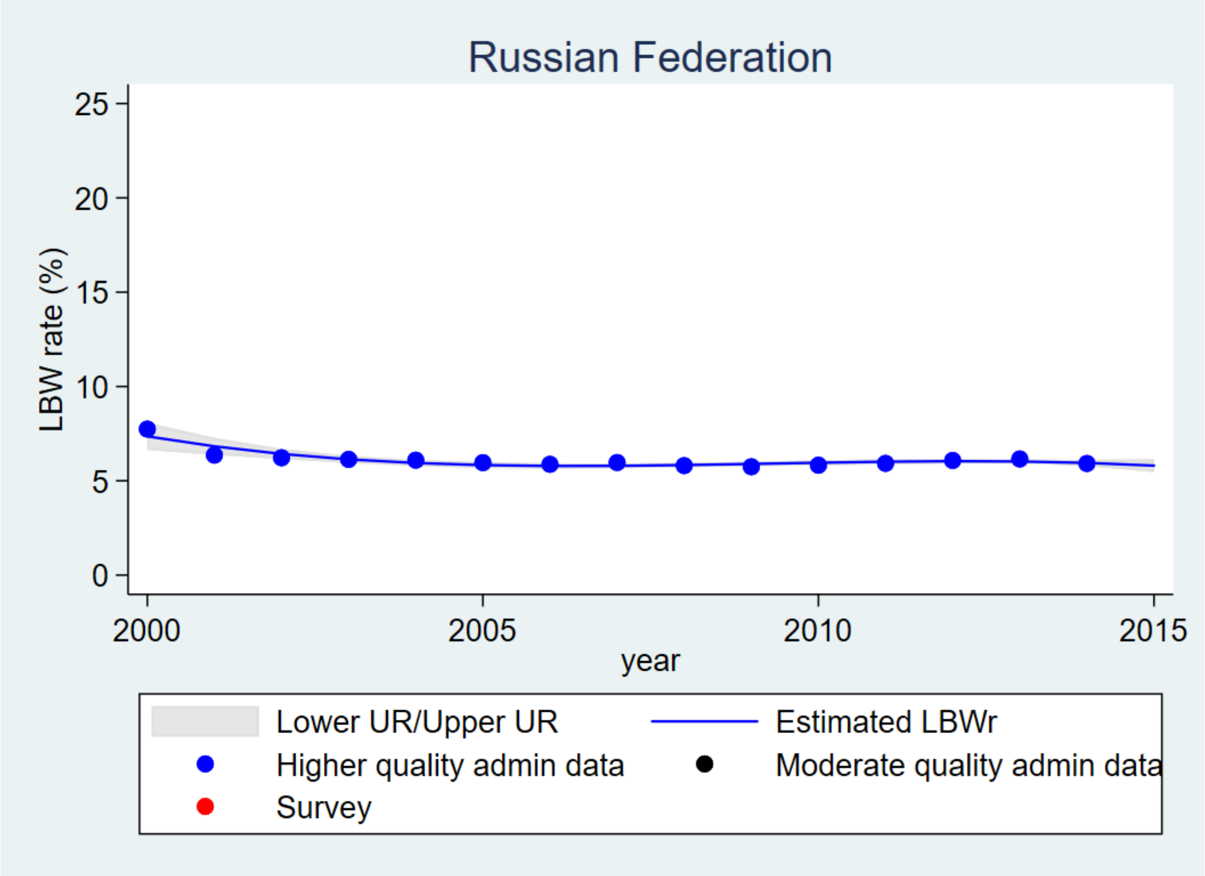
Russian Federation
LBW rate (%)
25
20
15
10
5
0
2000
2005
2010
2015
year
Lower UR/Upper UR
Estimated LBWr
Higher quality admin data
Moderate quality admin data
Survey

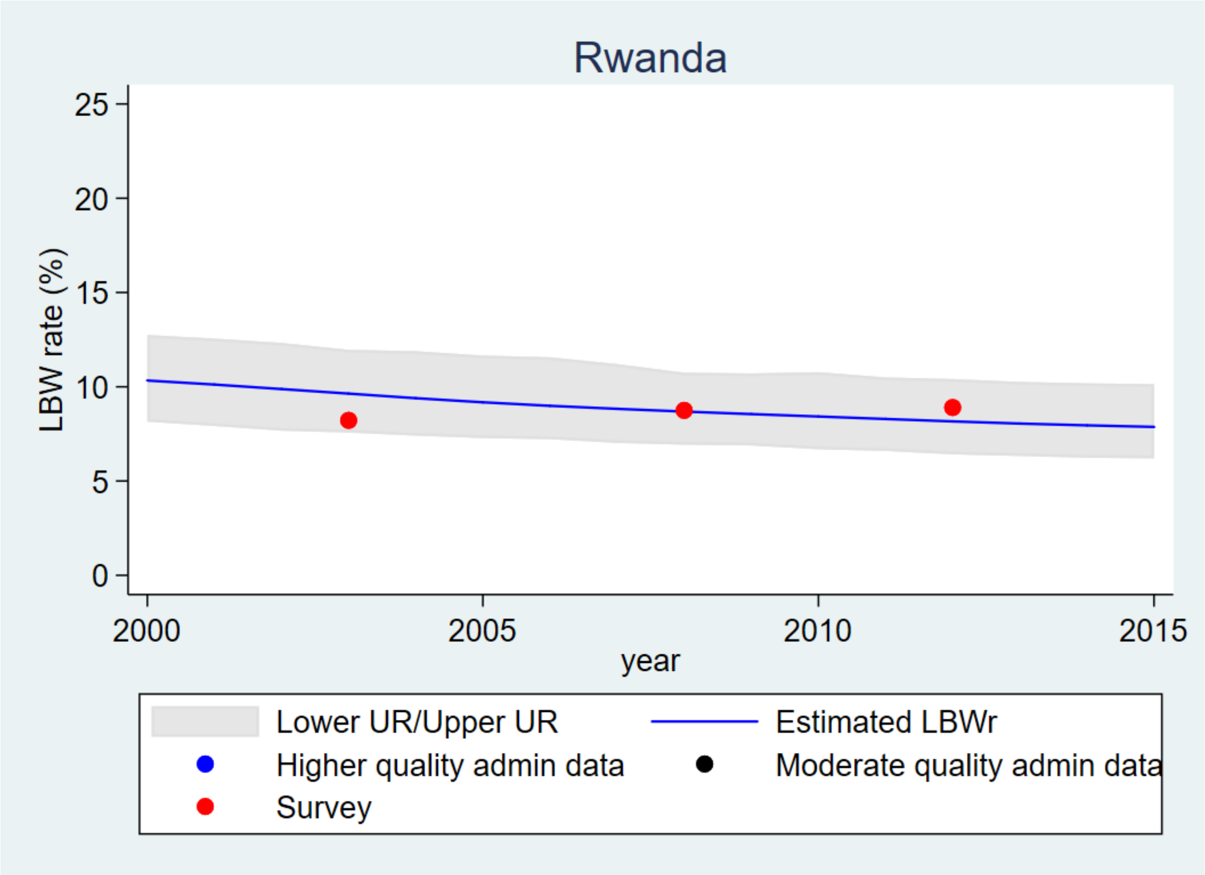
Rwanda
LBW rate (%)
25
20
15
10
5
0
2000
2005
2010
2015
year
Lower UR/Upper UR
Estimated LBWr
Higher quality admin data
Moderate quality admin data
Survey

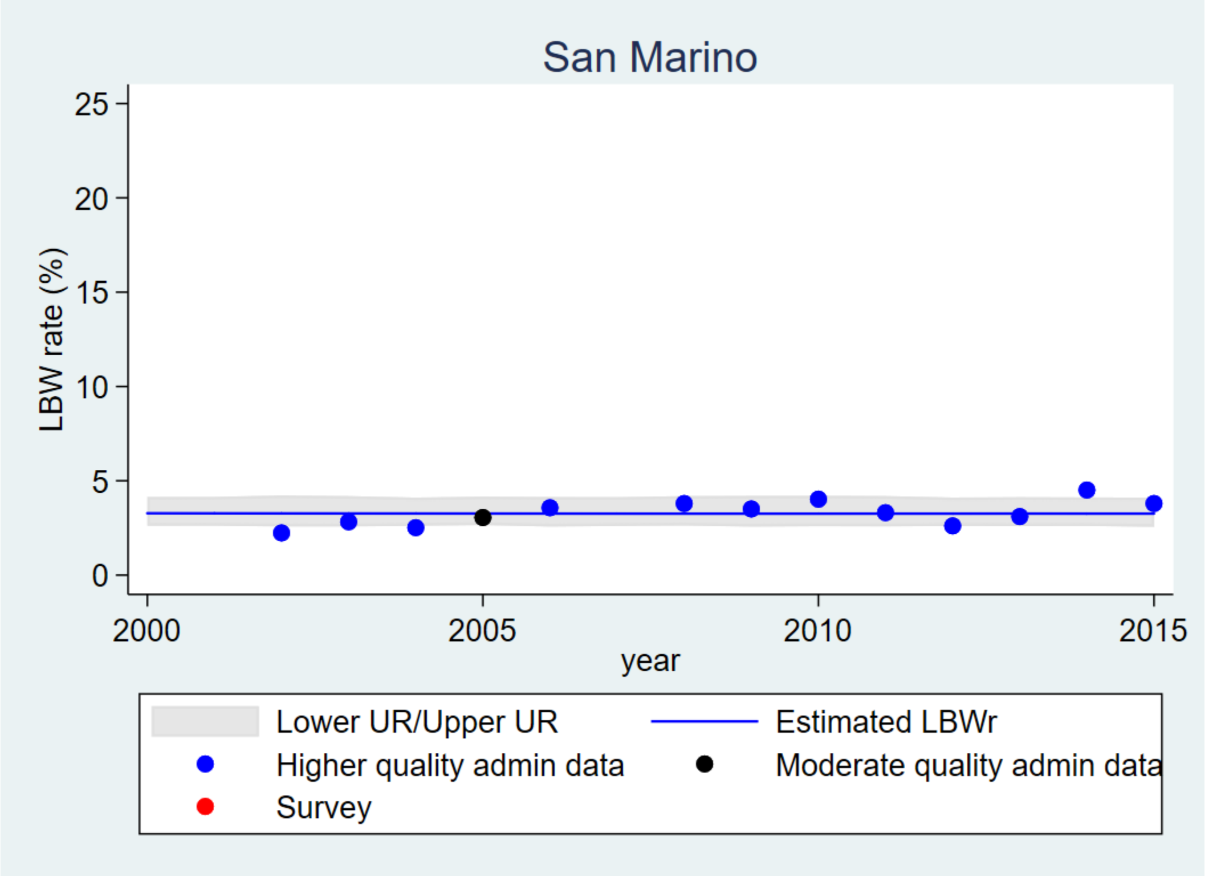
San Marino
LBW rate (%)
25
20
15
10
5
0
2000
2005
2010
2015
year
Lower UR/Upper UR
Estimated LBWr
Higher quality admin data
Moderate quality admin data
Survey

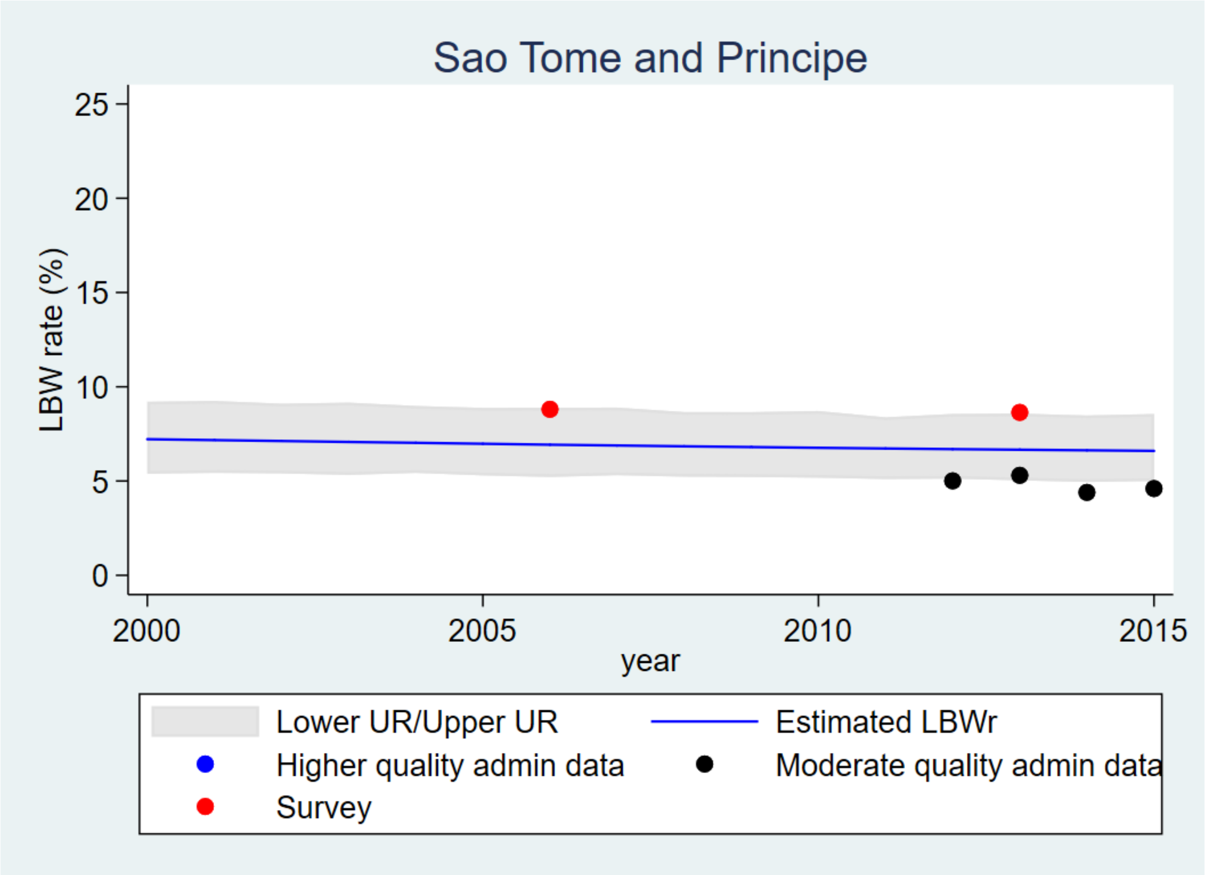
Sao Tome and Principe
LBW rate (%)
0
5
10
15
20
25
2000
2005
2010
2015
year
Lower UR/Upper UR
Estimated LBWr
Higher quality admin data
Moderate quality admin data
Survey

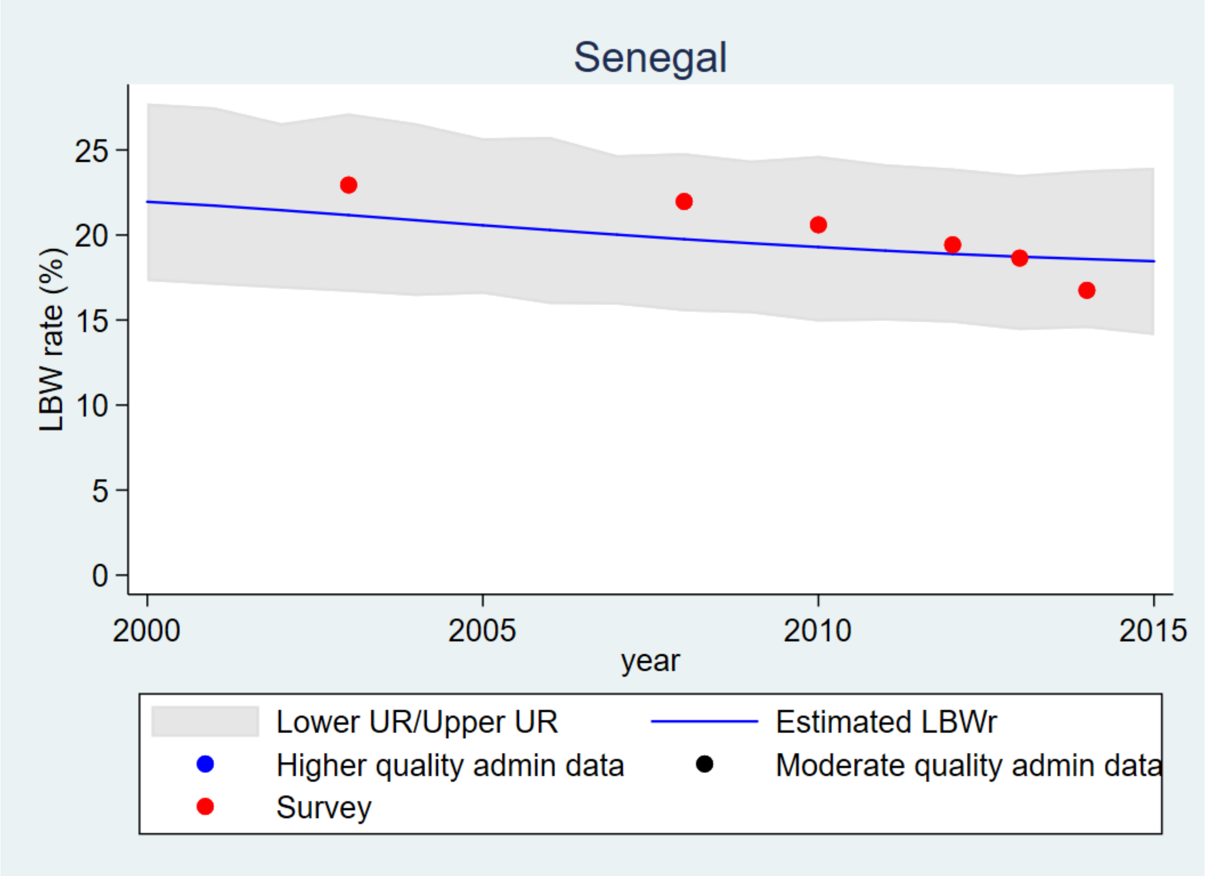
Senegal
LBW rate (%)
0
5
10
15
20
25
2000
2005
2010
2015
year
Lower UR/Upper UR
Estimated LBWr
Higher quality admin data
Moderate quality admin data
Survey

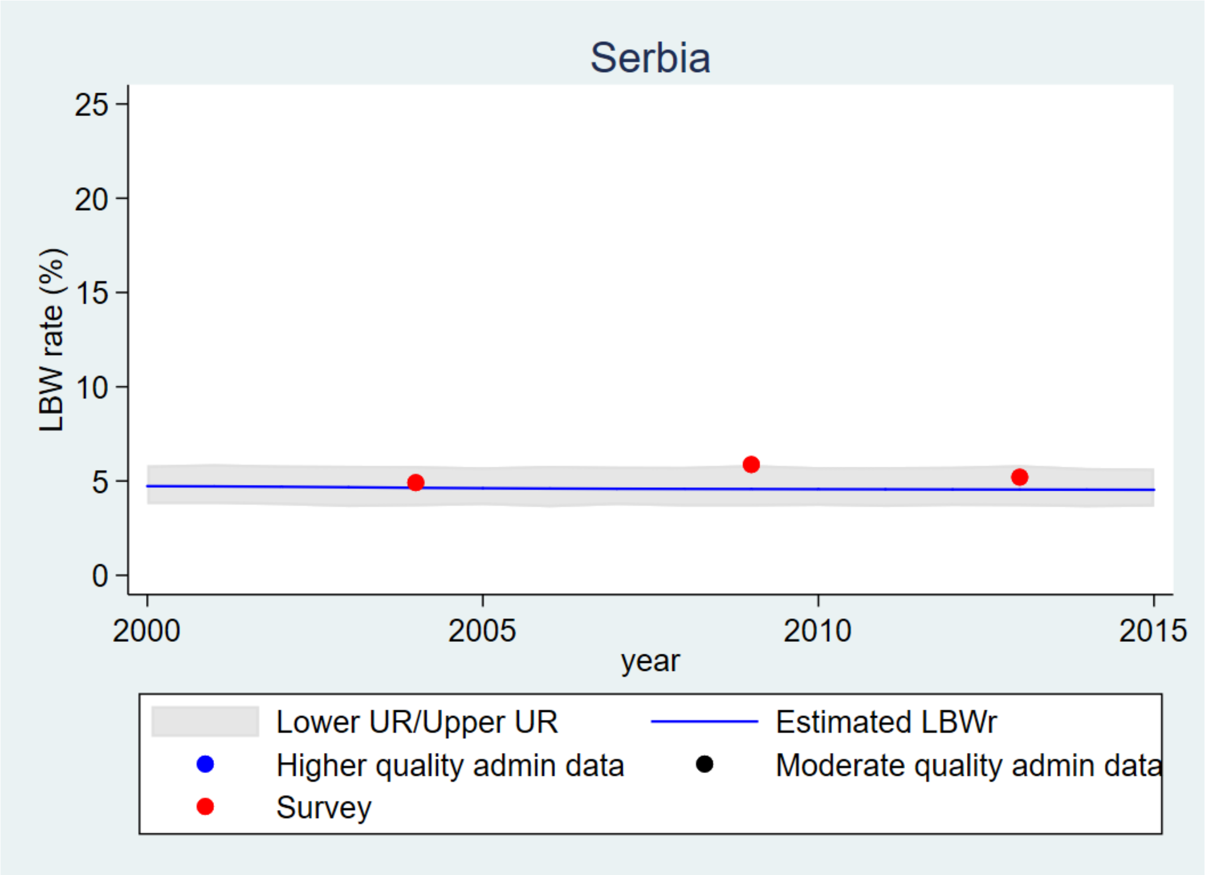
Serbia
LBW rate (%)
25
20
15
10
5
0
2000
2005
2010
2015
year
Lower UR/Upper UR
Estimated LBWr
Higher quality admin data
Moderate quality admin data
Survey

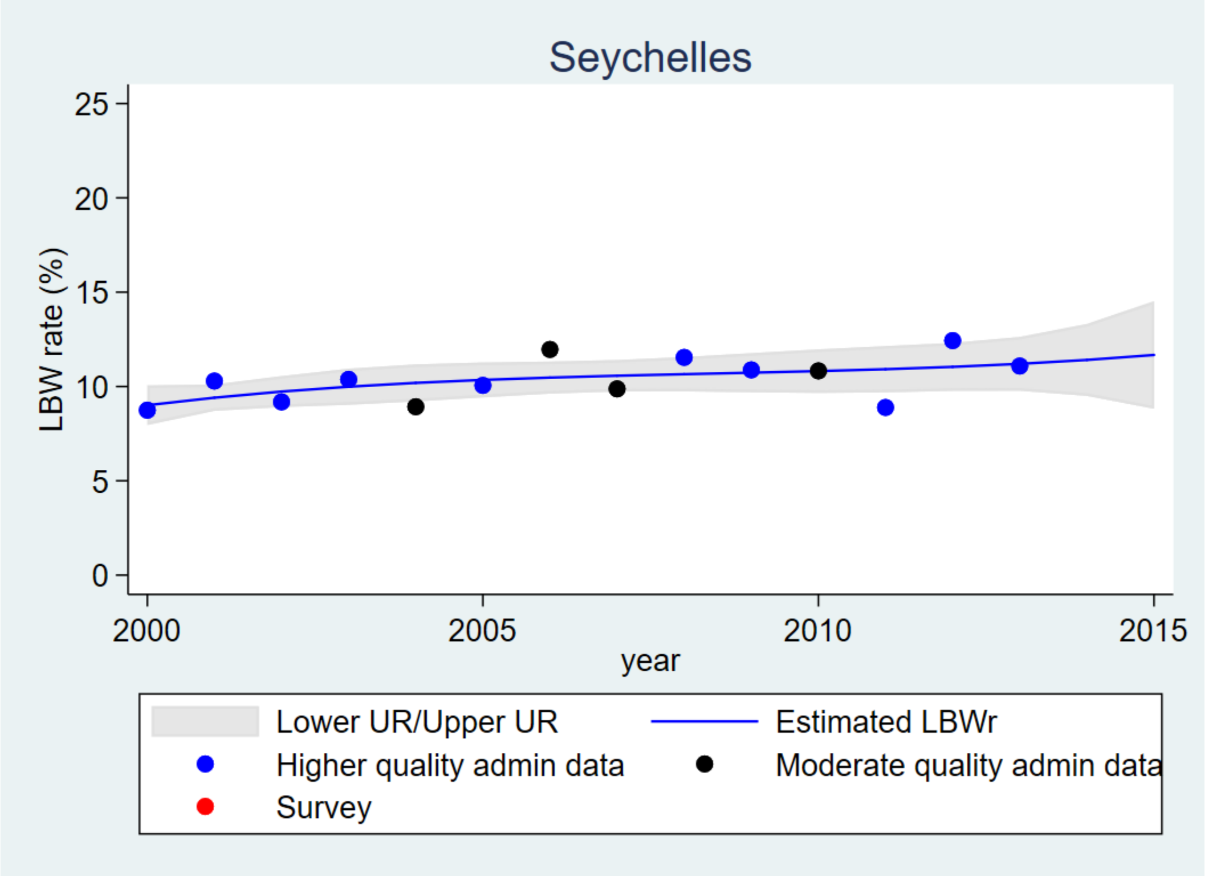
Seychelles
LBW rate (%)
25
20
15
10
5
0
2000
2005
2010
2015
year
Lower UR/Upper UR
Estimated LBWr
Higher quality admin data
Moderate quality admin data
Survey

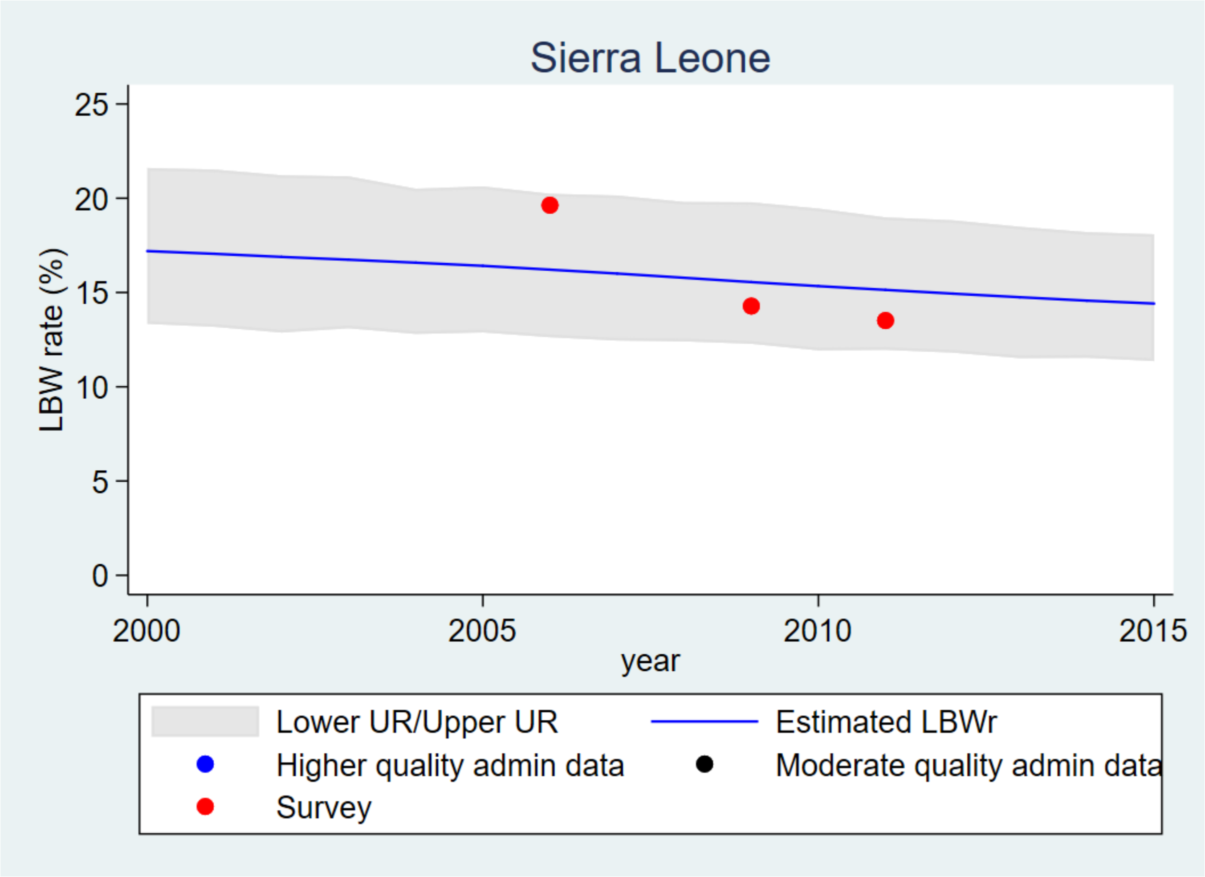
Sierra Leone
LBW rate (%)
25
20
15
10
5
0
2000
2005
2010
2015
year
Lower UR/Upper UR
Estimated LBWr
Higher quality admin data
Moderate quality admin data
Survey

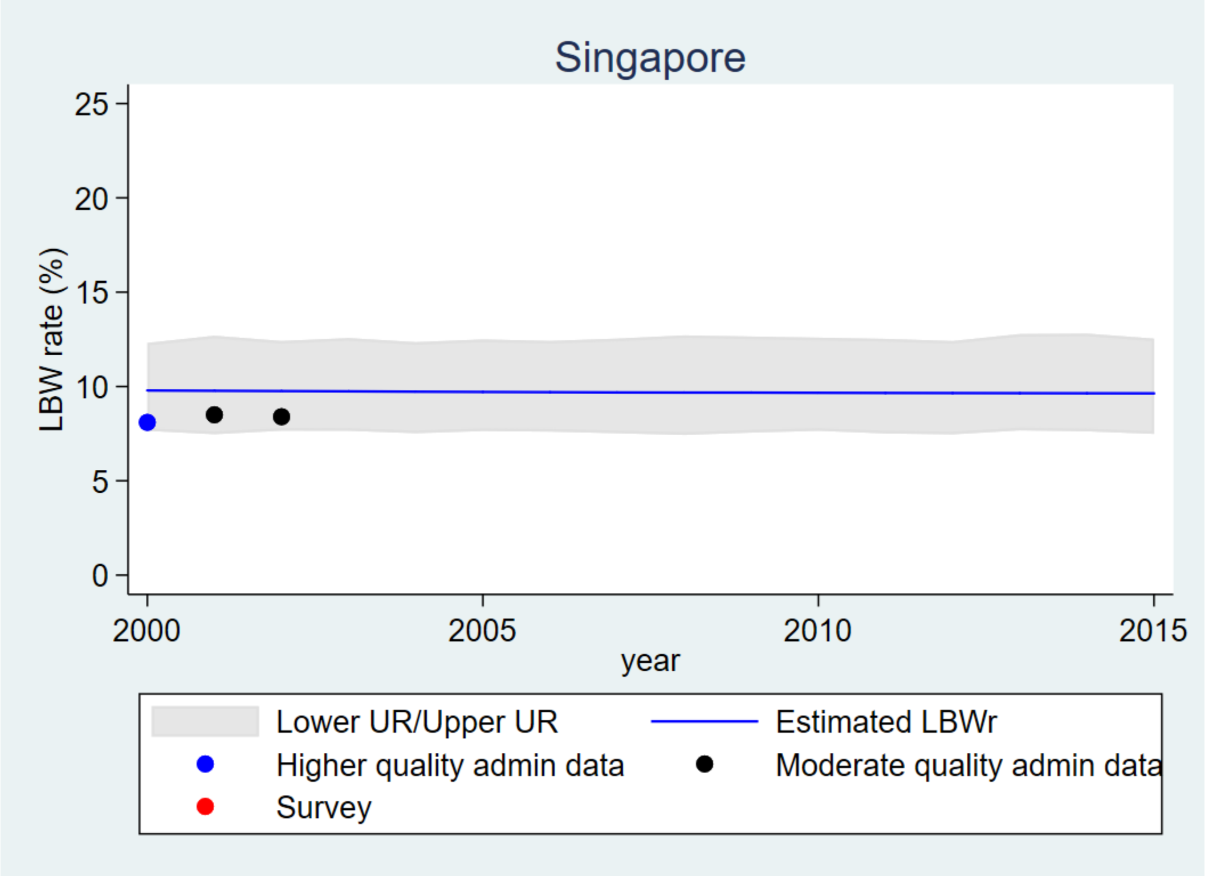
Singapore
LBW rate (%)
25
20
15
10
5
0
2000
2005
2010
2015
year
Lower UR/Upper UR
Estimated LBWr
Higher quality admin data
Moderate quality admin data
Survey

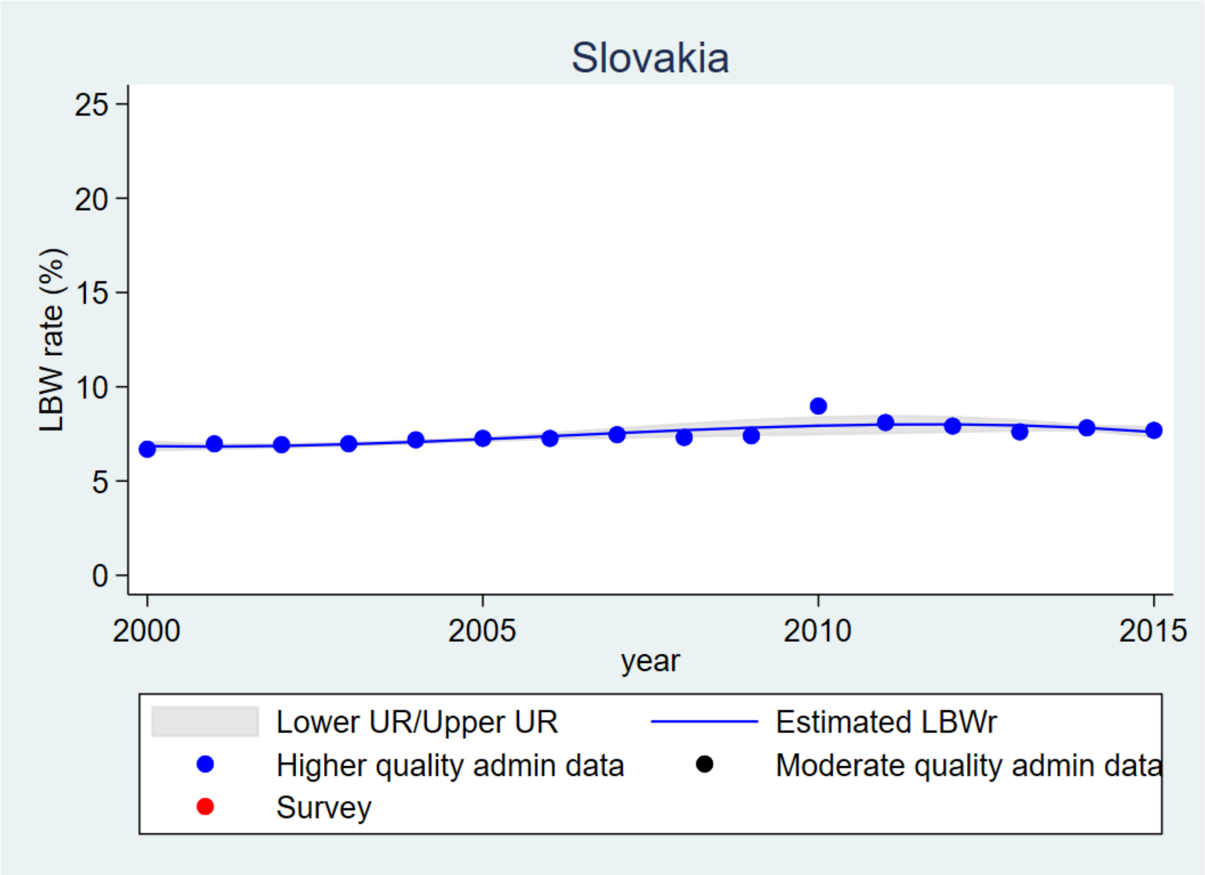
Slovakia
LBW rate (%)
0
5
10
15
20
25
2000
2005
2010
2015
year
Lower UR/Upper UR
Estimated LBWr
Higher quality admin data
Moderate quality admin data
Survey

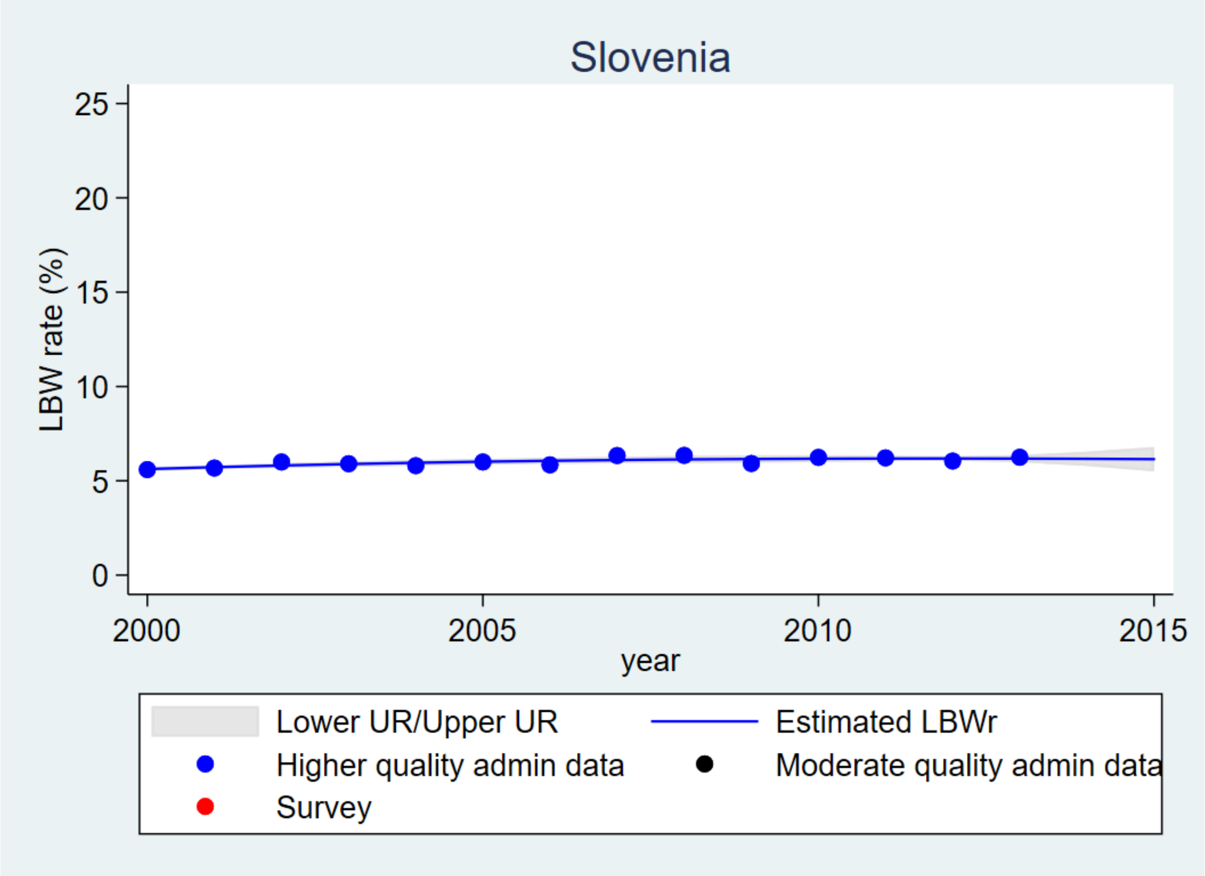
Slovenia
LBW rate (%)
0
5
10
15
20
25
2000
2005
2010
2015
year
Lower UR/Upper UR
Estimated LBWr
Higher quality admin data
Moderate quality admin data
Survey

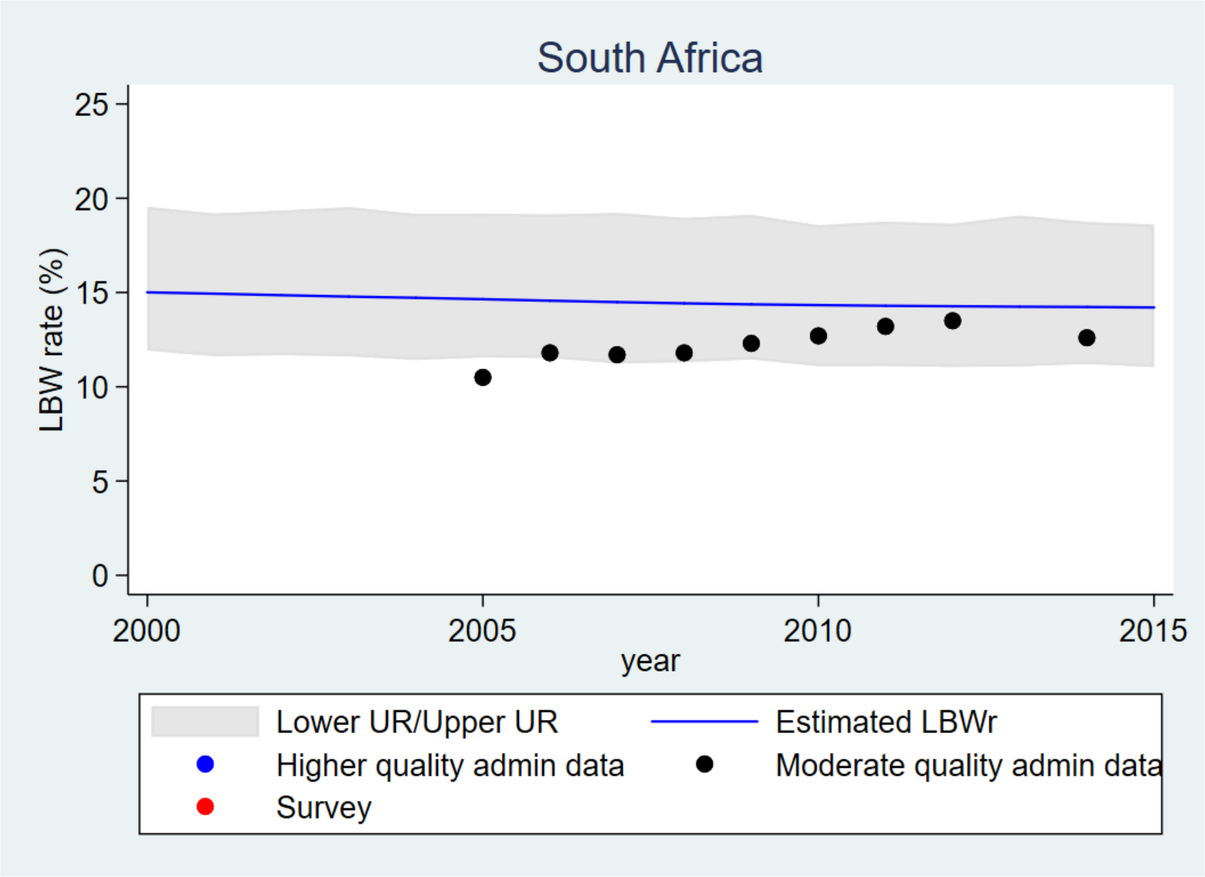

South Africa
25
20
15
10
5
0
LBW rate (%)
2000
2005
2010
2015
year
Lower UR/Upper UR
Estimated LBWr
Higher quality admin data
Moderate quality admin data
Survey

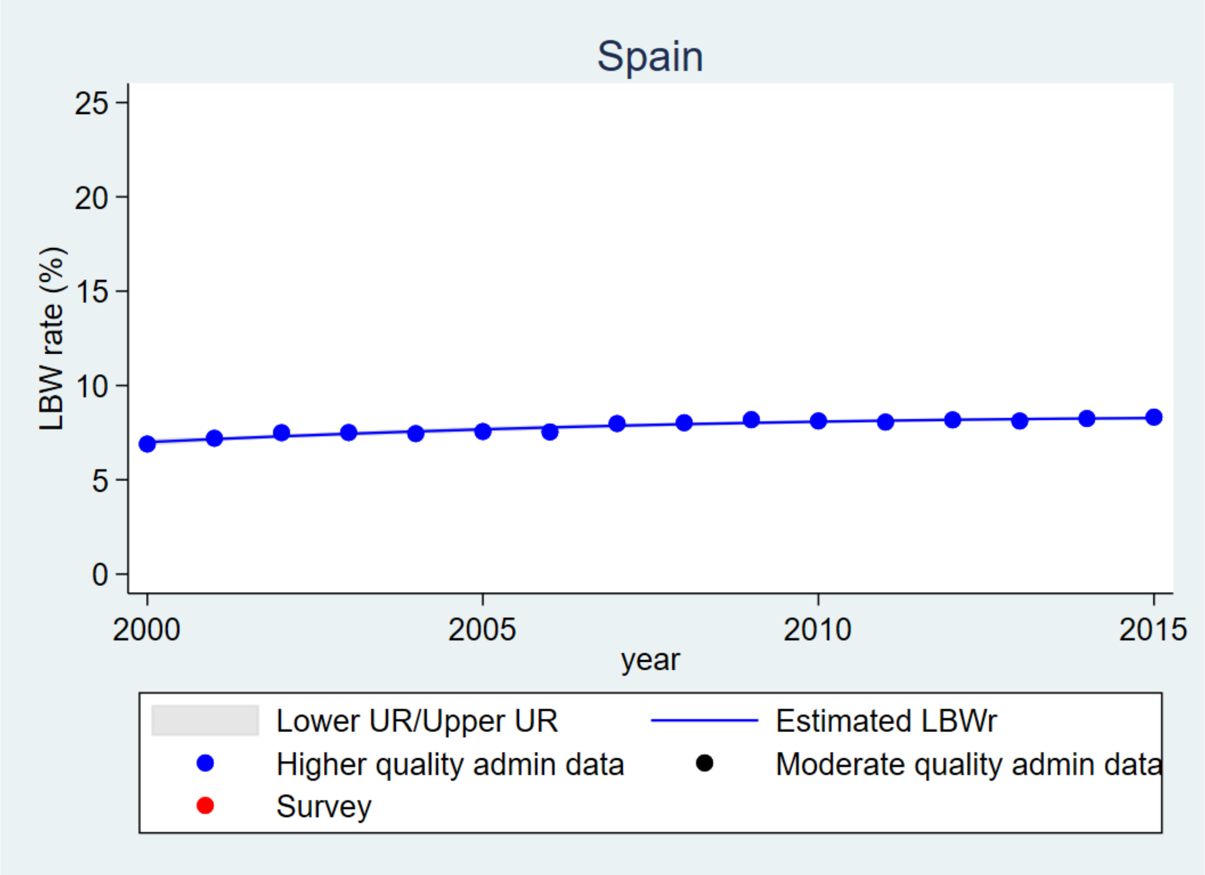

Spain
25
20
15
10
5
0
LBW rate (%)
2000
2005
2010
2015
year
Lower UR/Upper UR
Estimated LBWr
Higher quality admin data
Moderate quality admin data
Survey

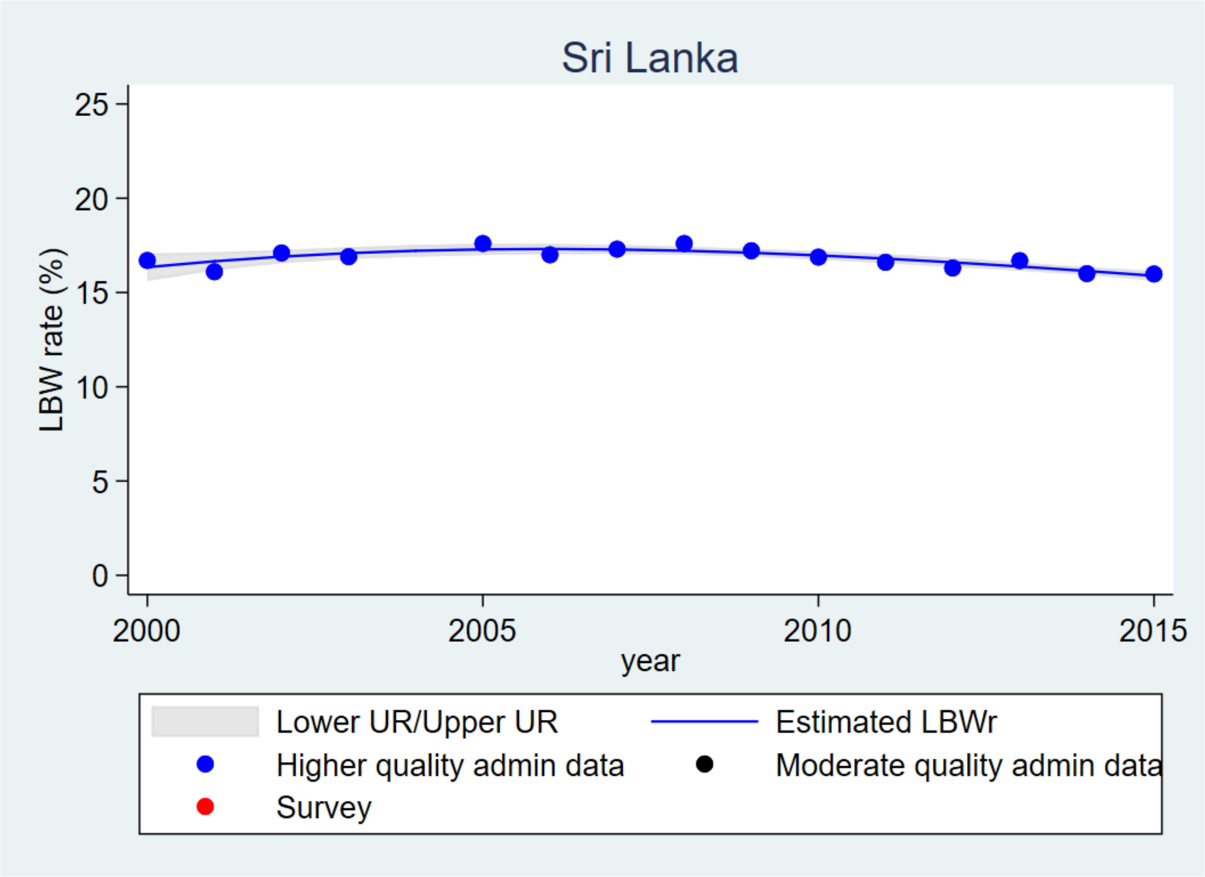
Sri Lanka
LBW rate (%)
25
20
15
10
5
0
2000
2005
2010
2015
year
Lower UR/Upper UR
Estimated LBWr
Higher quality admin data
Moderate quality admin data
Survey

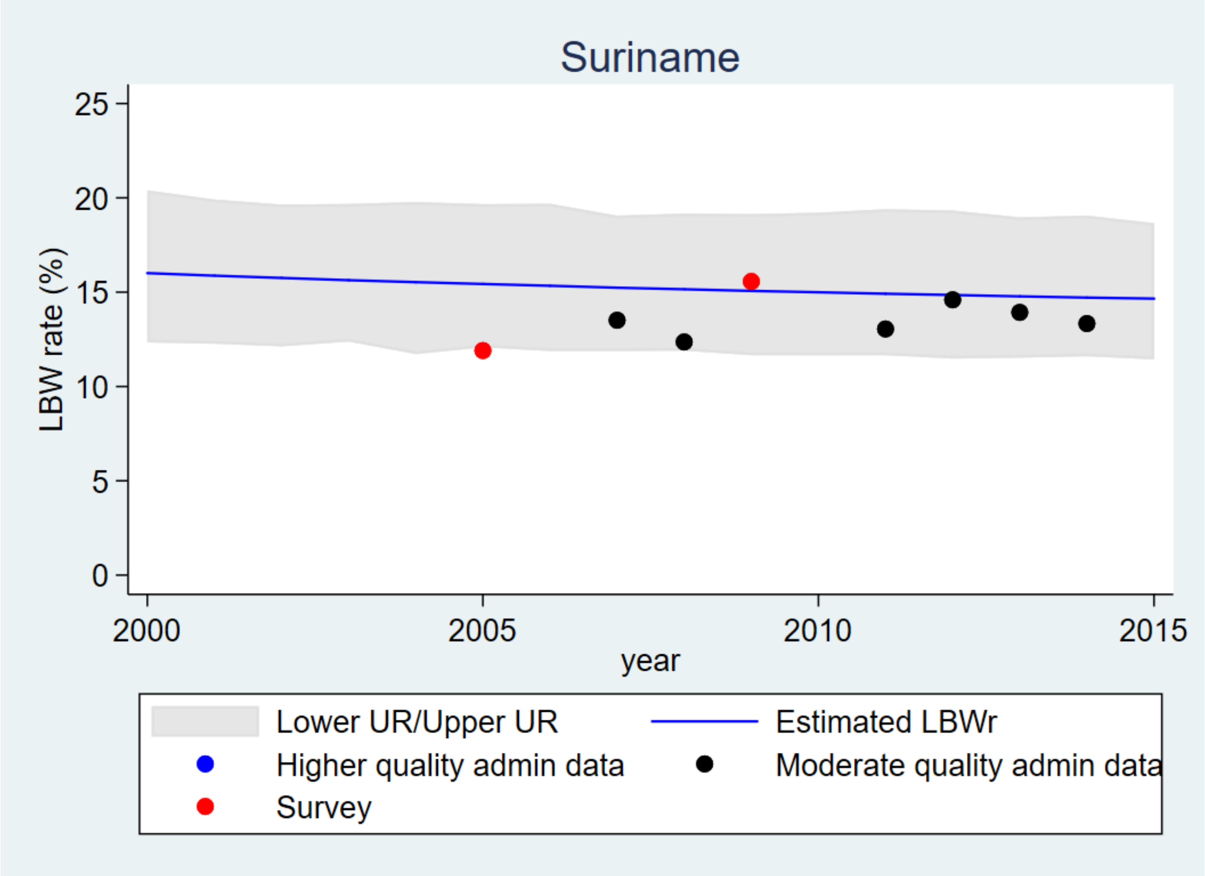
Suriname
LBW rate (%)
25
20
15
10
5
0
2000
2005
2010
2015
year
Lower UR/Upper UR
Estimated LBWr
Higher quality admin data
Moderate quality admin data
Survey

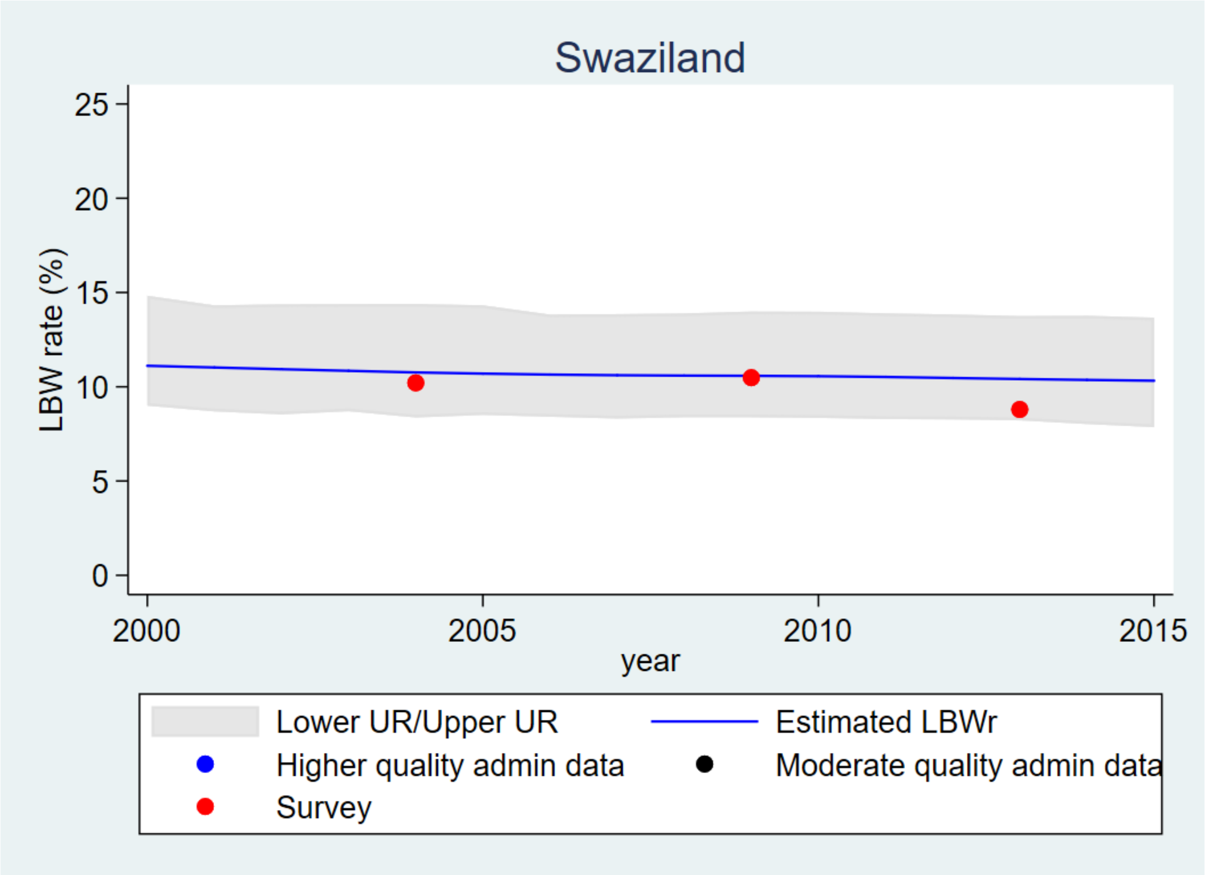
Swaziland
LBW rate (%)
25
20
15
10
5
0
2000
2005
2010
2015
year
Lower UR/Upper UR
Estimated LBWr
Higher quality admin data
Moderate quality admin data
Survey

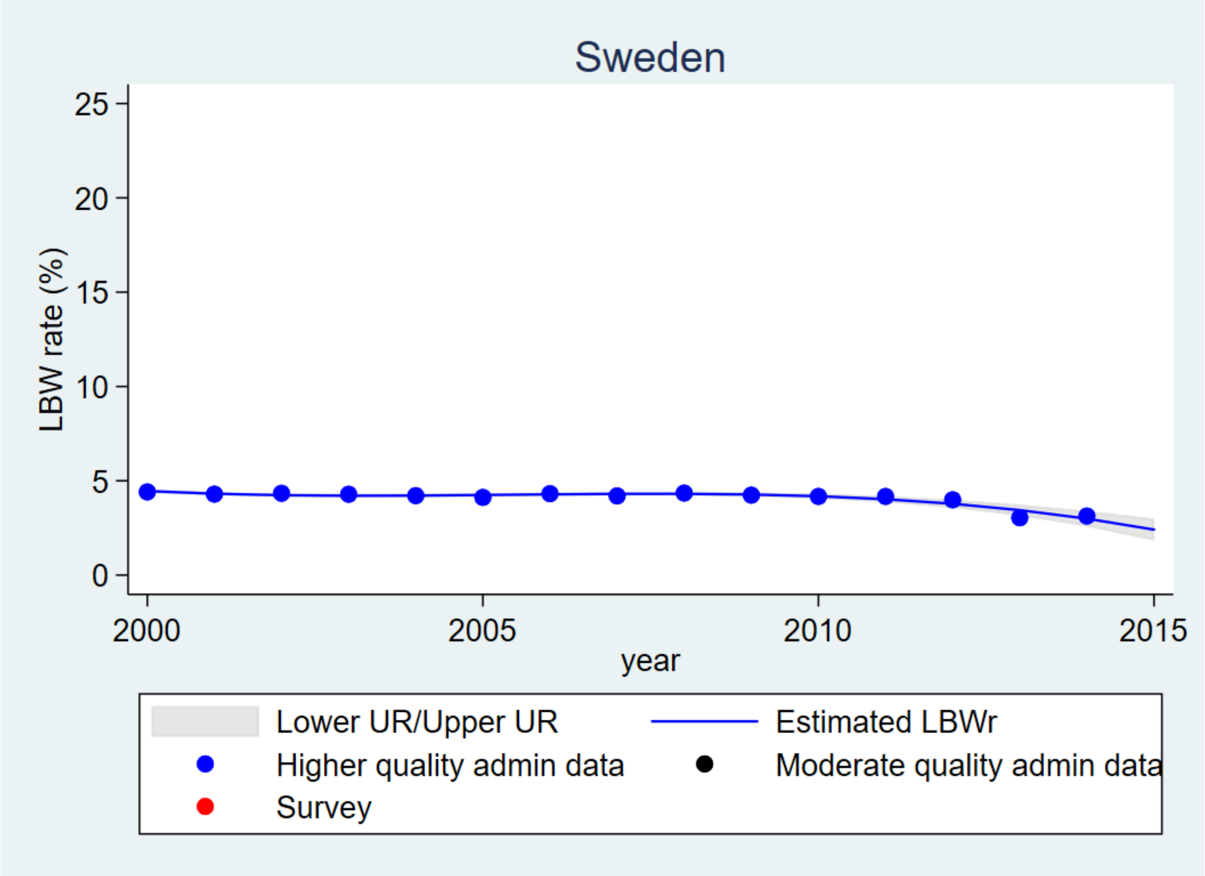
Sweden
LBW rate (%)
25
20
15
10
5
0
2000
2005
2010
2015
year
Lower UR/Upper UR
Estimated LBWr
Higher quality admin data
Moderate quality admin data
Survey

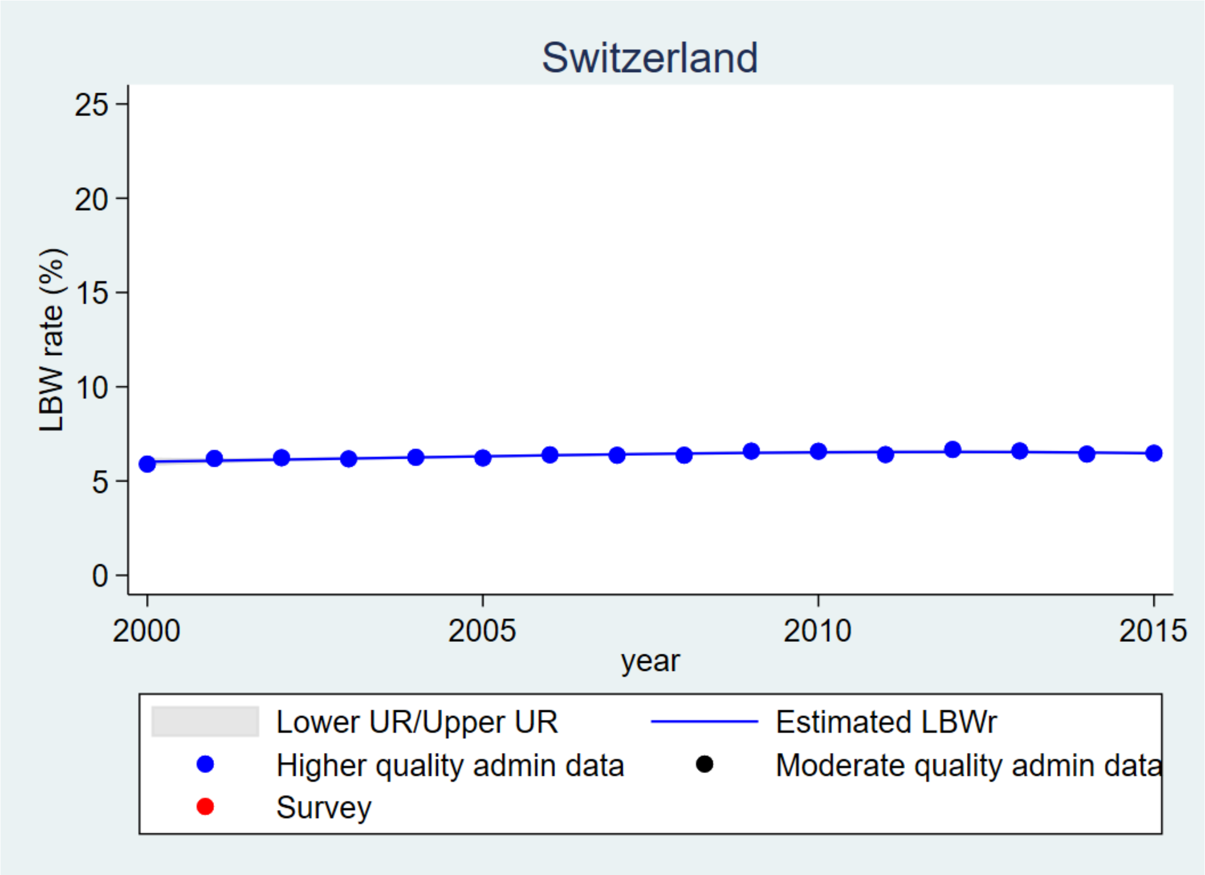
Switzerland
LBW rate (%)
0
5
10
15
20
25
2000
2005
2010
2015
year
Lower UR/Upper UR
Estimated LBWr
Higher quality admin data
Moderate quality admin data
Survey

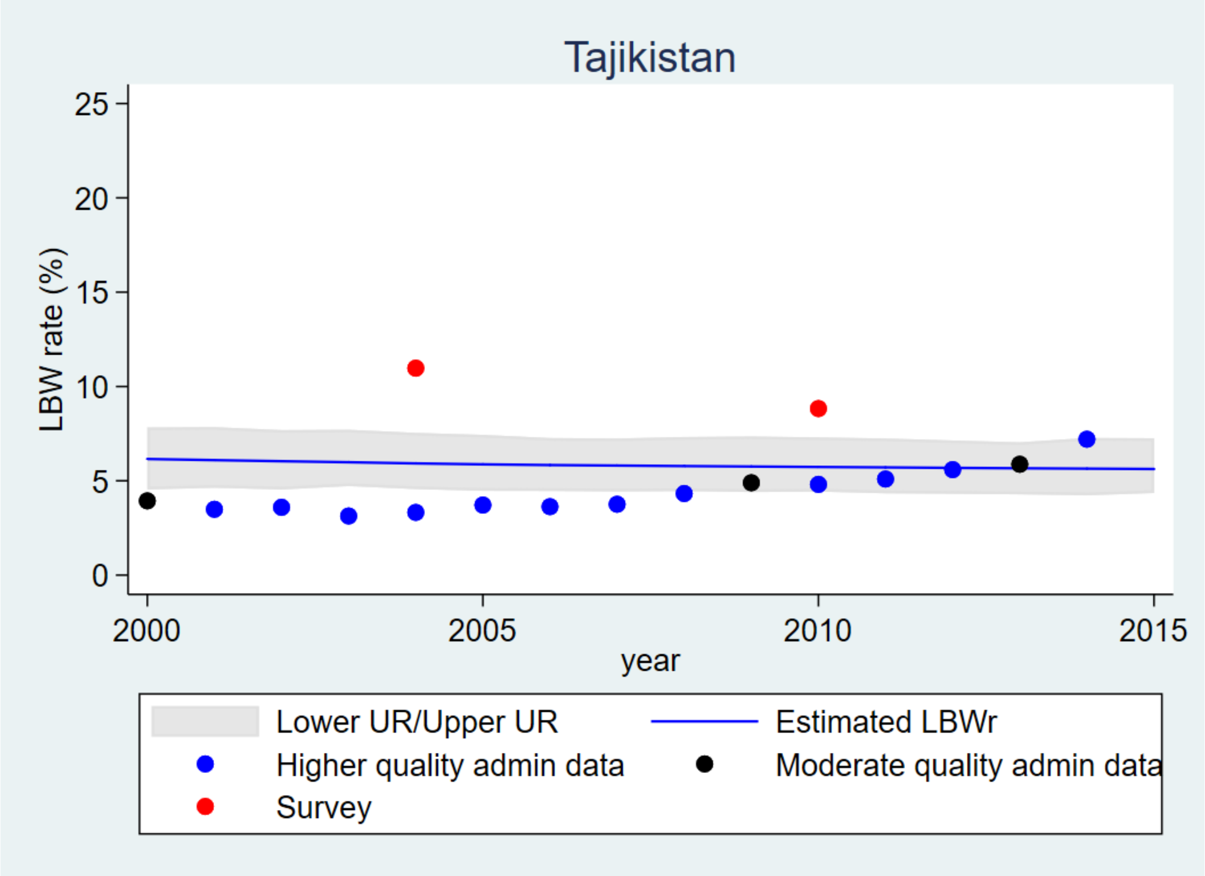
Tajikistan
LBW rate (%)
0
5
10
15
20
25
2000
2005
2010
2015
year
Lower UR/Upper UR
Estimated LBWr
Higher quality admin data
Moderate quality admin data
Survey

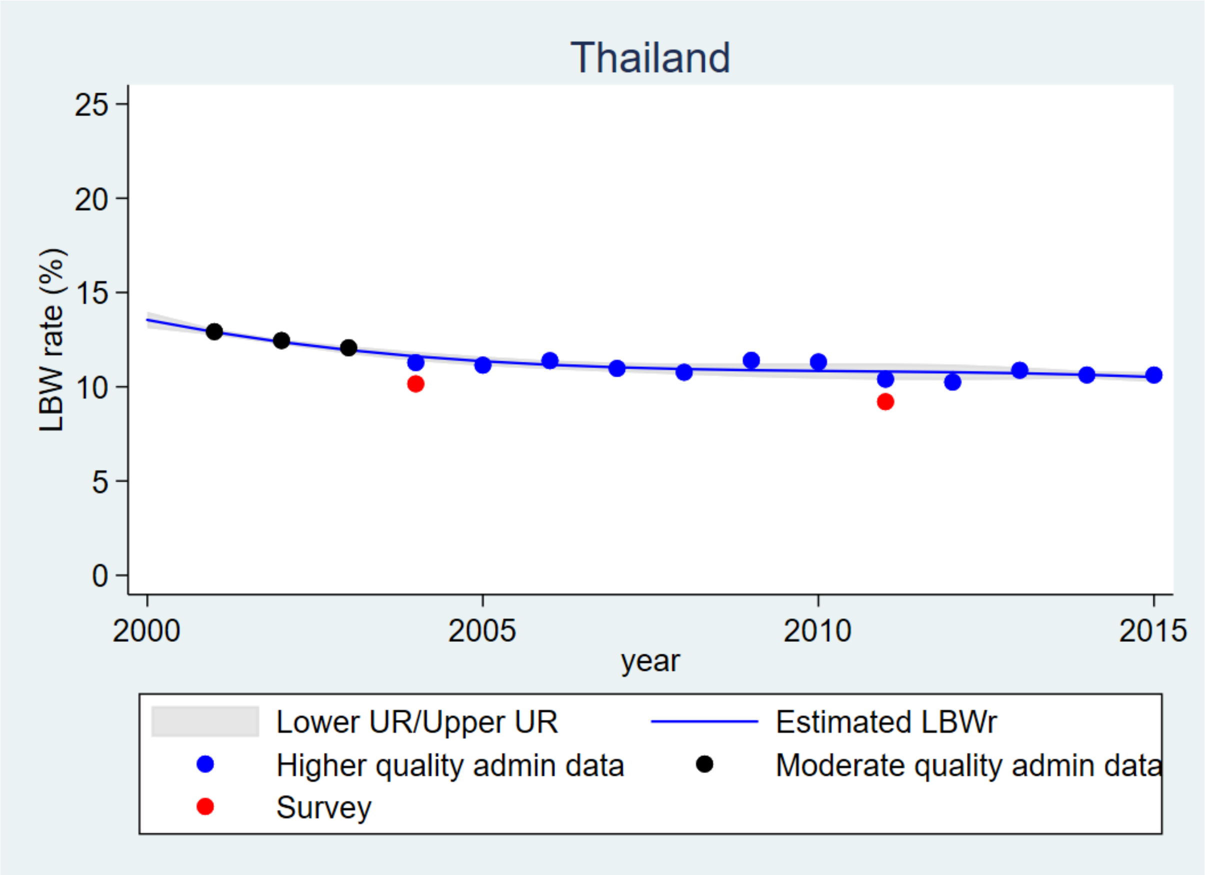
Thailand
LBW rate (%)
25
20
15
10
5
0
2000
2005
2010
2015
year
Lower UR/Upper UR
Estimated LBWr
Higher quality admin data
Moderate quality admin data
Survey

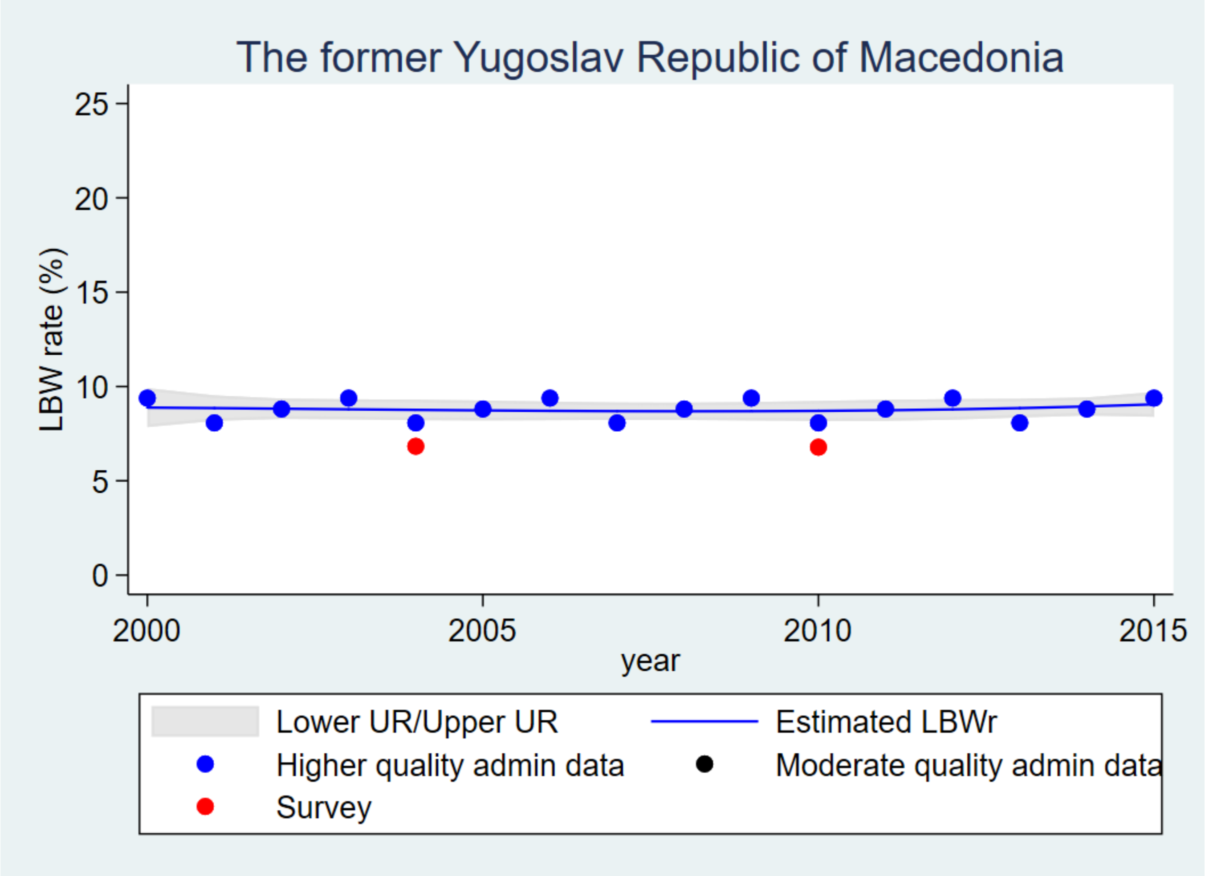
The former Yugoslav Republic of Macedonia
LBW rate (%)
25
20
15
10
5
0
2000
2005
2010
2015
year
Lower UR/Upper UR
Estimated LBWr
Higher quality admin data
Moderate quality admin data
Survey

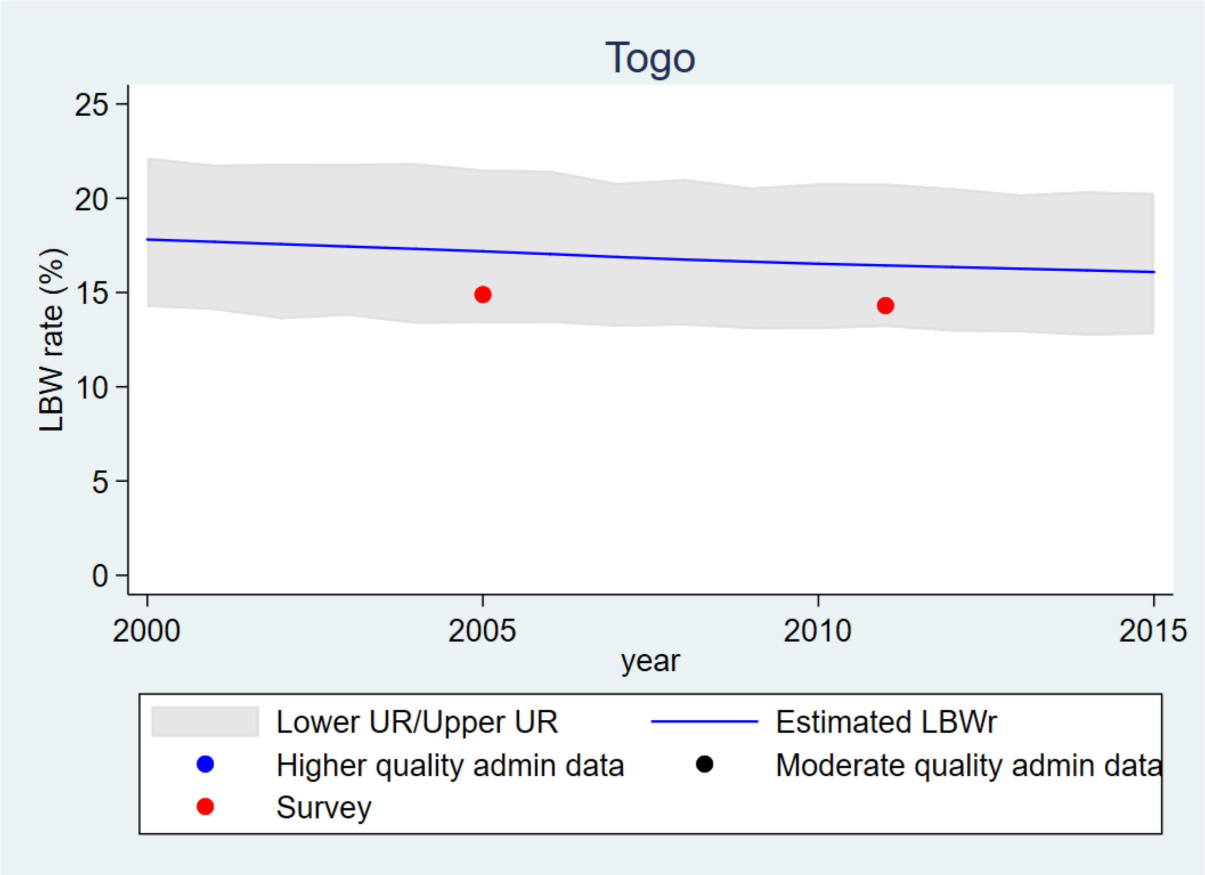
Togo
LBW rate (%)
0
5
10
15
20
25
2000
2005
2010
2015
year
Lower UR/Upper UR
Estimated LBWr
Higher quality admin data
Moderate quality admin data
Survey

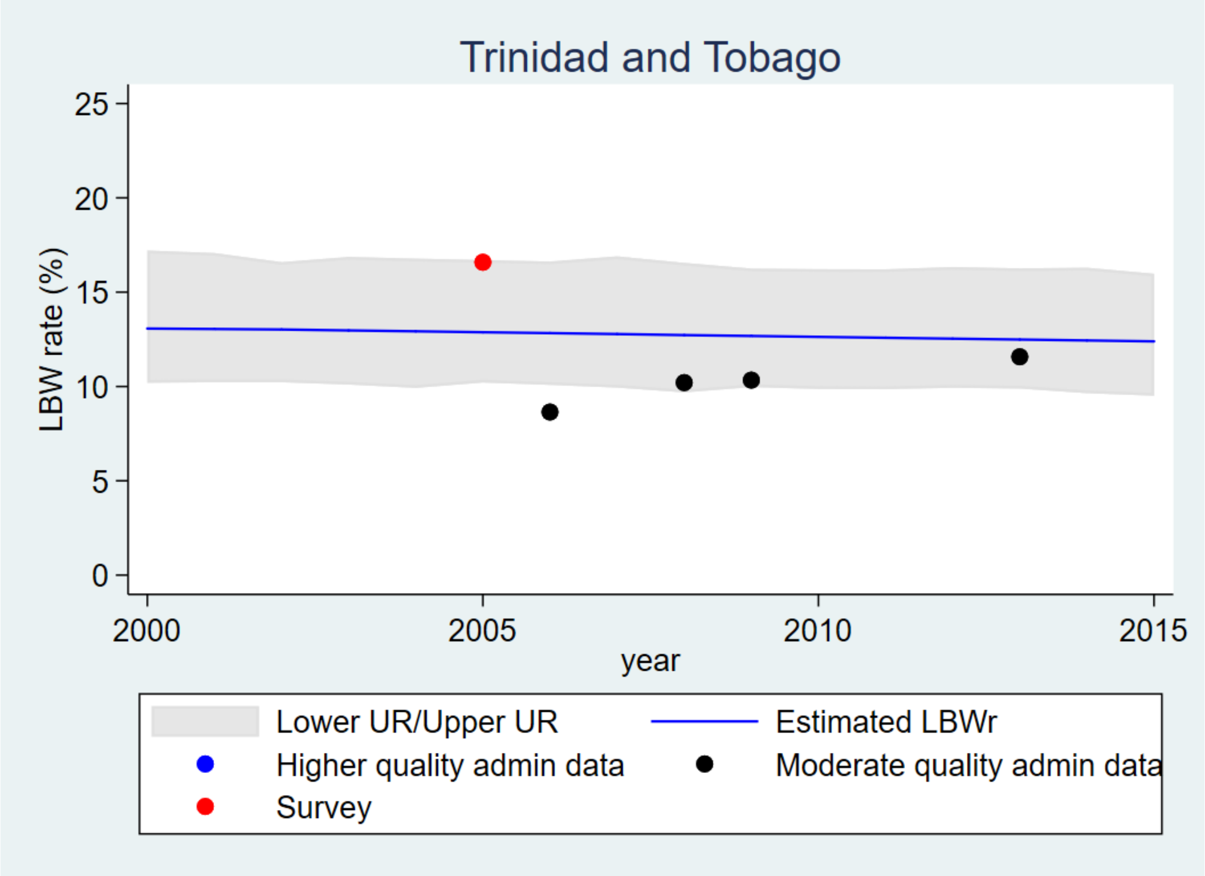
Trinidad and Tobago
LBW rate (%)
0
5
10
15
20
25
2000
2005
2010
2015
year
Lower UR/Upper UR
Estimated LBWr
Higher quality admin data
Moderate quality admin data
Survey

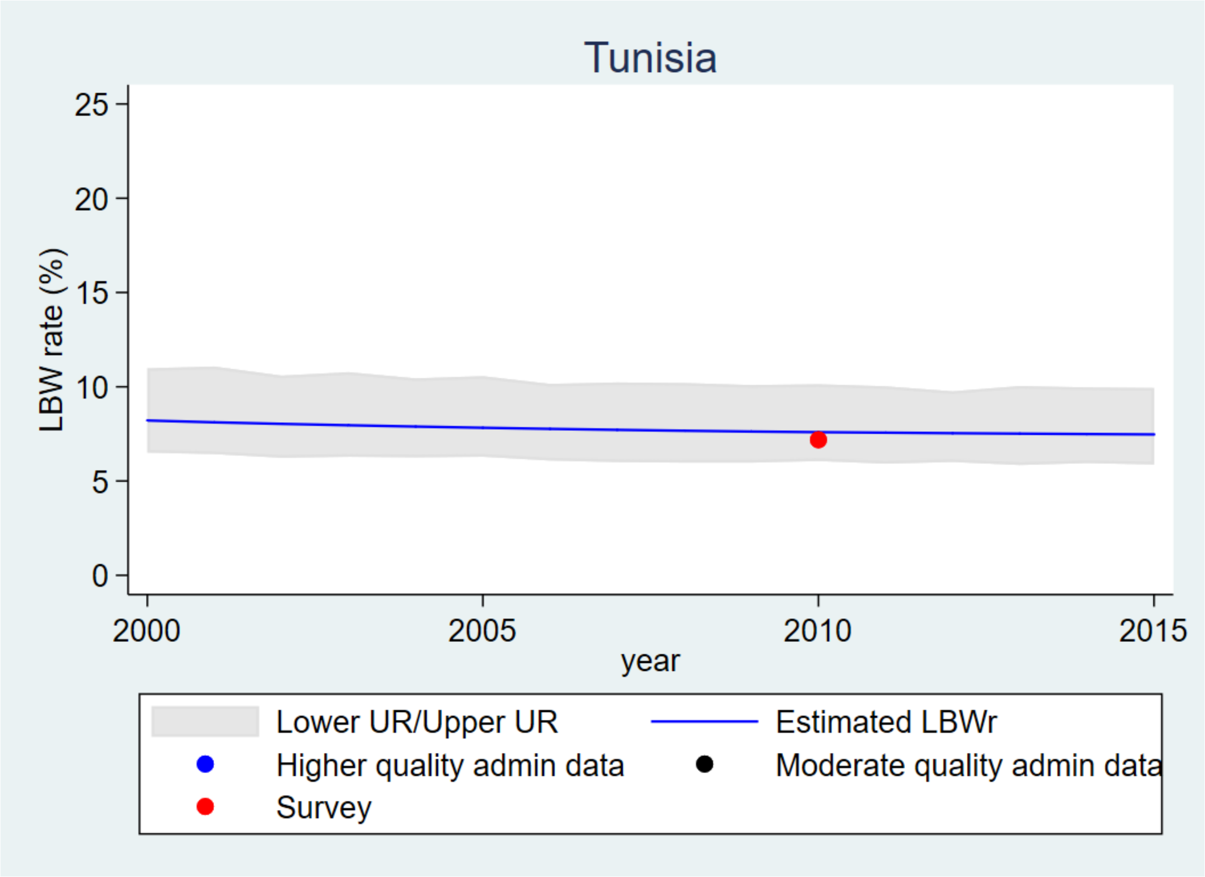

Tunisia
LBW rate (%)
0
5
10
15
20
25
2000
2005
2010
2015
year
Lower UR/Upper UR
Estimated LBWr
Higher quality admin data
Moderate quality admin data
Survey

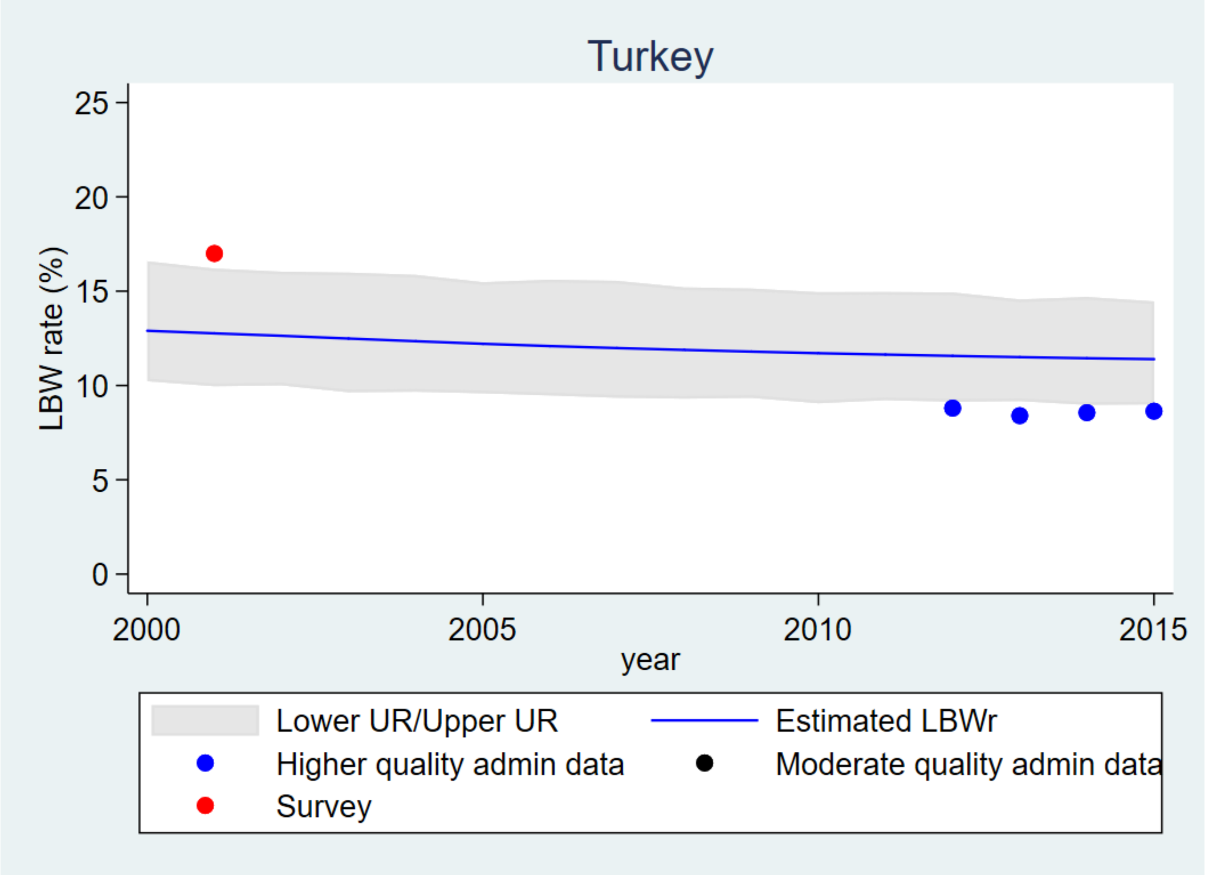

Turkey
LBW rate (%)
0
5
10
15
20
25
2000
2005
2010
2015
year
Lower UR/Upper UR
Estimated LBWr
Higher quality admin data
Moderate quality admin data
Survey

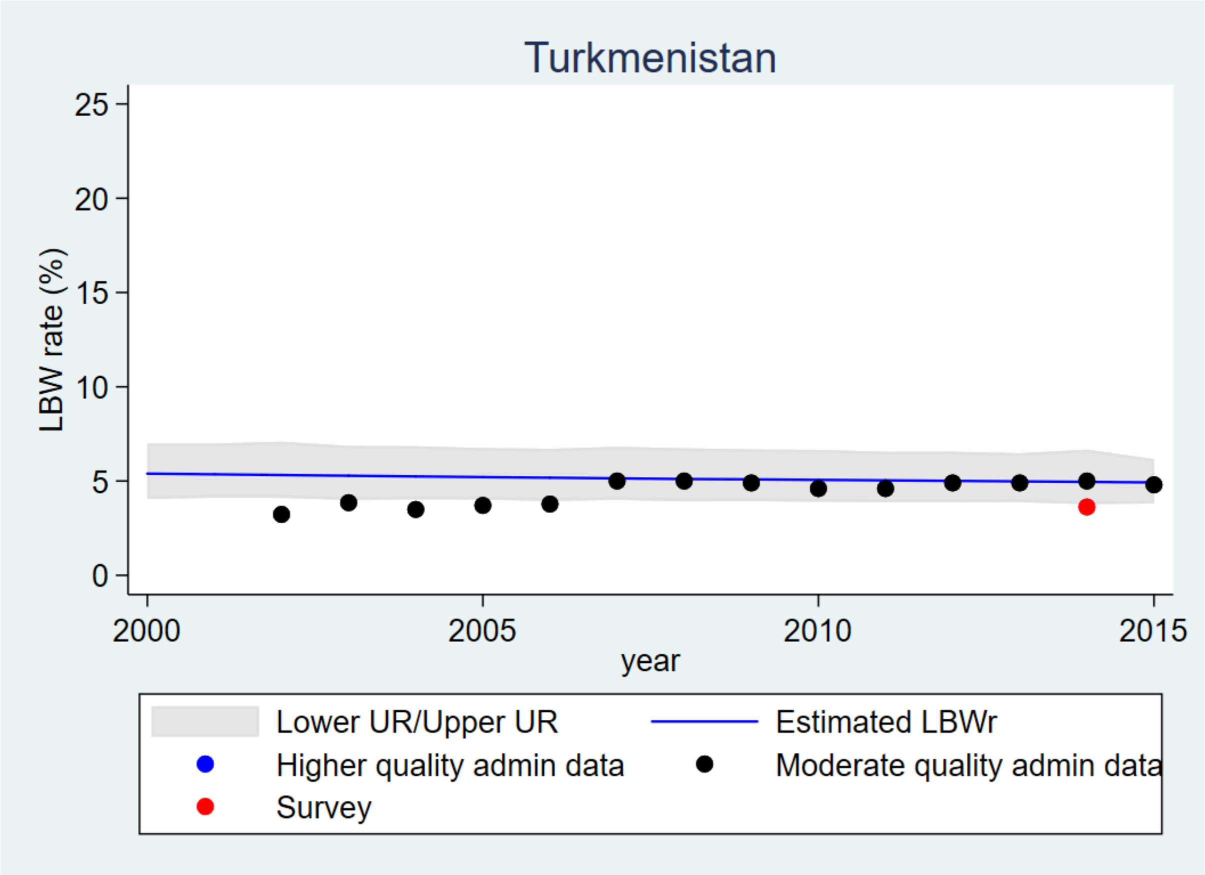

Turkmenistan
LBW rate (%)
25
20
15
10
5
0
2000
2005
2010
2015
year
Lower UR/Upper UR
Estimated LBWr
Higher quality admin data
Moderate quality admin data
Survey

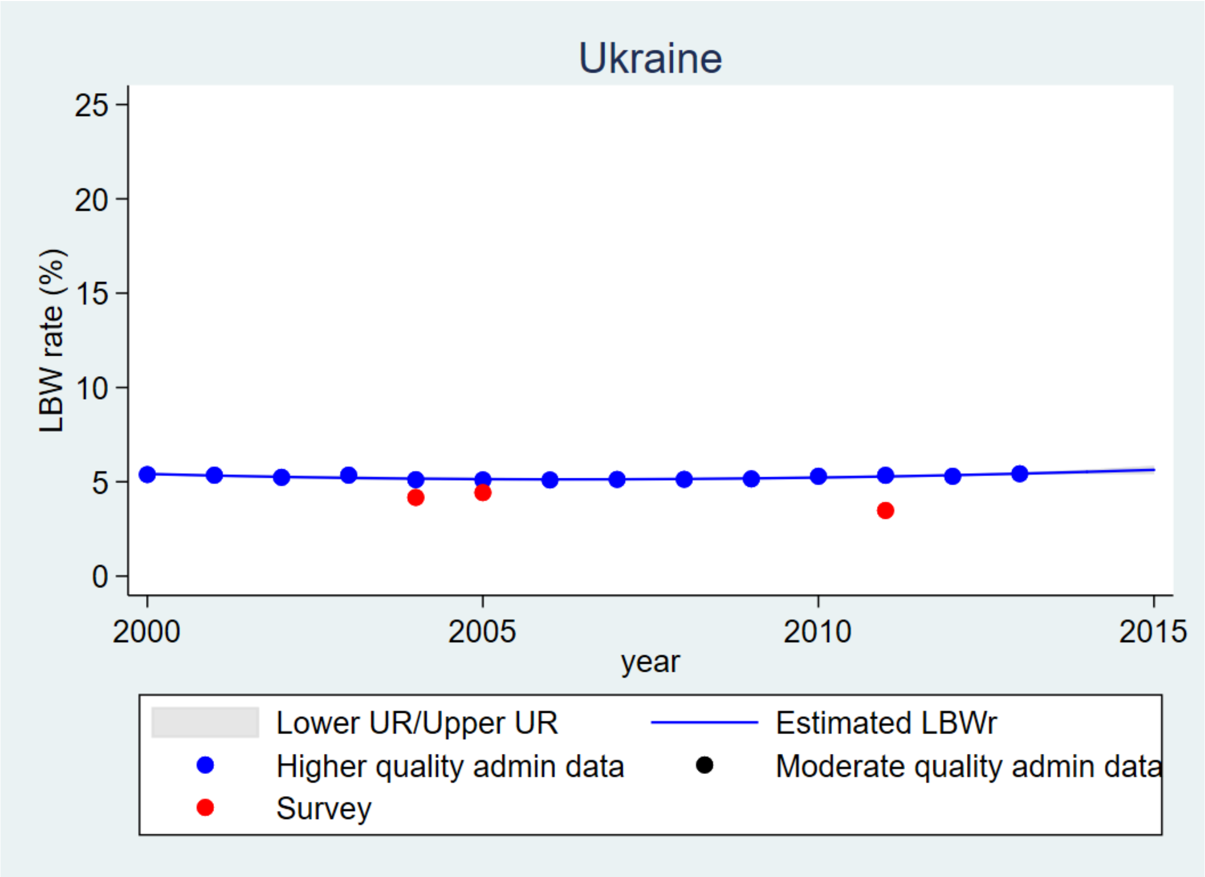

Ukraine
LBW rate (%)
25
20
15
10
5
0
2000
2005
2010
2015
year
Lower UR/Upper UR
Estimated LBWr
Higher quality admin data
Moderate quality admin data
Survey

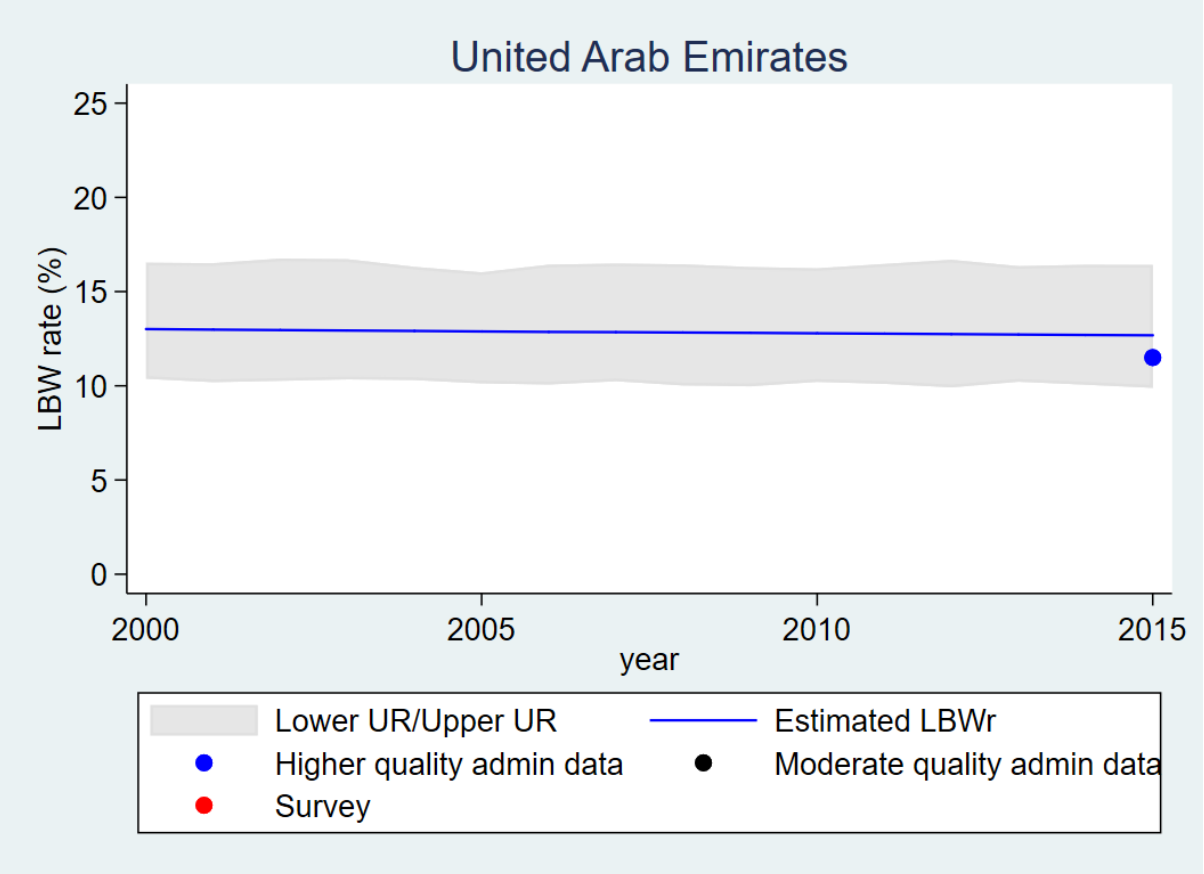

United Arab Emirates
LBW rate (%)
25
20
15
10
5
0
2000
2005
2010
2015
year
Lower UR/Upper UR
Estimated LBWr
Higher quality admin data
Moderate quality admin data
Survey

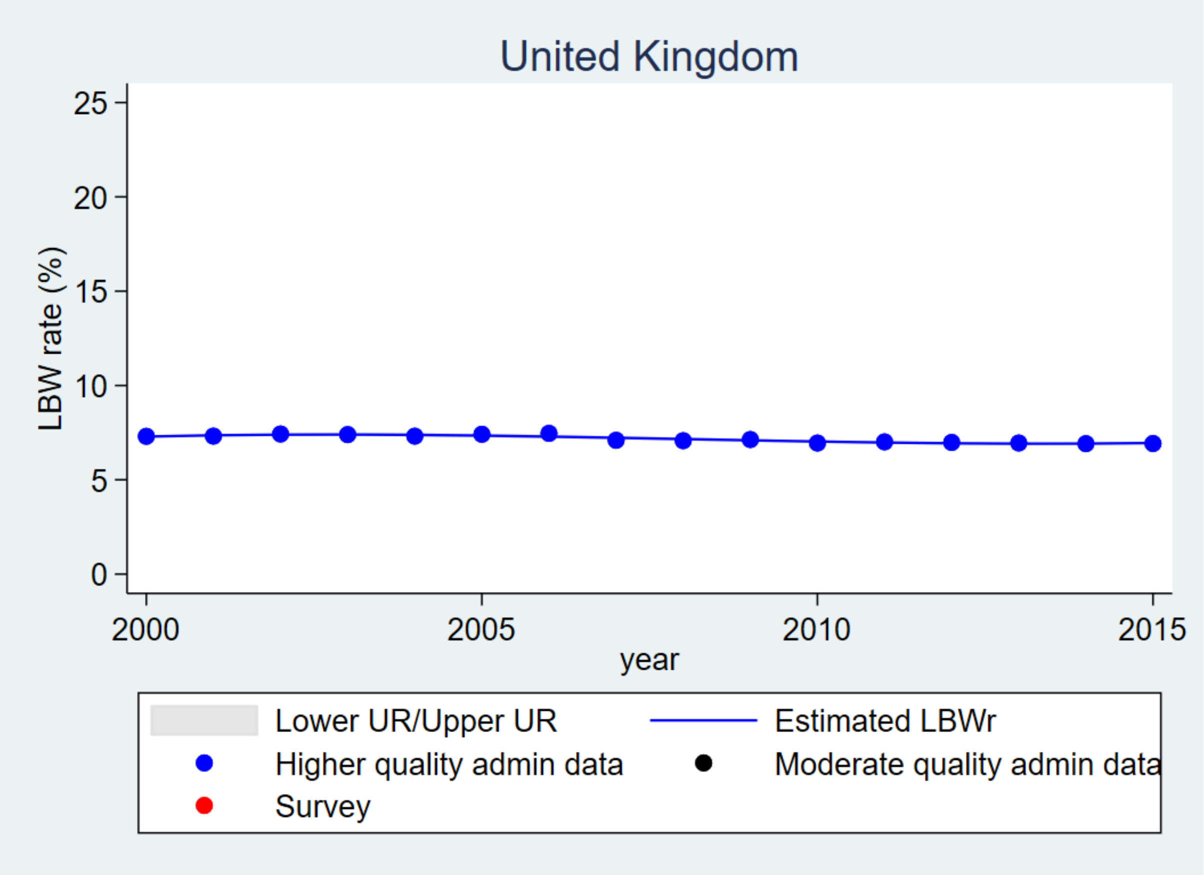

United Kingdom
LBW rate (%)
25
20
15
10
5
0
2000
2005
2010
2015
year
Lower UR/Upper UR
Estimated LBWr
Higher quality admin data
Moderate quality admin data
Survey

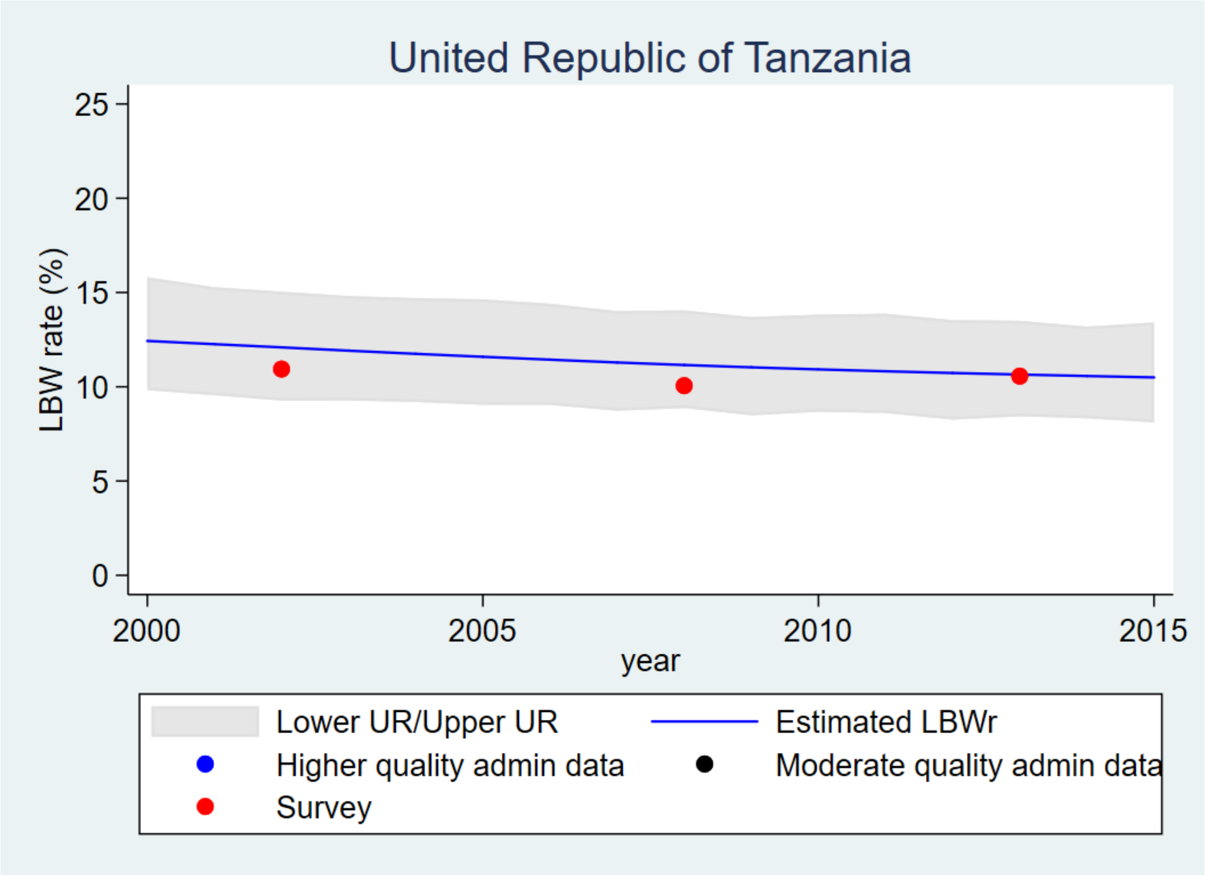
United Republic of Tanzania
25
20
15
10
5
0
LBW rate (%)
2000
2005
2010
2015
year
Lower UR/Upper UR
Estimated LBWr
Higher quality admin data
Moderate quality admin data
Survey

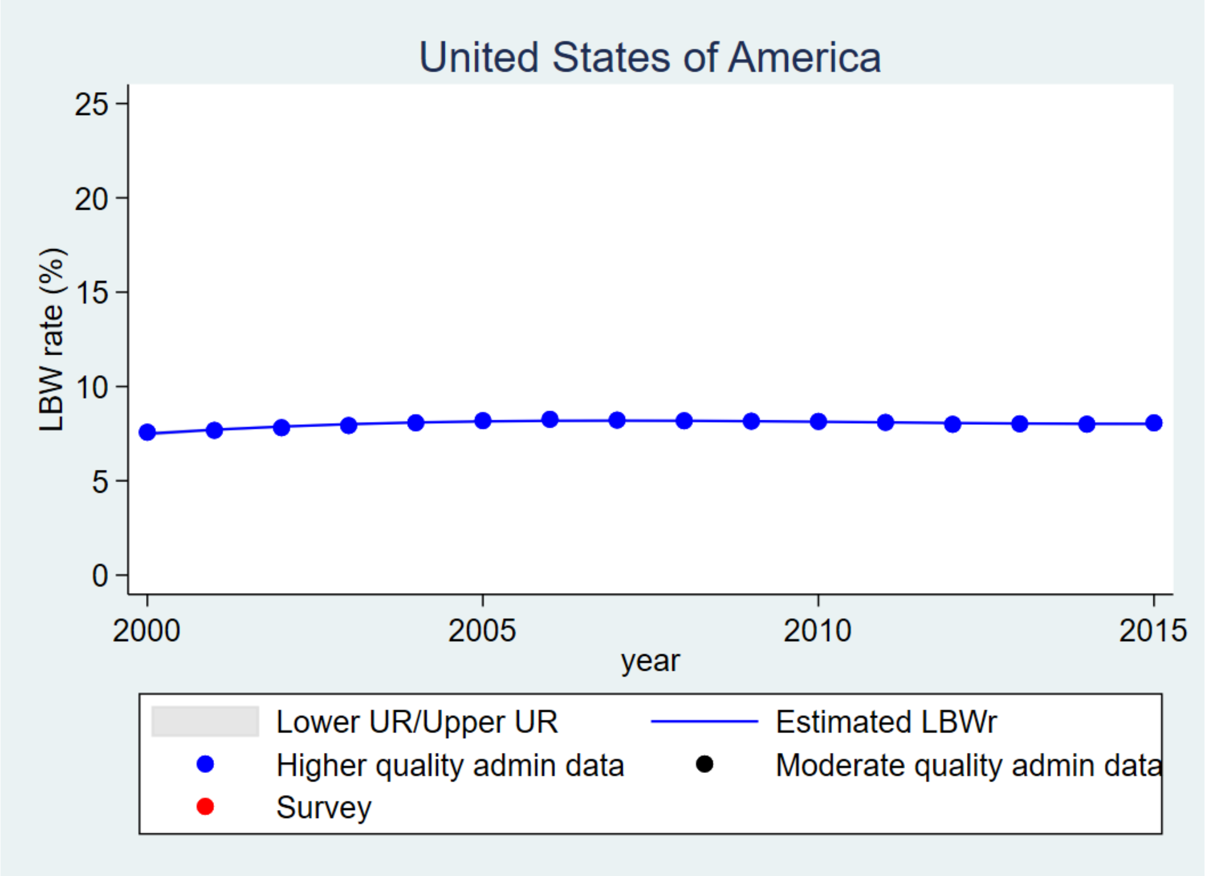
United States of America
25
20
15
10
5
0
LBW rate (%)
2000
2005
2010
2015
year
Lower UR/Upper UR
Estimated LBWr
Higher quality admin data
Moderate quality admin data
Survey

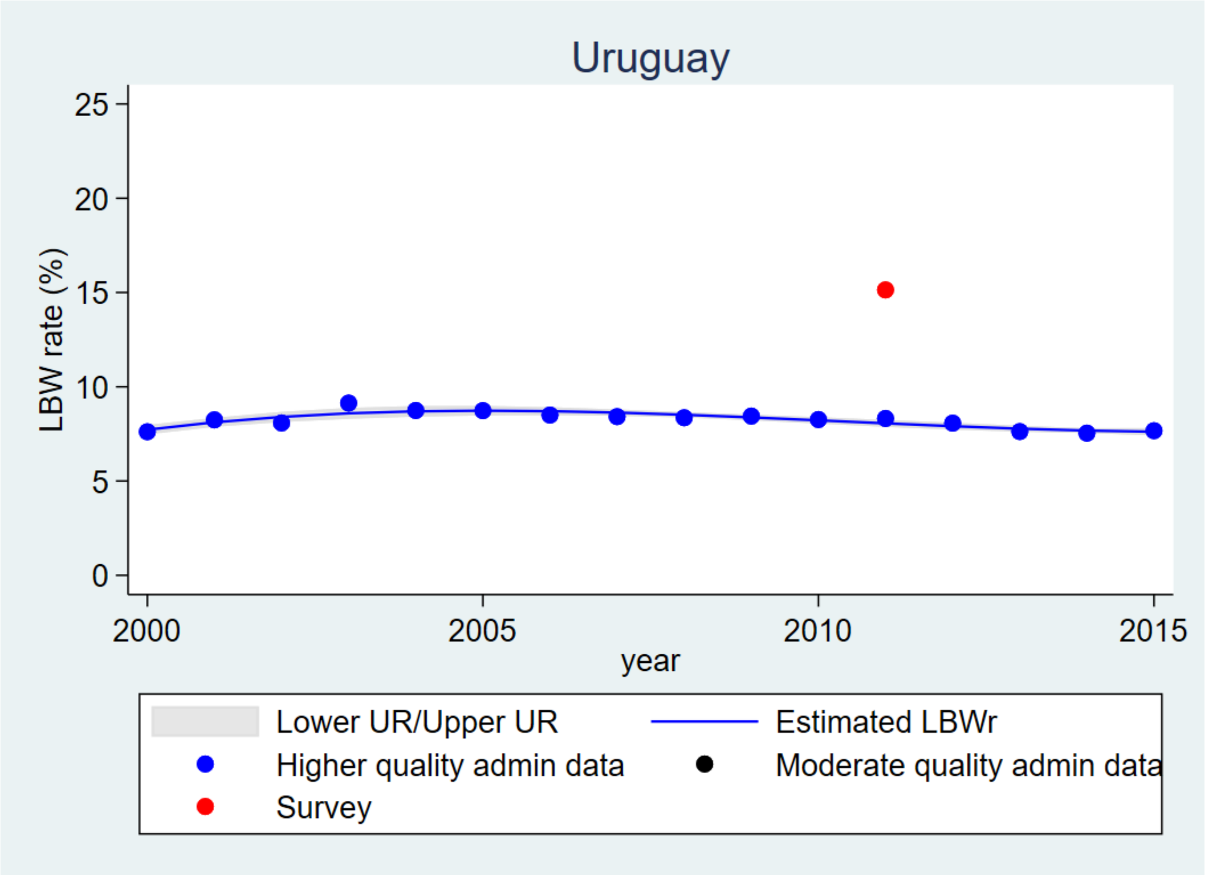
Uruguay
LBW rate (%)
25
20
15
10
5
0
2000
2005
2010
2015
year
Lower UR/Upper UR
Estimated LBWr
Higher quality admin data
Moderate quality admin data
Survey

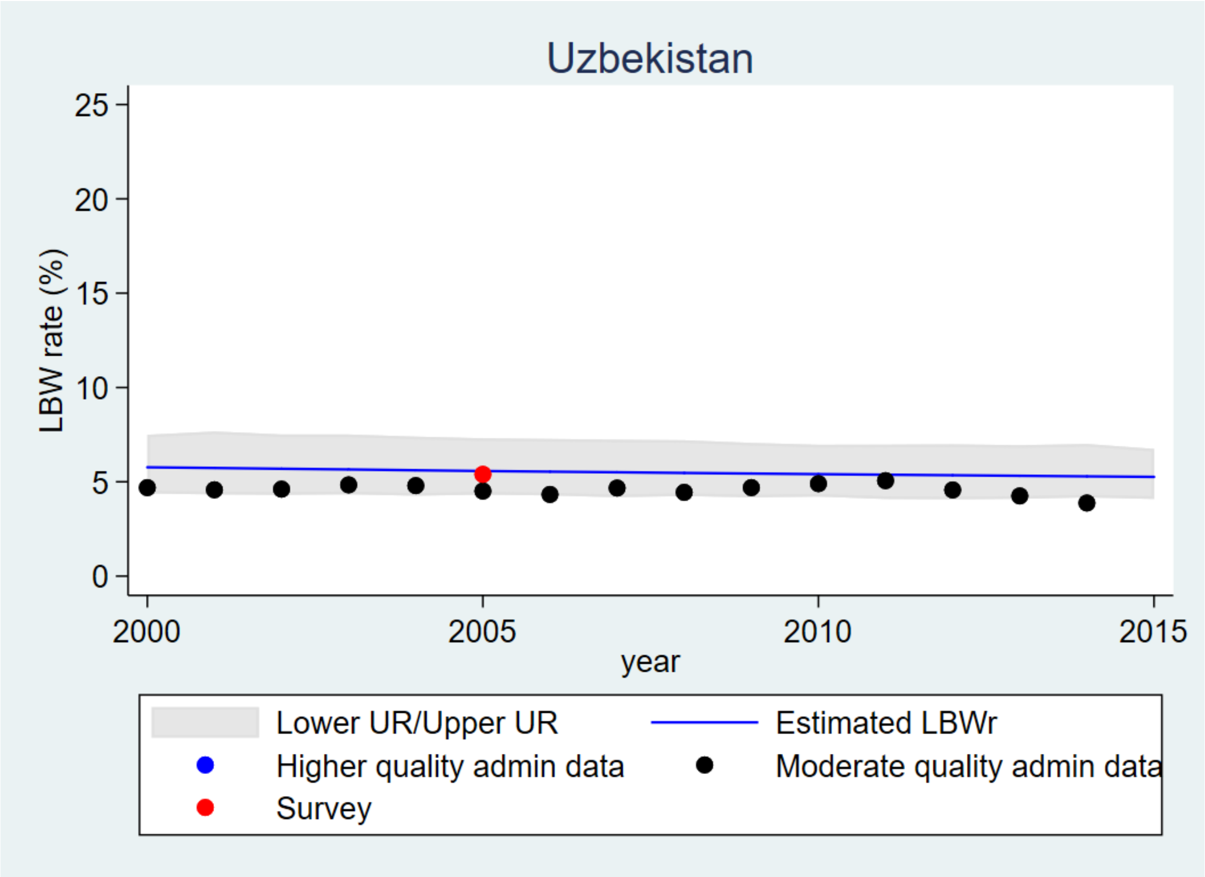
Uzbekistan
LBW rate (%)
25
20
15
10
5
0
2000
2005
2010
2015
year
Lower UR/Upper UR
Estimated LBWr
Higher quality admin data
Moderate quality admin data
Survey

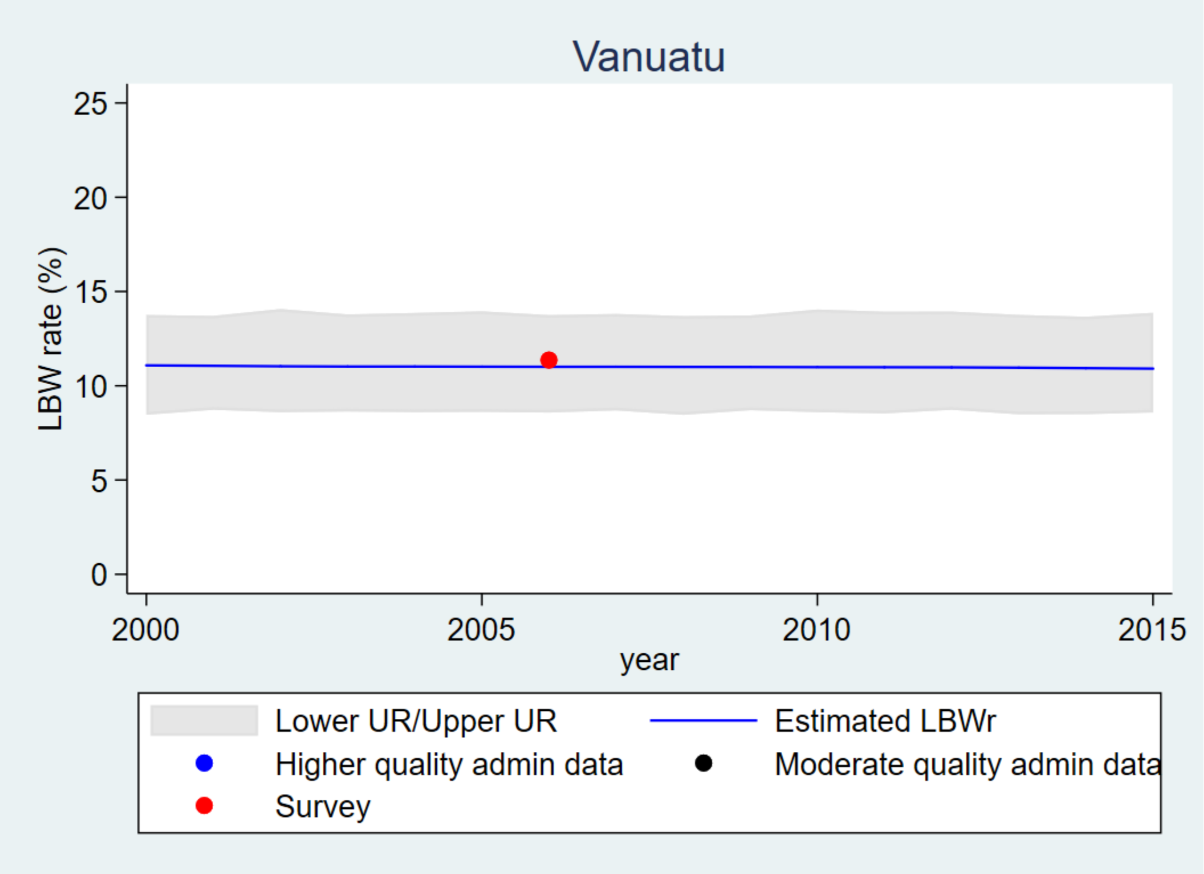
Vanuatu
LBW rate (%)
25
20
15
10
5
0
2000
2005
2010
2015
year
Lower UR/Upper UR
Estimated LBWr
Higher quality admin data
Moderate quality admin data
Survey

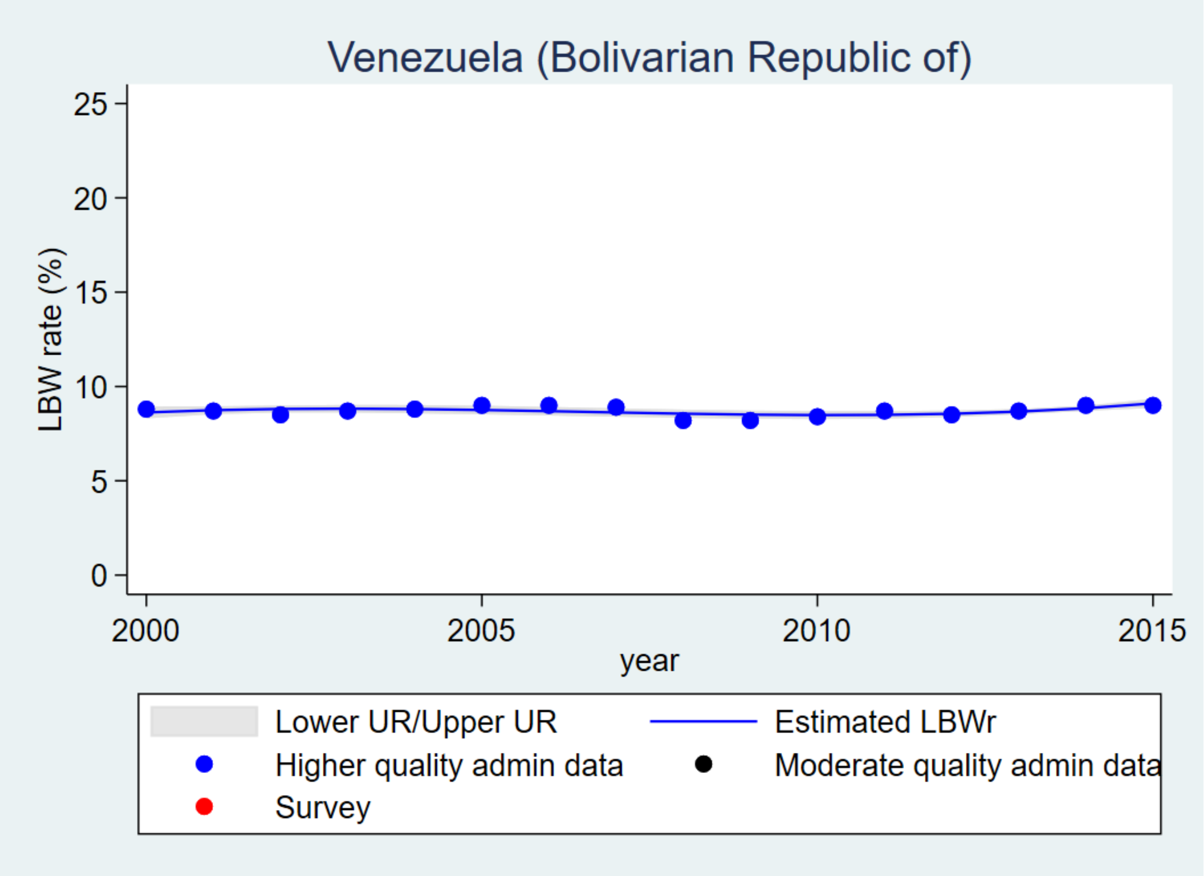
Venezuela (Bolivarian Republic of)
LBW rate (%)
25
20
15
10
5
0
2000
2005
2010
2015
year
Lower UR/Upper UR
Estimated LBWr
Higher quality admin data
Moderate quality admin data
Survey

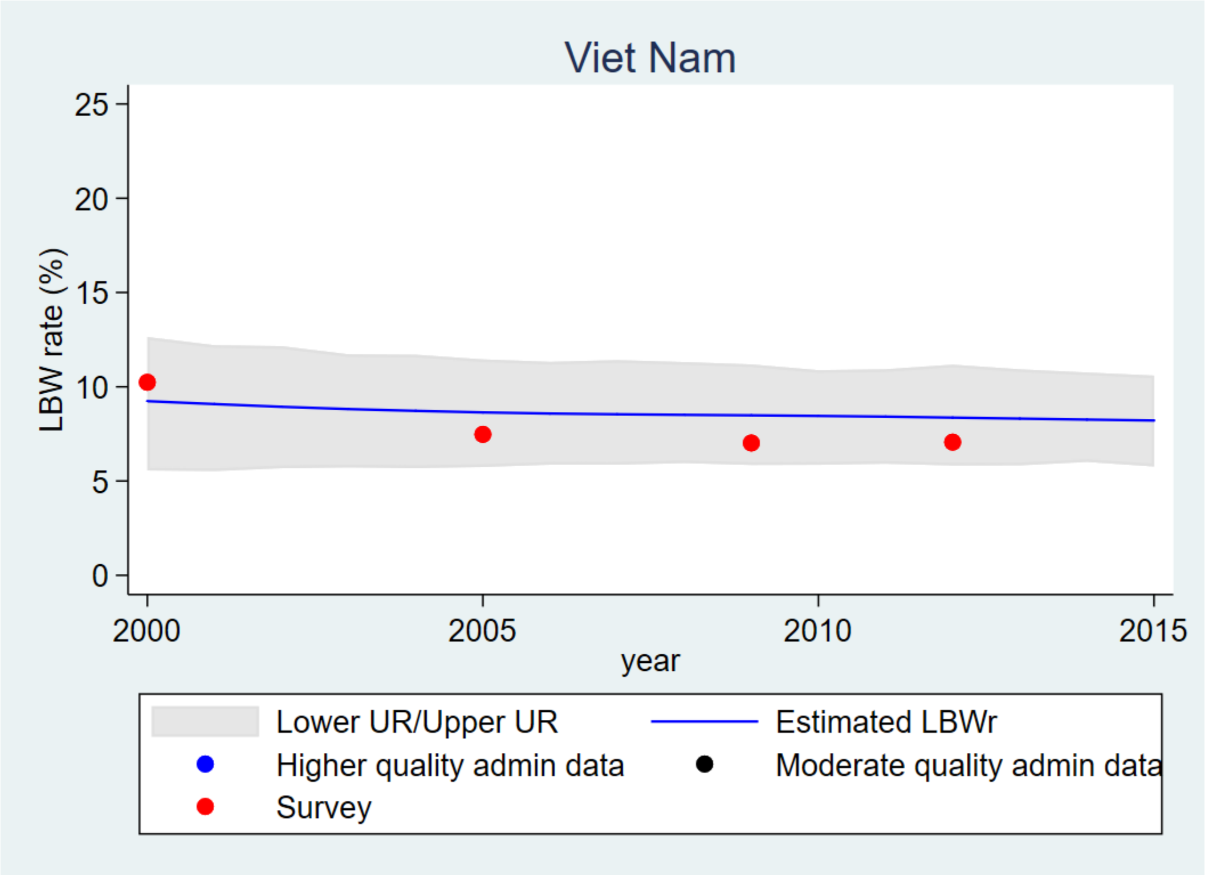
Viet Nam
LBW rate (%)
25
20
15
10
5
0
2000
2005
2010
2015
year
Lower UR/Upper UR
Estimated LBWr
Higher quality admin data
Moderate quality admin data
Survey

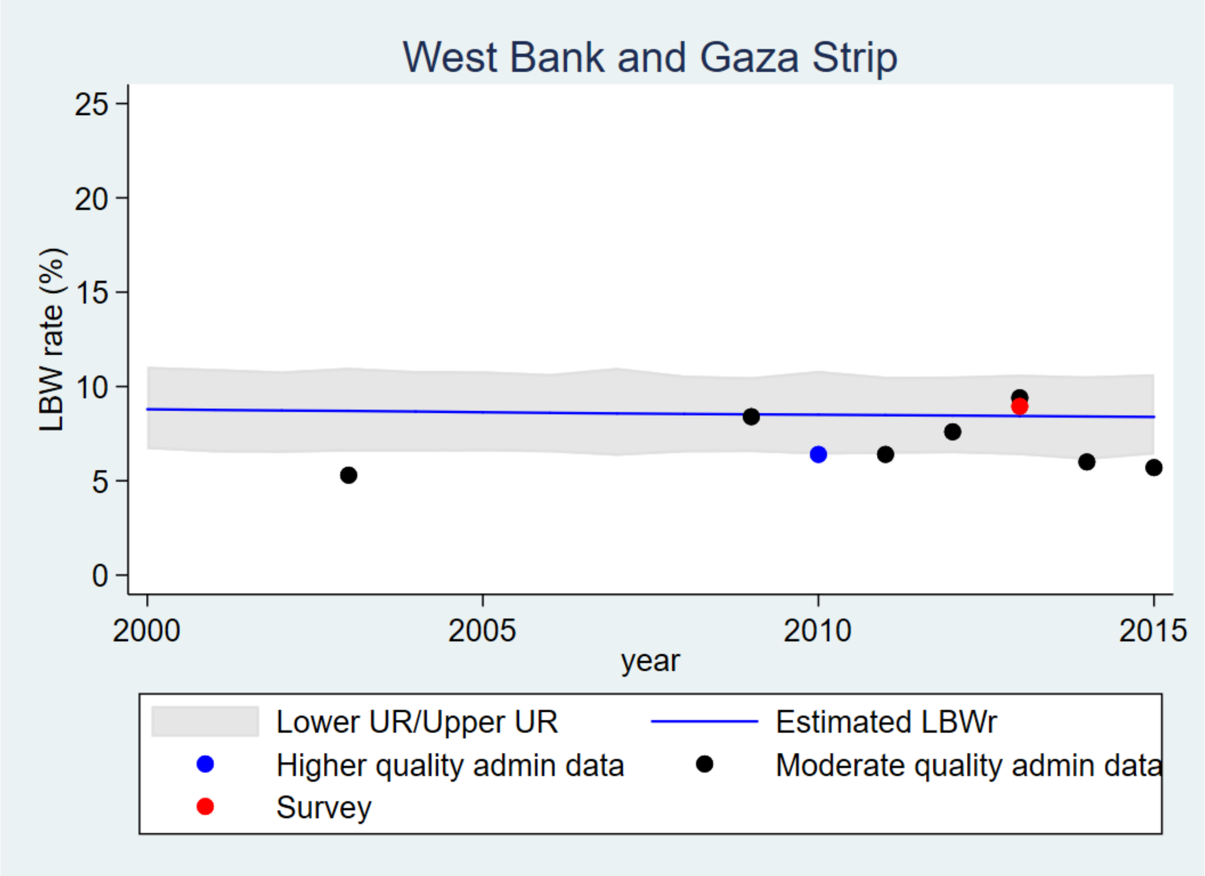
West Bank and Gaza Strip
LBW rate (%)
25
20
15
10
5
0
2000
2005
2010
2015
year
Lower UR/Upper UR
Estimated LBWr
Higher quality admin data
Moderate quality admin data
Survey

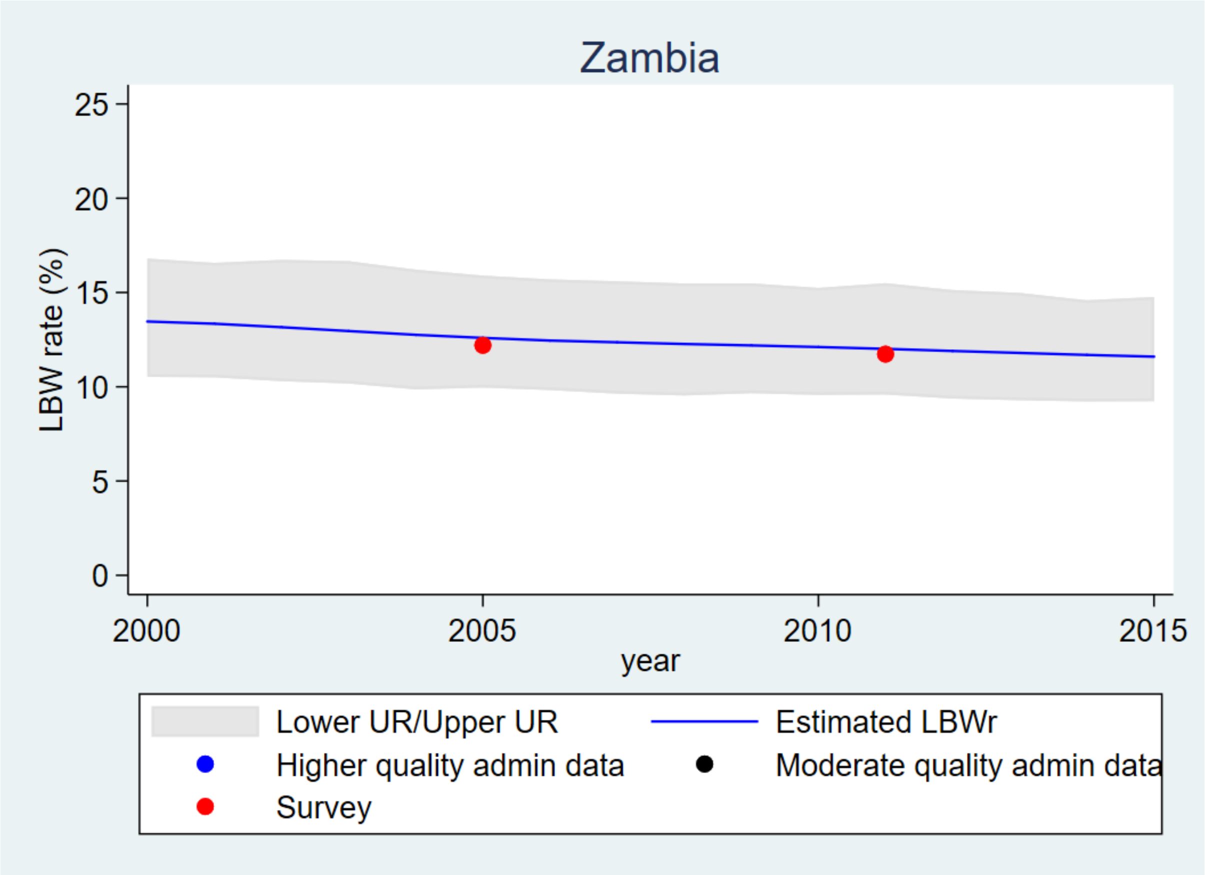
Zambia
LBW rate (%)
25
20
15
10
5
0
2000
2005
2010
2015
year
Lower UR/Upper UR
Estimated LBWr
Higher quality admin data
Moderate quality admin data
Survey

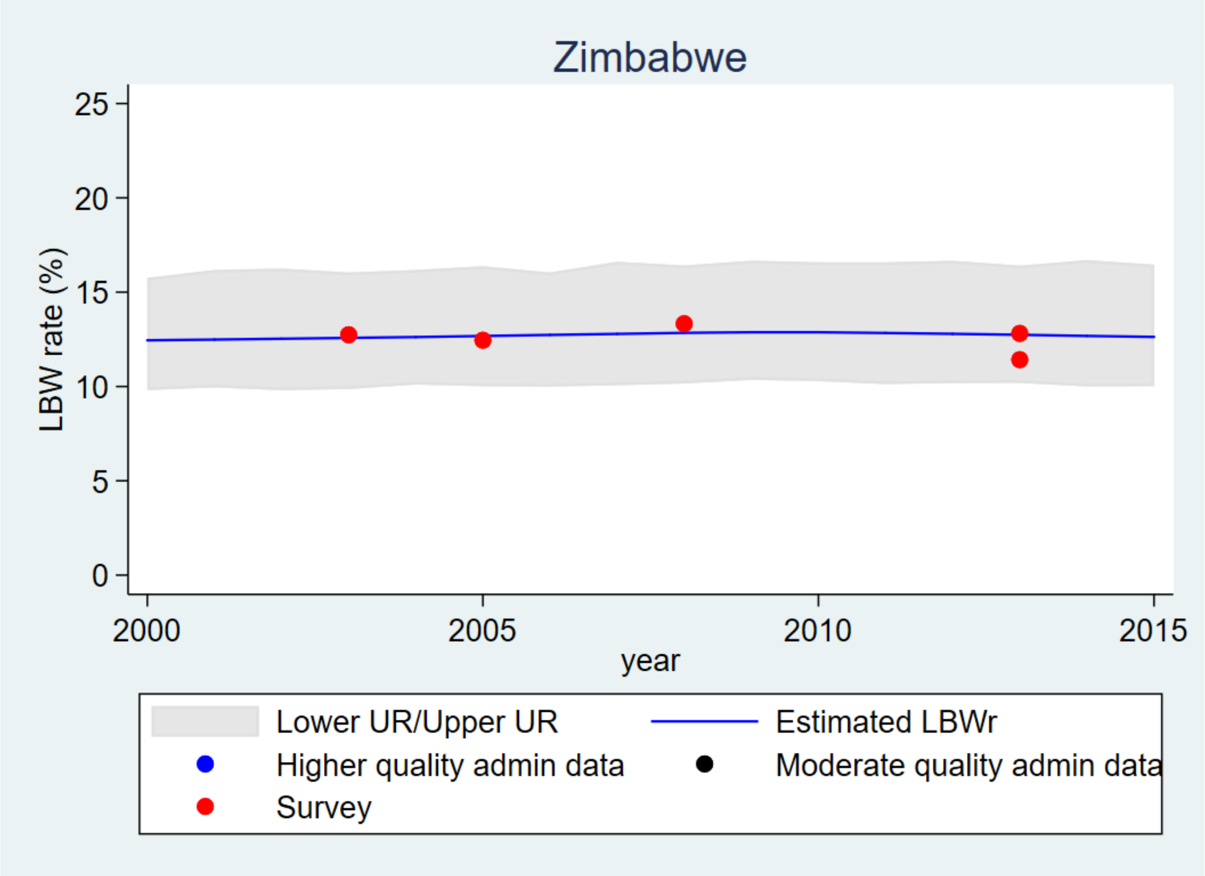
Zimbabwe
LBW rate (%)
25
20
15
10
5
0
2000
2005
2010
2015
year
Lower UR/Upper UR
Estimated LBWr
Higher quality admin data
Moderate quality admin data
Survey

9. ReferencesReferences

1. Stevens, G.A., et al., *Guidelines for Accurate and Transparent Health Estimates Reporting: the GATHER statement.* Lancet, 2016. **388**(10062): p. e19-e23.
2. Phillips, D.E., et al., *A composite metric for assessing data on mortality and causes of death: the vital statistics performance index.* Popul Health Metr, 2014. **12**: p. 14.
3. WHO, et al., *Trends in Maternal Mortality: 1990 to 2013* http://www.who.int/reproductivehealth/publications/monitoring/maternal-mortality-2013/en/, 2014.
4. Blencowe, H., et al., *National, regional, and worldwide estimates of stillbirth rates in 2015, with trends from 2000: a systematic analysis.* Lancet Glob Health, 2016. **4**(2): p. e98-e108.
5. Barro R and Lee JW, *A new Data Set of Educational Attainment in the World, 1950 - 2010.* Journal of development Economics, 2013: p. 184 - 198.
6. Stevens, G.A., et al., *Trends in mild, moderate, and severe stunting and underweight, and progress towards MDG 1 in 141 developing countries: a systematic analysis of population representative data.* Lancet, 2012. **380**(9844): p. 824-34.
7. UN Population Division, *World Population Prospects: the 2017 Revision.* http://esa.un.org/wpp/index.htm, 2017.
